# Trap-induced white circularly polarized persistent luminescence from a host-guest molecular system

**DOI:** 10.1038/s41467-026-76289-7

**Published:** 2026-08-07

**Authors:** Yajing Wang, Cunjian Lin, Yang Li, Chenhan Zhan, Rujun Yang, Zishuang Wu, Shunjie Shu, Wenpeng Ye, Wenting Deng, Jumpei Ueda, Yixi Zhuang, Rong-Jun Xie

**Affiliations:** 1 College of Materials, and Fujian Key Laboratory of Surface and Interface Engineering for High Performance Materials, Xiamen University, Xiamen, China; 2 Graduate School of Advanced Science and Technology, Japan Advanced Institute of Science and Technology, Nomi, Japan; 3 College of Chemistry and Chemical Engineering, Gannan Normal University, Ganzhou, China; 4 Institute of Flexible Electronics (IFE, Future Technologies), Xiamen University, Xiamen, China; 5 Key Laboratory of Flexible Electronics & Institute of Advanced Materials, Nanjing, China; 6 Shenzhen Research Institute of Xiamen University, Shenzhen, China; 7 State Key Laboratory of Physical Chemistry of Solid Surfaces, Xiamen University, Xiamen, China

**Keywords:** Polymers, Single-molecule fluorescence, Electron transfer

## Abstract

Circularly polarized luminescence (CPL), which encodes non-replicable optical information in the characteristics of chirality, wavelength, and time, holds significant promise for advanced information technologies. However, effective strategies for systematically regulating multiple optical properties remain elusive. Herein, we report a general approach that imparts hour-level dual-band persistent luminescence to CPL materials through the introduction of trap states into an organic host-guest molecular system. The electrons released from traps repopulate both the singlet and triplet states of the chiral guest molecules, producing dual-band white circularly polarized persistent luminescence for 4 h at room temperature. Systematic characterization reveals that two different chiral configurations leave the trap depth basically unaltered, while conferring pronounced CPL activity on these composites with a maximum luminescence dissymmetry factor *g*_lum_ of 1.6 × 10^−3^/−2.3 × 10^−3^. Leveraging these characteristics, we present a proof-of-concept demonstration of warm-white circularly polarized persistent luminescence materials for Morse-code encryption and multimodal information storage.

## Abstract (web summary)

Circularly polarized luminescence (CPL) holds significant promise for advanced information technologies but effective strategies for systematically regulating multiple optical properties remain elusive. Here, the authors report a general approach that imparts hour-level dualband persistent luminescence to CPL materials through the introduction of trap states into an organic host-guest molecular system.

## Introduction

Circularly polarized luminescence (CPL) materials are promising optical functional materials capable of emitting different left-handed and right-handed circularly polarized light^1,2^. Their distinctive chiroptical properties open up broad applications in 3D displays^3,4^, information encryption^5–7^, anti-counterfeiting^8,9^, and chiral sensing^10,11^. Among numerous luminescent materials, chiral organic small molecules characterized by low density and wide varieties offer unique advantages for the development of CPL materials^12,13^. They can modify molecular structures by replacing substituents to obtain the desired optical properties, such as emission wavelength, lifetime and luminescence dissymmetry factor (*g*_lum_)^14,15^. It is worth noting that CPL inherently carries additional optical information independent of the general wavelength and intensity^16^. To meet the growing demands of future information technologies for higher-capacity information encoding and enhanced detection sensitivity, research on achieving long-lived luminescence in CPL-active materials has attracted considerable attention^17–20^. Long-lived CPL materials can provide additional time and wavelength information different from steady-state CPL, while eliminating the influence of short-lived background fluorescence and non-polarized light^21,22^. Still, it remains a critical challenge to improve the luminescence lifetime of CPL materials.

Over the past few decades, significant progress has been made in the field of organic luminescence. A variety of strategies, including host-guest doping^23,24^, crystallization engineering^25,26^, and polymerization^27,28^, have been exploited, which greatly enhance the luminescence lifetime and diversify the material systems. These strategies have indeed proven effective in prolonging the lifetime of CPL^29–33^. Nevertheless, due to the nature of the spin-forbidden transition between the triplet and singlet states, the long-lived CPL reported to date remain confined to the millisecond-to-second range. A milestone was reached by Kabe and Adachi in 2017 by extending the lasting time for hours based on a charge separation mechanism in exciplex systems^34^. Through rational design of donor/acceptor molecules and the incorporation of other emitters, the emission wavelength and polarization properties could be tailored^35–38^. Our group recently reported that the charge separation process and long-lasting emission in host-guest two-component persistent luminescent (PersL) materials were likely related to trap states in organics^39^. Exploiting this mechanism, an hour-long PersL tunable from blue, to red and near-infrared (NIR) region has been realized by designing different emitters^40,41^.

The obtained afterglow colors remain mostly confined to yellow, green, and blue owing to the intricate chiral transfer between the CPL-active chiral units and the PersL emission group in a molecule^42–47^. However, it is rather difficult to develop white CPL materials that show long-lasting emission covering the entire visible light spectrum. Traditionally, white phosphor is produced by mixing complementary emissions from multiple luminescent centers^48–50^. This route requires intricate control of each component and the precise matching of chirality, lifetime, and intensity across all of them, which greatly complicates the application of materials. Alternatively, white PersL can be achieved through combining singlet-singlet and triplet-singlet transitions from a single luminescent center^51–53^. However, the resulting emission could only be observed in a short time, and it shows a significant color shift over time, as the singlet state intrinsically decays much faster than the triplet state. At present, there is still a lack of a universal strategy to achieve white PersL with an hour-long duration and stable color.

**Fig. 1:**
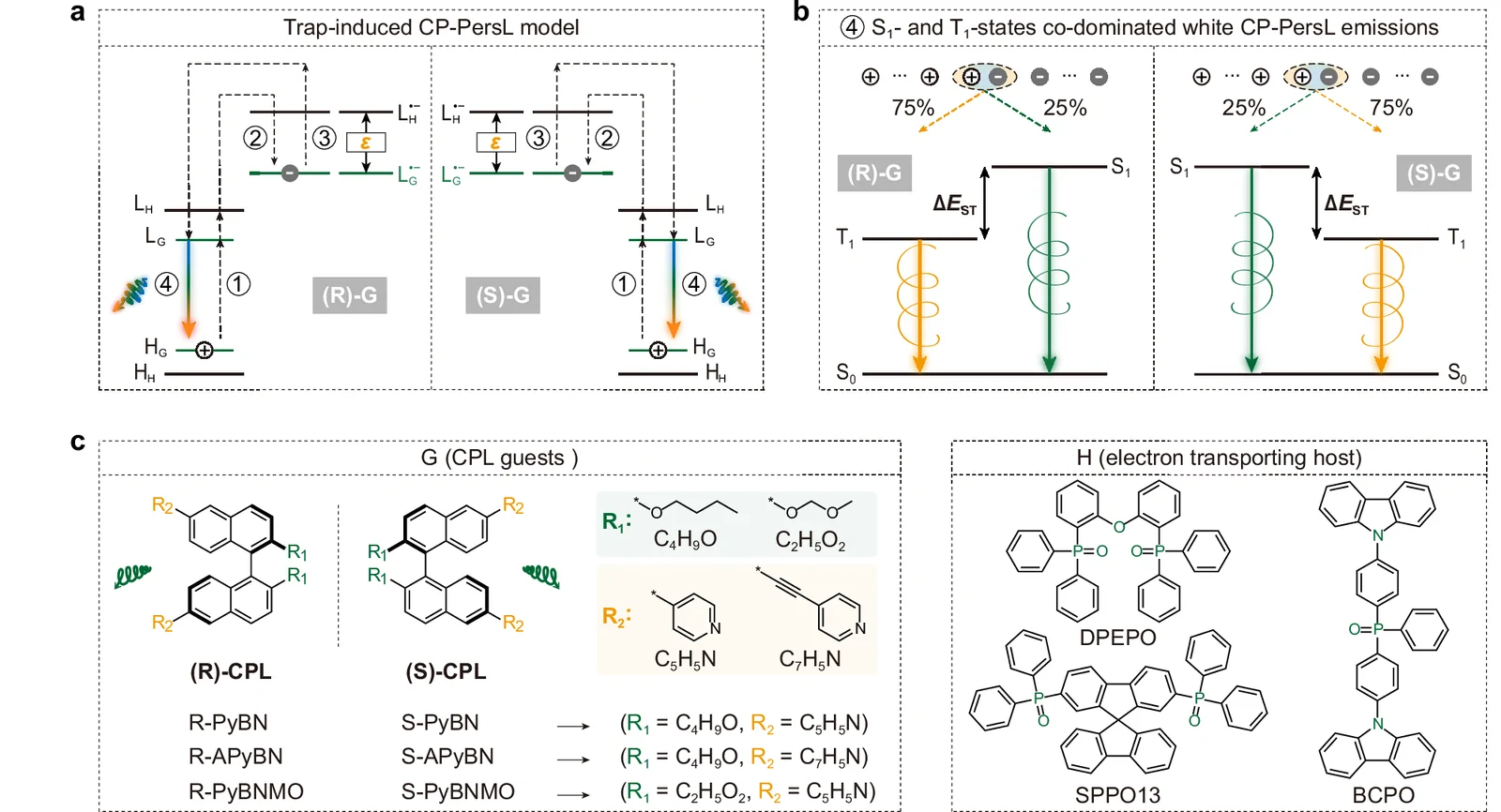
Molecular design to achieve dual-band white trap-induced CP-PersL in a host-guest molecular system. **a** Energy level model for trap-induced CP-PersL emissions. Under light excitation, charge carriers from chiral luminescent centers are excited (①) and captured by traps (②), forming guest radical anions (guest^•−^). The trapped charge carriers are detrapped by thermal stimulation or photo-stimulation (③) and subsequently recombine at the luminescent centers to give PersL (④). **b** Proposed mechanism of S_1_- and T_1_-states co-dominated dual-band white CP-PersL emissions. **c** Chemical structure of chiral molecules as guest (left) and host molecules (right). Three couples of chiral guests and three host molecules were studied in this work, including R/S-4,4’-(2,2’-dibutoxy-[1,1’-binaphthalene]−6,6’-diyl)dipyridine (R/S-PyBN), R/S-4,4’-((2,2’-dibutoxy-[1,1’-binaphthalene]−6,6’-diyl)bis(ethyne-2,1-diyl))dipyridine (R/S-APyBN), R/S-4,4’-(2,2’-bis(methoxymethoxy)-[1,1’-binaphthalene]−6,6’-diyl)dipyridine (R/S-PyBNMO), bis[2-(diphenylphosphino)phenyl] ether oxide (DPEPO), bis-4-(N-carbazolyl)phenyl)phenylphosphine oxide (BCPO), and 2,7-bis(diphenylphosphoryl)−9,9’-spirobi[fluorene] (SPPO13). Abbreviations and labels: trap depth (*ε*), electrons (gray balls), holes (white balls), the lowest unoccupied molecular orbital (LUMO) of the host (L_H_), the highest occupied molecular orbital (HOMO) of the host (H_H_), LUMO of the guest (L_G_), HOMO of the guest (H_G_), LUMO of the host radical anion (L_H_^•−^), and LUMO of the guest radical anion (L_G_^•−^).

In this work, we report dual-band white circularly polarized PersL (CP-PersL) emitters by constructing trap states in a host-guest molecular system. Axially chiral conjugated binaphthyl derivatives are used as guests, and molecules with fast electron mobility are used as hosts. The target trap depth, which is supposed to be the energy difference of two orbitals between the hosts and guests, is yielded by deliberate molecule selection (Fig. 1a). Dual-band white PersL is achieved by releasing trapped electrons that populate both the singlet (S_1_) and triplet (T_1_) states of guest molecules characterized by a large singlet–triplet energy gap (Δ*E*_ST_) (Fig. 1b). The composites retain the CPL properties, yielding a maximum luminescence dissymmetry factor *g*_lum_ of 1.6 × 10^-3^/-2.3 × 10^-3^. Leveraging these properties, we further explore their applications in Morse code encryption and multimodal information storage to improve the security and reliability of optical information as a conceptual demonstration.

## Results

### Characterization of R/S-PyBN and DPEPO

The guest molecules used in this work are prepared through the straightforward synthetic routes as outlined in Supplementary Figs. 1–4. We first take R/S-4,4’-(2,2’-dibutoxy-[1,1’-binaphthalene]−6,6’-diyl)dipyridine (R/S-PyBN)@bis[2-(diphenylphosphino)phenyl] ether oxide (DPEPO) composite (hereinafter named as R/S-PyBN@DPEPO) as a model material in the study (Fig. 1c). DPEPO serves as the host molecule because of its superior electron transport ability. The guests R/S-PyBN, categorized as donor-conjugate-acceptor (DπA)-type room-temperature phosphorescence (RTP) emitters, were synthesized as described in Supplementary Fig. 1. The guest molecules not only provide two different chiral configurations but also promote the intersystem crossing (ISC) process to generate triplet electrons through the coupling effect of two conjugated naphthalene units.

For R/S-PyBN, blue fluorescence emission was observed in the toluene solution (5×10^-5 ^mol/L), powder and neat film (Supplementary Figs. 5–7). Compared with the well-resolved structured emission of isolated molecules in toluene solution, the fluorescence of R/S-PyBN in powders and films featured a broad emission band with a pronounced red shift, which could be attributed to aggregation forms with stronger intermolecular interactions^54,55^. Meanwhile, the phosphorescence spectra of R/S-PyBN in both the powder and film forms displayed clear vibronic fine structures at 77 K, which are characteristics of radiative transitions from the local excited state (LE) excitons^56,57^. Low-temperature steady-state fluorescence and phosphorescence spectra verified that the R/S-PyBN films exhibited large energy gaps Δ*E*_ST_ of approximately 0.52/0.52 eV. At room temperature (RT), the fluorescence (with one peak at 483/483 nm) decayed much faster than the phosphorescence (with two peaks at 562/560 and 606/606 nm), confirming the RTP character (Supplementary Fig. 8 and Supplementary Table 1). In contrast, DPEPO, irrespective of in powder or neat film form, exhibited nearly identical fluorescence and delayed fluorescence bands either at 77 K or at RT (Supplementary Fig. 9). At 77 K, the emission band of DPEPO film is peaked at approximately at 465 nm with a fluorescence lifetime of 215.31 ms (Supplementary Fig. 10 and Supplementary Table 1). Furthermore, cyclic voltammetry (CV) analysis shows that the LUMO and HOMO of R/S-PyBN are positioned at −2.52/−2.52 eV and −5.62/−5.62 eV, respectively. They are sandwiched between the LUMO (−2.04 eV) and HOMO (−6.44 eV) of DPEPO. This is different from the interlaced energy-level structure of the typical exciplex systems. (Supplementary Fig. 11)^58^.

### Photophysical properties of R/S-PyBN@DPEPO

**Fig. 2:**
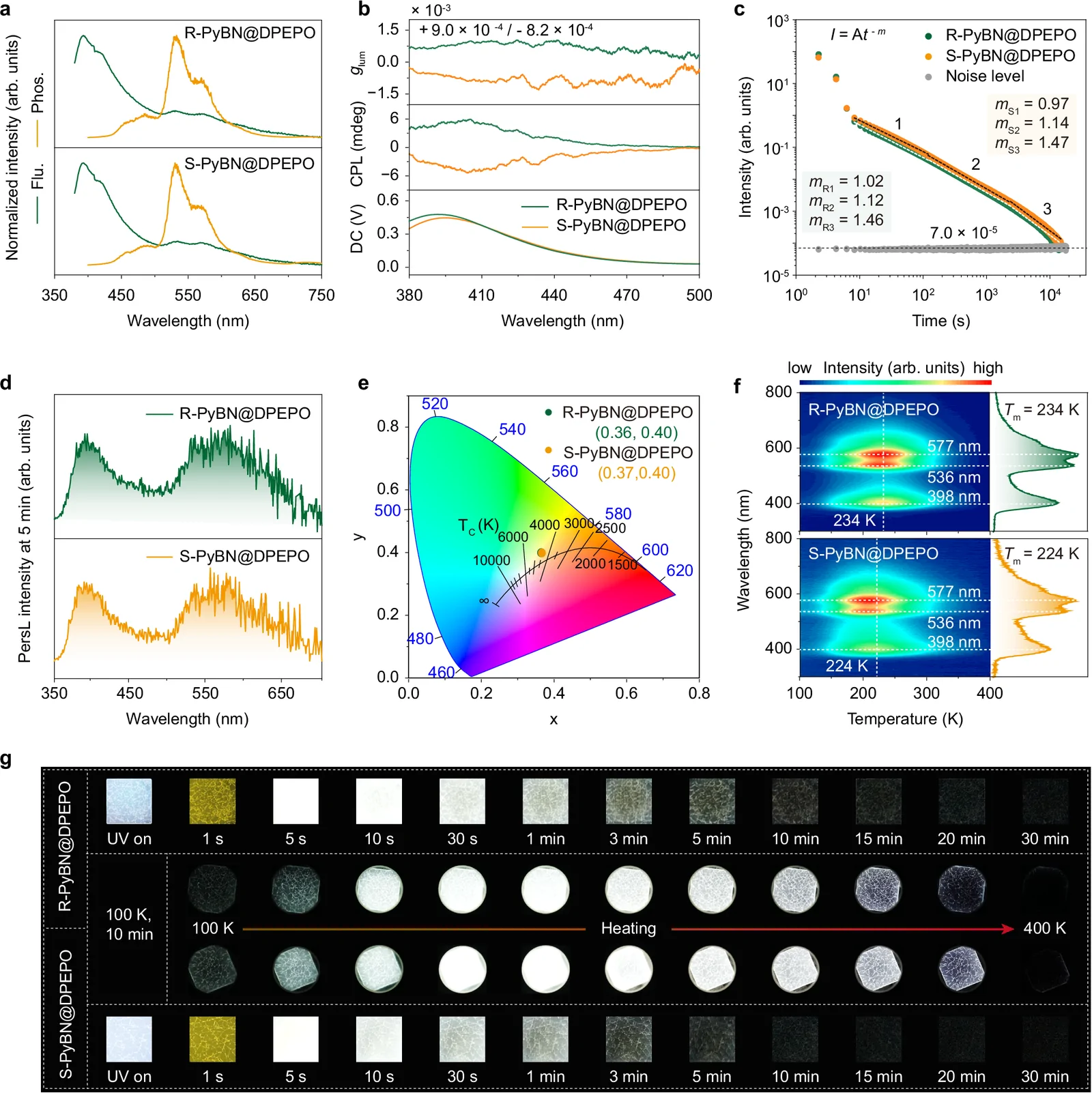
Photophysical properties of the R/S-PyBN@DPEPO. **a** Steady-state fluorescence (Flu., green curves) and phosphorescence spectra (Phos., orange curves, delay time 8 ms). **b** *g*_lum_, CPL, and direct current (DC) spectra under excitation of 340 nm. **c** PersL decay curves at RT after UV irradiation for 60 s. The noise level of the detector is also given in gray dots. **d** PersL spectra at RT after UV irradiation (365 nm) for 60 s. The PersL spectra were recorded with a delay time of 5 min. **e** CIE chromaticity diagram of the PersL obtained at 5 min. **f** Intensity-wavelength-temperature TL spectra (3D-plot) with a heating rate of 50 K min^-1^. The emission spectra are extracted from the 3D-plot and given in the right side. **g** Photographs of the samples to show PersL decay at RT and TL. The samples were irradiated with UV light for 60 s before measurements. The PersL images of the two samples were recorded after a delay time from 1 s to 30 min, while the TL images were collected during heating from 100 to 400 K. Camera settings: exposure time 1/1000 s, ISO 400 for UV on and 1 s; exposure time 2.5 s, ISO 40000 for PersL decay; exposure time 1 s, ISO 8000 for TL process.

The R/S-PyBN@DPEPO films were prepared by the conventional melt-casting method under a nitrogen atmosphere at 320 °C. The optimal doping ratio of the guest/host composites was determined to be 1 wt% based on the measurements of PersL decay curves (Supplementary Fig. 12). Thermogravimetric analysis (TGA) demonstrated that the R/S-PyBN@DPEPO films (1 wt%, unless otherwise specified) did not undergo obvious thermal decomposition under a nitrogen atmosphere, with their decomposition temperatures (*T*_d_, 10% weight loss) of 370/378 °C, exceeding the preparation temperature of 320 °C (Supplementary Fig. 13). In contrast to DPEPO film, the absorption and emission spectra of the R/S-PyBN@DPEPO films closely resembled those of pure R/S-PyBN films (Supplementary Fig. 14). The absorption spectra of R/S-PyBN@DPEPO showed a primary absorption band in the range of 300-400 nm corresponding to the π-π* transitions of the guests^59^. Under excitation of 365 nm, the R/S-PyBN@DPEPO predominantly exhibited blue fluorescence emissions peaked at ~393/393 nm, with relatively weak contributions by triplet excited states (Fig. 2a). The luminescence quantum yields (QY) of R/S-PyBN@DPEPO films were 38.4/39.8% under ambient condition, indicating the existence of a rigid environment provided by the host matrix. Notably, the phosphorescence spectra of the R/S-PyBN@DPEPO films retained vibronic fine structures consistent with those of the neat guest films, showing two emission peaks at 531/531 and 572/572 nm. The corresponding lifetimes measured at RT under microsecond pulsed-lamp excitation were 701.33/677.07 ms and 762.13/716.39 ms, respectively, as determined from exponential fits by the time required for the emission intensity to decrease to 1/e of its initial value (Supplementary Fig. 15 and Supplementary Table 2). Similar phosphorescence features were observed in 1 wt% R/S-PyBN@PMMA, further supporting that the emission remained dominated by the guest molecules (Supplementary Fig. 16). We note that, owing to the weakened aggregation effect caused by the dispersion of guest molecules in the host-guest system, both the fluorescence and phosphorescence emissions of the R/S-PyBN@DPEPO films showed a blue shift compared to those of pure R/S-PyBN film^60,61^. Meanwhile, the R/S-PyBN@DPEPO exhibited CPL features with a luminescence dissymmetry factor (*g*_lum_) of 9.0 × 10^-4^/-8.2 × 10^-4^ (Fig. 2b). The circular dichroism (CD) spectra with opposite Cotton effects confirmed that the intrinsic axial chirality of binaphthyl in the guests was preserved in the composites (Supplementary Fig. 17).

The R/S-PyBN@DPEPO exhibited long-lasting emissions after continuous excitation for 1 min, which could be detected with a photomultiplier tube (PMT) for over 4 h before the signal attenuated to the noise level of detector (Fig. 2c). The emission intensity followed an exponential decay in the first several seconds and then obeyed an inverse power-law dependence on decay time (*I* = A*·t*^*-m*^ *or lgI = lg*A *- m·lgt*). Such power-law decay (*m* = 0.1-2) is explained as the long-range charge separation and recombination process according to the Debye-Edwards law^34,37,62^. Notably, the time-resolved spectra revealed the evolution of singlet and triplet contributions in the entire emission processes (Fig. 2d and Supplementary Fig. 18). The emission spectra recorded in the range of 1 ms to 5 s were predominantly governed by the triplet states. In contrast, the relative contribution of singlet states increased substantially after 5 s (from 5 s to 60 min), reaching an intensity comparable to that of triplet emissions. These results indicate that, in the PersL process, electrons are populated into the S_1_ and T_1_ states, causing both singlet-singlet and triplet–singlet transitions to generate long-lasting PersL. The resultant dual-band PersL spectra covered complementary blue and yellow regions, yielding warm-white PersL with Commission International de l’Eclairage (CIE) coordinates of (0.36, 0.40)/(0.37, 0.40) (Fig. 2e).

We further recorded the thermoluminescence (TL) spectra in an intensity-wavelength-temperature (3D-plot) mode. After charging the samples at 100 K for 5 min, both the singlet-singlet and triplet-singlet emissions were observed in the whole temperature range from 100 to 380 K, confirming that the emissions always came from the guests (Fig. 2f and Supplementary Fig. 19). Finally, we took photographs to visualize the PersL and TL of the R/S-PyBN@DPEPO. As depicted in Fig. 2g, the two samples showed almost the same warm-white emissions after removal of the ultraviolet (UV) light or heating from 100 to 400 K, indicating that the chirality of the guests had little influence on the PersL.

### CP-PersL and trap characterizations of R/S-PyBN@DPEPO

**Fig. 3:**
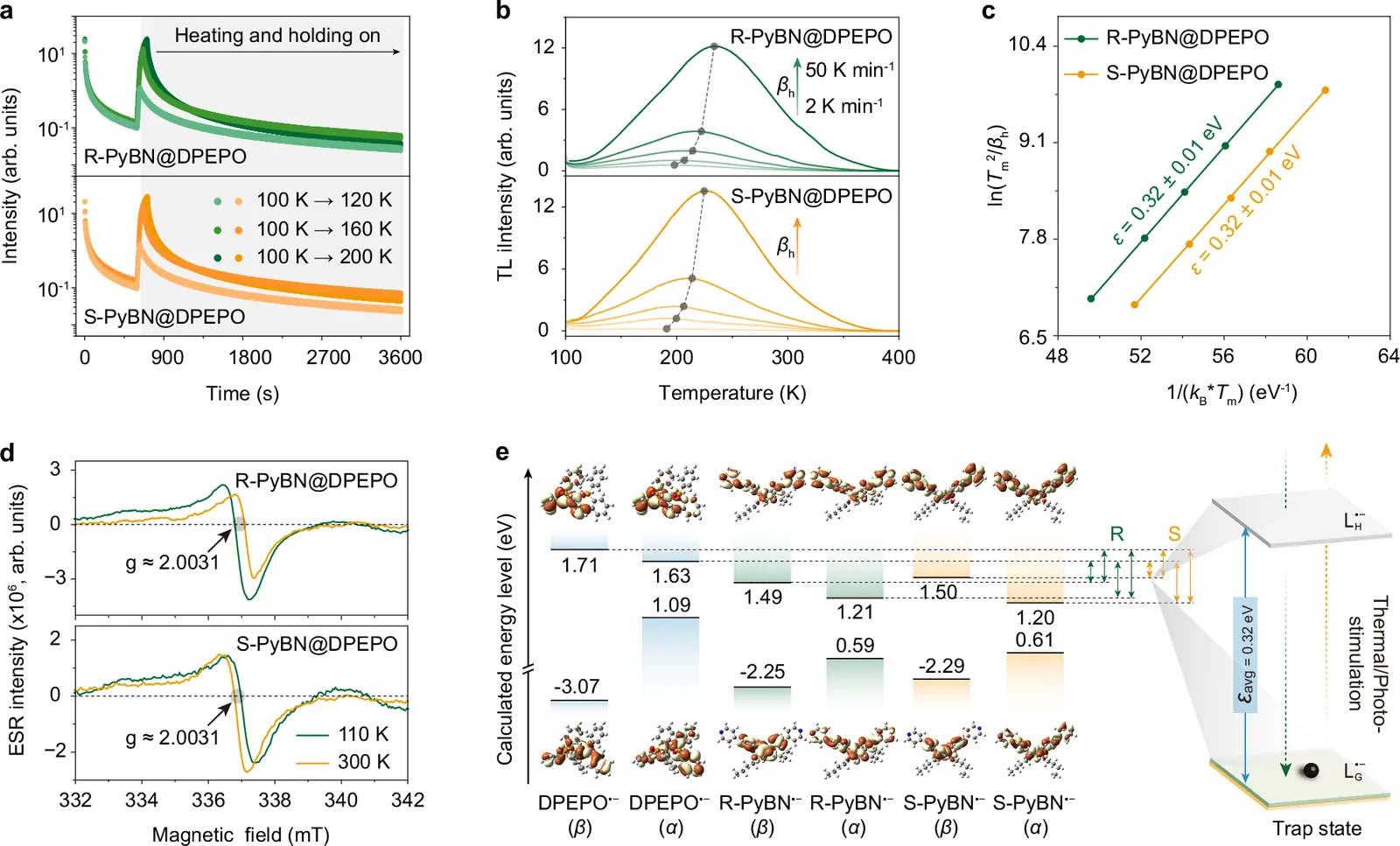
CP-PersL and trap characterization in R/S-PyBN@DPEPO. **a** PersL decay curves under different thermal stimulation conditions. The R/S-PyBN@DPEPO were irradiated by UV light for 60 s and kept at 100 K for 10 min. They were then heated to 120, 160 and 200 K with a heating rate of 50 K min^-1^. **b** TL glow curves with different heating rates of 2, 5, 10, 20 and 50 K min^-1^. The R/S-PyBN@DPEPO were irradiated by UV light for 300 s before the TL measurements. **c** Estimation of trap depth by using the Randall-Wilkins model. **d** ESR spectra recorded after UV irradiation at 110 K (green curves) and 300 K (orange curves). **e** Orbital configurations of radical anions by using the DFT calculation at the B3LYP/def2-SVP level of theory. There are two different densities of electrons with spin *α* and spin *β* in an open-shell system. *ε*_*avg*_ shows the averaged energy difference between the LUMO level of DPEPO^•−^ and that of R/S-PyBN^•−^. The trapping (detrapping) process corresponds to the electron transition from DPEPO^•−^ to R/S-PyBN^•−^ (and vice versa).

After UV irradiation for 1 min and decay at 100 K for 10 min, the R/S-PyBN@DPEPO were heated to higher temperatures and a large increase in emission intensity was observed (Fig. 3a). For example, heating from 100 to 200 K produced an ~180/198-fold enhancement in the R/S-PyBN@DPEPO. Similarly, following UV irradiation and decay at RT for 5 min, we imposed a NIR light beam (980 nm) on the samples and found that the emission intensity was increased by ~30/26 times upon the NIR photo-stimulation (Supplementary Fig. 20). The intensity enhancement was accompanied by a faster decay rate. By using pulsed NIR light, for instance, switching on and off every 30 s, the PersL intensity could be periodically regulated. These results indicate that the PersL in the R/S-PyBN@DPEPO is a temperature- and photo-stimulation-dependent emission, which is likely attributed to the effect of traps.

Systematic TL experiments were conducted to probe the possible traps in the R/S-PyBN@DPEPO. As shown in Fig. 3b, broad TL glow bands covering from 100 to 400 K were observed. *T*_m_ increased as the heating rate (*β*_*h*_) increased. It is noteworthy that R/S-PyBN@DPEPO showed a *T*_m_ value of ~234/224 K under a heating rate of 50 K min^-1^. In contrast, the pure R/S-PyBN exhibited almost no TL peak and DPEPO displayed a peak at ~180 K, indicating that the deep traps were not attributed exclusively to either the host or guest molecule. In R/S-PyBN@DPEPO films, those traps were generated due to the intermolecular interactions between the guest and host molecules (Supplementary Fig. 21). The trap depth (*ε*) was estimated by using the Randall-Wilkins model^63^,

1 $$\frac{{\beta }_{h}\varepsilon }{{{{{\rm{k}}}}}_{{{{\rm{B}}}}}{{{\cdot }}}{T}_{{{{\rm{m}}}}}^{2}}\,=\,s{{{\cdot }}}exp \left(\frac{-\varepsilon }{{{{{\rm{k}}}}}_{{{{\rm{B}}}}}{{{\cdot }}}{T}_{{{{\rm{m}}}}}}\right)$$

where k_B_ is the Boltzmann constant, and *s* is the frequency factor (in s^-1^). According to Fig. 3c, the two samples R/S-PyBN@DPEPO showed a similar trap depth of ~0.32/0.32 eV. The above results suggest that the chiral configuration has a negligible influence on the trap depth of the R/S-PyBN@DPEPO. This might be due to the fact that the molecular orbitals of the guests (and their radical anions/cations) are not affected by chirality^64–66^, which can be further verified from the following molecular orbital simulation.

Absorption spectra of the R/S-PyBN@DPEPO were recorded to reveal the variation of molecule species during UV irradiation. The difference in spectra (Δabs.) indicated that new absorption bands at approximately 300 and 400 nm emerged after UV irradiation (Supplementary Figs. 22, 23). The wavelength regions coincided with those of the R/S-PyBN in their oxidized (~300 nm) and reduced states (~400 nm) obtained from controlled electrochemical reactions (Supplementary Fig. 24). The above results indicated that UV irradiation on the R/S-PyBN@DPEPO might produce radical cations and anions (*i.e*., R/S-PyBN^•+^ and R/S-PyBN^•-^). Meanwhile, the electron spin resonance (ESR) spectra show signals at a g-factor of ~2.0031 upon UV irradiation (Fig. 3d). To exclude potential interference from short-lived triplet electrons, the ESR signals of R/S-PyBN@DPEPO were recorded at various storage time (from 10 min to 28 days) at RT. The ESR signals decayed slowly over time, yet remained detectable after 28 days at RT (Supplementary Fig. 25). The above results confirm the formation of radicals with unpaired electrons upon UV irradiation and their long-term persistence at RT.

We further used density functional theory (DFT) method (at the B3LYP/def2-SVP level of theory) to calculate the frontier molecular orbitals (FMOs) of closed-shell (DPEPO and R/S-PyBN) and open-shell species (DPEPO^•+^, DPEPO^•-^, R/S-PyBN^•+^ and R/S-PyBN^•-^) (Fig. 3e and Supplementary Table 3). The calculated electron cloud distributions and orbital shapes of the R/S molecules were nearly identical, and thus the energy levels of the FMOs were essentially the same in both closed-shell and open-shell systems. Considering two different densities of electrons with spin *α* and spin *β* in the open-shell system, the energy levels of radical cations and anions were averaged (see the Method for more details). The resulting energy differences between the LUMO levels of R/S-PyBN^•−^ and those of DPEPO^•−^ were ~0.32/0.32 eV, which were in agreement with the trap depths (~0.32/0.32 eV) obtained from the TL measurements (Fig. 3e). This suggested that the energy gap of the LUMO between R/S-PyBN^•−^ and DPEPO^•−^ was likely responsible for the energy barrier that electrons need to release from traps and recombine with luminescence centers. Moreover, since the LUMO energy levels of R/S-PyBN^•−^ was basically not affected by the configuration, the chiral configuration has negligible influence on the trap depth of the R/S-PyBN@DPEPO. Notably, despite the similar trap depths in each R/S pair, the PersL duration could vary owing to factors such as different trap densities and unavoidable preparation errors.

Building on the proposed trap-induced CP-PersL model^39,67,68^ and the above results, here we summarize the electron transition processes of CP-PersL in host-guest molecular systems by taking the R/S-PyBN@DPEPO as an example, which consist of four steps (Fig. 1a):

① Excitation and charge separation:

$$({{{\rm{R/S}}}}-{{{\rm{PyBN}}}})+{{{\rm{DPEPO}}}}{\to }^{{{\rm{hv}}}}{({{{\rm{R/S}}}}-{{{\rm{PyBN}}}})}^{*}+{{{\rm{DPEPO}}}}{\to }^{{{\rm{hv}}}}({{{\rm{R/S}}}}-{{{\rm{PyBN}}}})^{{{{\rm{\bullet }}}}+}+{{{\rm{DPEPO}}}}^{{{{\rm{\bullet }}}}-*}$$

② Charge trapping:

$${{{\rm{DPEPO}}}}^{{{{\rm{\bullet }}}}-*}+({{{\rm{R/S}}}}-{{{\rm{PyBN}}}}){ \longrightarrow }^{{{\rm{trapping}}}}{{{\rm{DPEPO}}}}+({{{\rm{R/S}}}}-{{{\rm{PyBN}}}})^{{{{\rm{\bullet }}}}-*}$$

③ Charge detrapping:

$${{{\rm{DPEPO}}}}+{({{{\rm{R/S}}}}-{{{\rm{PyBN}}}})}^{{{{\bullet }}}- * }{ \longrightarrow }^{{{\rm{detrapping}}}}{{{\rm{DPEPO}}}}^{{{{\bullet }}}- * }+({{{\rm{R/S}}}}-{{{\rm{PyBN}}}})$$

④ Charge recombination and photon emission:

$${{{\rm{DPEPO}}}}^{{{{\bullet }}}- * }+({{{\rm{R/S}}}}-{{{\rm{PyBN}}}})^{{{{\bullet }}}+}\to {{{\rm{DPEPO}}}}+{({{{\rm{R/S}}}}-{{{\rm{PyBN}}}})}^{ * }\to {{{\rm{DPEPO}}}}+{({{{\rm{R/S}}}}-{{{\rm{PyBN}}}})}^{ * }+{{{\rm{hv}}}}$$

where the asterisk indicates that the species contains an electron at the LUMO level, and h*v* is the irradiation light or photon emission. The mechanism suggests that under UV irradiation, electrons in the chiral guest R/S-PyBN are excited. Some excited electrons rapidly return to the guest molecule to undergo radiative emission. Others are transferred to the DPEPO, producing two metastable radical species (R/S-PyBN)^•+^ and DPEPO^•−*^. Due to the superior electron transport ability, electrons are mobile within host molecules, and then captured by another guest that has not lost electrons to generate (R/S-PyBN)^•−*^. The energy gap of the LUMO levels between (R/S-PyBN)^•−*^ and DPEPO^•−*^ forms an energy barrier *ε*. Finally, the electron is released from the traps under thermal stimulation or photo-stimulation and recombines with the original luminescent center (R/S-PyBN)^•+^. It should be noted that detrapped electrons from higher-lying energy levels repopulate both the triplet and singlet states of R/S-PyBN with a large value of Δ*E*_*ST*_ ( ~ 0.52/0.52 eV), thereby achieving dual-peak emission lasting for hours (Fig. 1b).

### Warm-white CP-PersL in other host-guest molecular systems

We further synthesized other chiral guest molecules with similar electronic configurations, such as R/S-4,4’-((2,2’-dibutoxy-[1,1’-binaphthalene]−6,6’-diyl)bis(ethyne-2,1-diyl))dipyridine (R/S-APyBN), by introducing an alkyne bond to extend the distance between the pyridine and naphthalene rings and R/S-4,4’-(2,2’-bis(methoxymethoxy)-[1,1’-binaphthalene]−6,6’-diyl)dipyridine (R/S-PyBNMO), by modifying the structure of R_1_ group. They were combined with various electron transport hosts (DPEPO, BCPO, and SPPO13) to synthesize a series of host-guest systems.

**Fig. 4:**
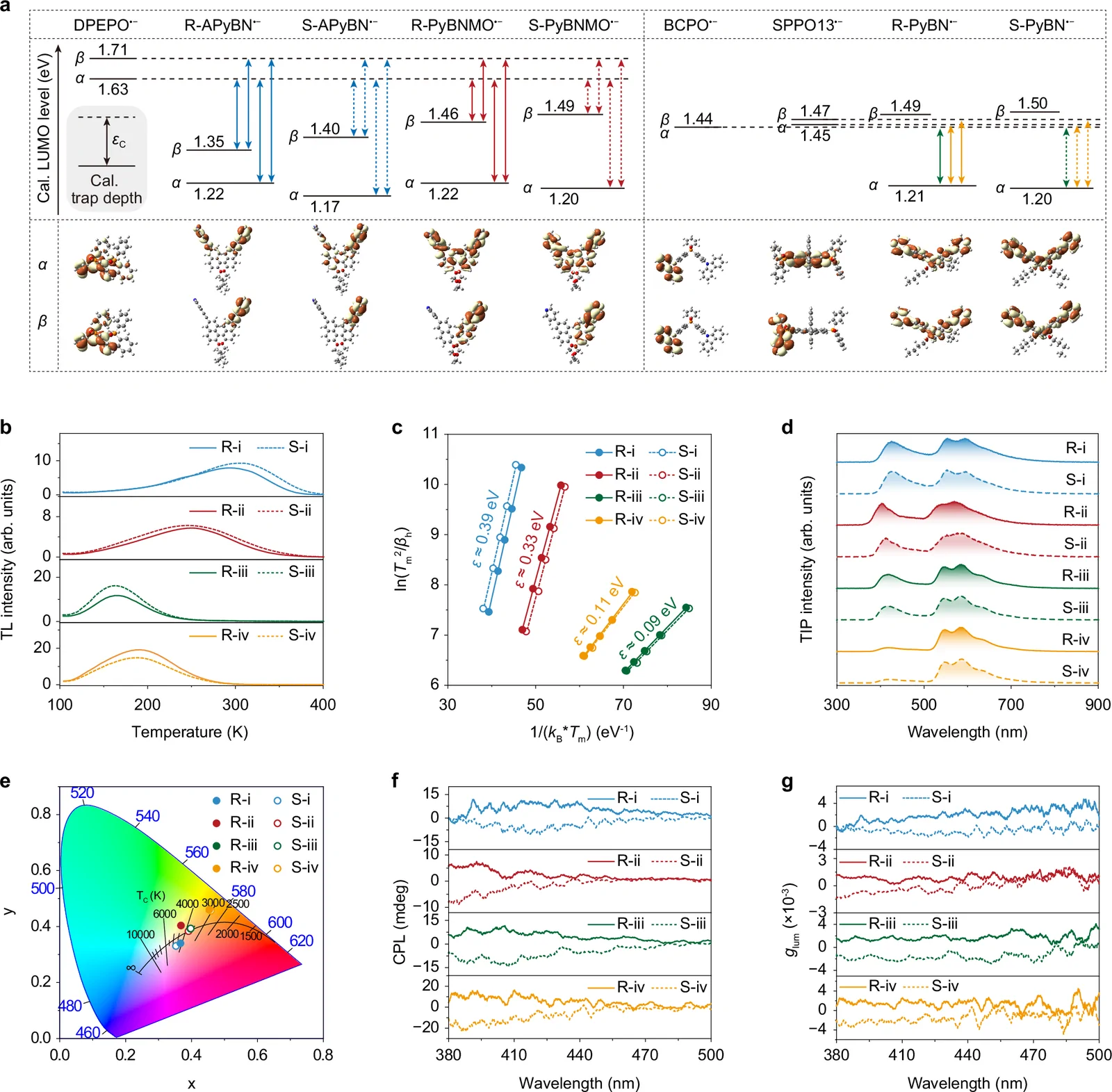
Warm-white CP-PersL in various host-guest molecular systems. **a** LUMO states of radical anions R/S-APyBN and R/S-PyBNMO compared with those of DPEPO, and LUMO states of R/S-PyBN compared with those of BCPO and SPPO13. **b** TL glow curves of R/S-APyBN@DPEPO (R-i/S-i), R/S-PyBNMO@DPEPO (R-ii/S-ii), R/S-PyBN@BCPO (R-iii/S-iii), and R/S-PyBN@SPPO13 (R-iv/S-iv). The samples were first excited by UV light for 300 s at 100 K. The heating rate was kept at 50 K min^-1^. **c** Estimation of trap depths with the Randall-Wilkins model. **d** PersL spectra. **e** CIE chromaticity of PersL. **f** CPL spectra. **g** *g*_lum_ spectra under excitation of 340 nm.

Here, we take R/S-APyBN@DPEPO (i), R/S-PyBNMO@DPEPO (ii), R/S-PyBN@BCPO (iii), and R/S-PyBN@SPPO13 (iv) to show their photophysical properties (Supplementary Figs. 26, 27). The orbital configurations of these host and guest molecules were predicted with DFT calculation and the detailed information is summarized in Fig. 4a and Supplementary Table 3. Under UV irradiation, all of these composite films exhibited blue emissions, with corresponding QY values of 19.0/20.3% for R/S-APyBN@DPEPO, 33.8/42.6% for R/S-PyBNMO@DPEPO, 31.9/35.2% for R/S-PyBN@BCPO, and 21.1/20.1% for R/S-PyBN@SPPO13 (Supplementary Table 4). After UV excitation, they all showed short-lived yellow phosphorescence and hour-long PersL. The PersL decay curves all followed the Debye-Edwards power-law relationship (Supplementary Fig. 28). According to the TL measurements, these host-guest systems exhibited different peak temperatures in the glow curves, yet the values for each R/S pair were similar (Fig. 4b and Supplementary Table 4). The estimated trap depths were ~0.39/0.39 eV for R/S-APyBN@DPEPO, ~0.33/0.33 eV for R/S-PyBNMO@DPEPO, ~0.09/0.09 eV for R/S-PyBN@BCPO and ~0.11/0.11 eV for R/S-PyBN@SPPO13 (Fig. 4c and Supplementary Figs. 29–32).

The estimated trap depths aligned well with the average energy gaps (arithmetic mean values) obtained from the energy differences between the LUMO levels of guest^•−^ and those of host^•−^ (Supplementary Table 5). The above results indicate that the trap depths could be tailored by selecting different combinations of guest and host materials. All the samples exhibited warm-white PersL and TL emissions with dual-band characteristics jointly contributed by singlet-singlet and triplet-singlet transitions (Supplementary Figs. 33, 34), giving CIE coordinates of (0.37, 0.34)/(0.35, 0.33), (0.37, 0.40)/(0.39, 0.38), (0.40, 0.39)/(0.40, 0.39), and (0.45, 0.46)/(0.46, 0.46), respectively (Fig. 4d, e). Furthermore, we confirmed that all these host-guest systems maintained the chiral characteristics of the ground state and the excited state (Fig. 4f and Supplementary Fig. 35). The recorded values of *g*_lum_ were 1.5 × 10^-3^/-1.1 × 10^-3^ for R/S-APyBN@DPEPO, 9.6 × 10^-4^/-7.9 × 10^-4^ for R/S-PyBNMO@DPEPO, 1.2 × 10^−3^/-1.4 × 10^-3^ for R/S-PyBN@BCPO, and 1.6 × 10^-3^/−2.3 × 10^−3^ for R/S-PyBN@SPPO13, respectively (Fig. 4f, g). These results demonstrated that trap-induced white CP-PersL materials could be realized in organic materials through precise design of host and guest molecules, with detrapped electrons undergoing repopulation of the singlet and triplet excited states.

### Application demonstration

**Fig. 5:**
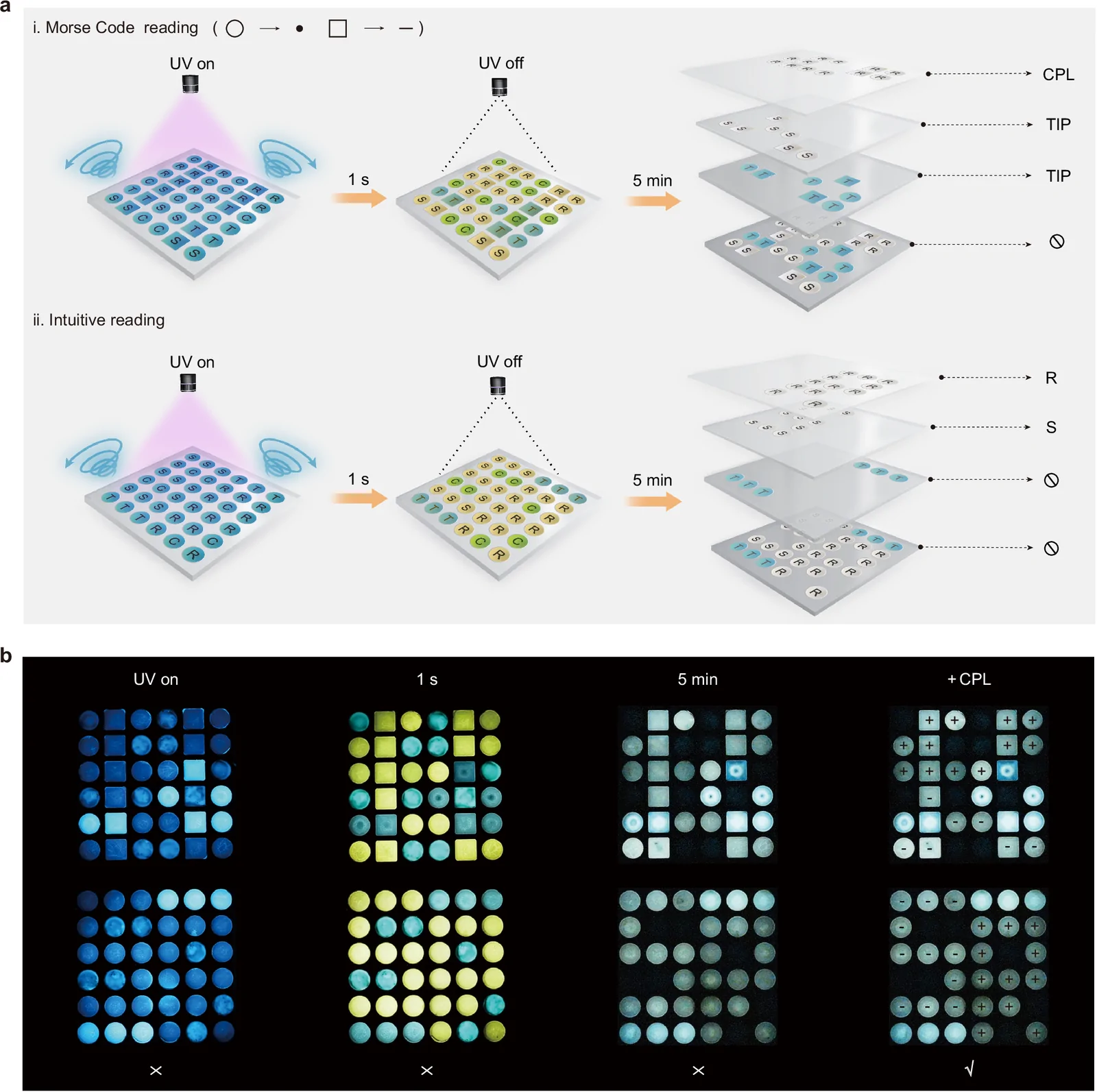
Applications of warm-white CP-PersL materials for optical information encryption. **a** Schematic illustration of Morse code reading (i) and intuitive reading (ii) of optical information under 365 nm excitation (UV on), and after ceasing the UV excitation. **b** The corresponding photographs. The excitation time was 60 s. The size of these 6 × 6 matrix brass panels was 59 × 59 × 3 mm^3^. The side length of each square groove and the diameter of the circular groove were both 7.5 mm while the depth was 1.5 mm. The wrong and correct information were indicated by cross-shaped and check-shaped markers, respectively. Positive- and negative-polarity labels represented right-handed and left-handed circular polarized signals, respectively. Camera exposure settings: shutter 1/2000 s, ISO 100 for UV on; shutter 1/4000 s, ISO 20000 for 1 s; shutter 10 s, ISO 40000 (i) and shutter 5 s, ISO 64000 (ii) for 5 min.

Considering the unique properties of warm-white PersL combined with CPL in the synthesized materials, we further conducted a proof-of-concept experiment on optical information encryption (Fig. 5). For simplification, R-PyBN@DPEPO, S-PyBN@DPEPO, a previously reported RTP carbon dots sample^69^, and another long-lived thermally activated delayed fluorescence (TADF) compound 8 wt % 2,7-di(9H-carbazol-9-yl)-1,8-naphthyridine@DPEPO (DCz-ND@DPEPO) are denoted as R, S, C, and T, respectively. Their chemical structures and photophysical properties are given in Supplementary Figs. 36, 37. In Morse code demonstration, a 6 × 6 matrix containing circular and square grooves was designed for encoding, with the circular and the square grooves representing dots and dashes, respectively (Fig. 5a). The four materials were loaded into different grooves. We set warm-white-light emission with a delay time as the decoded information. Under UV irradiation, all materials exhibited similar blue luminescence. After ceasing the irradiation, the four compounds emitted light with different colors. With a delay time of 5 min after irradiation, green emission from C completely disappeared. With the help of CPL analysis and Morse code table, the hidden information CPL (right-handed) and TIP (left-handed) were finally interpreted. In addition, we used the same method to fill these materials into another 6 × 6 matrix with pure circular grooves for intuitive reading (Fig. 5b). Following similar rules, the information R (right-handed) and S (left-handed) were directly distinguished.

## Discussion

In summary, we proposed a simple way to realize white CP-PersL emissions by introducing traps to chiral guest molecules, which involved dual emissions from the singlet and triplet states. The single-component doped dual-band white CP-PersL was obtained by repopulation of detrapped electrons into singlet and triplet states during the charge recombination process. It was worth noting that the two enantiomers, namely R-PyBN and S-PyBN, were synthesized and incorporated into DPEPO to prepare composite films. The films exhibited warm-white PersL lasting more than 4 h, which exceeded most of the previously reported chiral small-molecule systems (Supplementary Table 6). Meanwhile, these films exhibited asymmetric factors *g*_lum_ of 9.0 × 10^-4^/-8.2 × 10^-4^. Importantly, the design route that simultaneously achieved white CP-PersL could be applied to a variety of material systems.

The dual emission of PersL from triplet–singlet and singlet–singlet transitions in this work expands the material family of organic CP-PersL and provides a theoretical framework for developing organic emitters with lifetimes including millisecond/second-level phosphorescence and hour-level PersL. The differentiation and spectral tuning of two distinct long-lived luminescence types coexisting within a material system hold considerable promise for organic luminescent materials, although the performance demonstrated herein still warrants improvement. Future molecular engineering of chiral guest molecules with superior CPL performance and lower singlet energy levels may further enhance the *g*_*lum*_ values and achieve a more ideal white-light emission, potentially opening new avenues for optical information storage and anti-counterfeiting.

## Methods

### Materials and characterizations

R/S-PyBN and R/S-PyBNMO were synthesised by palladium-catalysed Suzuki cross-coupling reaction. R/S-APyBN were synthesized by Sonogashira cross-coupling reaction. The detailed preparation procedures are given in Supplementary Information. The chemical structures of the guests were characterized by nuclear magnetic resonance (NMR) spectroscopy (JNM-ECZ400S/L, JEOL) and high-resolution mass spectra (HRMS, UHPLC-QTOF, Agilent). The purity of the used guests and hosts were verified by high-performance liquid chromatography (HPLC, LCMS2020, Shimadzu). The NMR, HRMS and HPLC spectra are provided in Supplementary Fig. 38–62. The results confirmed the high chemical purity of the samples and suitability for this study.

### Fabrication of composite films

A mixture of guest (R/S-PyBN, R/S-APyBN, or R/S-PyBNMO; 1 mg) and host (99 mg) molecules were heated up to 320 °C on a quartz substrate inside an anaerobic glovebox. After the mixture is melted, the molten liquid was thoroughly stirred and then rapidly cooled to RT. Finally, the mixture was encapsulated in a glass cover with a size of 2 × 2 cm^2^ using a high-temperature UV-curable adhesive under a nitrogen atmosphere. In order to reduce the experimental error, all the host-guest composite films were prepared by using the same method.

### Fabrication of samples for application demonstration

1 wt% R-PyBN@DPEPO, S-PyBN@DPEPO and 8 wt% DCz-ND@DPEPO composite films were prepared in air using the same melt-casting method as mentioned above. They were crushed into powder and filled into the corresponding grooves for the second melt-quenching. Finally, it was put into an anaerobic glovebox box for the third melt-quenching. The CDs powder and the high-temperature UV-curable adhesive were filled into the corresponding grooves at a mass ratio of 1:1. These materials were encapsulated in a 6 × 6 matrix brass panel with a size of 59 × 59 × 3 mm^3^ in a nitrogen atmosphere using a high-temperature UV-curable adhesive.

### Measurements of photophysical properties

Thermogravimetric analysis (TGA) was conducted on a thermal analyzer (TG 209 F3 Tarsus, NETZSCH) at a heating rate of 10 °C/min under a nitrogen atmosphere. The luminescence quantum yield was measured on the absolute quantum yield spectrometer (C11347, Hamamatsu Photonics). Absorption spectra were measured with a UV/visible/NIR spectrophotometer (UV-3600Plus, Shimadzu). Emission spectra and lifetime decay curves were collected in a fluorescence spectrometer (FLS980, Edinburgh and QE-Pro, Ocean Optics). ESR spectra were obtained on the X-band electron paramagnetic resonance spectrometer (EMXplus-9.5/12, Bruker). The cyclic voltammetry curves and electrochemical deposition operation were carried out by an electrochemical workstation (CHI660E, CH Instruments). The experimental HOMO and LUMO energy were estimated with cyclic voltammetry with ferrocene/ferrocenium as standard correction (platinum as working electrode, platinum wire as auxiliary electrode, and silver wire as reference electrode). The radical cation and anion of guest /host were obtained by electrical oxidation or reduction reaction when the guest /host were dissolved in acetonitrile containing 0.1 M TBAPF_6_ under a nitrogen atmosphere. CPL spectra with dissymmetry factor (*g*_lum_) were recorded by a JASCO CPL-300 commercial instrument at a scanning speed of 200 nm min^-1^. Specifically, *g*_lum_ quantifies the degree of circular polarization in a CPL system, and is defined as: *g*_lum_ = 2(*I*_L_ − *I*_R_) / (*I*_L_ + *I*_R_); where *I*_L_ and *I*_R_ are the intensities of left- and right-handed circularly polarized light, respectively. CD spectra were measured by JASCO J-1700. Photographs of the samples were taken with a digital camera (α7SIII, SONY).

### PersL measurements

TL, PersL spectra and decay curves were obtained by using a custom-made measurement system. In a typical TL measurement, the samples placed in a cryostat controlled by a cooling heating stage (THMS600E, Linkam Scientific Instruments) were first cooled to 100 K. Subsequently, the samples were charged by using a 365 nm UV flashlight with a power of 10 W. 20 s after creasing the excitation source, the sample were heated to 400 K at a certain heating rate. During the heating process, the emission intensity (including TL emission and PersL decay profile) was simultaneously monitored by a filter-attached PMT (R928P, Hamamatsu Photonics), a luminance meter (LM−5, Evenfine), a multimeter (2400, Keithley) and a high-voltage power supply (HVC1800, Zolix). At the same time, the transient luminescence emission spectrum was obtained simultaneously by a multi-channel spectrometer (QE-Pro, Ocean Optics). The above-mentioned measurement systems were driven by LabVIEW-based computer programs. An NIR laser at 980 nm (~200 mW cm^-2^) was projected onto the surface of the sample as a photo-stimulation source.

### Theoretical calculations

The DFT calculations were performed on simplified models of isolated molecules (Supplementary Data 1). All molecules were optimized at the B3LYP/def2-SVP level for the equilibrium geometries of ground state (S_0_) using the Gaussian 09 program (Revision B.01). Subsequently, the FMOs based on the S_0_ geometry were depicted for the closed-shell and open-shell systems. For the open-shell radical anions, the discrete *α* and *β* spin molecular orbitals resulted in four different LUMO energy differences between the two molecular systems. To compare with the single trap-depth value estimated from TL measurements for the macroscopic sample (composed of a large number of molecules), we adopted a simplified arithmetic averaging approach to obtain a single energy-level difference. However, it should be noted that such simplification reflects the overall trend and does not fully capture the actual solid-state host-guest environment. Furthermore, the DFT-derived energy level values could also be limited by the choice of functionals and basis sets. The DFT calculations should be regarded as a qualitative evaluation method of the trap depth rather than quantitative support.

## Supplementary information

Supplementary Information

Description of Additional Supplementary Information

Supplementary Data 1

Transparent Peer Review file

## Source data

Source Data

## Data Availability

Source data are provided with this paper. The source data that support the findings of this study have been deposited in the figshare repository (https://doi.org/10.6084/m9.figshare.32791377). Any requests on data and experiments can be also available from the corresponding authors. Received: ((will be filled in by the editorial staff)) Revised: ((will be filled in by the editorial staff)) Published online: ((will be filled in by the editorial staff)). Source data are provided with this paper.
