# Supplementary material for: Trap-induced white circularly polarized persistent luminescence from a host-guest molecular system
Source: Nat Commun. 2026 Aug 7;17:9517. doi: 10.1038/s41467-026-76289-7 (PMC13547245; doi:10.1038/s41467-026-76289-7)
Supplement: Supplementary file 1 [file 41467_2026_76289_MOESM1_ESM.pdf]

Supplementary Information

Trap-Induced White Circularly Polarized Persistent Luminescence from A Host-Guest Molecular System

Yajing Wang[1,#], Cunjian Lin[2,#], Yang Li[3], Chenhan Zhan[1], Rujun Yang[1], Zishuang Wu[4], Shunjie Shu[1], Wenpeng Ye[5], Wenting Deng[3], Jumpei Ueda[2,*], Yixi Zhuang[1,6*], Rong-Jun Xie[1,7*]

[1]College of Materials, and Fujian Key Laboratory of Surface and Interface Engineering for High Performance Materials, Xiamen University, Xiamen 361005, China.

[2]Graduate School of Advanced Science and Technology, Japan Advanced Institute of Science and Technology, Nomi 923-1292, Japan.

[3]College of Chemistry and Chemical Engineering, Gannan Normal University, Ganzhou 341000, China.

[4]Institute of Flexible Electronics (IFE, Future Technologies), Xiamen University, Xiamen 361005, China.

[5]Key Laboratory of Flexible Electronics & Institute of Advanced Materials, Nanjing Tech University, Nanjing 211816, China.

[6]Shenzhen Research Institute of Xiamen University, Shenzhen 518000, China.

[7]State Key Laboratory of Physical Chemistry of Solid Surfaces, Xiamen University, Xiamen 361005, China.

[#]These authors contributed equally: Yajing Wang, Cunjian Lin.

*Correspondences: ueda-j@jaist.ac.jp; zhuangyixi@xmu.edu.cn; rjxie@xmu.edu.cn

Contents

I. Synthesis of Molecules 3

II. Photophysical Properties of R/S-PyBN and DPEPO 7

III. Photophysical Properties of R/S-PyBN@DPEPO Films 10

IV. Trap Characteristics of R/S-PyBN@DPEPO Films 13

V. Theoretical Calculations of Frontier Molecular Orbitals (FMOs) 16

VI. Other Guest-Host Systems Showing White CP-PersL 17

VII. Demonstration of Applications 24

VIII. Background Information 25

IX. Nuclear Magnetic Resonance (NMR) Spectra, High-Resolution Mass Spectra (HRMS) and High-Performance Liquid Chromatography (HPLC) 31

X. Supplementary References 41

I. Synthesis of Molecules

General. Unless otherwise noted, all reagents used in the experiments were purchased from commercial sources without further purification. DPEPO, BCPO, and SPPO13 were obtained from Xi'an Polymer Light Technology Corp. R/S-PyBNMO, DCz-ND, and carbon dots (CDs) were designed and synthesized according to Refs. 1–3.

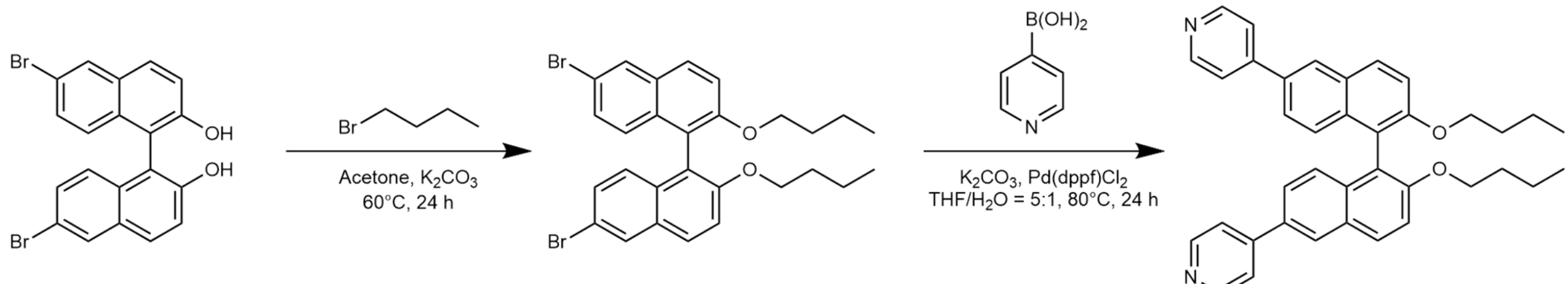

R/S-1 R/S-2 R/S-PyBN

Supplementary Figure 1 | Synthetic route of R/S-4,4'-(2,2'-dibutoxy-[1,1'-binaphthalene]-6,6'-diyl)dipyridine (R/S-PyBN) molecules.

Synthesis of R/S-PyBN. At room temperature (RT), 150 mL of acetone, 3.0 g of compound **R-1** (6.8 mmol, 1 eq), 4.65 g of 4-bromobutane (34 mmol, 5 eq), and 18.0 g of potassium carbonate (136 mmol, 20 eq) were sequentially added to a 250 mL round bottom flask and refluxed at 65 °C. The reaction was stopped after 24 hours. The reaction solution was cooled to RT, filtered, and the filtrate was concentrated. The obtained product **R-2** is sufficiently pure and does not require further processing. At RT, 100 mL of THF, 20 mL of H_2O, 1 g of compound **R-2** (1.8 mmol, 1 eq), 1.77 g of pyridine boronic acid (14.4 mmol, 8 eq), 1.5 g of potassium carbonate (10.8 mmol, 6 eq), and 130 mg of Pd(dppf)Cl_2 (0.18 mmol, 0.1 eq) were sequentially added to a 250 mL round bottom flask. After stirring for 5 minutes, the mixture was refluxed at 80 °C. The reaction was stopped after 48 hours. The reaction solution was cooled to RT and the solution was concentrated. It was then washed with 50 mL of H_2O and extracted three times with dichloromethane (DCM). The organic layer was then washed with pure water and saturated sodium chloride (50 mL), dried over anhydrous sodium sulfate, filtered, and the filtrate was concentrated. The crude product was purified by column chromatography on silica gel (PE: EA: NEt_3 =5:3:1) to obtain a yellow solid **R-PyBN** (0.50 g, yield: 50.2%); When the raw material is **S-1**, a light yellow solid **S-PyBN** (0.51 g, yield: 50.3%) is obtained.

For **R-PyBN**, ^{1}H NMR (400 MHz, $CDCl_3$) δ 8.68 – 8.62 (m, 2H), 8.15 (d, J = 1.9 Hz, 1H), 8.07 – 8.00 (m, 1H), 7.62 – 7.55 (m, 2H), 7.53 – 7.44 (m, 2H), 7.26 (d, J = 9.1 Hz, 1H), 4.06 – 3.92 (m, 2H), 1.50 – 1.35 (m, 2H), 1.02 (dqd, J = 14.2, 7.5, 1.5 Hz, 2H), 0.65 (t, J = 7.4 Hz, 3H). ^{13}C NMR (100 MHz, $CDCl_3$) δ 155.47, 150.35, 148.45, 134.39, 132.88, 130.02, 129.19, 126.53 (d, *J* = 11.0 Hz), 124.84, 121.64, 120.05, 116.21, 69.26, 31.39, 18.84, 13.66. HRMS (ESI): m/z calcd for $C_{38}H_{36}N_2O_2$ $[M+H]^+$: 553.2855, found: 553.2882.

For **S-PyBN**, ^{1}H NMR (400 MHz, $CDCl_3$) δ 8.68 – 8.62 (m, 4H), 8.15 (d, J = 1.9 Hz, 2H), 8.07 – 8.00 (m, 2H), 7.62 – 7.56 (m, 4H), 7.55 – 7.44 (m, 4H), 7.27 (d, J = 0.8 Hz, 2H), 4.06 – 3.92 (m, 4H), 1.48 – 1.37 (m, 4H), 1.02 (dqd, J = 14.4, 7.4, 1.5 Hz, 4H), 0.65 (t, J = 7.4 Hz, 6H). ^{13}C NMR (100 MHz, $CDCl_3$) δ 155.48, 150.34, 148.46, 134.39, 132.88, 130.02, 129.19,

126.53 (d, J = 11.5 Hz), 124.84, 121.65, 120.06, 116.21, 69.26, 31.38, 18.84, 13.66. HRMS (ESI): m/z calcd for $C_{38}H_{36}N_2O_2$ $[M+H]^+$: 553.2855, found: 553.2884.

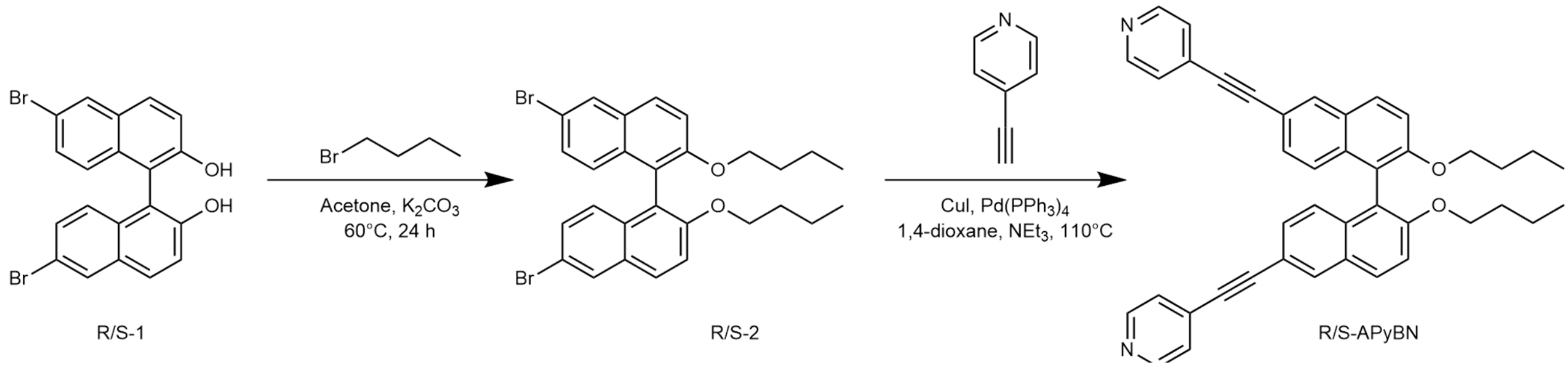

Supplementary Figure 2 | Synthetic route of R/S-4,4'-((2,2'-dibutoxy-[1,1'-binaphthalene]-6,6'-diyl)bis(ethyne-2,1-diyl))dipyridine (R/S-APyBN) molecules.

Synthesis of R/S-APyBN. Synthesize **R/S-2** according to the steps 1. At RT, 500 mg of compound **R-2** (0.9 mmol, 1 eq), 300 mg of alkynyl pyridine (2 mmol, 4 eq), 280 mg of $Pd(Ph_3P)_4$ (0.25 mmol, 0.35 eq), and 55 mg of CuI (0.3 mmol, 0.4 eq) were sequentially added to a 250 mL double necked flask with nitrogen protection. Then, 40 mL of triethylamine and 20 mL of 1,4-dioxane were added, followed by nitrogen protection. The reaction was monitored at 110 °C using a dot plate (PE: EA: NEt_3=1:1:0.1), and stopped after 24 hours of reaction. After cooling the reaction solution to RT, add 125 mL of saturated ammonium chloride solution, extract 3-5 times with DCM, then take the organic layer and dry it with anhydrous sodium sulfate. Filter and concentrate the filtrate. The crude product was further purified by column chromatography on silica gel using PE: EA: NEt_3=3:1:0.1 as eluent to obtain yellow solid **R-APyBN** (0.32 g, yield: 58.9%); When the raw material is **S-2**, a yellow solid **S-APyBN** (0.33 g, yield: 60.8%) is obtained.

For **R-APyBN**, ^{1}H NMR (400 MHz, $CDCl_3$) δ 8.63 – 8.47 (m, 4H), 8.07 (d, *J* = 1.7 Hz, 2H), 7.93 – 7.82 (m, 2H), 7.45 – 7.30 (m, 6H), 7.27 (dd, *J* = 8.7, 1.7 Hz, 2H), 7.10 – 7.04 (m, 2H), 4.02 – 3.87 (m, 4H), 1.42 – 1.29 (m, 4H), 1.05 – 0.91 (m, 4H), 0.62 (t, *J* = 7.4 Hz, 6H). ^{13}C NMR (100 MHz, $CDCl_3$) δ 155.77, 149.78, 134.10, 132.45, 131.79, 129.52, 128.56, 128.50, 125.64, 125.56, 119.96, 116.74, 116.02, 94.98, 86.52, 77.38, 77.27, 77.06, 76.75, 69.12, 31.29, 18.78, 13.57. HRMS (ESI): m/z calcd for $C_{42}H_{36}N_2O_2$ $[M+H]^+$: 601.2855, found: 601.2884.

For **S-APyBN**, ^{1}H NMR (400 MHz, $CDCl_3$) δ 8.61 – 8.55 (m, 4H), 8.11 (d, *J* = 1.6 Hz, 2H), 7.93 (d, *J* = 9.0 Hz, 2H), 7.47 – 7.35 (m, 6H), 7.30 (dd, *J* = 8.8, 1.7 Hz, 2H), 7.11 (d, *J* = 8.8 Hz, 2H), 4.04 – 3.90 (m, 4H), 1.45 – 1.37 (m, 4H), 1.08 – 0.93 (m, 4H), 0.65 (t, *J* = 7.4 Hz, 6H). ^{13}C NMR (100 MHz, $CDCl_3$) δ 155.79, 149.86, 134.12, 132.50, 131.81, 129.57, 128.56 (d, *J* = 3.1 Hz), 125.64 (d, *J* = 7.6 Hz), 119.95, 116.75, 116.03, 94.99, 86.56, 69.13, 31.31, 18.81, 13.63. HRMS (ESI): m/z calcd for $C_{42}H_{36}N_2O_2$ $[M+H]^+$: 601.2855, found: 601.2880.

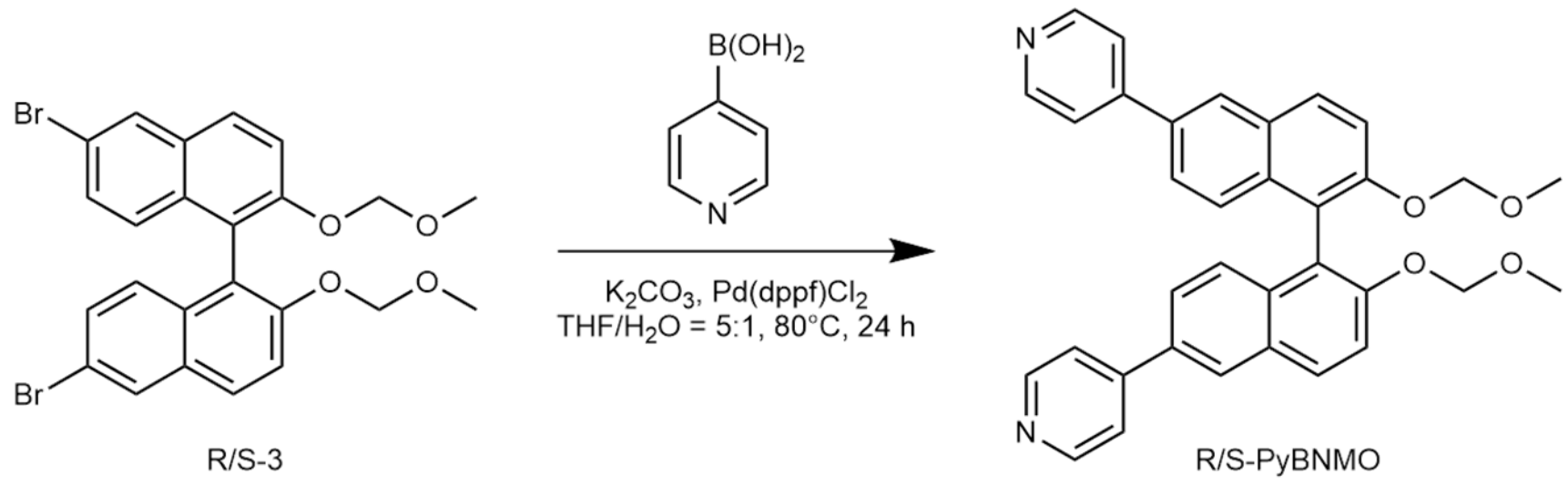

Supplementary Figure 3 | Synthetic route of R/S-4,4'-(2,2'-bis(methoxymethoxy)-[1,1'-binaphthalene]-6,6'-diyl)dipyridine (R/S-PyBNMO) molecules.

Synthesis of R/S-PyBNMO. At RT, 100 mL of THF, 20 mL of H_2O, 1 g of compound **R-3** (1.8 mmol, 1 eq), 1.77 g of pyridine boronic acid (14.4 mmol, 8 eq), 1.5 g of potassium carbonate (10.8 mmol, 6 eq), and 130 mg of Pd(dppf)Cl_2 (0.18 mmol, 0.1 eq) were sequentially added to a 250 mL round bottom flask. After stirring for 5 minutes, the mixture was refluxed at 80 °C. The reaction was stopped after 24 hours. The reaction solution was cooled to RT, filtered, and the filtrate was concentrated. It was then washed with 50 mL of H_2O and extracted three times with DCM. The organic layer was then washed with pure water and saturated sodium chloride (50 mL), dried over anhydrous sodium sulfate, filtered, and the filtrate was concentrated and passed through a silica gel column (PE: EA: NEt_3 =5:3:1) to obtain a yellow solid **R-PyBNMO** (0.55 g, yield: 50.2%); When the raw material is **S-3**, a light yellow solid **S-PyBNMO** (0.57 g, yield: 50.2%) is obtained.

For R-PyBNMO, ^{1}H NMR (400 MHz, $CDCl_3$) δ 8.69 – 8.63 (m, 4H), 8.18 (d, *J* = 1.9 Hz, 2H), 8.06 (d, *J* = 9.0 Hz, 2H), 7.66 (d, *J* = 9.1 Hz, 2H), 7.61 – 7.55 (m, 4H), 7.51 (dd, *J* = 8.8, 2.0 Hz, 2H), 7.27 (d, *J* = 8.8 Hz, 2H), 5.13 (d, *J* = 6.8 Hz, 2H), 5.04 (d, *J* = 6.9 Hz, 2H), 3.19 (s, 6H). ^{13}C NMR (100 MHz, $CDCl_3$) δ 153.62, 150.41, 148.19, 134.17, 133.67, 130.31, 129.94, 126.57 (d, *J* = 10.0 Hz), 125.12, 121.68, 120.80, 117.94, 95.15, 56.05. HRMS (ESI): m/z calcd for $C_{34}H_{28}N_2O_4$ $[M+H]^+$: 529.2127, found: 529.2135.

For **S-PyBNMO**, ^{1}H NMR (400 MHz, $CDCl_3$) δ 8.66 (d, *J* = 5.3 Hz, 4H), 8.18 (d, *J* = 1.9 Hz, 2H), 8.06 (d, *J* = 9.0 Hz, 2H), 7.66 (d, *J* = 9.0 Hz, 2H), 7.61-7.56 (m, 4H), 7.51 (dd, *J* = 8.8, 1.9 Hz, 2H), 7.27 (d, *J* = 8.8 Hz, 2H), 5.13 (d, *J* = 6.8 Hz, 2H), 5.04 (d, *J* = 6.9 Hz, 2H), 3.19 (s, 6H). ^{13}C NMR (100 MHz, $CDCl_3$) δ 150.39, 126.58 (d, *J* = 10.6 Hz), 121.70, 117.95, 77.31, 56.05. HRMS (ESI): m/z calcd for $C_{34}H_{28}N_2O_4$ $[M+H]^+$: 529.2127, found: 529.2137.

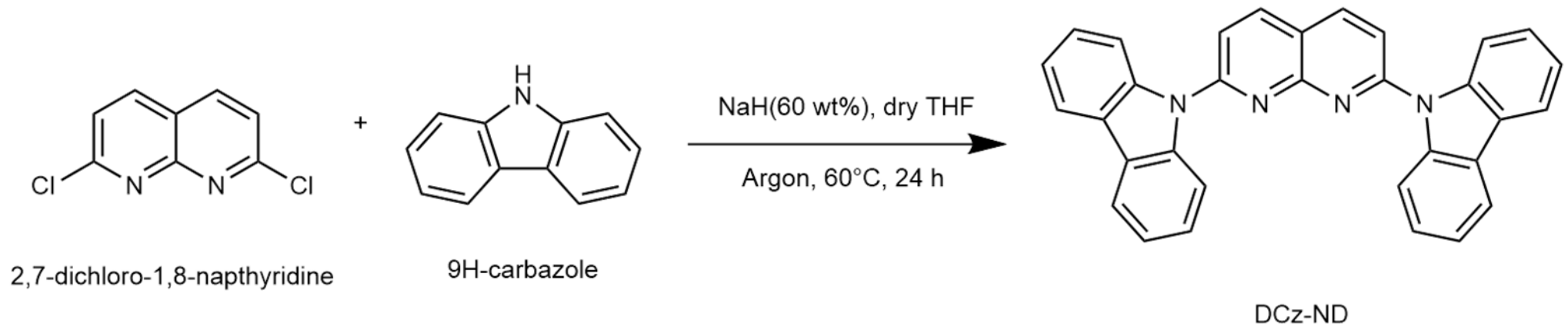

Supplementary Figure 4 | Synthetic route of 2,7-di(9H-carbazol-9-yl)-1,8-naphthyridine (DCz-ND) molecules.

Synthesis of DCz-ND. A mixture of 9H-carbazole (502 mg, 3 mmol) and sodium hydride (60 wt%, 240 mg, 6 mmol) in dry tetrahydrofuran was stirred under an argon atmosphere at RT for 30 minutes. To this mixture, 2,7-dichloro-1,8-

naphthyridine (200 mg, 1 mmol) was added in one portion. The reaction was heated to 60°C and stirred for 24 h. After cooling, the mixture was poured into water and extracted with DCM (3 × 100 mL). The combined organic extracts were concentrated under reduced pressure, and the resulting residue was purified by column chromatography on silica gel, eluting with petroleum ether/DCM (2:1, v/v), to yield the product as a pale yellow powder DCz-ND (322 mg, yield: 70%). ^{1}H NMR (500 MHz, $CDCl_3$) δ 8.40 (d, *J* = 8.6 Hz, 1H), 8.22 (d, *J* = 8.3 Hz, 2H), 8.12 (d, *J* = 7.7 Hz, 2H), 7.96 (d, *J* = 8.5 Hz, 1H), 7.51-7.44 (m, 2H), 7.36 (t, *J* = 7.5 Hz, 2H). ^{13}C NMR (126 MHz, $CDCl_3$) δ 154.84, 139.60, 126.82, 125.31, 122.08, 120.44, 117.91, 112.53. HRMS (ESI): m/z calcd for $C_{32}H_{20}N_4$ $[M+H]^+$: 461.1766, found: 461.2060.

II. Photophysical Properties of R/S-PyBN and DPEPO

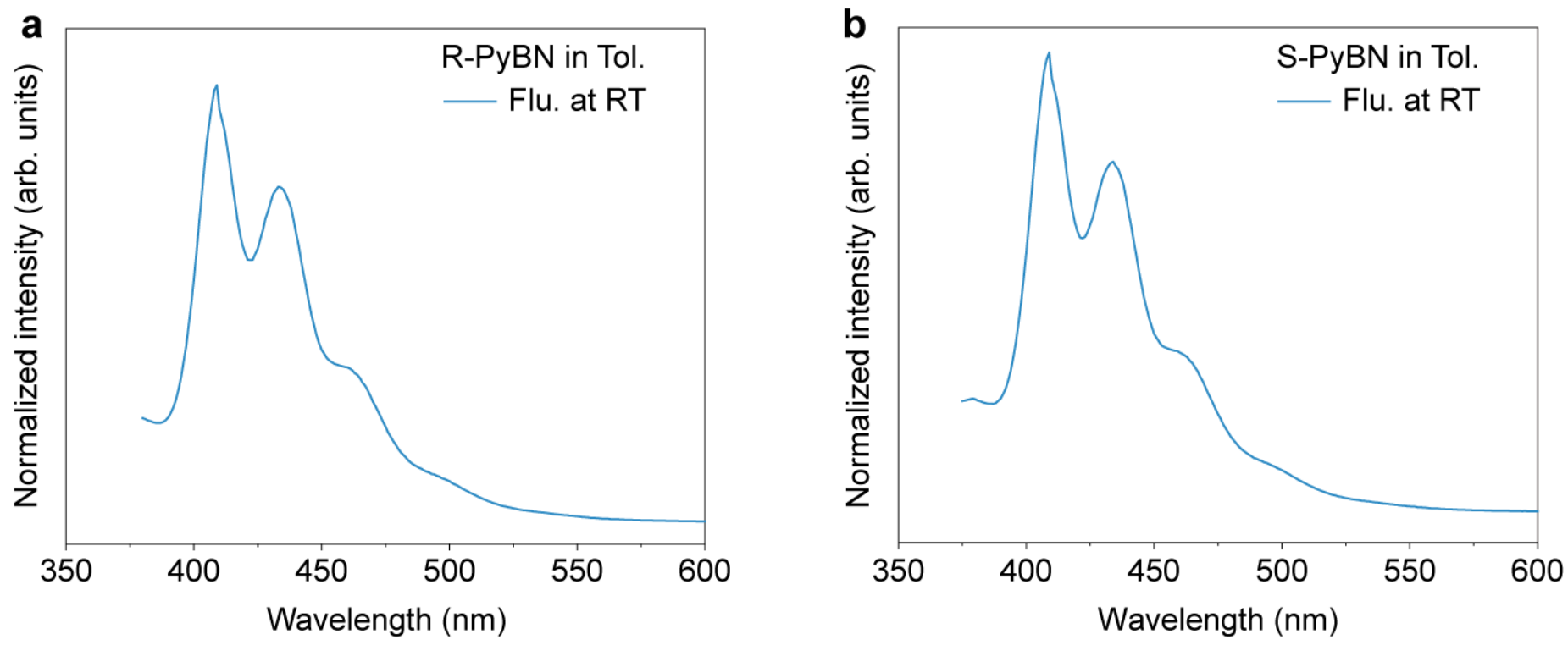

Supplementary Figure 5 | Fluorescence (Flu.) spectra of R/S-PyBN in toluene at RT. a R-PyBN. **b** S-PyBN.

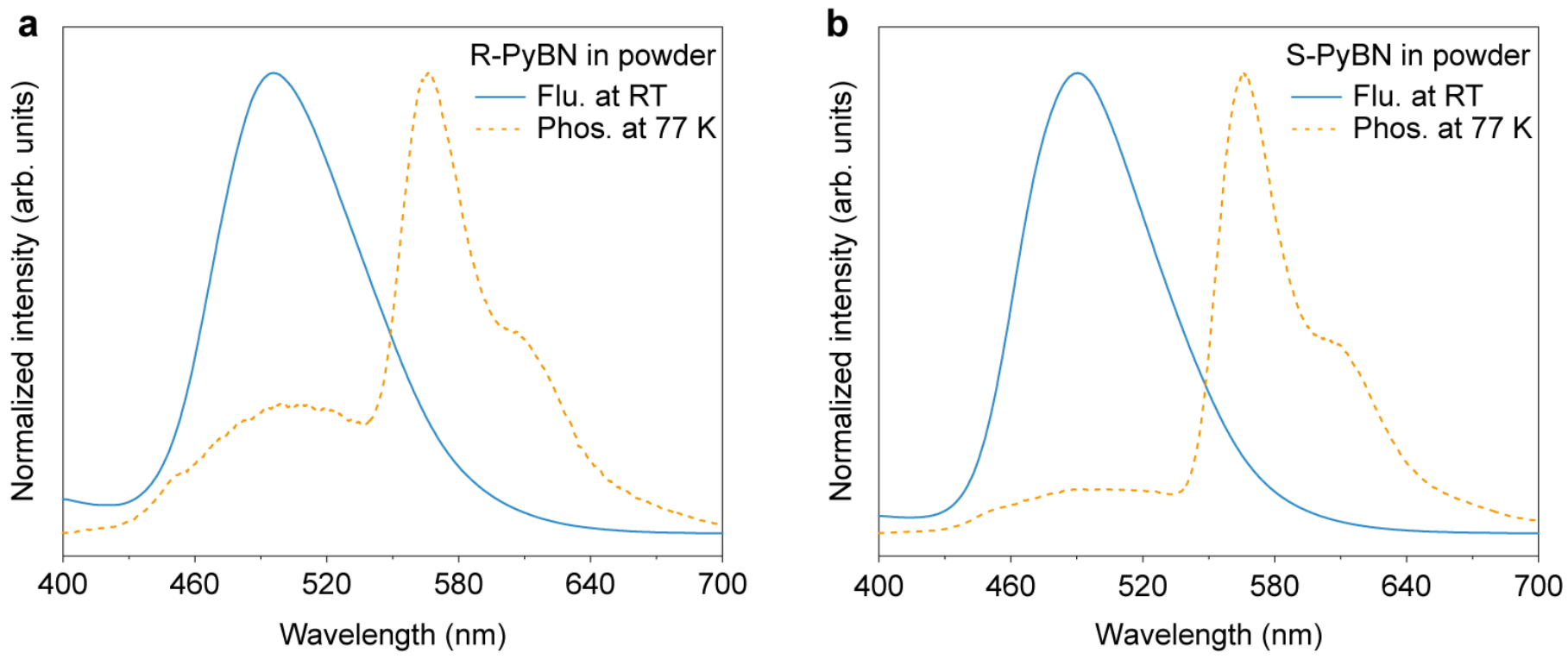

Supplementary Figure 6 | Fluorescence (Flu., at RT) and phosphorescence (Phos., delayed time 8 ms, at 77 K) spectra of R/S-PyBN in powder form. a R-PyBN. **b** S-PyBN.

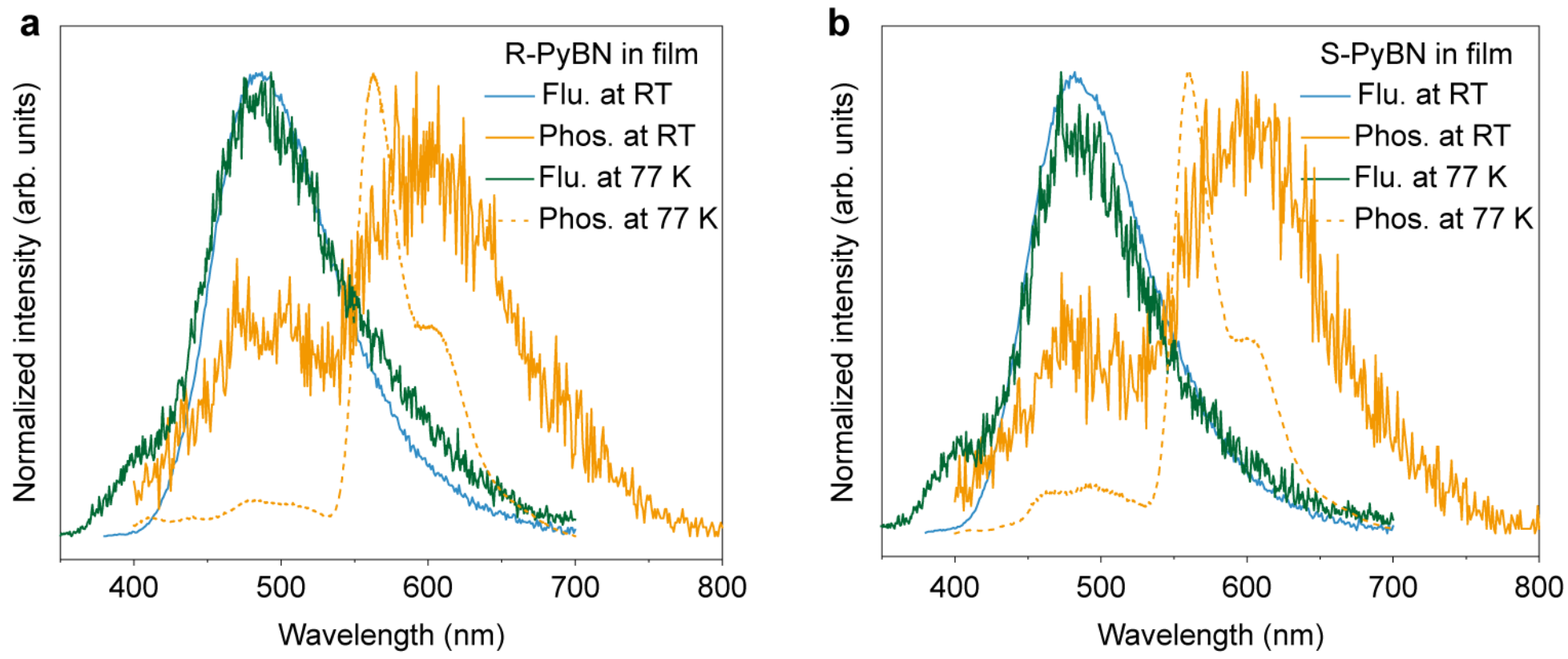

Supplementary Figure 7 | Fluorescence (Flu.) and phosphorescence (Phos.) spectra of R/S-PyBN in film. a R-PyBN. **b** S-PyBN. The delayed time of phosphorescence was 100 μs at RT and 8 ms at 77 K.

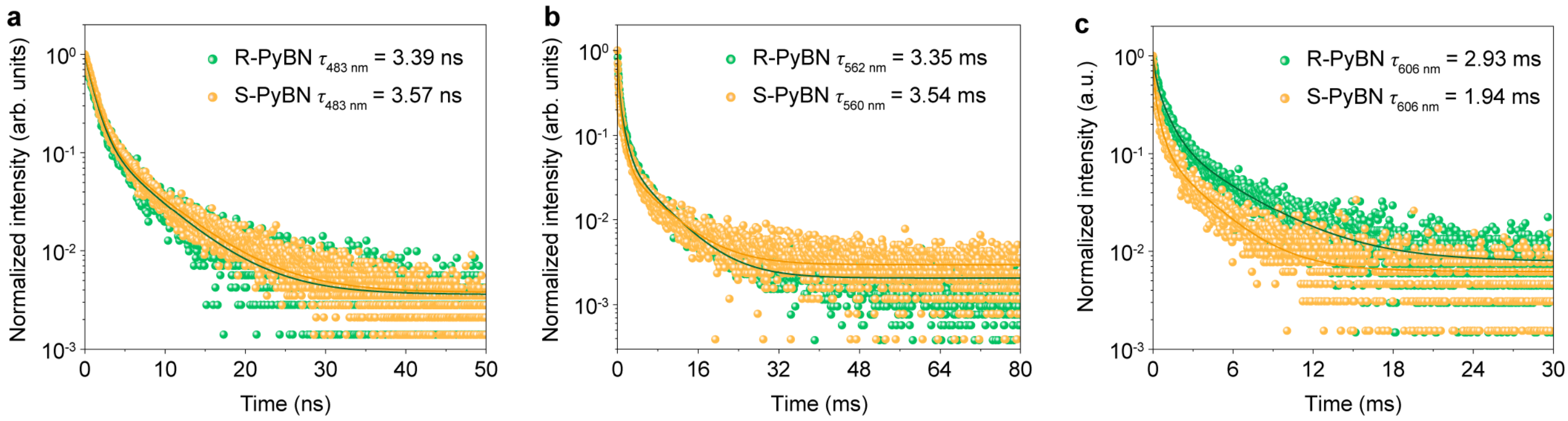

Supplementary Figure 8 | Normalized fluorescence (prompt) lifetime decay curves of the emissions at 483/483 nm (a), 562/560 nm (b) and 606/606 nm (c) for R/S-PyBN films at RT.

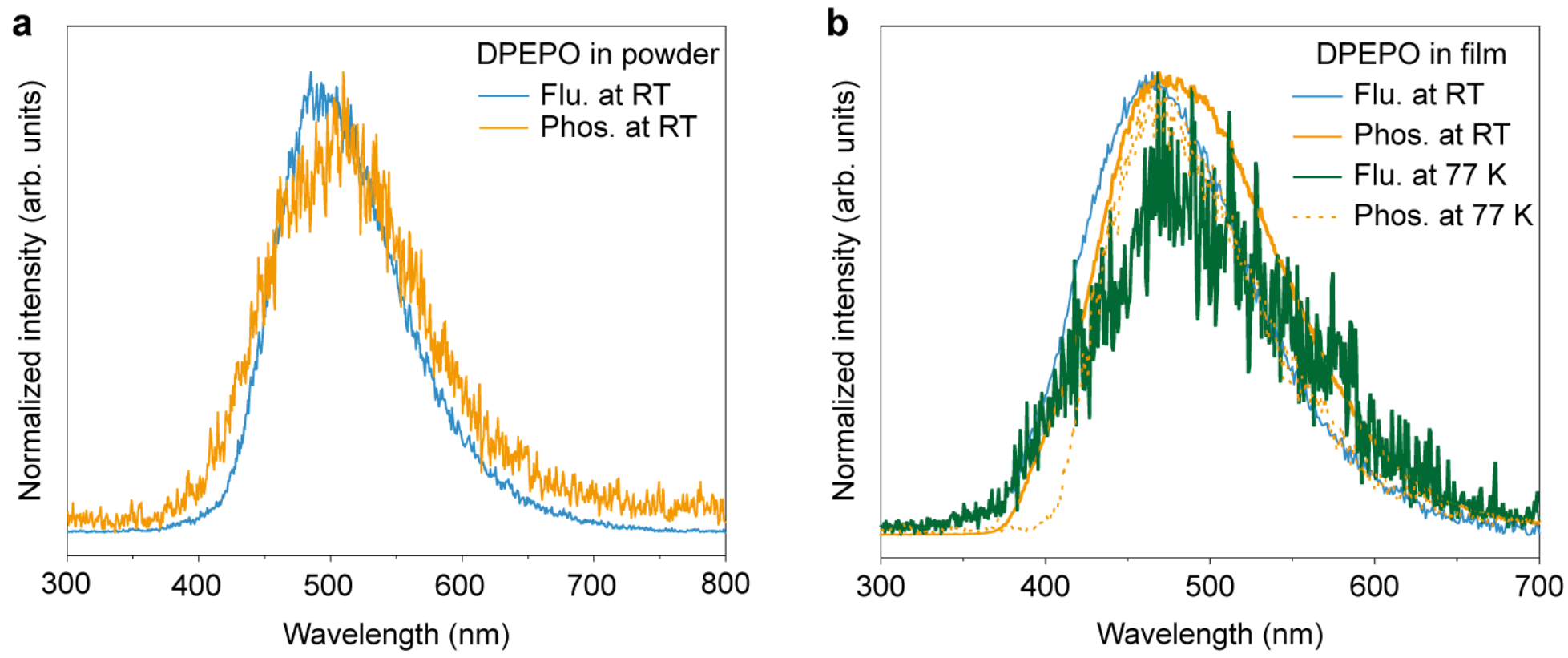

Supplementary Figure 9 | Fluorescence (Flu.) and phosphorescence (Phos.) spectra of DPEPO in powder (a) and in neat film (b). The delayed time of phosphorescence was 5 ms at RT and 8 ms at 77 K.

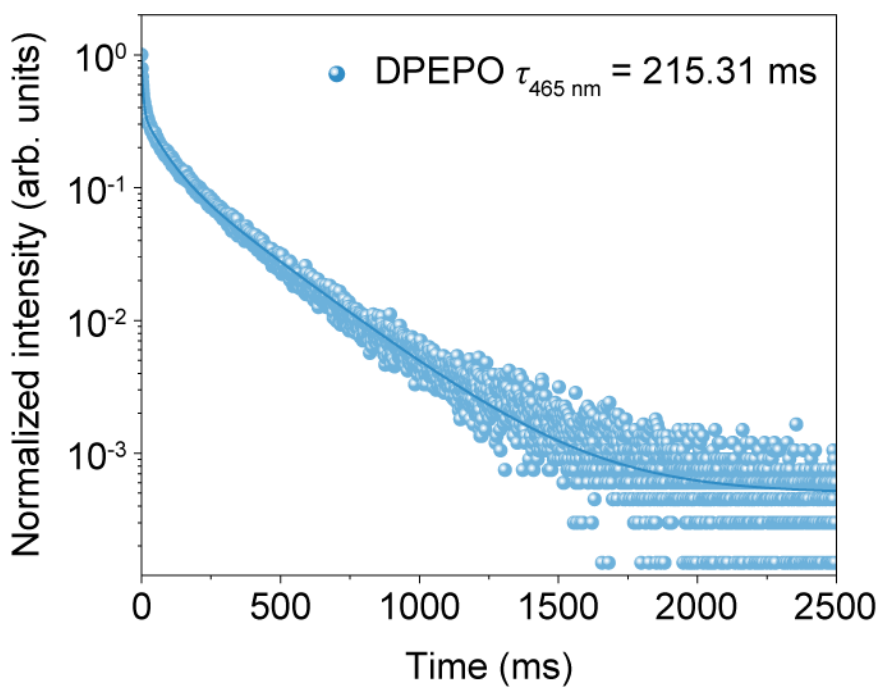

Supplementary Figure 10 | Normalized fluorescence (prompt) lifetime decay curves of the emissions at 465 nm for DPEPO films at 77 K.

Supplementary Table 1 | Fitted fluorescence and phosphorescence lifetimes of R/S-PyBN and DPEPO in neat films.

Film	Wavelength (nm)	Condition	Fluorescence					Phosphorescence						
			τ_1 (ns)	A_1 (%)	τ_2 (ns)	A_2 (%)	τ^a (ns)	τ_1 (ms)	A_1 (%)	τ_2 (ms)	A_2 (%)	τ_3 (ms)	A_3 (%)	τ^a (ms)
**R-PyBN**	483	RT	1.09	50.63	5.74	49.37	3.39	-	-	-	-	-	-	-
	562	RT	-	-	-	-	-	0.19	15.13	0.99	37.54	6.24	47.33	3.35
	606	RT	-	-	-	-	-	0.17	5.55	0.86	35.67	4.44	58.78	2.93
**S-PyBN**	483	RT	1.16	51.49	6.12	48.51	3.57	-	-	-	-	-	-	-
	560	RT	-	-	-	-	-	0.82	40.12	0.11	10.43	6.47	49.46	3.54
	606	RT	-	-	-	-	-	0.08	7.91	0.47	31.56	2.95	60.53	1.94
**DPEPO**	465	77 K	-	-	-	-	-	5.52	4.58	72.89	24.44	277.88	70.98	215.31

[a]Determined from the fitting function of $I = \sum A_i e^{-t/\tau_i}$ according to lifetime decay curves, where A_i is the exponential factor for lifetime τ_i. The average lifetimes of fluorescence/phosphorescence are calculated by $\tau = \sum A_i \tau_i$.

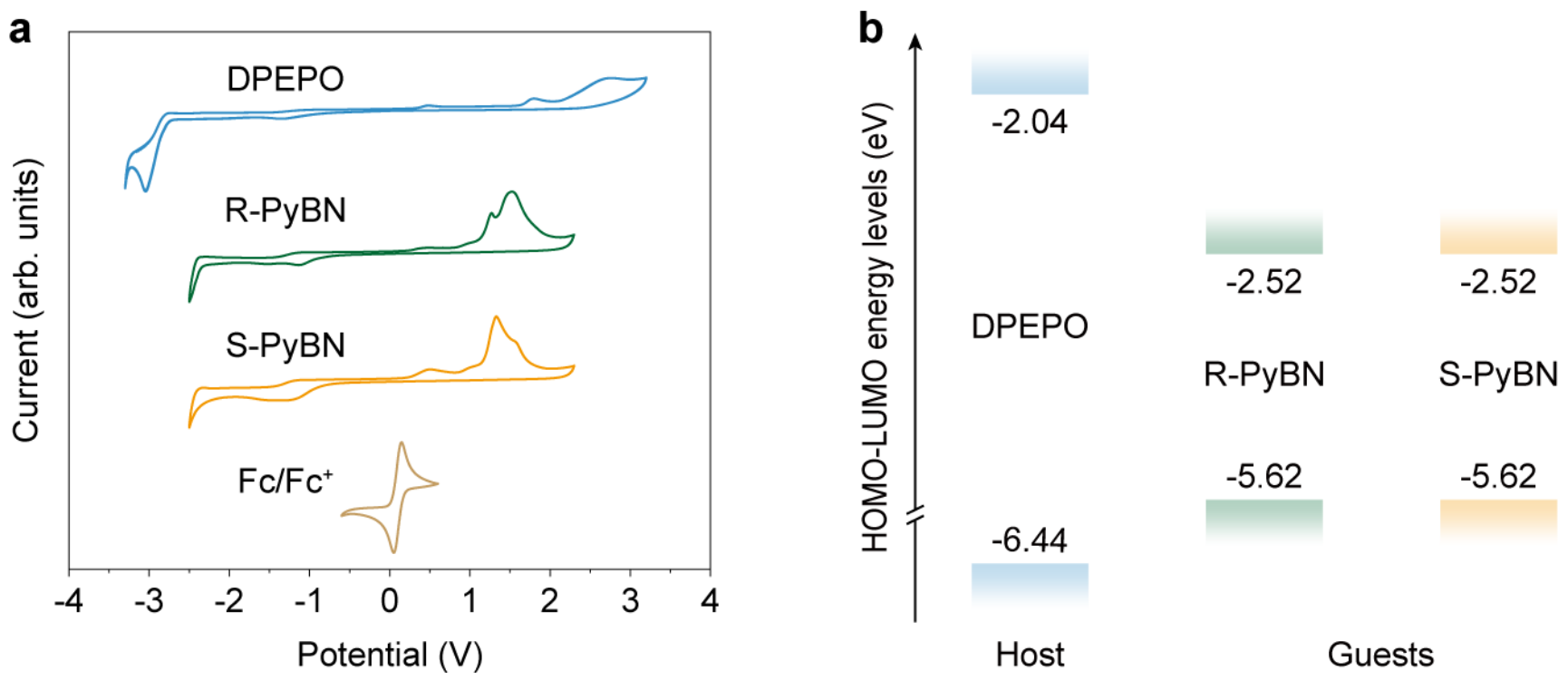

Supplementary Figure 11 | Electrochemical properties and frontier orbital energies. a, Cyclic voltammograms of DPEPO, R-PyBN, and S-PyBN (from top to bottom) in dried MeCN with $TBAPF_6$ recorded versus Fc/Fc^+ at a scan rate of 0.1 V s^{-1} under a N_2 atmosphere. b, Calculated HOMO and LUMO levels of above materials on the basis of the onset of each oxidation and reduction curves.

III. Photophysical Properties of R/S-PyBN@DPEPO Films

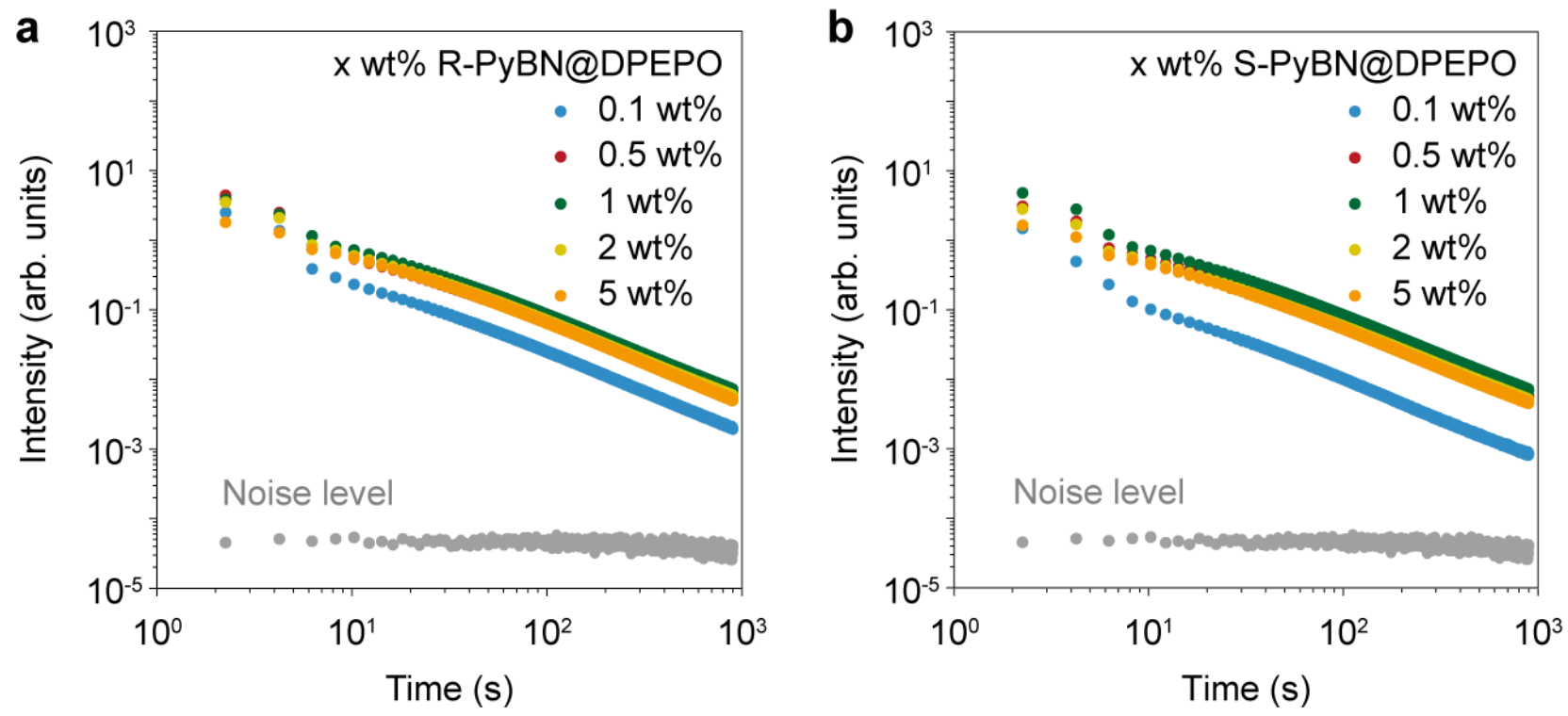

Supplementary Figure 12 | PersL decay curves of x wt% R-PyBN@DPEPO (a) and S-PyBN@DPEPO (b) with different doping concentration at RT (x = 0.1, 0.5, 1, 2, and 5 wt%, excitation time = 60 s).

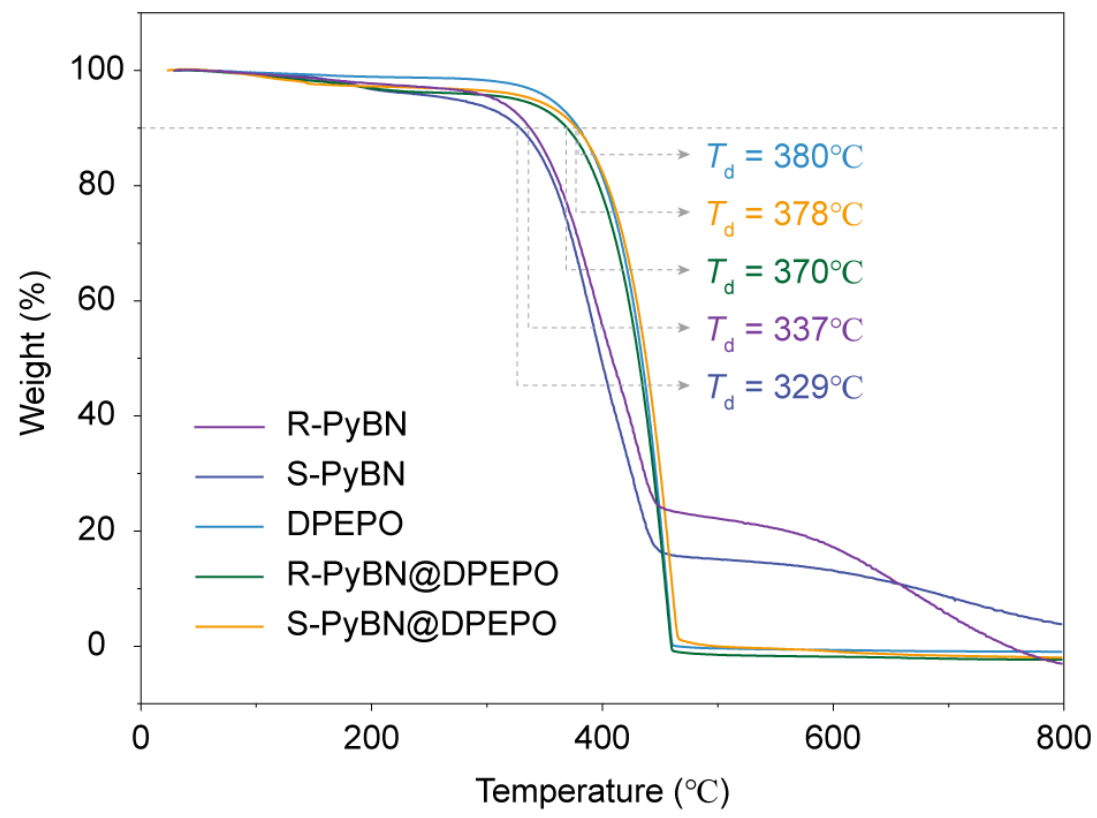

Supplementary Figure 13 | Thermogravimetric analysis (TGA) curves of R/S-PyBN, DPEPO, and R/S-PyBN@DPEPO under a nitrogen atmosphere.

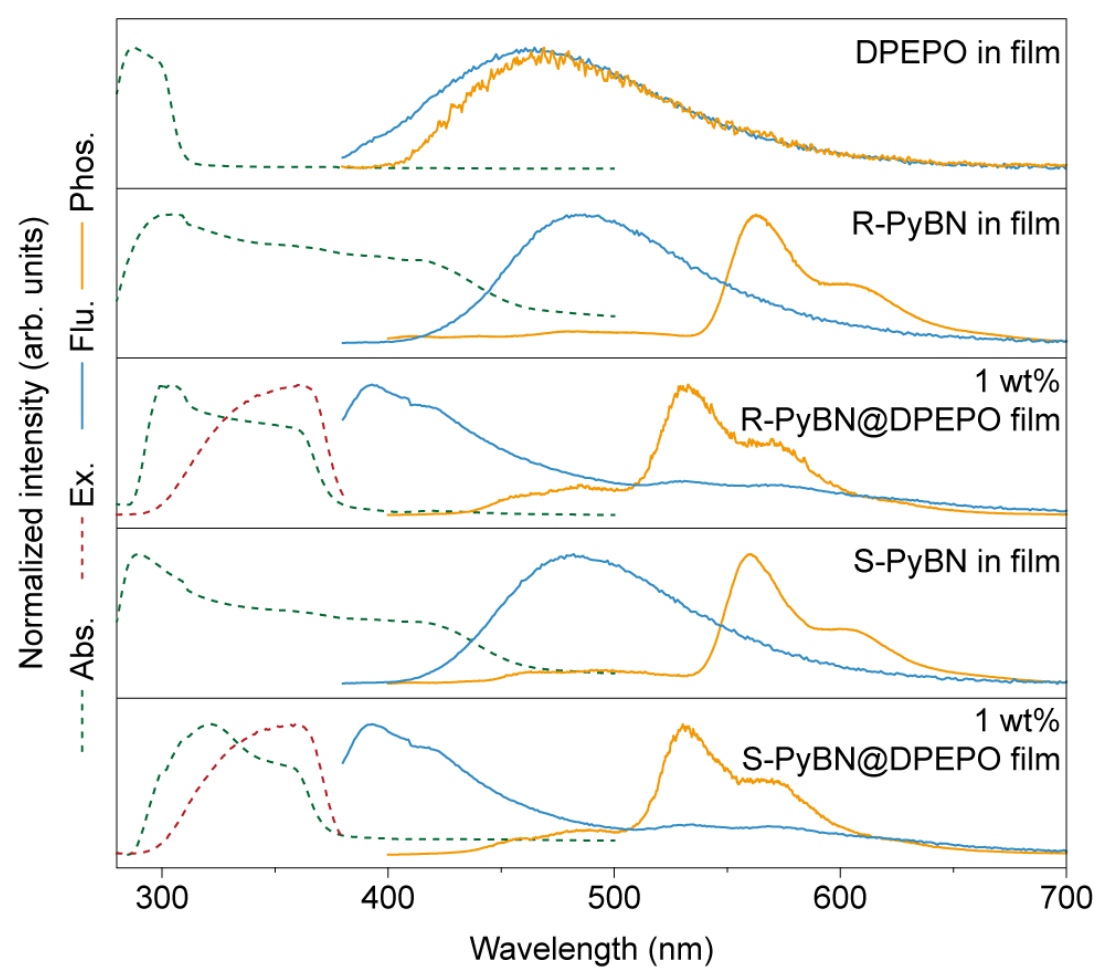

Supplementary Figure 14 | Ultraviolet-visible absorption (Abs., green curves), excitation (Ex., red curves), fluorescence (Flu., blue curves), and phosphorescence (Phos., orange curves, λ_{ex} = 365 nm, delayed time 8 ms, at 77 K) spectra of DPEPO in a neat film, R/S-PyBN in neat films, and 1 wt% R/S-PyBN@DPEPO in melt-casting films.

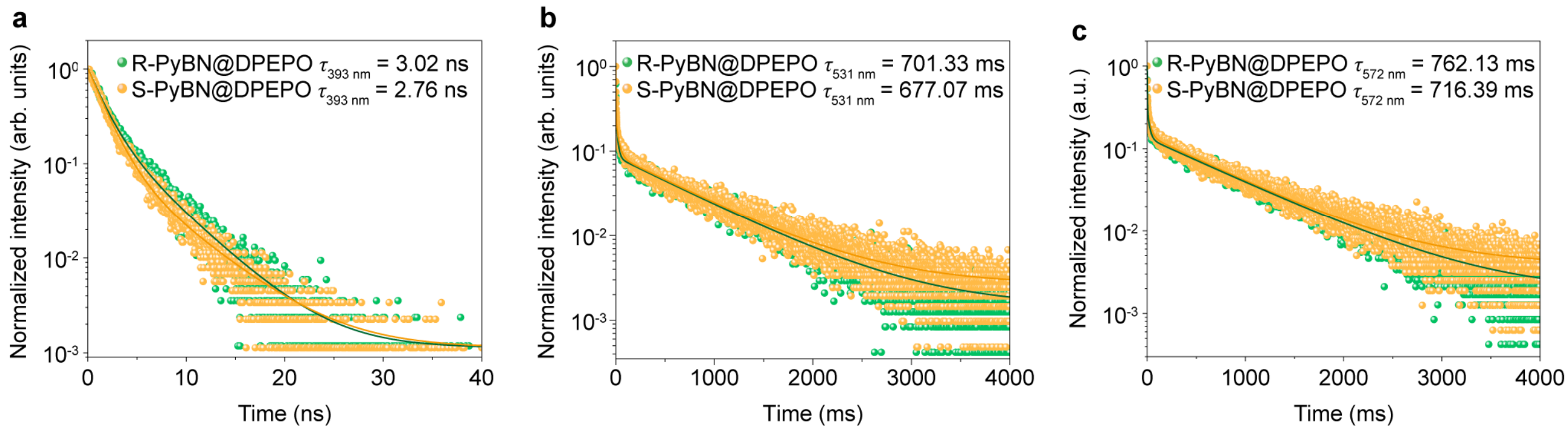

Supplementary Figure 15 | Normalized fluorescence (prompt) lifetime decay curves of the emission bands at 393/393 nm (a), 531/531 nm (b) and 572/572 nm (c) for 1 wt% R/S-PyBN@DPEP in melt-casting films at RT.

Supplementary Table 2 | Fitted fluorescence and phosphorescence lifetimes of R/S-PyBN@DPEPO films.

Film	Wavelength (nm)	Condition	Fluorescence					Phosphorescence						
			τ_1 (ns)	A_1 (%)	τ_2 (ns)	A_2 (%)	τ^a (ns)	τ_1 (ms)	A_1 (%)	τ_2 (ms)	A_2 (%)	τ_3 (ms)	A_3 (%)	τ^a (ms)
**R-PyBN@DPEPO**	393	RT	1.48	48.30	4.46	51.70	3.02	-	-	-	-	-	-	-
	531	RT	-	-	-	-	-	1.6	1.78	17.39	5.11	752.27	93.11	701.33
	572	RT	-	-	-	-	-	1.94	1.25	22.87	3.17	796.59	95.58	762.13
**S-PyBN@DPEPO**	393	RT	1.58	67.45	5.22	32.55	2.76	-	-	-	-	-	-	-
	531	RT	-	-	-	-	-	2.16	2.15	28.06	6.20	736.84	91.65	677.07
	572	RT	-	-	-	-	-	1.82	1.07	27.71	4.45	756.91	94.48	716.39

[a]Determined from the fitting function of $I = \sum A_i e^{-t/\tau_i}$ according to lifetime decay curves, where A_i is the exponential factor for lifetime τ_i. The average lifetimes of fluorescence/phosphorescence are calculated by $\tau = \sum A_i \tau_i$.

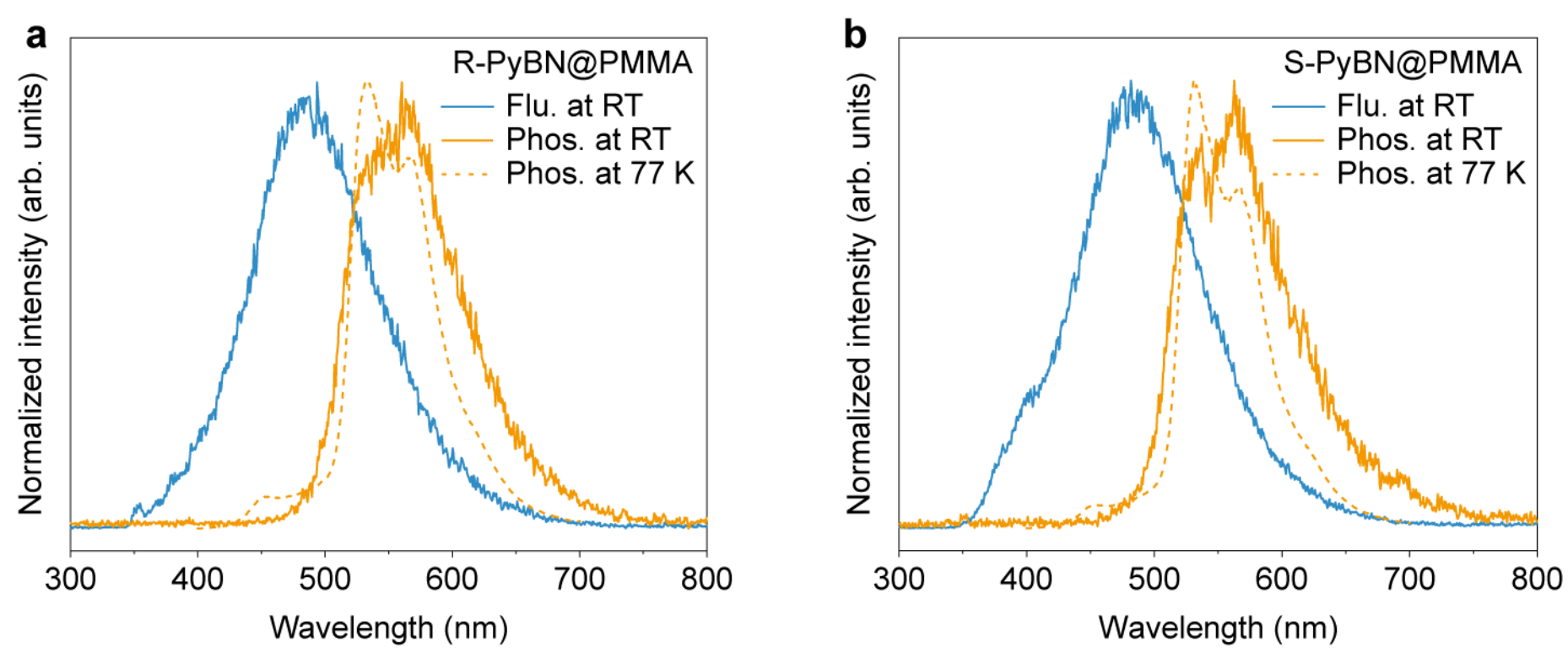

Supplementary Figure 16 | Fluorescence (Flu.) and phosphorescence (Phos.) spectra of 1 wt% R-PyBN@PMMA (a) and S-PyBN@PMMA (b) films. The delayed time of phosphorescence was 5 ms at RT and 8 ms at 77 K.

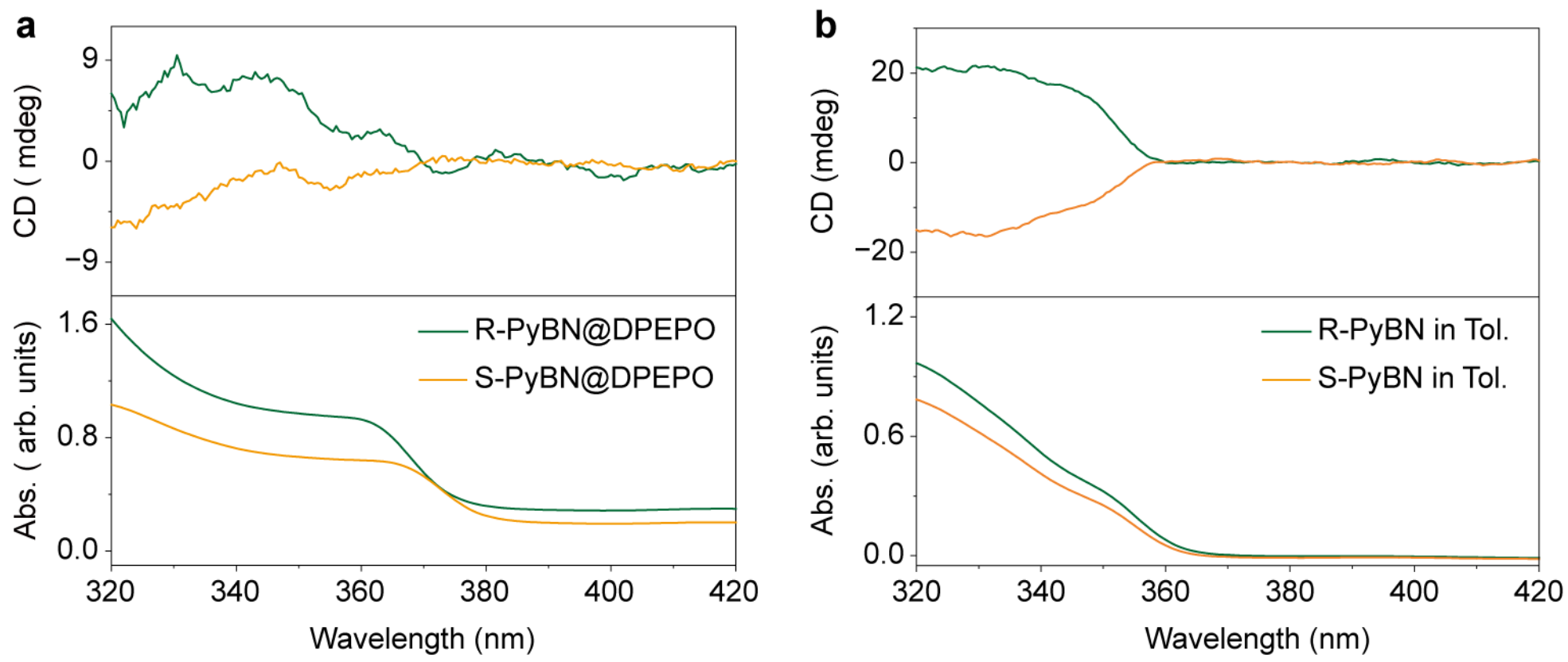

Supplementary Figure 17 | Circular dichroism (CD, top) and ultraviolet-visible absorption (Abs., bottom) spectra of R/S-PyBN@DPEPO in melt-casting films (a) and R/S-PyBN in toluene (b) at RT.

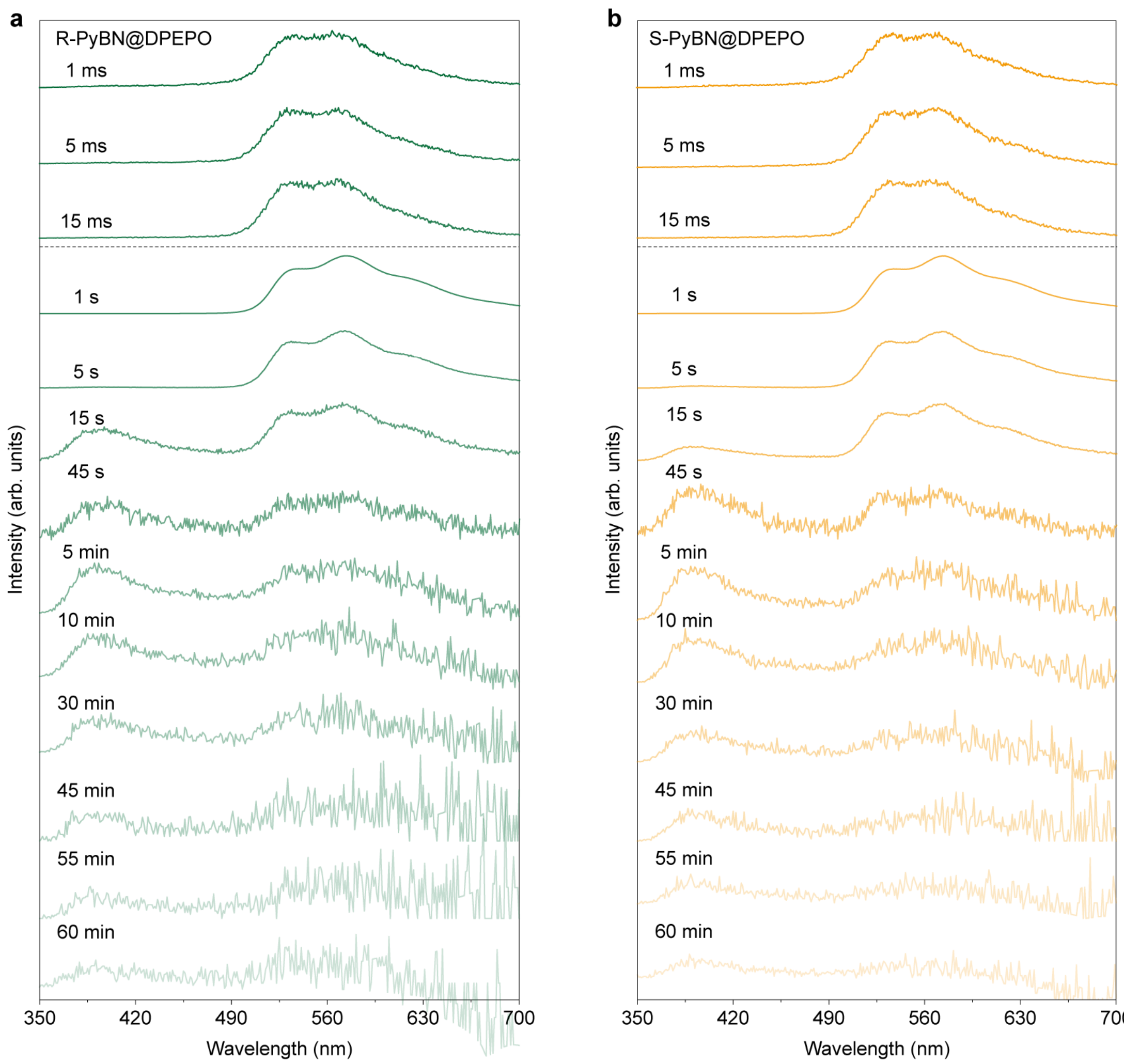

Supplementary Figure 18 | Time-resolved emission spectra of R-PyBN@DPEPO (a) and S-PyBN@DPEPO (b) films. The spectra recorded at 1, 5, and 15 ms were obtained after cessation of 355 nm short-pulse excitation, whereas the spectra from 1 s to 60 min were collected after 1 min of continuous 365 nm excitation.

IV. Trap Characteristics of R/S-PyBN@DPEPO Films

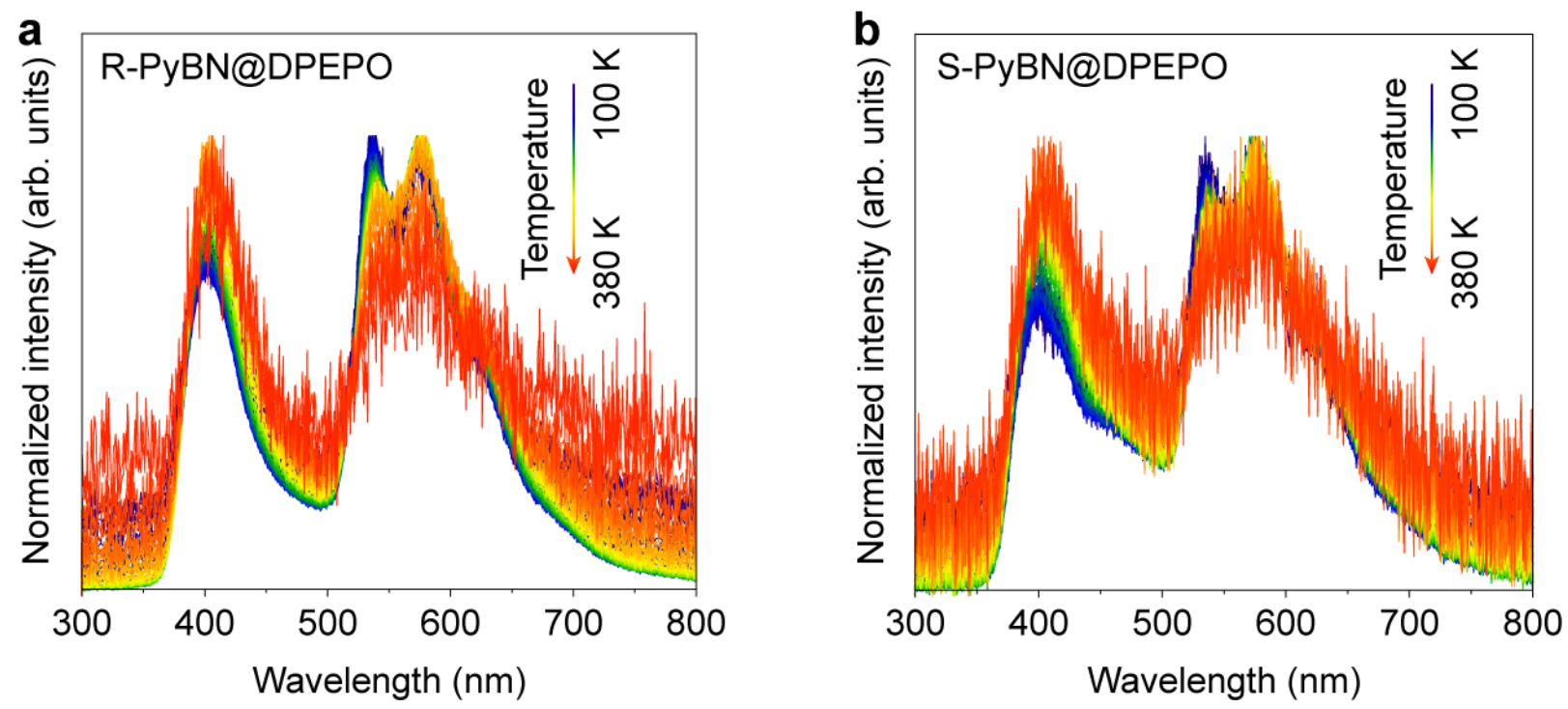

Supplementary Figure 19 | Normalized emission spectra of R-PyBN@DPEPO (a) and S-PyBN@DPEPO (b) films at different temperature extracted from the contour mapping of thermoluminescence (TL) with a heating rate of 50 K min^{-1} (redraw from Fig. 2d).

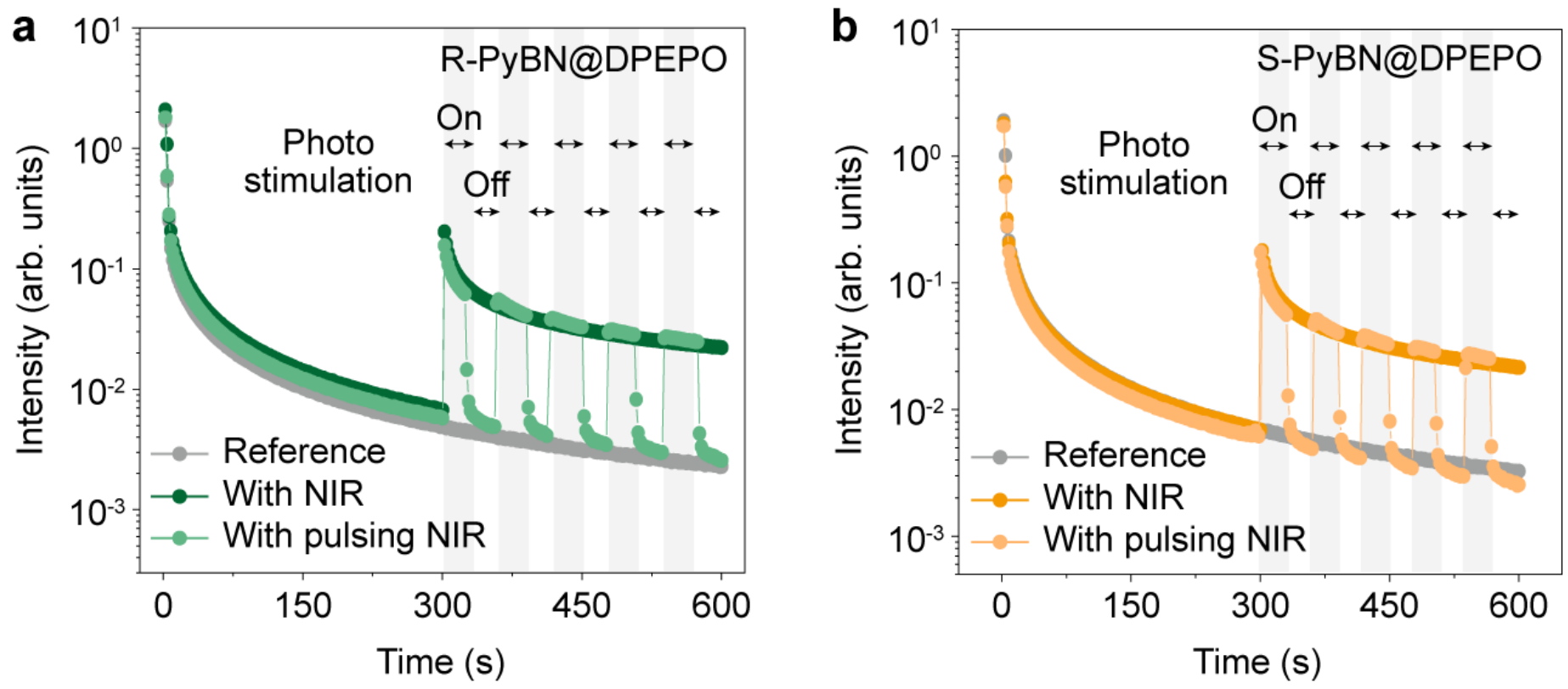

Supplementary Figure 20 | PersL decay curves of 1 wt% R-PyBN@DPEPO (a) and S-PyBN@DPEPO (b) films with 980 nm NIR photo-stimulation in a continuous output mode, NIR photo-stimulation in a pulsed mode (repeating on and off every 30 s), and without NIR photostimulation.

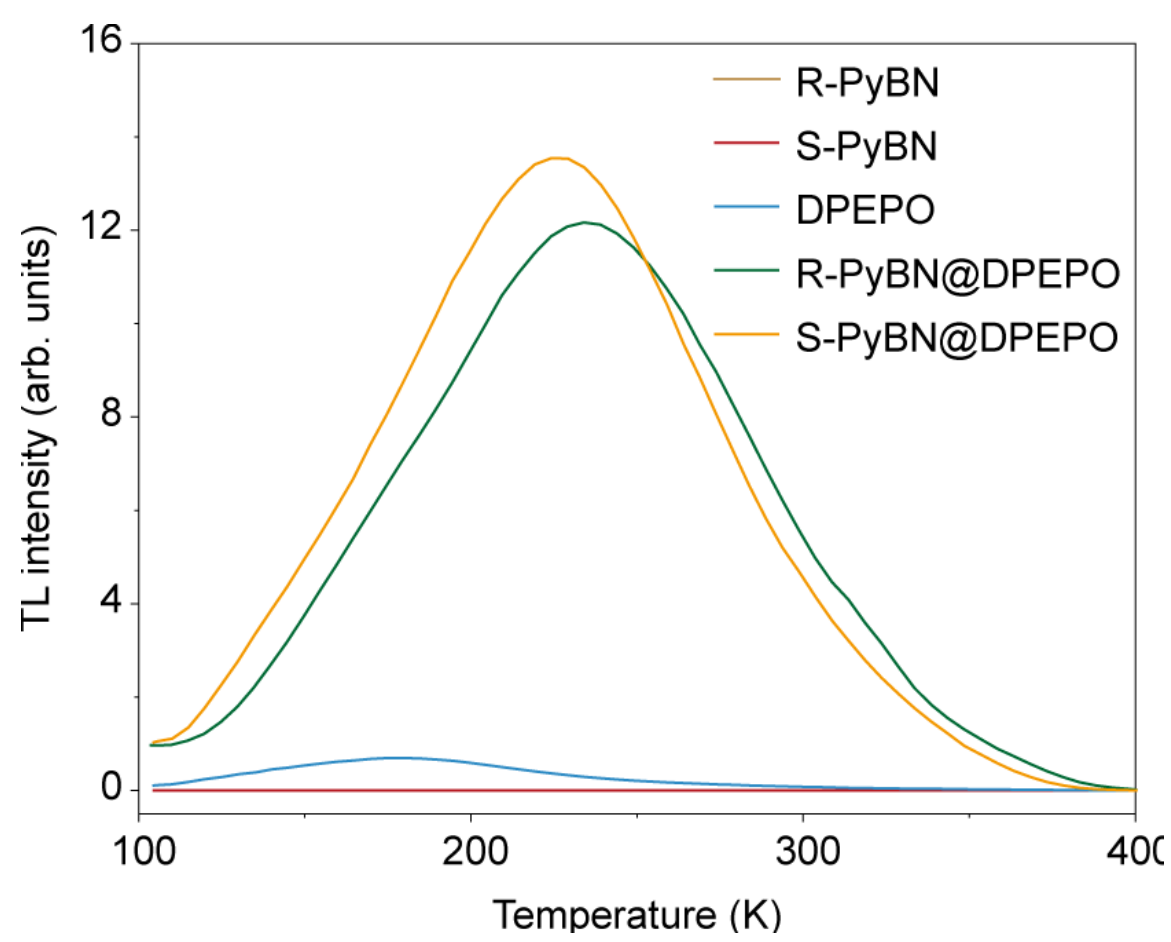

Supplementary Figure 21 | TL glow curves of R/S-PyBN, DPEPO, and R/S-PyBN@DPEPO with a heating rate of 50 K min^{-1}. The samples were irradiated by UV light for 300 s before the TL measurements.

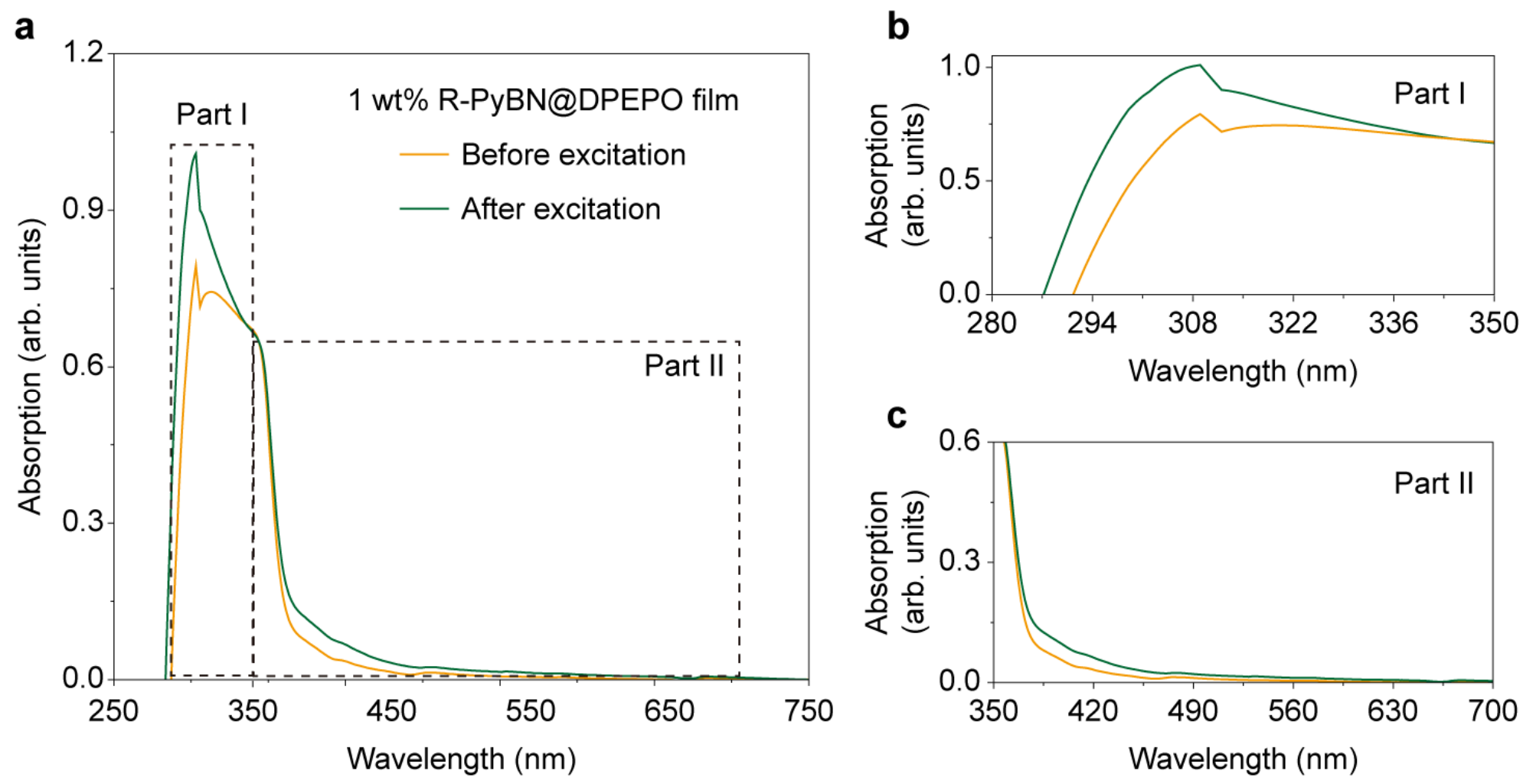

Supplementary Figure 22 | Absorption spectra of 1 wt% R-PyBN@DPEPO film before and after 365 nm excitation. a, Full-range absorption spectra. b, c, Magnified views of the 280–350 nm (b) and 350–700 nm (c) regions. The sample before excitation was wrapped in aluminium foil and stored in the dark for 24 h prior to measurement.

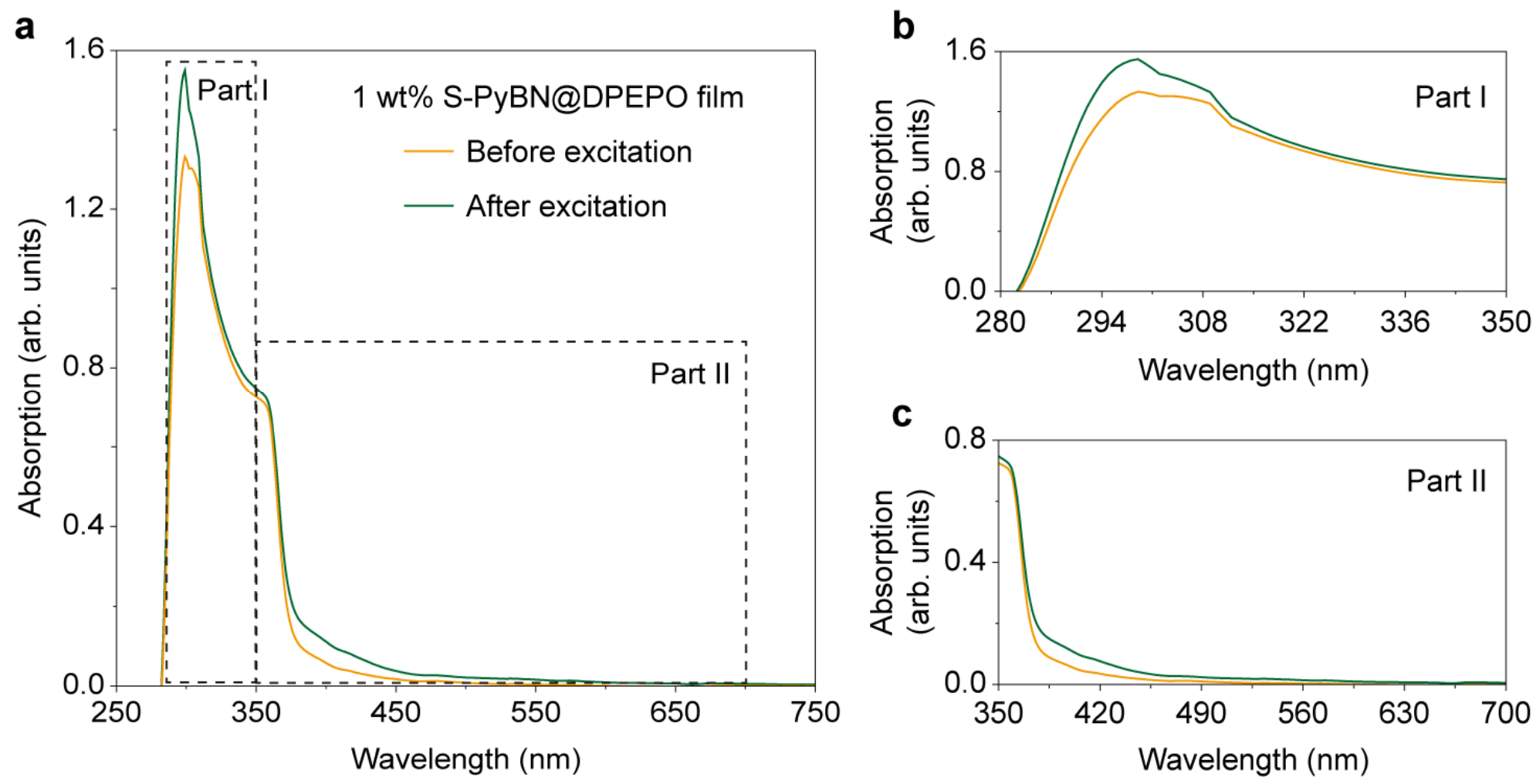

Supplementary Figure 23 | Absorption spectra of 1 wt% S-PyBN@DPEPO film before and after 365 nm excitation. a, Full-range absorption spectra. b, c, Magnified views of the 280–350 nm (b) and 350–700 nm (c) regions. The sample before excitation was wrapped in aluminium foil and stored in the dark for 24 h prior to measurement.

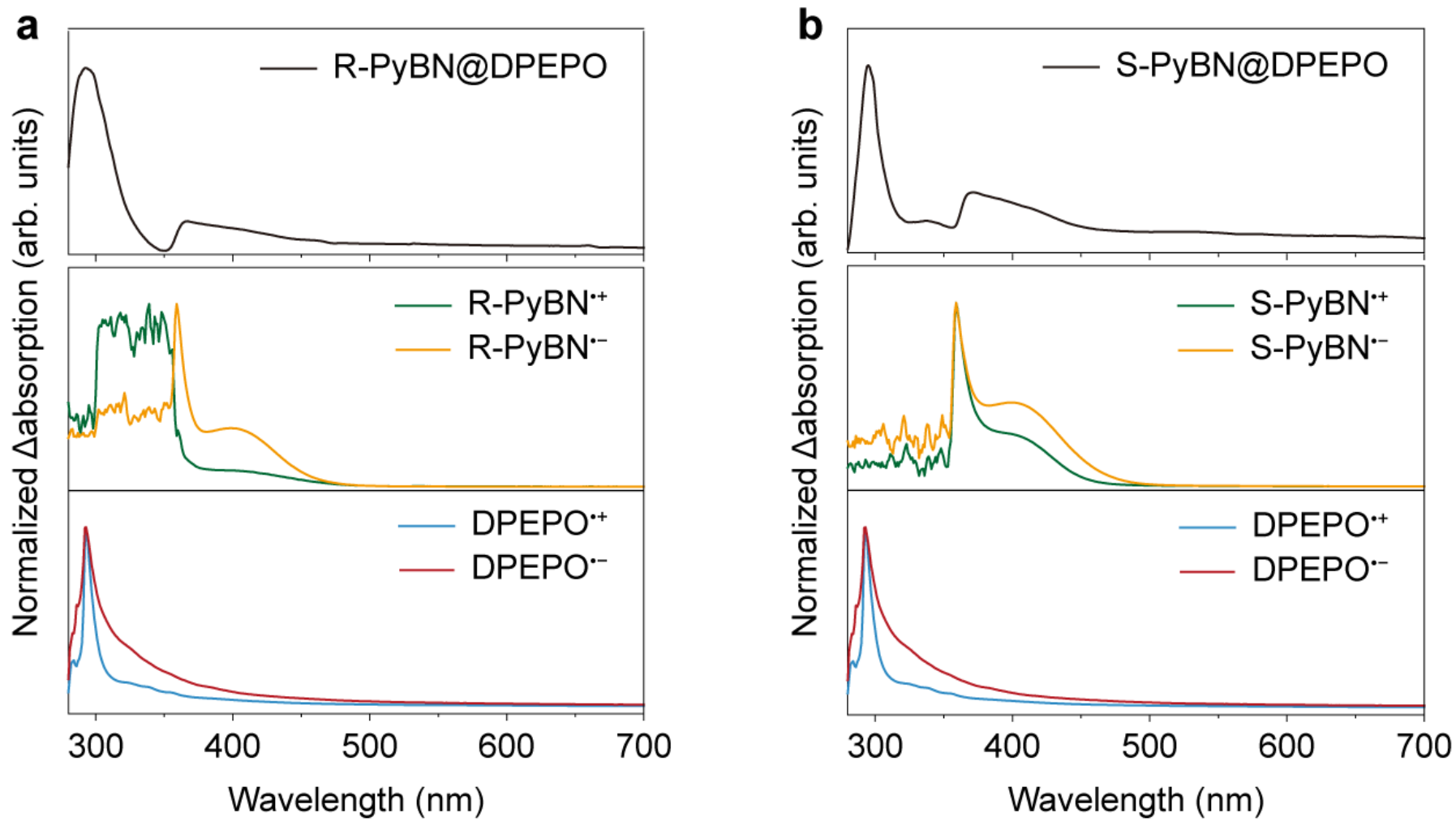

Supplementary Figure 24 | Self-absorption spectra (black curves, top) obtained by the differences in absorptions of 1 wt% R-PyBN@DPEPO (a) and S-PyBN@DPEPO (b) films after and before the 365 nm excitation. The middle and bottom figures are the reference absorption spectra of R/S-PyBN radical cation (R/S-PyBN$^{\bullet+}$, green curves), guest radical anions (R/S-PyBN$^{\bullet-}$, orange curves), DPEPO radical cation (DPEPO$^{\bullet+}$, blue curves) and host radical anions (DPEPO$^{\bullet-}$, red curves) in dichloromethane.

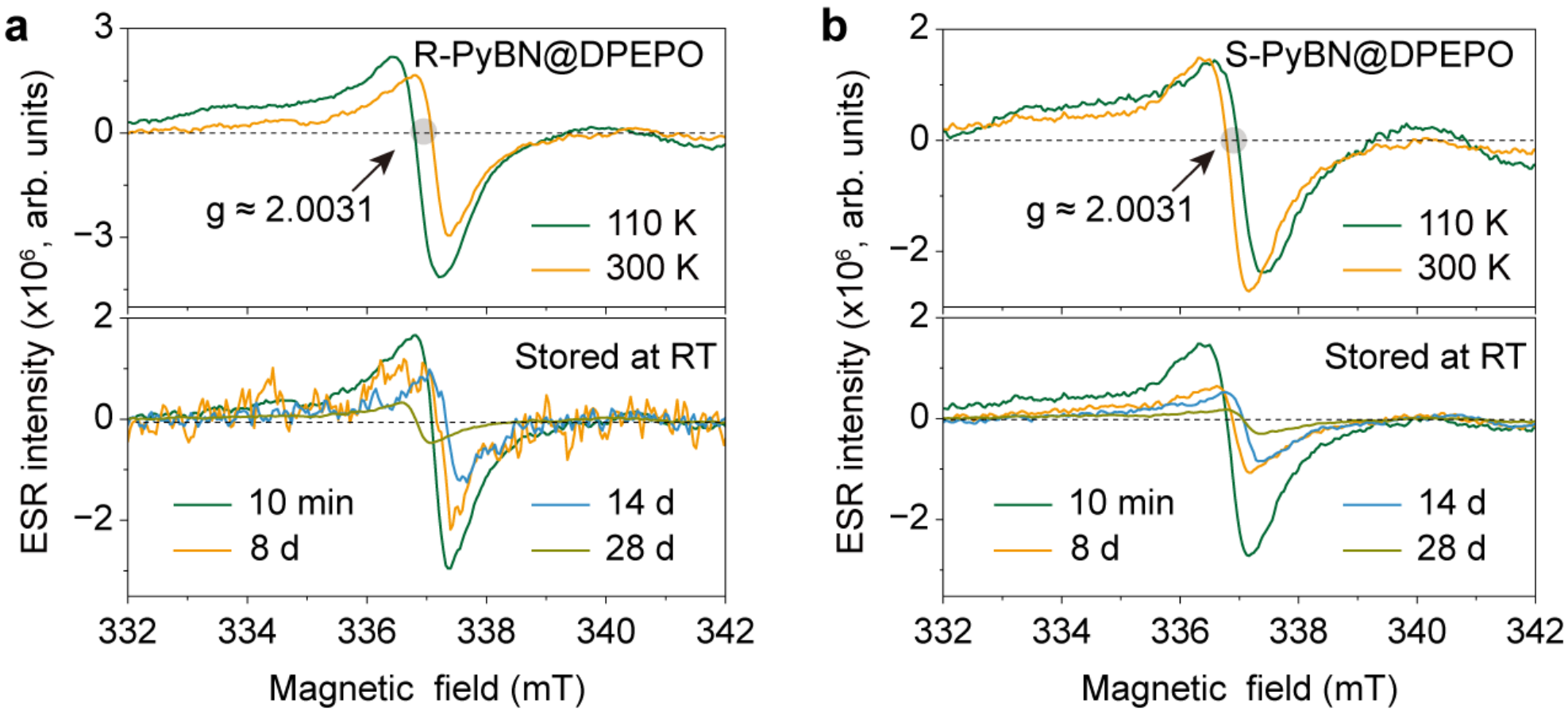

Supplementary Figure 25 | ESR spectra of 1 wt% R-PyBN@DPEPO (a) and S-PyBN@DPEPO (b) films recorded after UV irradiation at 110 and 300 K (top) and after 365 nm excitation at different storage time (bottom, from 10 minutes to 28 days).

V. Theoretical Calculations of Frontier Molecular Orbitals (FMOs)

Supplementary Table 3 | Schematic diagrams showing the chemical structure and TD-DFT-calculated energy levels and main orbital configurations of DPEPO, BCPO, SPPO13, R/S-PyBN, R/S-APyBN, and R/S-PyBNMO molecules and that of radical cations and anions.

Name	Structure	Calculated LUMO and HOMO distribution (eV)				
		Molecule	Radical cation (*α*)	Radical cation (*β*)	Radical anion (*α*)	Radical anion (*β*)
DPEPO	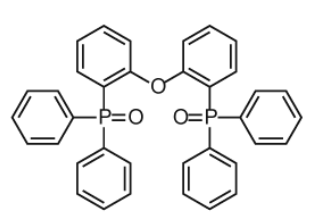	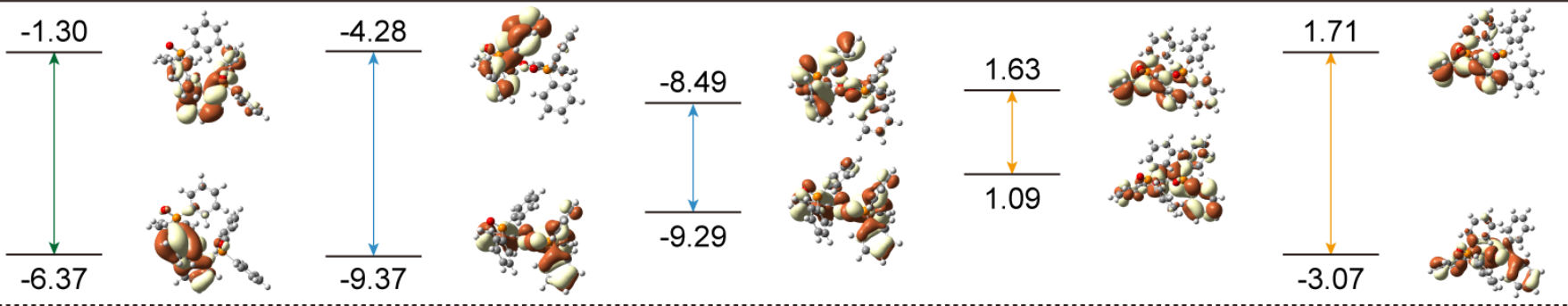 -1.30 / -6.37	-4.28 / -9.37	-8.49 / -9.29	1.63 / 1.09	1.71 / -3.07
BCPO	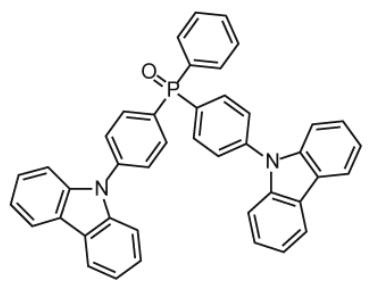	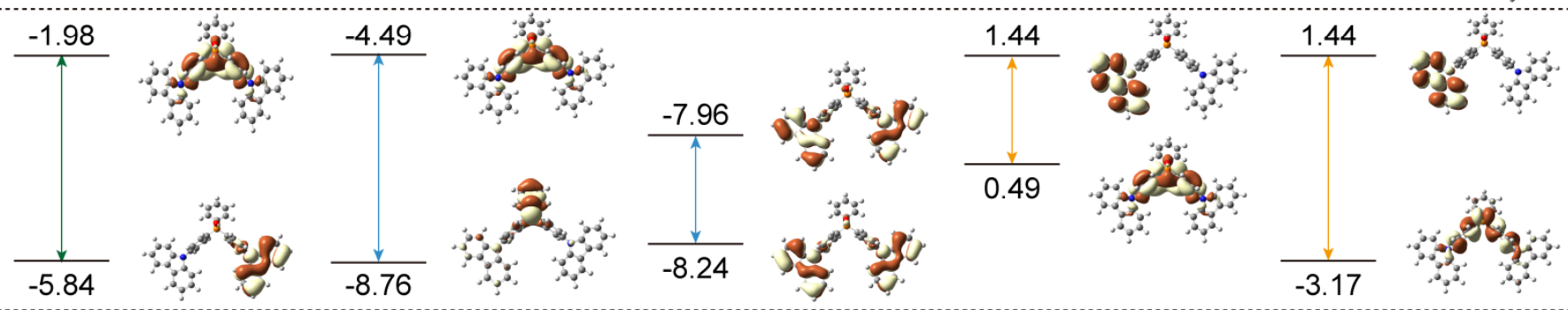 -1.98 / -5.84	-4.49 / -8.76	-7.96 / -8.24	1.44 / 0.49	1.44 / -3.17
SPPO13	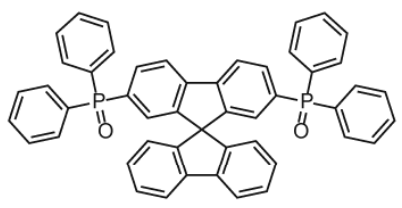	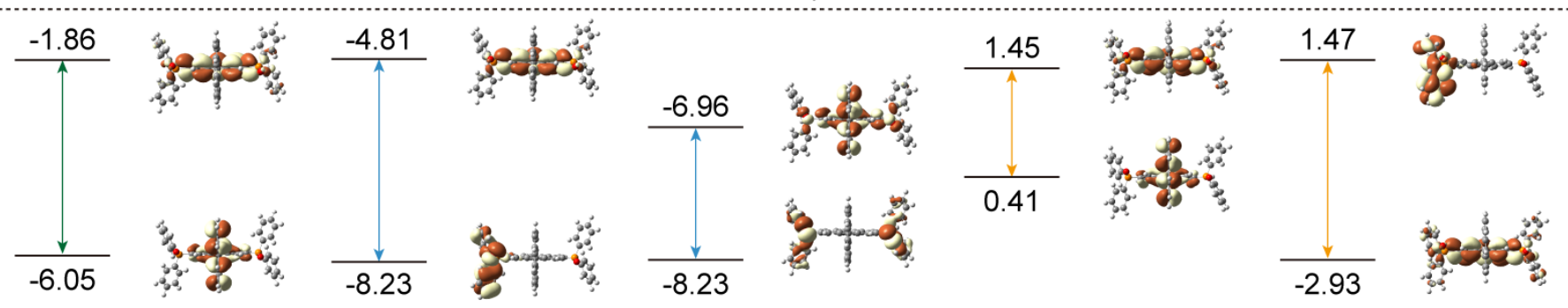 -1.86 / -6.05	-4.81 / -8.23	-6.96 / -8.23	1.45 / 0.41	1.47 / -2.93
R-PyBN	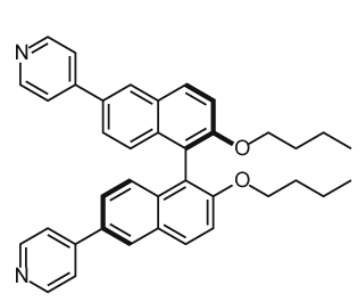	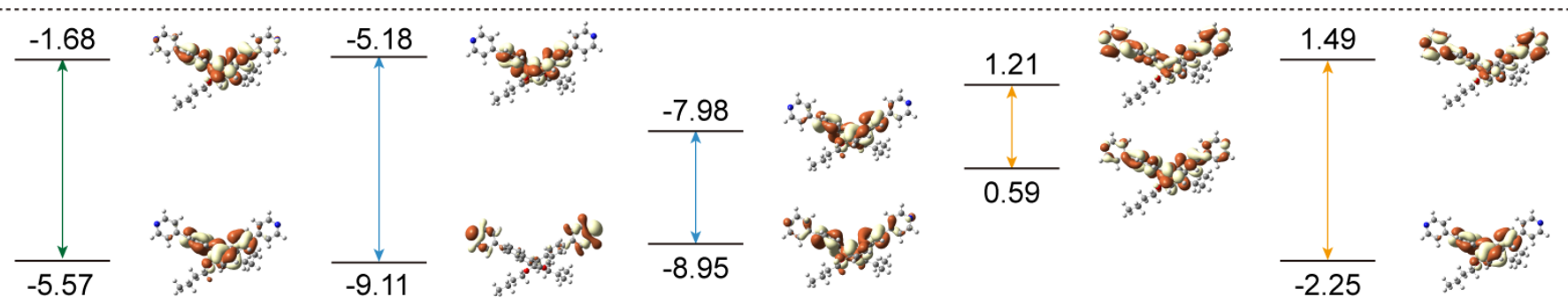 -1.68 / -5.57	-5.18 / -9.11	-7.98 / -8.95	1.21 / 0.59	1.49 / -2.25
S-PyBN	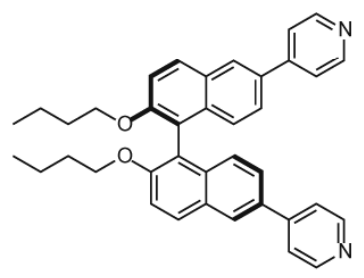	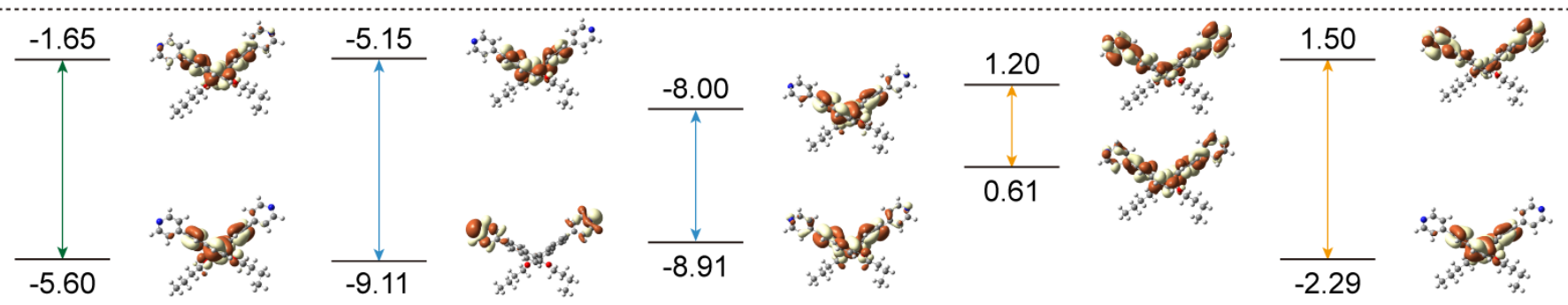 -1.65 / -5.60	-5.15 / -9.11	-8.00 / -8.91	1.20 / 0.61	1.50 / -2.29
R-APyBN	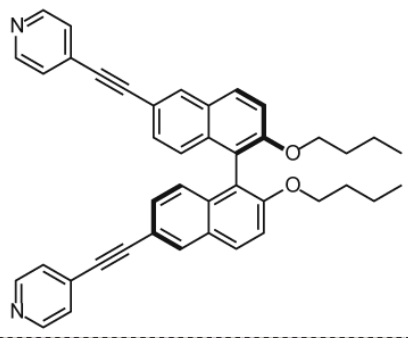	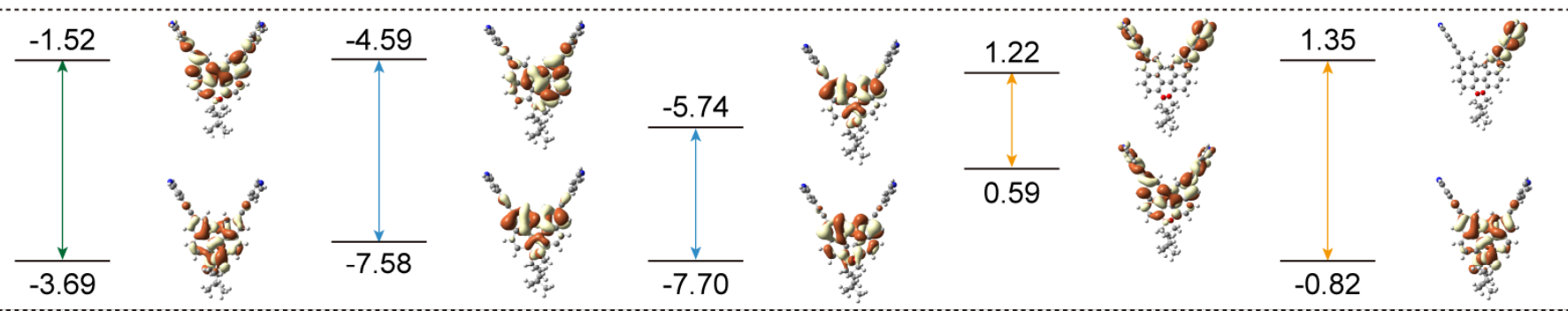 -1.52 / -3.69	-4.59 / -7.58	-5.74 / -7.70	1.22 / 0.59	1.35 / -0.82
S-APyBN	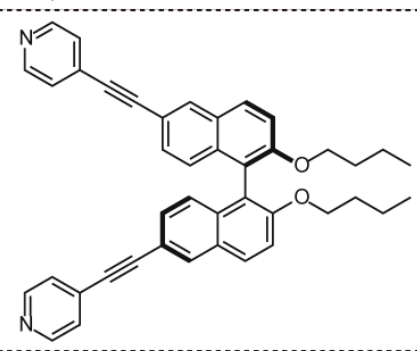	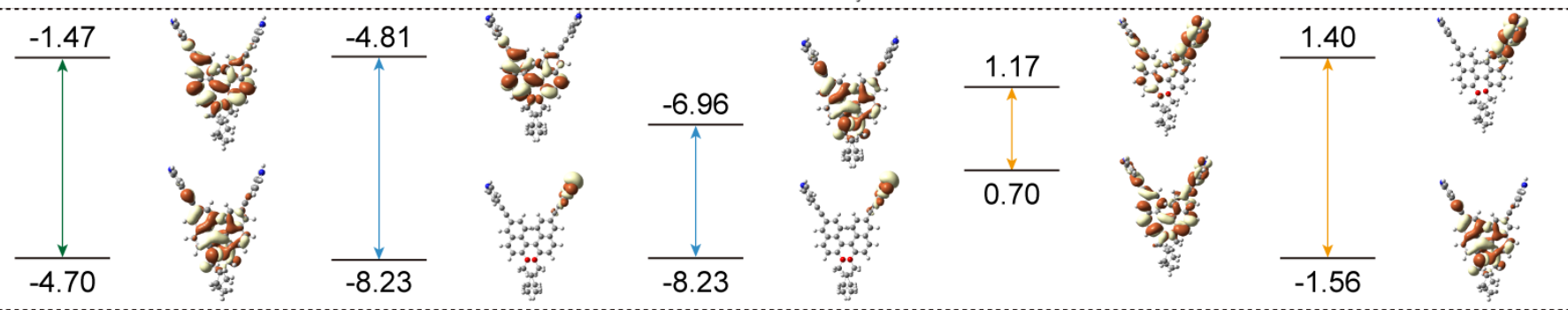 -1.47 / -4.70	-4.81 / -8.23	-6.96 / -8.23	1.17 / 0.70	1.40 / -1.56
R-PyBNMO	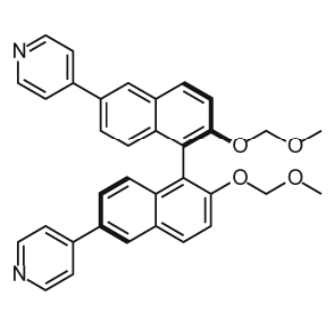	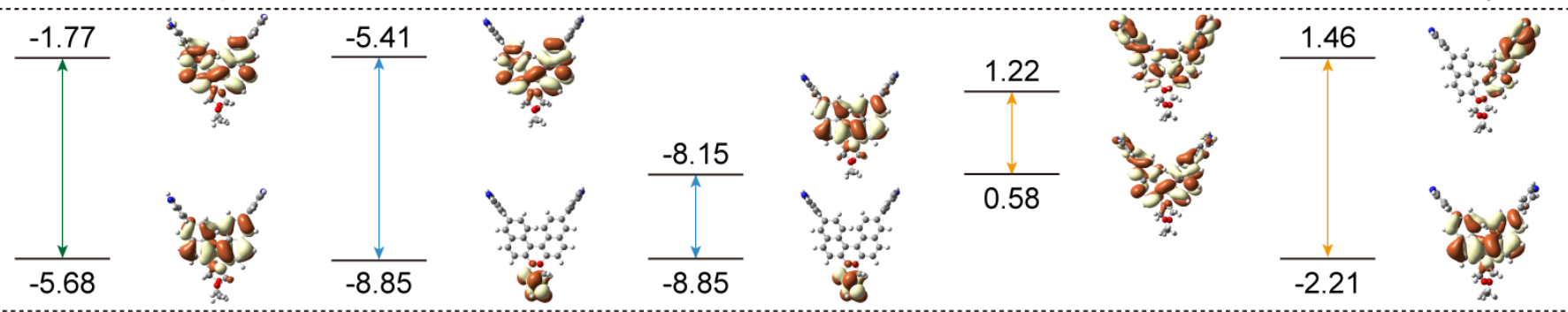 -1.77 / -5.68	-5.41 / -8.85	-8.15 / -8.85	1.22 / 0.58	1.46 / -2.21
S-PyBNMO	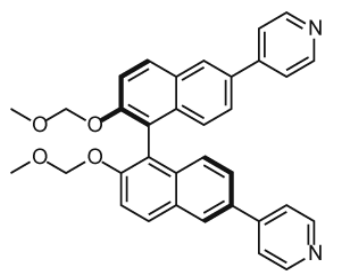	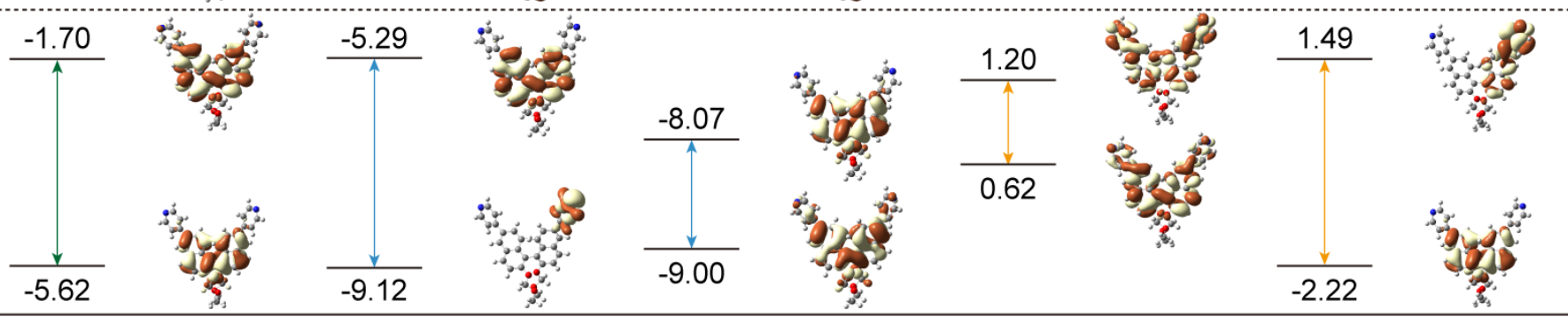 -1.70 / -5.62	-5.29 / -9.12	-8.07 / -9.00	1.20 / 0.62	1.49 / -2.22

VI. Other Guest-Host Systems Showing White CP-PersL

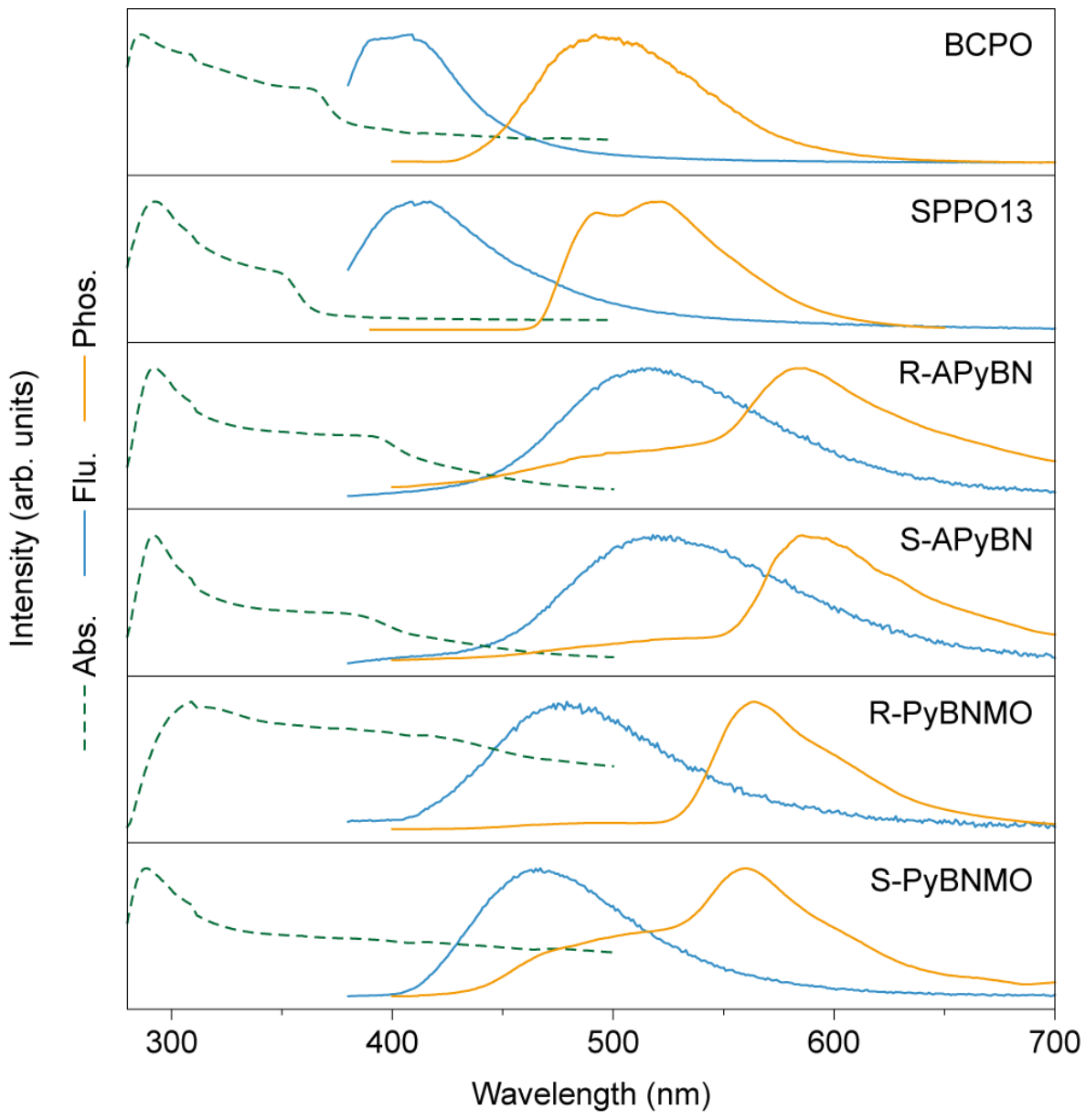

Supplementary Figure 26 | Ultraviolet-visible absorption (Abs., green curves), fluorescence (Flu., blue curves), and phosphorescence (Phos., orange curves, λ_{ex} = 365 nm, delayed time 8 ms, at 77 K) spectra of BCPO, SPPO13, R/S-APyBN and R/S-PyBNMO in melt-casting films.

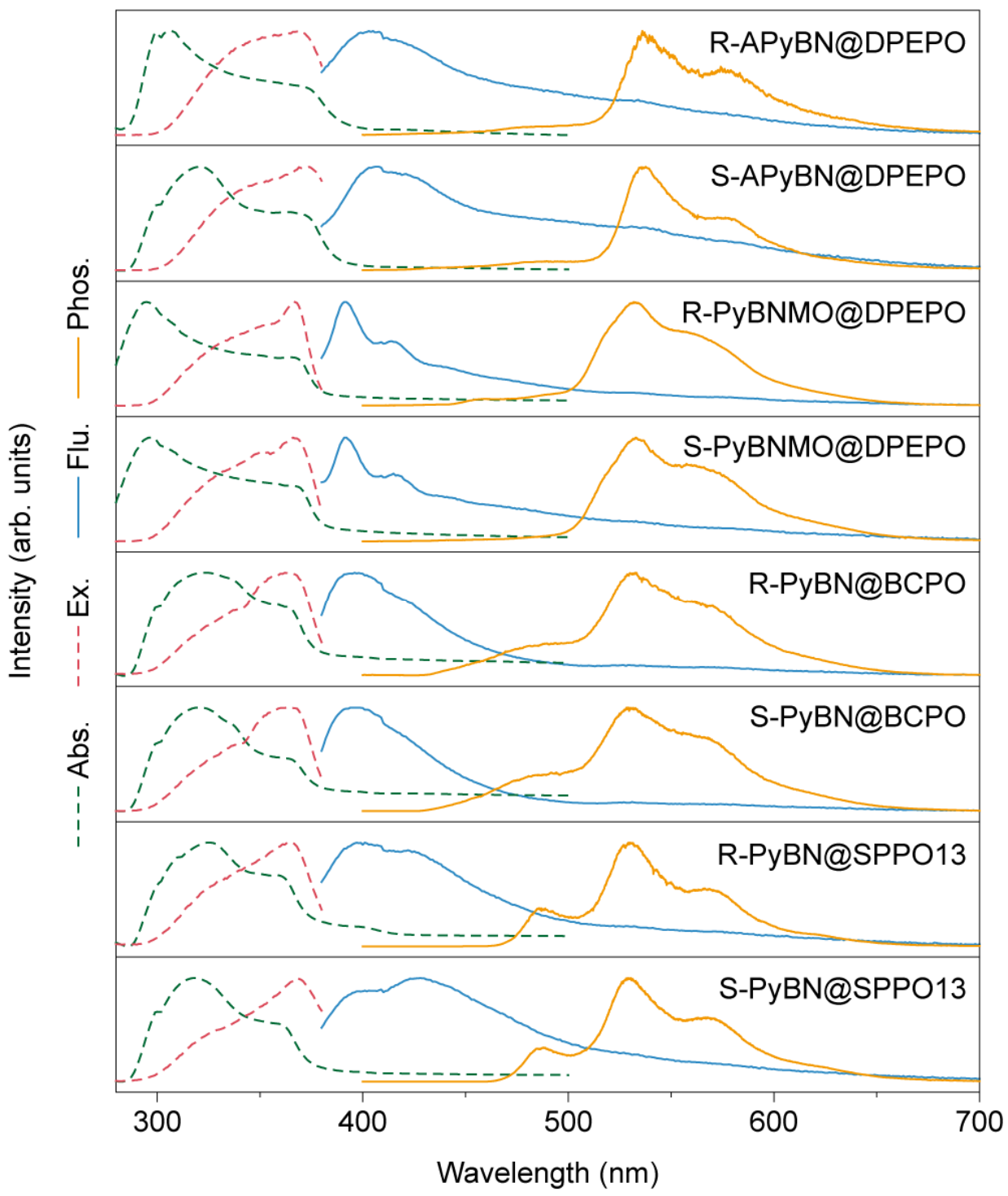

Supplementary Figure 27 | Ultraviolet-visible absorption (Abs., green curves), excitation (Ex., red curves), fluorescence (Flu., blue curves), and phosphorescence (Phos., orange curves, λ_{ex} = 365 nm, delayed time 8 ms, at 77 K) spectra of 1 wt% R/S-APyBN@DPEPO, R/S-PyBNMO@DPEPO, R/S-PyBN@BCPO, and R/S-PyBN@SPPO13 in melt-casting films.

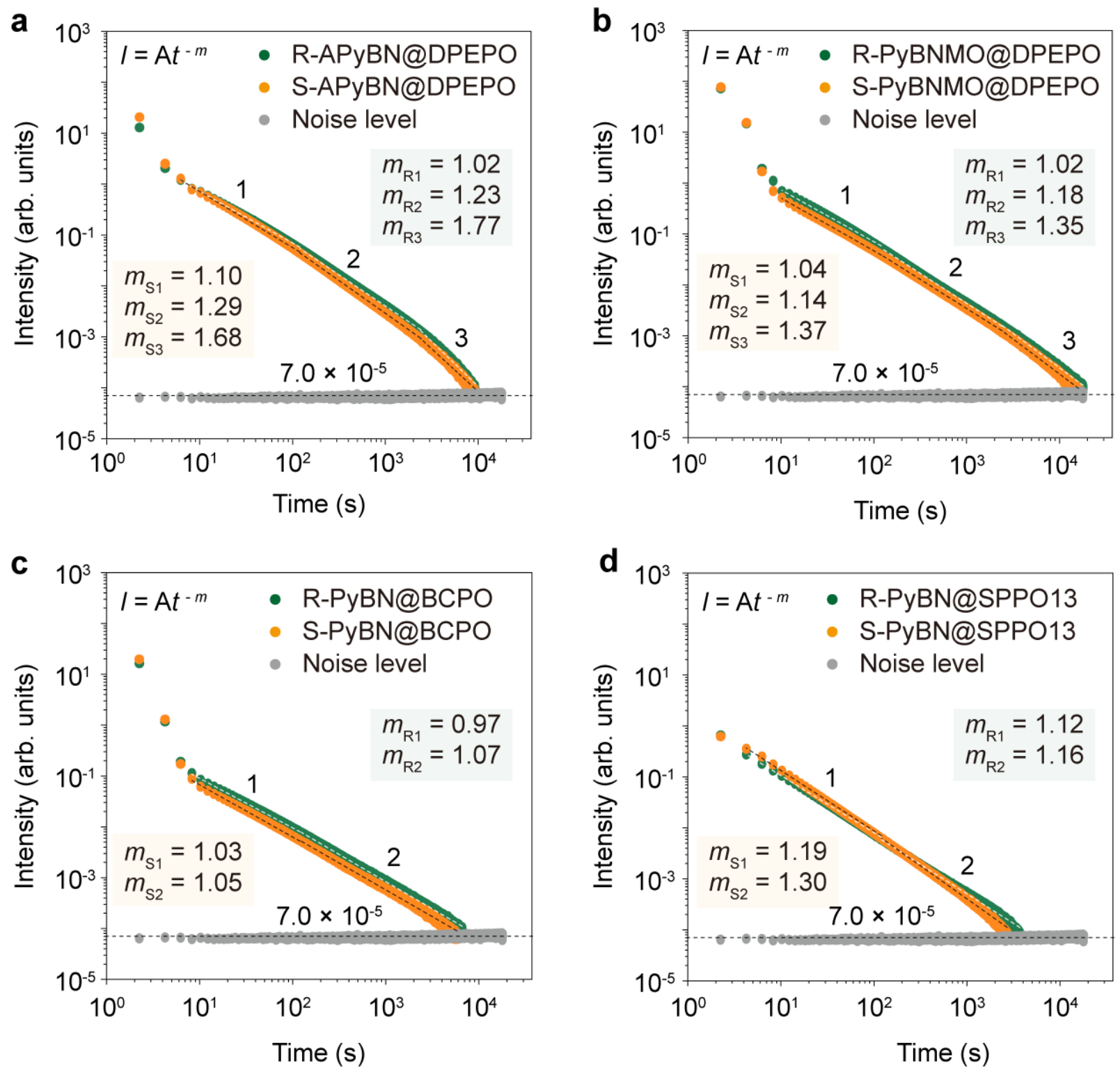

Supplementary Figure 28 | Log-log plot of PersL decay curves of R/S-APyBN@DPEPO (a), R/S-PyBNMO@DPEPO (b), R/S-PyBN@BCPO (c) and R/S-PyBN@SPPO13 (d) under 365 nm excitation. The excitation temperature and recoding temperature were RT. The excitation time was 60 s.

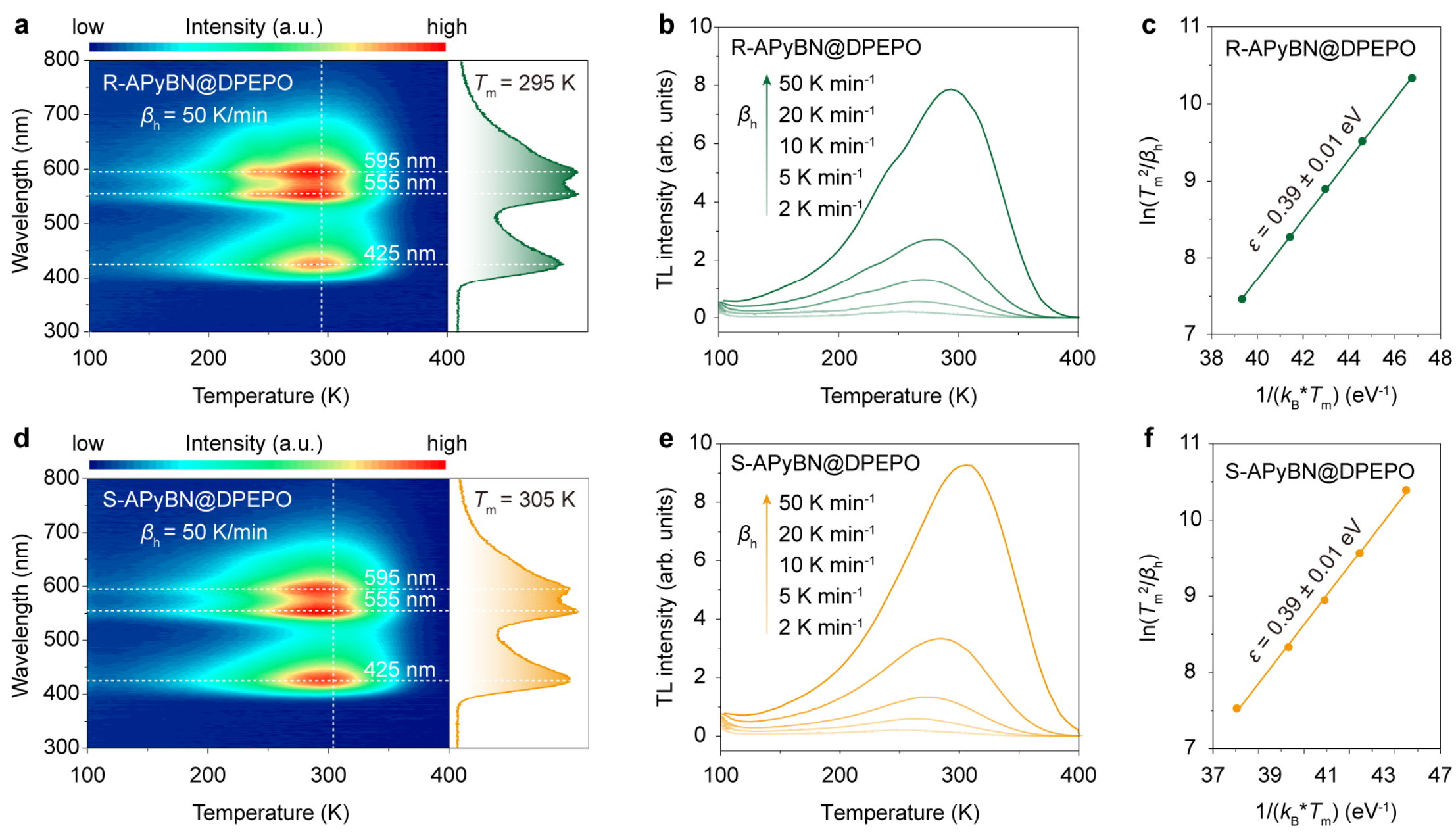

Supplementary Figure 29 | TL characterization of R/S-APyBN@DPEPO. a, d, Intensity-wavelength-temperature TL glow graph (3D-plot) with a heating rate of 50 K min^{-1} PersL emission spectra (right part) are obtained from the 3D-plot. b, e, TL spectra with different heating rates (2, 5, 10, 20, and 50 K min^{-1}) from bottom to top (λ_{ex} = 365 nm, excitation time = 300 s, excitation temperature = 100 K). c, f, Estimation of the trap depth by using the Randall-Wilkins model.

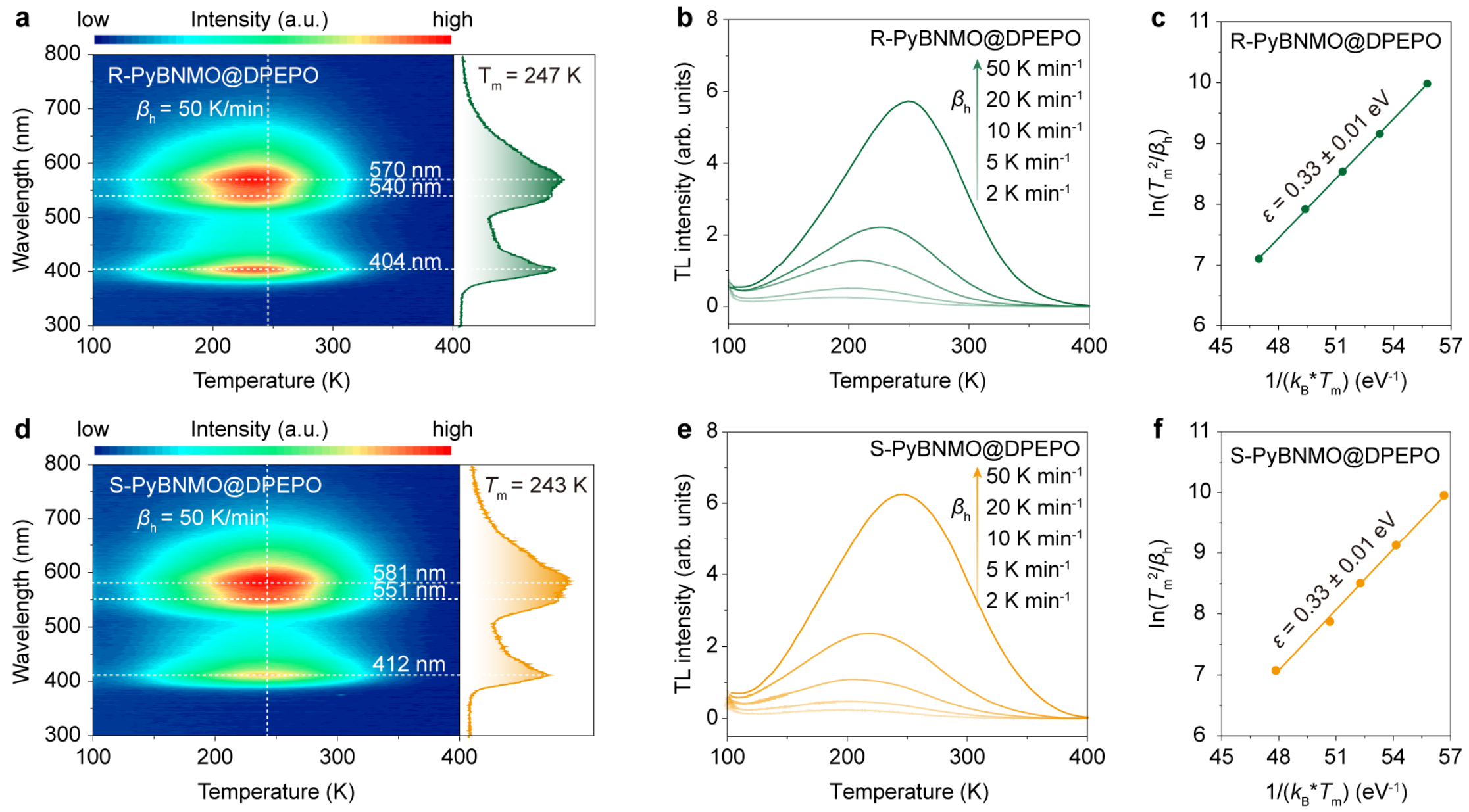

Supplementary Figure 30 | TL characterization of R/S-PyBNMO@DPEPO. a, d, Intensity-wavelength-temperature TL glow graph (3D-plot) with a heating rate of 50 K min^{-1}. PersL emission spectra (right part) are obtained from the 3D-plot. b, e, TL spectra with different heating rates (2, 5, 10, 20, and 50 K min^{-1}) from bottom to top (λ_{ex} = 365 nm, excitation time = 300 s, excitation temperature = 100 K). c, f, Estimation of the trap depth by using the Randall-Wilkins model.

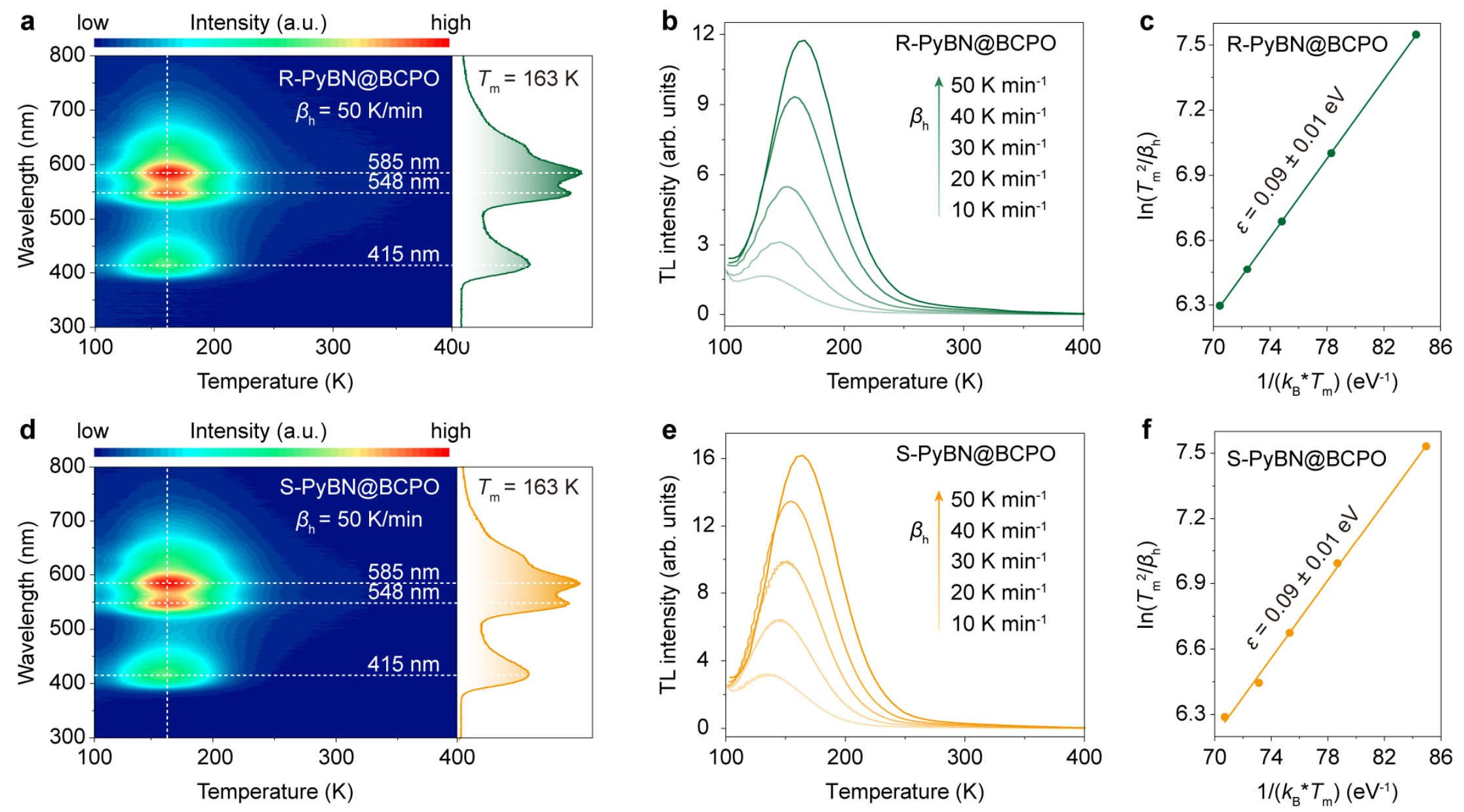

Supplementary Figure 31 | TL characterization of R/S-PyBN@BCPO. a, d, Intensity-wavelength-temperature TL glow graph (3D-plot) with a heating rate of 50 K min^{-1}. PersL emission spectra (right part) are obtained from the 3D-plot. b, e, TL spectra with different heating rates (10, 20, 30, 40, and 50 K min^{-1}) from bottom to top (λ_{ex} = 365 nm, excitation time = 300 s, excitation temperature = 100 K). c, f, Estimation of the trap depth by using the Randall-Wilkins model.

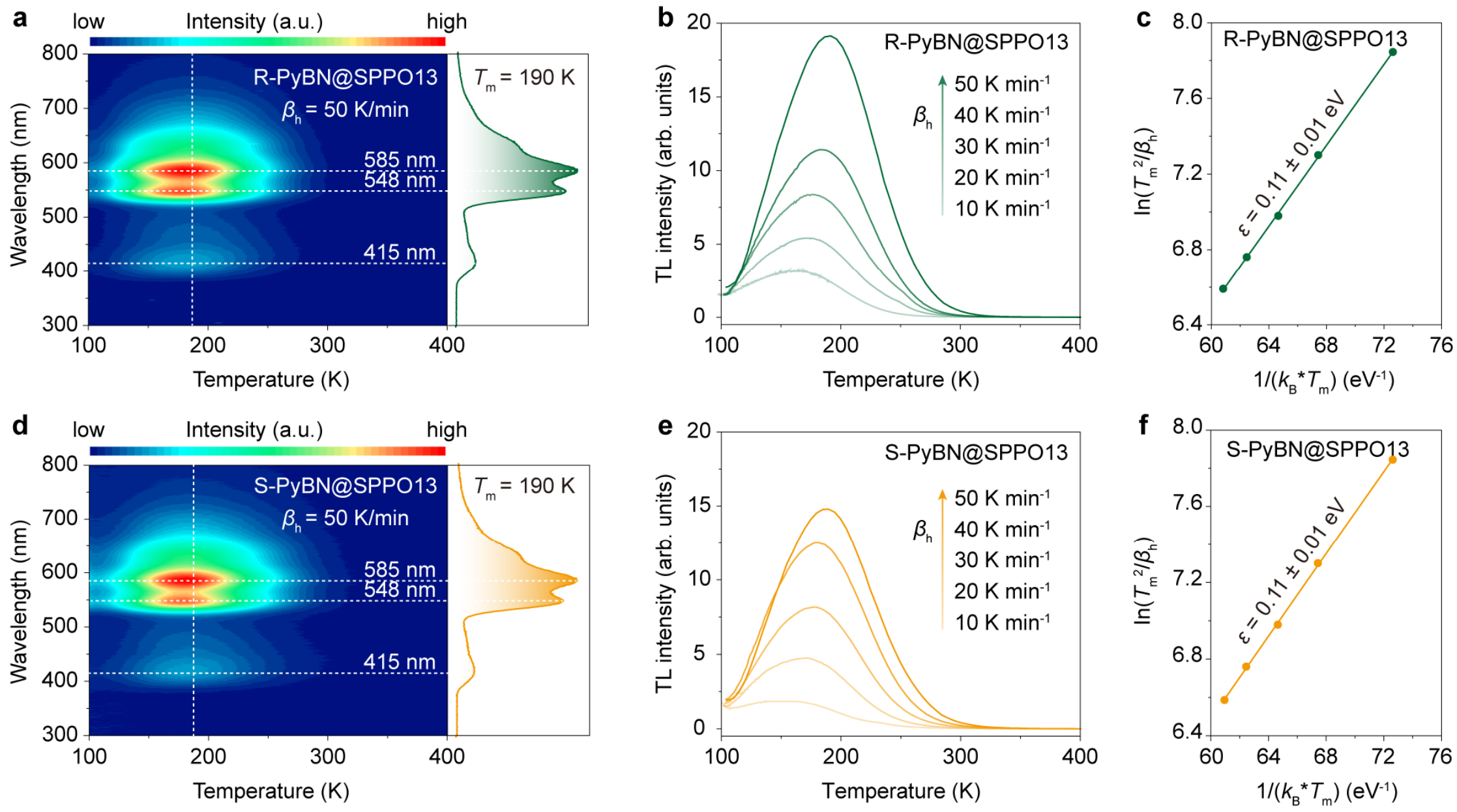

Supplementary Figure 32 | TL characterization of 1 wt% R/S-PyBN@SPPO13. a, d, Intensity-wavelength-temperature TL glow graph (3D-plot) with a heating rate of 50 K min^{-1}. PersL emission spectra (right part) are obtained from the 3D-plot. b, e, TL spectra with different heating rates (10, 20, 30, 40, and 50 K min^{-1}) from bottom to top (λ_{ex} = 365 nm, excitation time = 300 s, excitation temperature = 100 K). c, f, Estimation of the trap depth by using the Randall-Wilkins model.

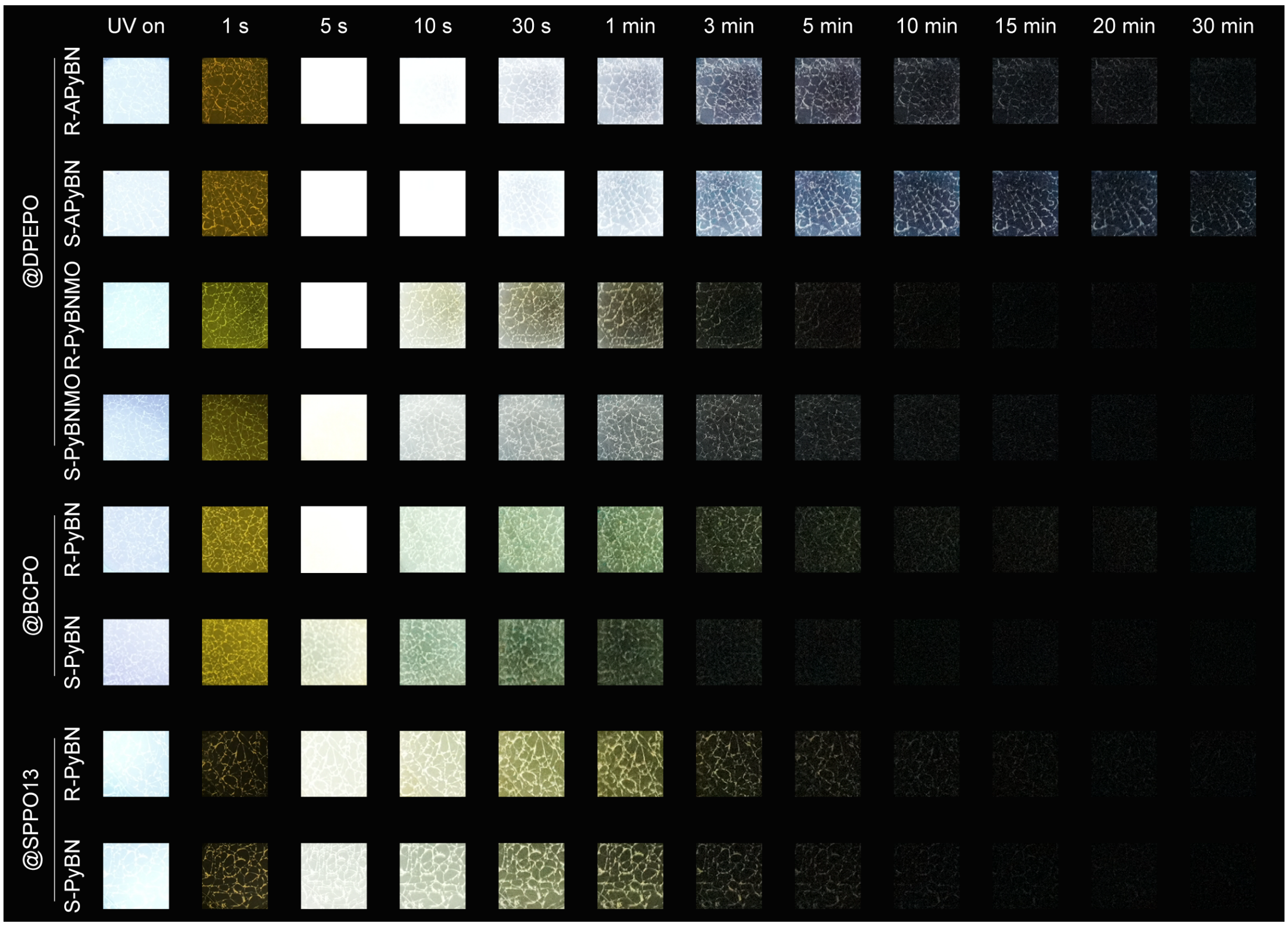

Supplementary Figure 33 | PersL decay photographs of R/S-APyBN@DPEPO, and R/S-PyBNMO@DPEPO, R/S-PyBN@BCPO and R/S-PyBN@SPPO13 films at RT under 365 nm UV lamp excitation (UV on), and at various time after

the excitation off (different time labels). Camera exposure settings: 1) UV on and 1 s: Shutter 1/1000 s, ISO 400; 2) 5 s-30 min: Shutter 2.5 s, ISO 40000.

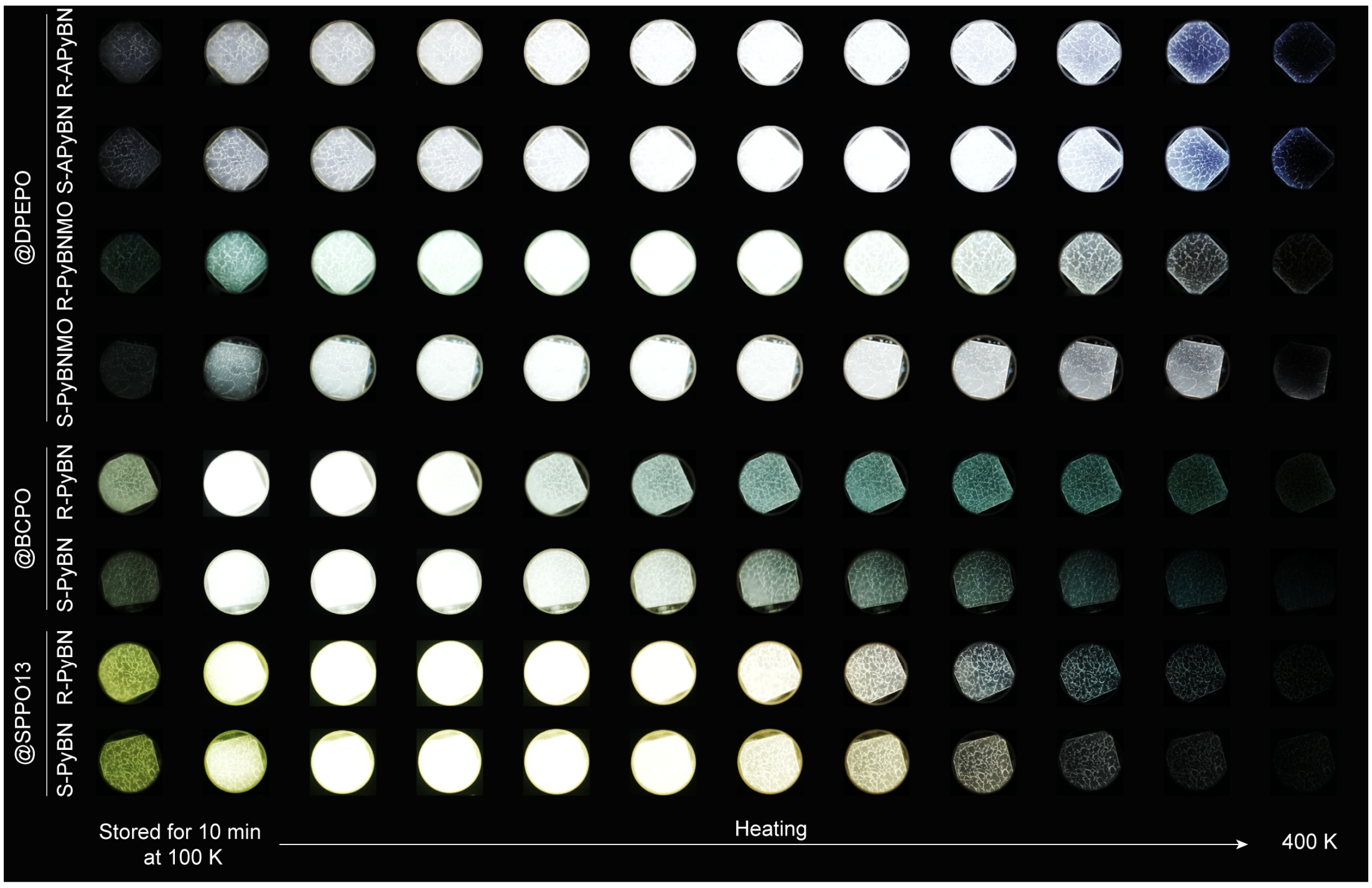

Supplementary Figure 34 | TL photographs of R/S-APyBN@DPEPO, R/S-PyBNMO@DPEPO, R/S-PyBN@BCPO, and R/S-PyBN@SPPO13 films stored at 100 K for 10 min in dark after 365 nm excitation off, and heated from 100 K to 400 K. The emission colour may deviate from the original colour due to the intensity saturation of camera. Camera exposure settings: Shutter 1 s, ISO 8000.

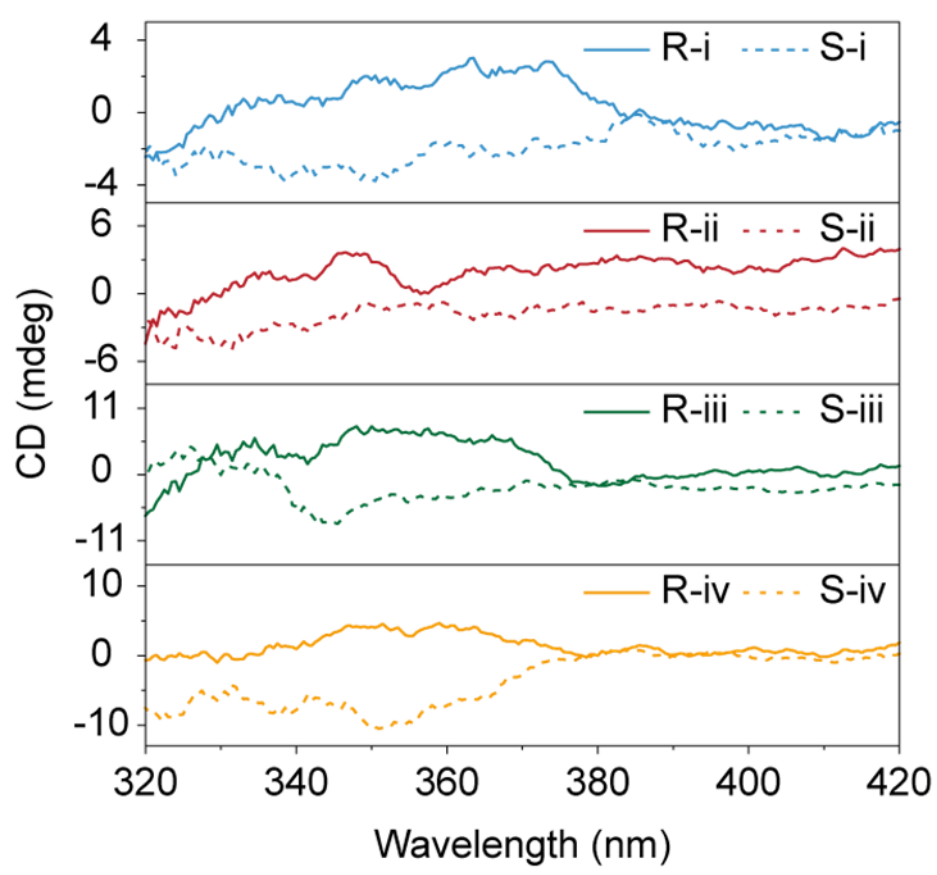

Supplementary Figure 35 | CD spectra of R/S-APyBN@DPEPO (R-i/S-i), R/S-PyBNMO@DPEPO (R-ii/S-ii), R/S-PyBN@BCPO (R-iii/S-iii), and R/S-PyBN@SPPO13 (R-iv/S-iv) melt-casting films at RT.

Supplementary Table 4 | Summaries of CP-PersL performances in various guest-host systems.

Guest	Host	Flu. (nm)[a]	Phos. (nm)[b]	TL (nm)[c]	QY (%)[d]	PersL duration (h)[e]
**R-PyBN**	**DPEPO**	393	531, 572	398, 536, 577	38.4	3.87
**S-PyBN**		393	531, 572	398, 536, 577	39.8	4.20
**R-APyBN**		407	537, 577	425, 555, 595	19.0	2.58
**S-APyBN**		407	537, 577	425, 555, 595	20.3	2.58
**R-PyBNMO**		392	533, 563	404, 540, 570	33.8	4.78
**S-PyBNMO**		392	533, 563	412, 551, 581	42.6	4.29
**R-PyBN**	**BCPO**	397	531, 570	415, 548, 585	31.9	1.55
**S-PyBN**		397	531, 570	415, 548, 585	35.2	1.48
**R-PyBN**	**SPPO13**	397	530, 571	409, 548, 585	21.1	1.03
**S-PyBN**		397	530, 571	409, 548, 585	20.1	0.75

[a]Fluorescence maximum at RT under 365 nm irradiation.

[b]Phosphorescence maximum at 77 K (delayed time 8 ms).

[c]Emission maximum under TL measurements.

[d]The luminescence quantum yield (QY) at RT under 365 nm irradiation.

[e]PMT monitored PersL duration after ceasing UV lamp.

Supplementary Table 5 | Summaries of trap depth properties in various guest-host systems. The doping concentration of the guest was fixed as 1 wt%.

Guest	Host	$\varepsilon_{cal.}$ (eV)[a]				$\Delta\varepsilon_{cal.}$ (eV)[b]	ε_{MV} (eV)[c]	$\Delta\varepsilon$ (eV)[d]
		α_H - β_G	α_H - α_G	β_H - α_G	β_H - β_G			
**R-PyBN**	**DPEPO**	0.14	0.42	0.50	0.22	0.32	0.32±0.01	0±0.01
**S-PyBN**		0.13	0.43	0.51	0.21	0.32	0.32±0.01	0±0.01
**R-APyBN**		0.28	0.41	0.49	0.36	0.385	0.39±0.01	0.005±0.01
**S-APyBN**		0.23	0.46	0.54	0.31	0.385	0.39±0.01	0.005±0.01
**R-PyBNMO**		0.17	0.41	0.49	0.25	0.33	0.33±0.01	0±0.01
**S-PyBNMO**		0.14	0.43	0.51	0.22	0.325	0.33±0.01	0.005±0.01
**R-PyBN**	**BCPO**	-0.05	0.23	0.23	-0.05	0.09	0.09±0.01	0±0.01
**S-PyBN**		-0.06	0.24	0.24	-0.06	0.09	0.09±0.01	0±0.01
**R-PyBN**	**SPPO13**	-0.04	0.24	0.26	-0.02	0.11	0.11±0.01	0±0.01
**S-PyBN**		-0.05	0.25	0.27	-0.03	0.11	0.11±0.01	0±0.01

[a]Energy differences calculated from the values between LUMO energy of host radical anion and that of guest radical anion at B3LYP/def2-SVP level of theory.

[b]Average value of calculated energy differences.

[c]Trap depth calculated by plotting $\ln(T_m^2/\beta_h)$ against $1/(k_B \cdot T_m)$ under TL measurement *via* the Randall-Wilkins model.

[d]Differences between $\varepsilon_{cal.}$ and ε_{MV}.

VII. Demonstration of Applications

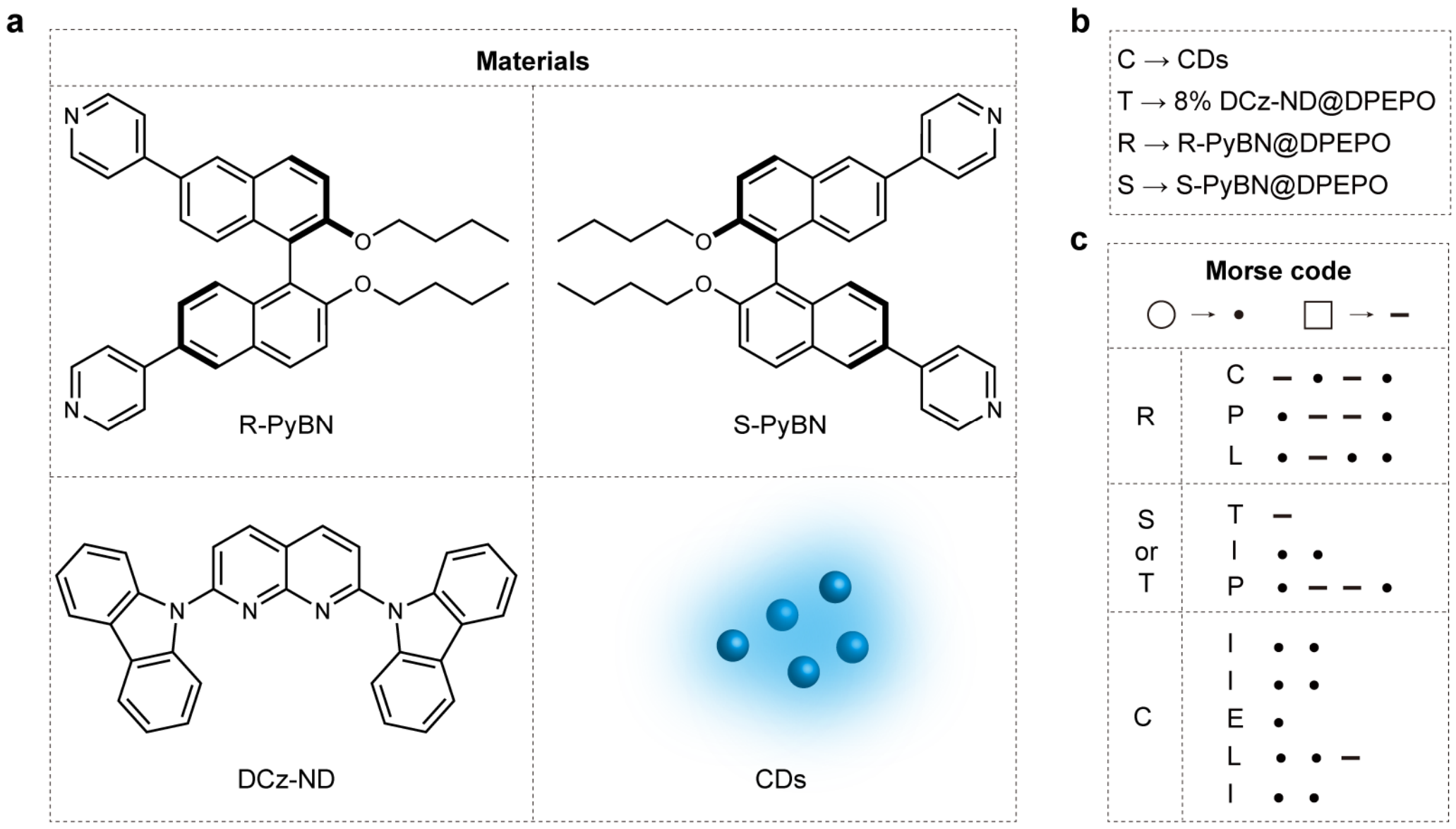

Supplementary Figure 36 | Chemical structures (a), abbreviations (b), and corresponding morse codes (c) of the materials used in the application.

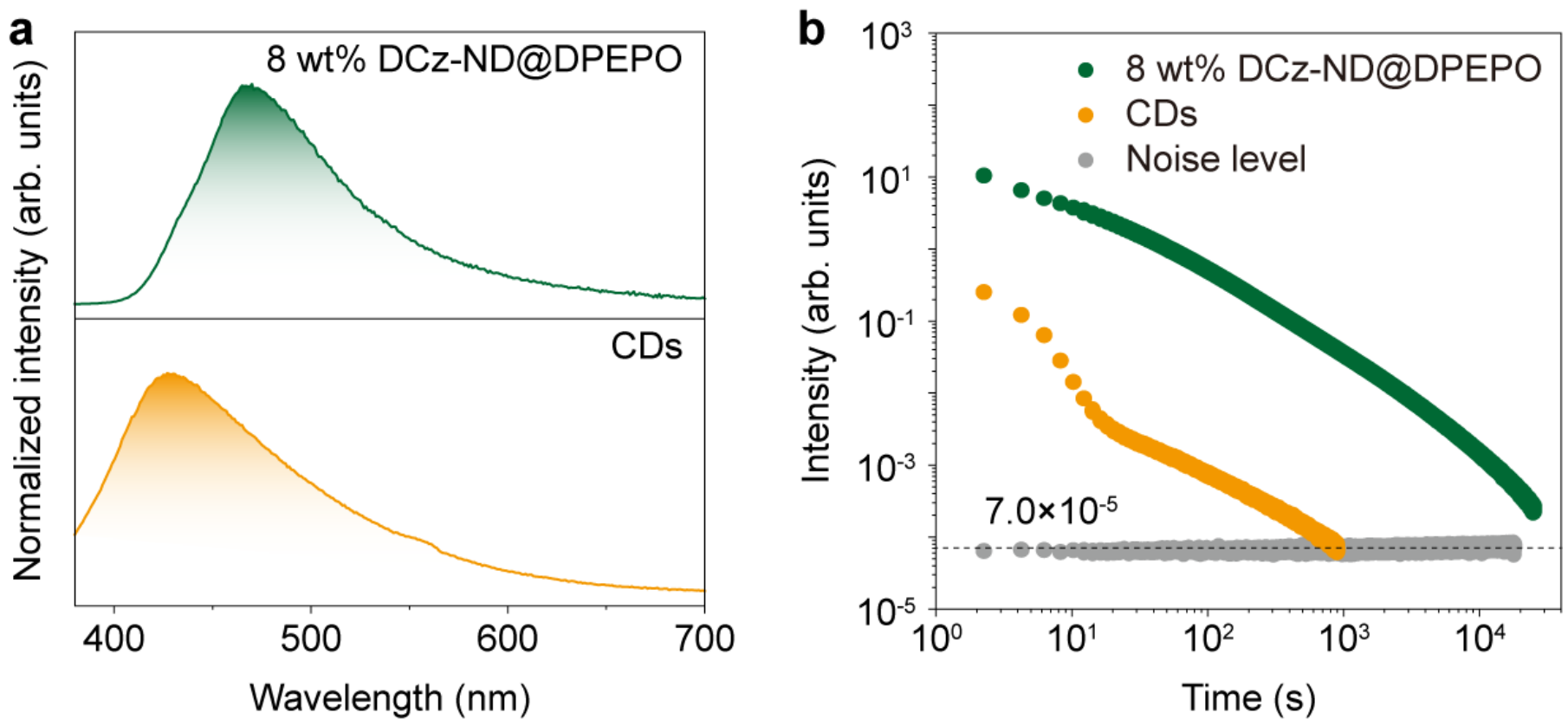

Supplementary Figure 37 | Fluorescence spectra and PersL decay curves of DCz-ND@DPEPO films. a, Fluorescence spectra of 8 wt% DCz-ND@DPEPO film (green curve) and CDs (orange curve) under 365 nm excitation. b, Log–log plots of PersL decay curves for the blend films measured at room temperature following excitation at RT for 60 s.

VIII. Background Information

Supplementary Table 6 | Representative white PersL and CP-PersL materials with corresponding performance.[a]

<table>
<tr><th colspan="2">Molecular formula</th><th>Excitation source (nm)</th><th>Emission (nm)</th><th>|g_{lum}| ($\times 10^{-3}$)</th><th>Lifetime (ms-h)[b] or decay time (s-h)[c]</th><th>Reference</th></tr>
<tr><td>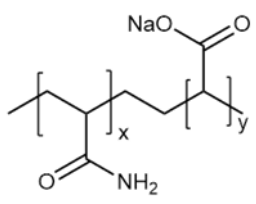
Host-1: APAM</td><td>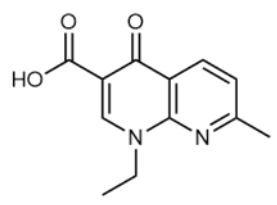
Guest-1: NDA</td><td rowspan="2">365</td><td rowspan="2">457, 567, 675</td><td rowspan="2">-</td><td rowspan="2">98.36 ms[a]</td><td rowspan="2">4</td></tr>
<tr><td>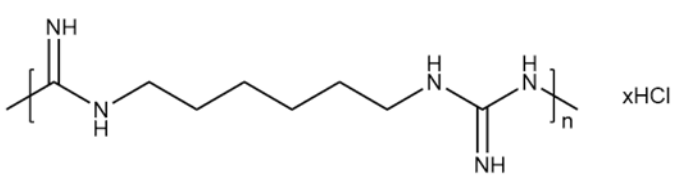
Host-2: PHMB</td><td>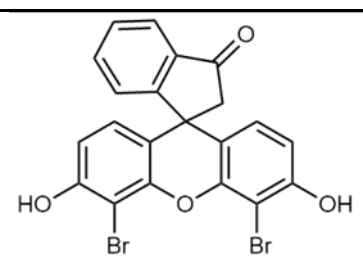
Guest-2: DBF</td></tr>
<tr><td colspan="2">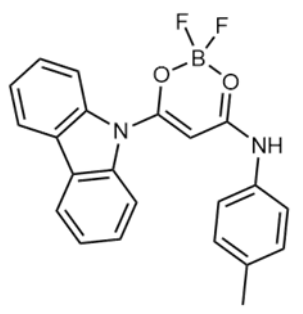
CzBMe</td><td>365</td><td>430, 460, 535</td><td>-</td><td>0.61 s[a]</td><td>5</td></tr>
<tr><td>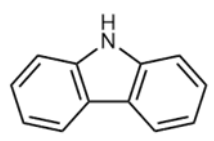
Host: Cz</td><td>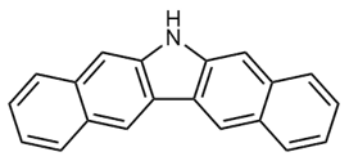
Guest: DBCz</td><td>365</td><td>458, 550 (40/60℃)</td><td>-</td><td>632 ms[a]</td><td>6</td></tr>
<tr><td rowspan="2">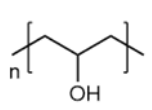
Host: PVA</td><td>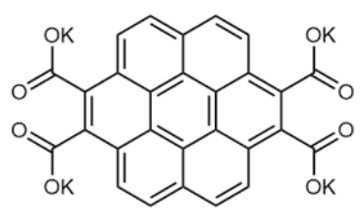
Guest-1: CS</td><td rowspan="2">346</td><td rowspan="2">400-700</td><td rowspan="2">-</td><td rowspan="2">~2.6 s[a]</td><td rowspan="2">7</td></tr>
<tr><td>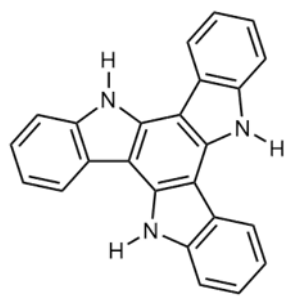
Guest-2: TAT</td></tr>
<tr><td colspan="2">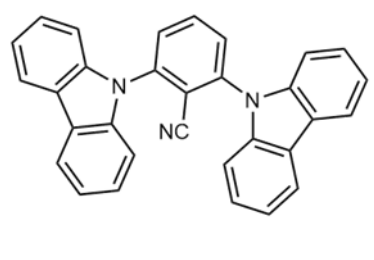
DCBN</td><td>365</td><td>460, 544</td><td>-</td><td>964.3 ms[a]</td><td>8</td></tr>
<tr><td>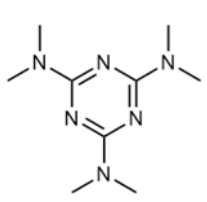
Host: MF</td><td>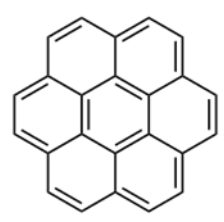
Guest: COR</td><td>365</td><td>448, 568 (370 K)</td><td>-</td><td>4.83 s[a]</td><td>9</td></tr>
</table>

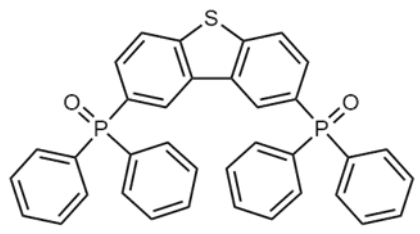<br>Host: PPT	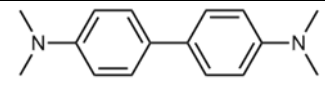<br>Guest-1: TMB<br>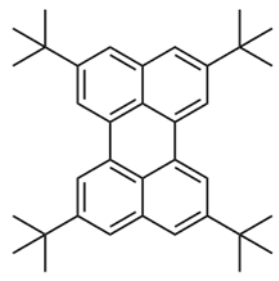<br>Guest-2: TBPe<br>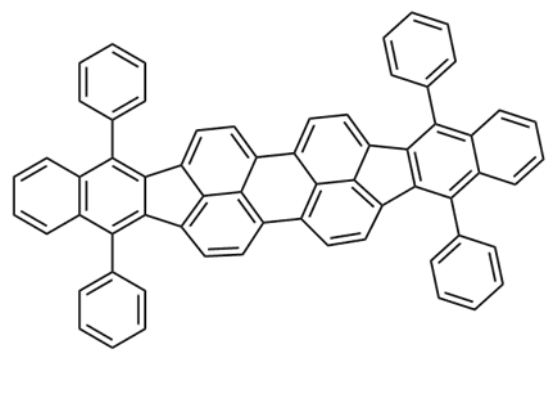<br>Guest-3: DBP	355	522, 497, 665	-	92 s[b]	10
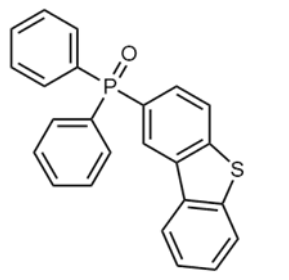<br>Host: DBTSPO	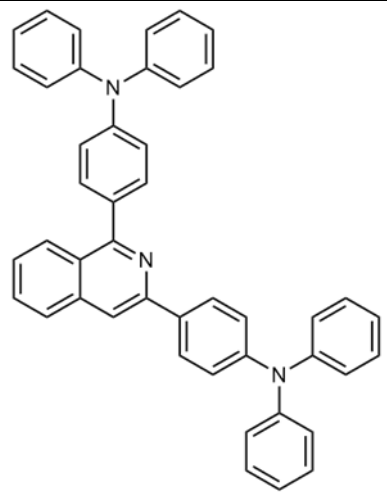<br>Guest: KZLDTPA	410	465, 577	-	112.3 ms[a] / 40 min[b]	11
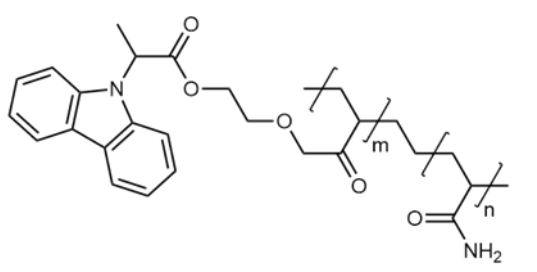<br>Host: R/S-PAMCOOCz$_x$	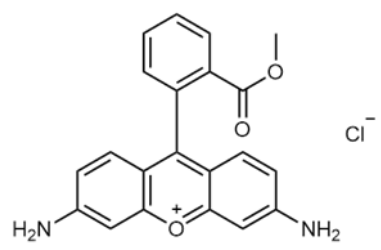<br>Guest: Rh123	299	414, 442, 470, 574	5.5	1.9 s[a]	12
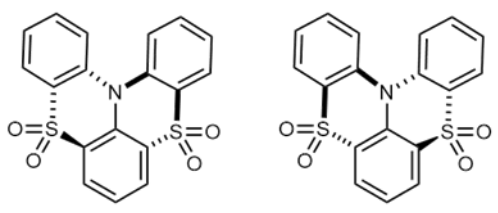<br>(+)-(P)/(-)-(M)-BTPT		-	458	7.8	13.7 ms[a]	13
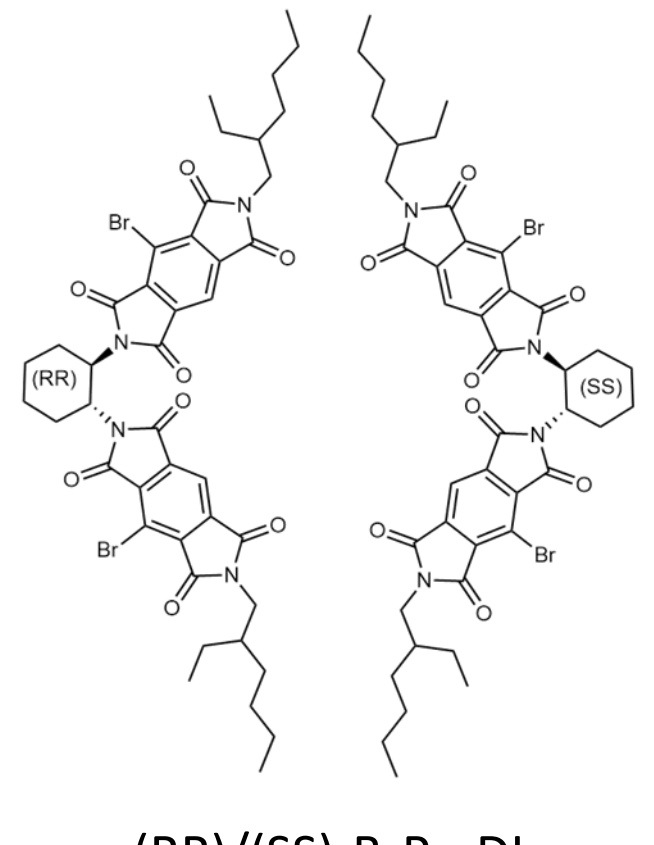<br>(RR)/(SS)-BrPmDI		310	500	1.3	31.5 ms[a]	14
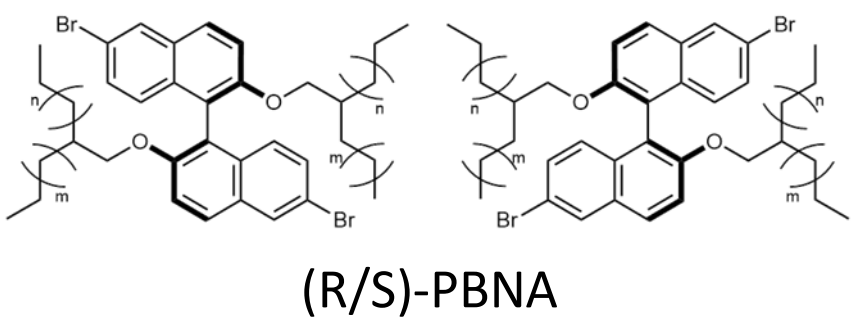<br>(R/S)-PBNA		290	551, 520	9.4	34 ms[a]	15

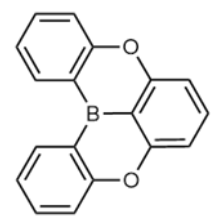<br>Host: DOBA<br>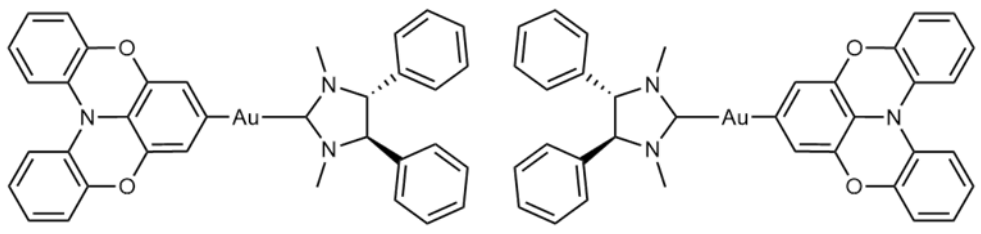<br>Guest: R/S-DPXZAu	340	492	1.4	42 ms[a]	16
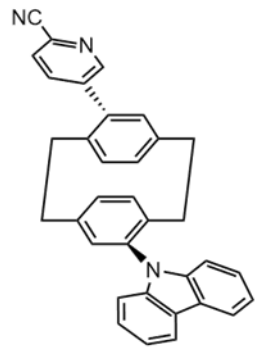<br>(+)-RP/(-)-SP-PCP-PyCNCz	-	550	7.2	528 ms[a]	17
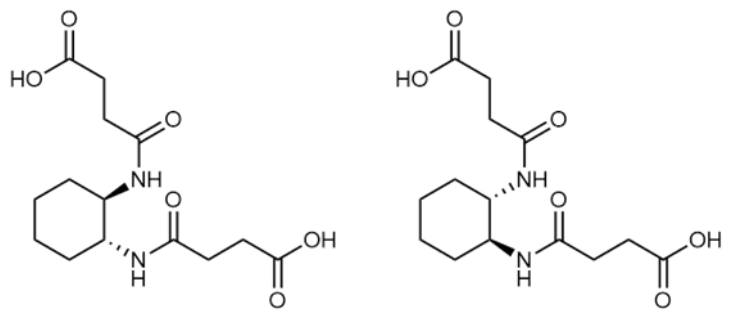<br>(R, R)/(S, S)-DAACH	240-360	470-530	2.3	587 ms[a]	18
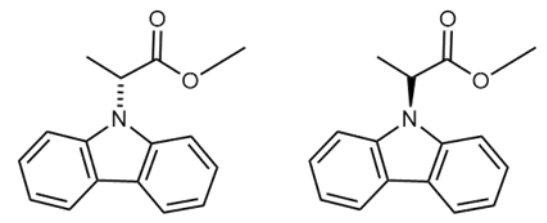<br>R/S-COOCz	365	550, 596	2.3	600 ms[a]	19
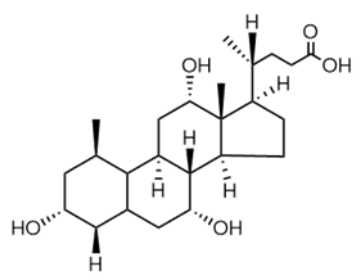<br>Host: CLA<br>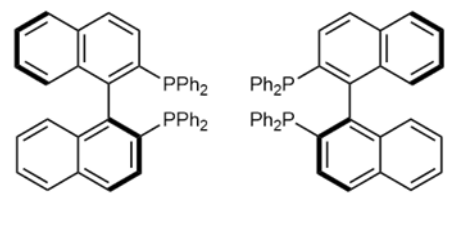<br>Guest-1: R/S-BINAP<br>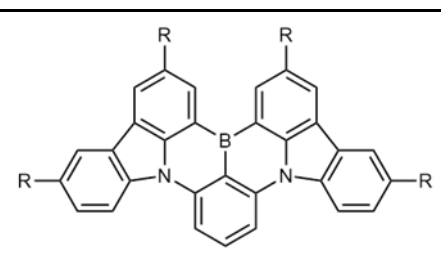<br>Guest-2: BCzBN, R=C(CH)$_3$	300	502	3.5	720 ms[a]	20
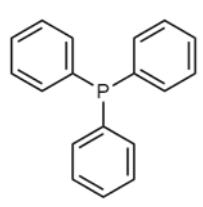<br>Host-1: TPP<br>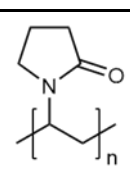<br>Host-2: PVP<br>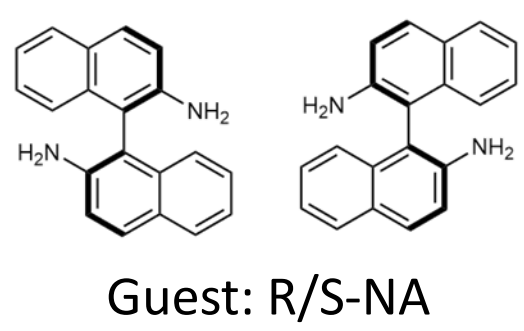<br>Guest: R/S-NA	365	535	0.9	780.5 ms[a]	21
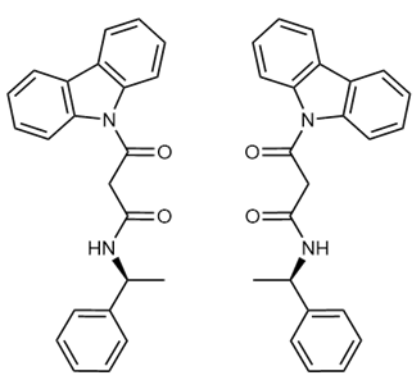<br>R/S-CzCOPEA	300	365, 385, 408, 528, 574, 624	5.6	946.44 ms[a]	22

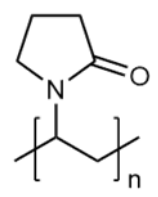<br>Host: PVP	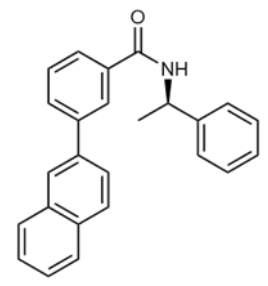<br>Guest-1: Na-R/S	300	514	2.1	601 ms[a]	23
	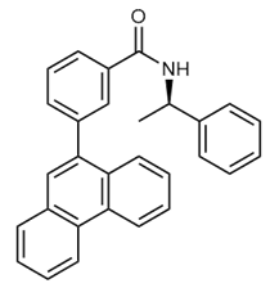<br>Guest-2: Pa-R/S	360	533	2.2	1017 ms[a]	
	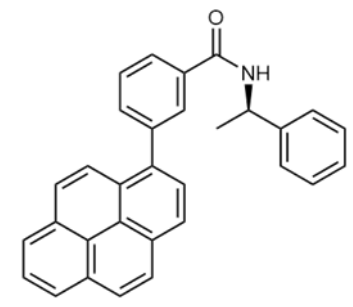<br>Guest-3: Py-R/S	380	612	2.5	341 ms[a]	
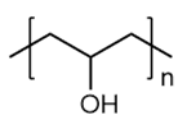<br>Host: PVA	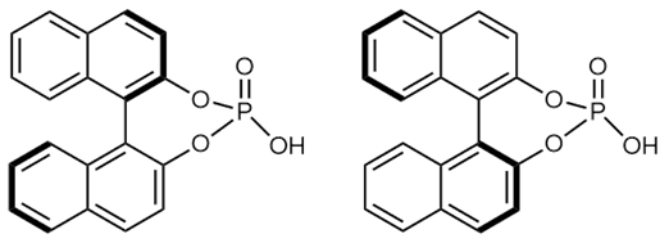<br>Guest: R/S-BNDHP	312	501, 534	6.89	1025.5 ms[a]	24
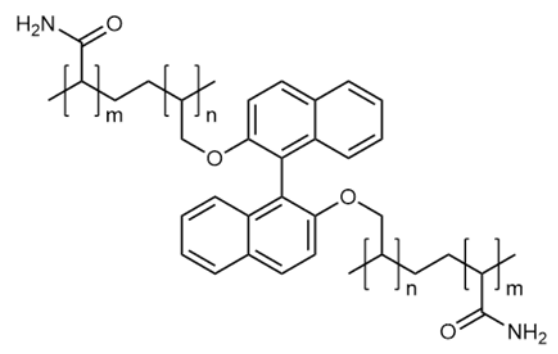<br>R/S-PAMBNAX		254	545	10.6	1078.44 ms[a]	25
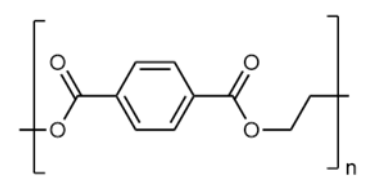<br>Host: PET	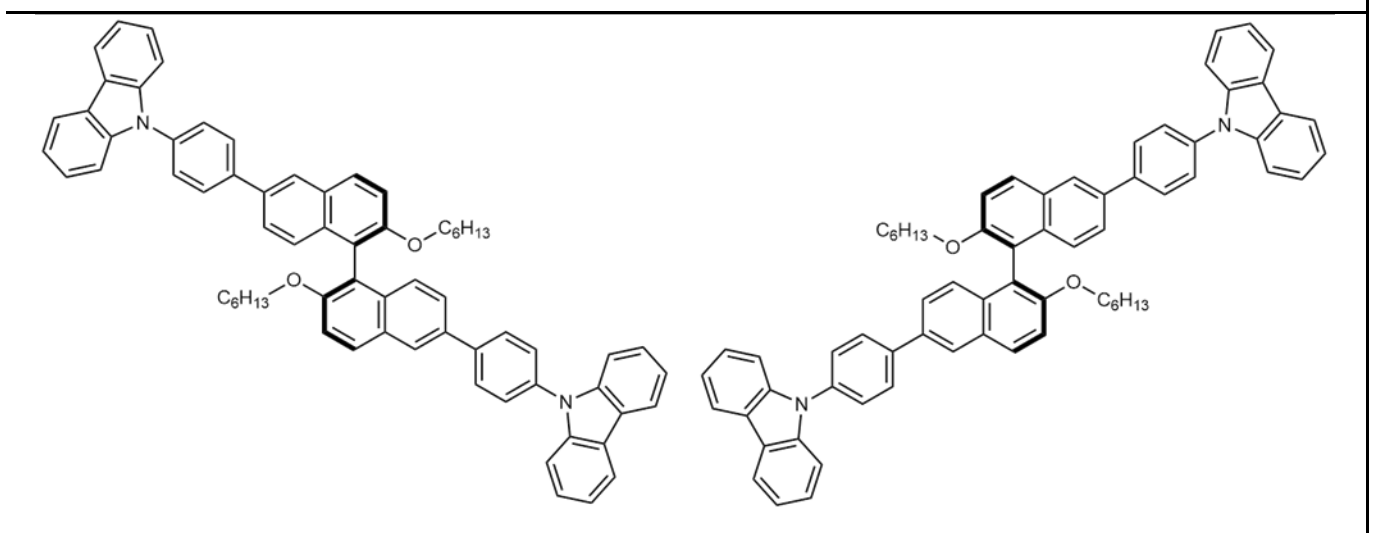<br>Guest: R/S-BINAC	365	580	0.61	2960 ms[a] / > 1 h[b]	26
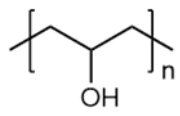<br>Host: PVA	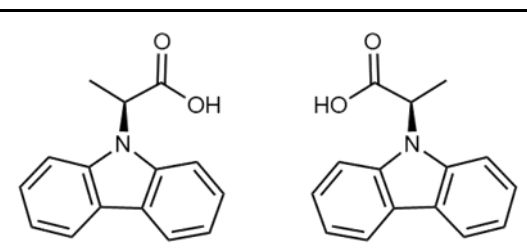<br>Guest: R/S-COOHCz	300	416, 444	50	3.3 s[a]	27
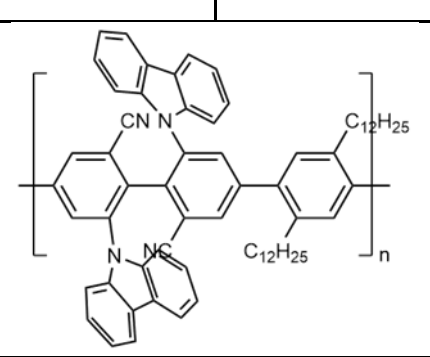		365	489	5.2	33 s[a] (at 77 K)	28

R/S-P12-BCzBCN						
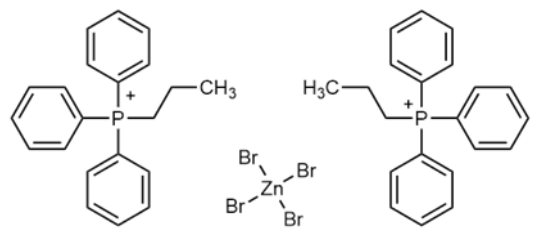<br>Host: $(PTP)_2ZnBr_4$	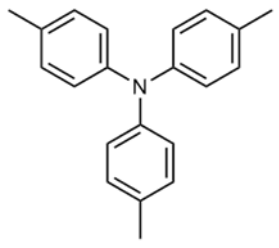<br>Guest-1: R1	395	510	11	> 1 h[b]	29
	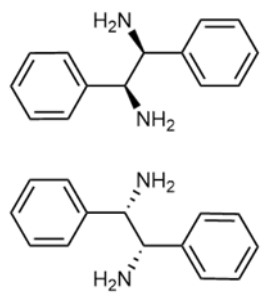<br>Guest-2: (R,R)/(S,S)					
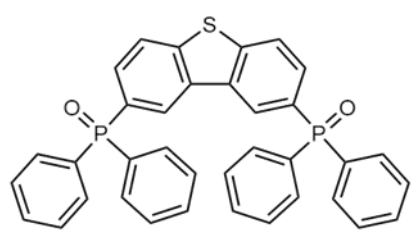<br>Host: PPT	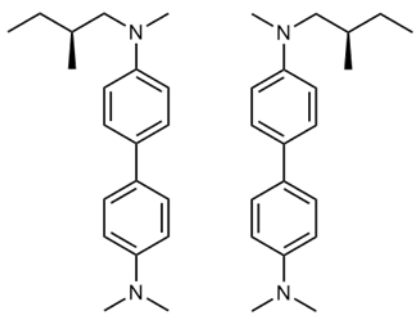<br>Guest-1: R/S-TBTMB	365	525	4.5	> 1.5 h[b]	30
	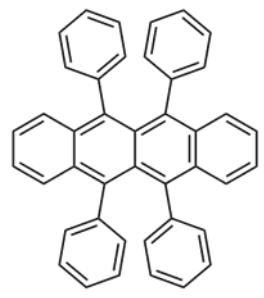<br>Guest-1+Guest-2: Rubrene	365	600	2.3	> 1 h[b]	
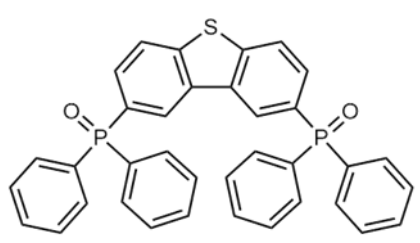<br>Host-1: PPT	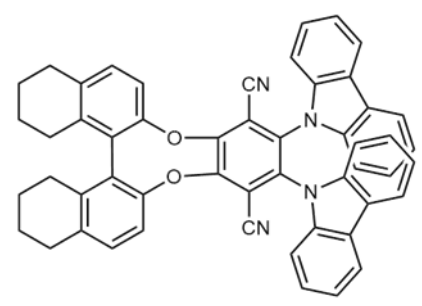<br>Guest-1: R/S-OBN-Cz	365	520	0.28	> 2 h[b]	31
	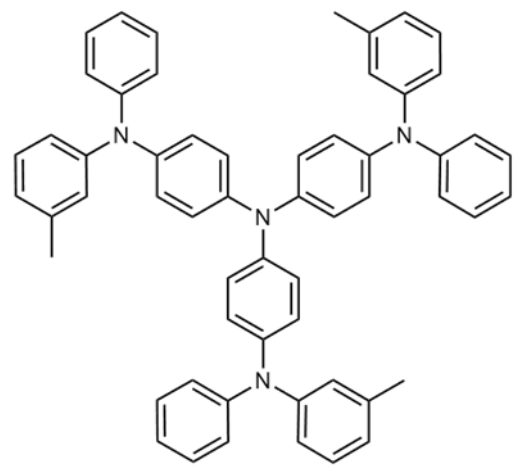<br>Guest-2: m-MTDATA					

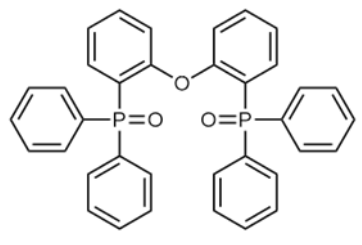<br>Host-1: DPEPO<br>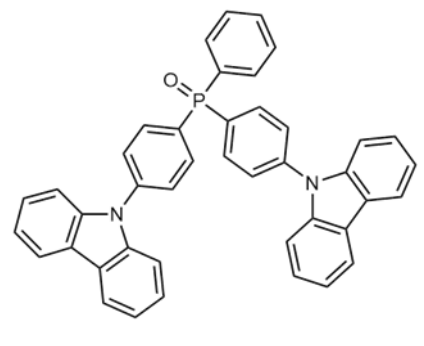<br>Host-2: BCPO<br>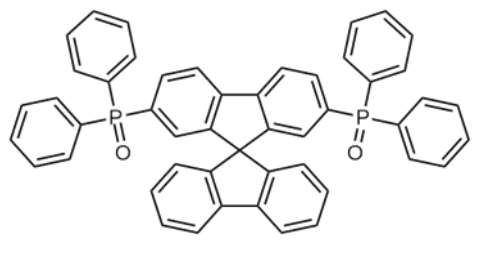<br>Host-3: SPPO13	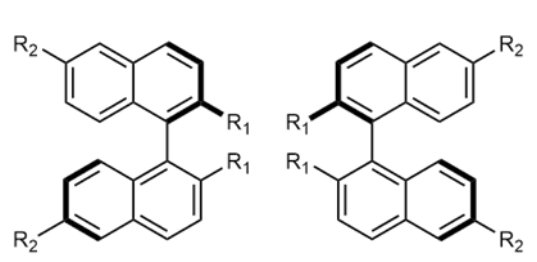<br>Guest:<br>R/S-PyBN (R_1 = C_4H_9O, R_2 = C_5H_5N)<br>R/S-APyBN (R_1 = C_4H_9O, R_2 = C_7H_5N)<br>R/S-PyBNMO (R_1 = $C_2H_5O_2$, R_2 = C_5H_5N)	365	390-700	0.82-2.3	0.75-4.78 h[b] (R/S-PyBN: 762.13 ms[a])	This work

[a]The selected parameters are the best values reported in the references.

[b]Measured by using a time-correlated single photon counting method (lifetime).

[c]Measured by using a photomultiplier tube (decay time).

IX. Nuclear Magnetic Resonance (NMR) Spectra, High-Resolution Mass Spectra (HRMS) and High-Performance Liquid Chromatography (HPLC)

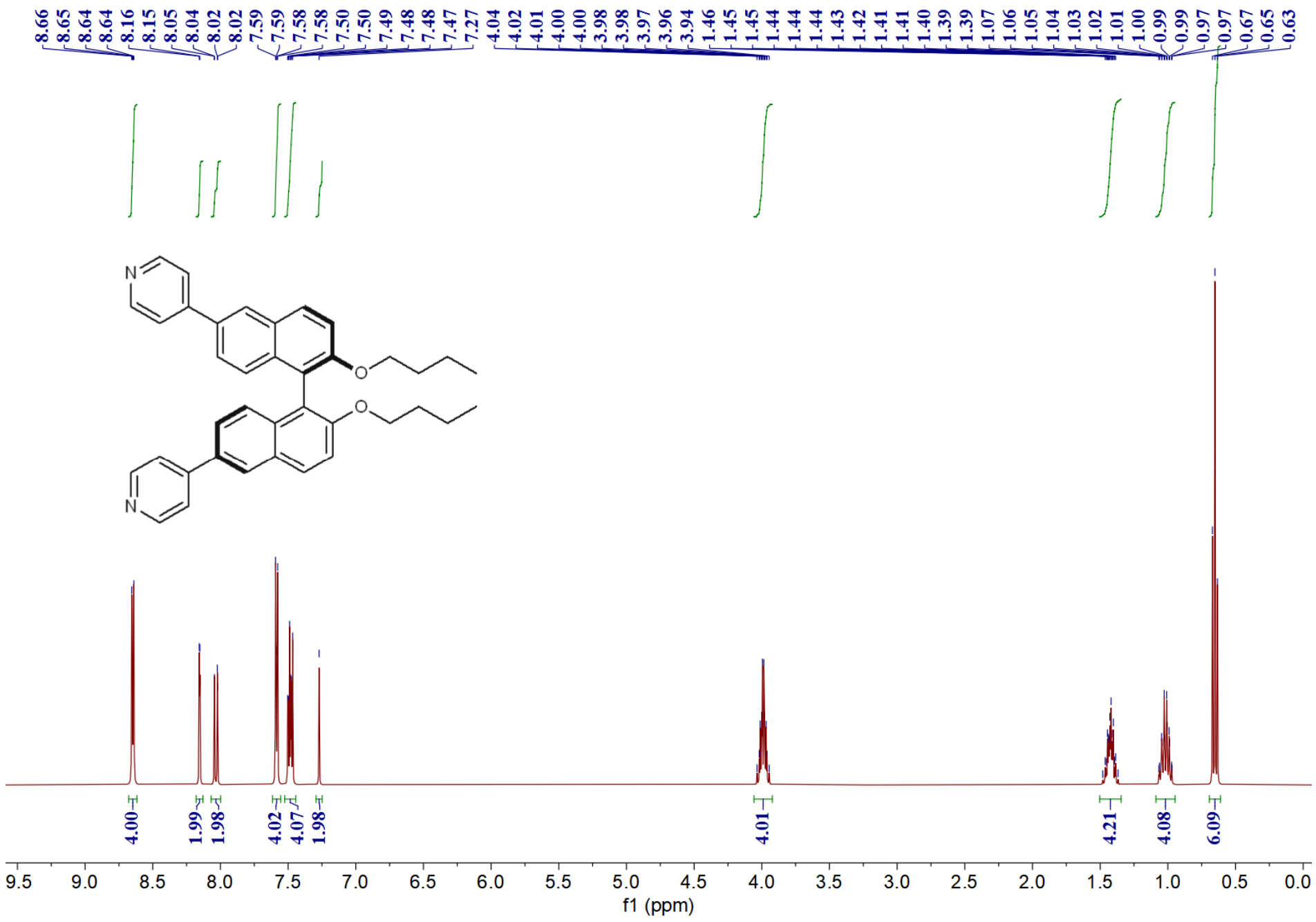

Supplementary Figure 38 | ^{1}H-NMR spectra of R-PyBN.

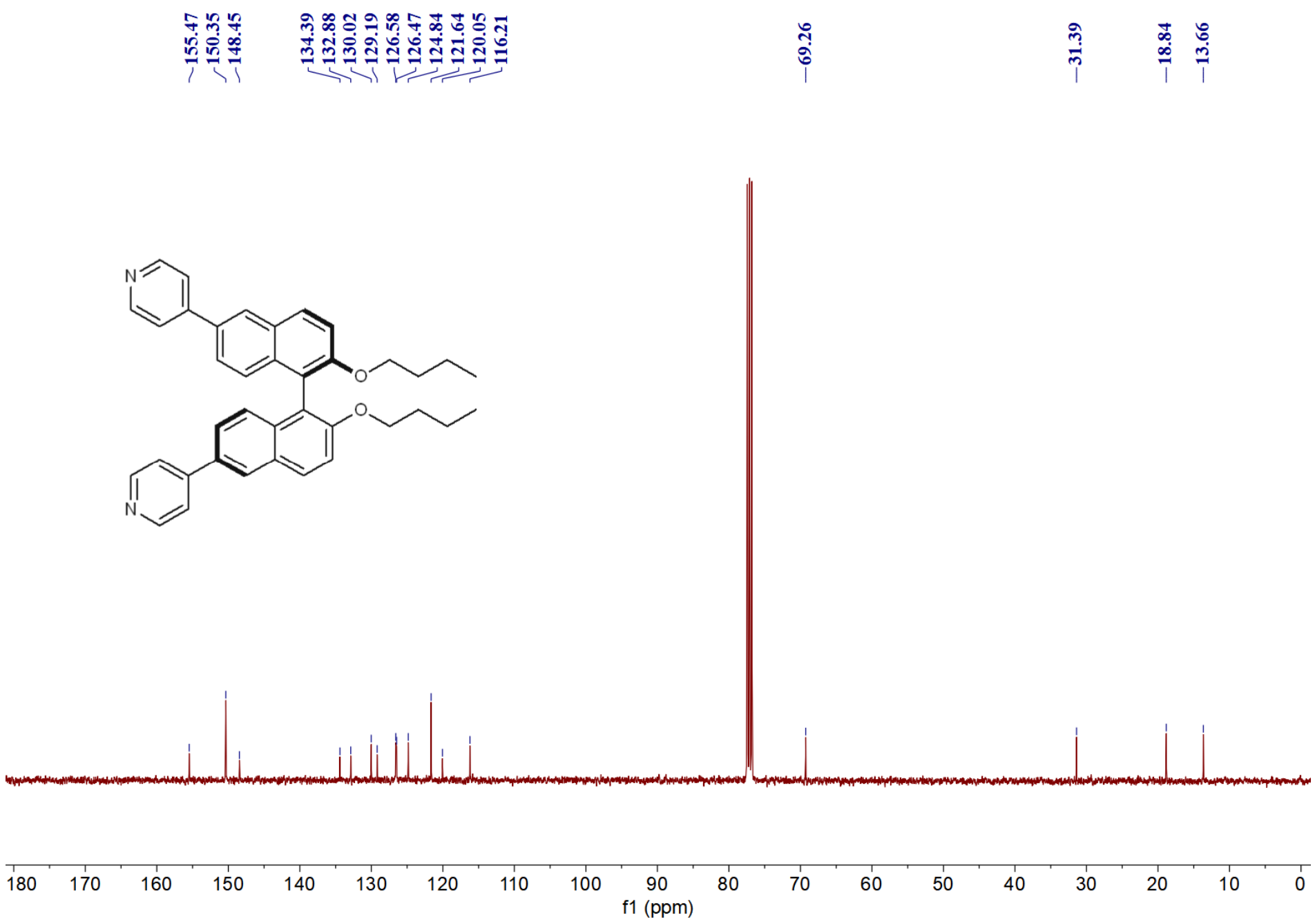

Supplementary Figure 39 | ^{13}C-NMR spectra of R-PyBN.

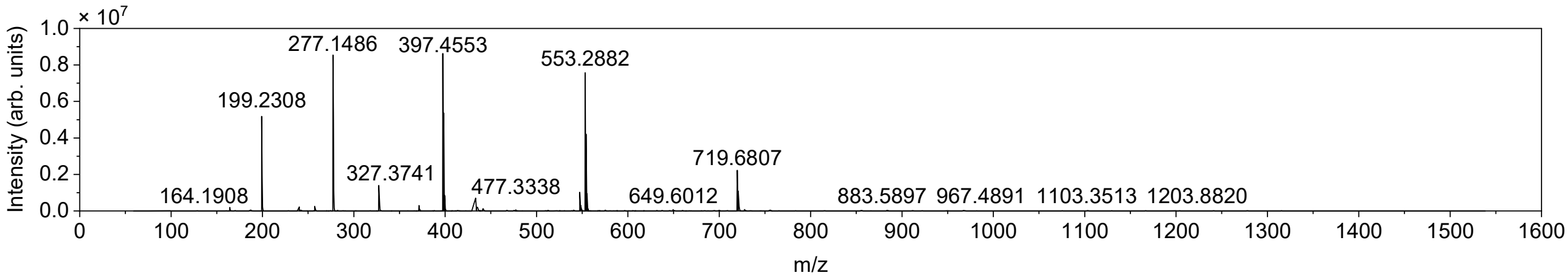

Supplementary Figure 40 | HRMS spectra of R-PyBN.

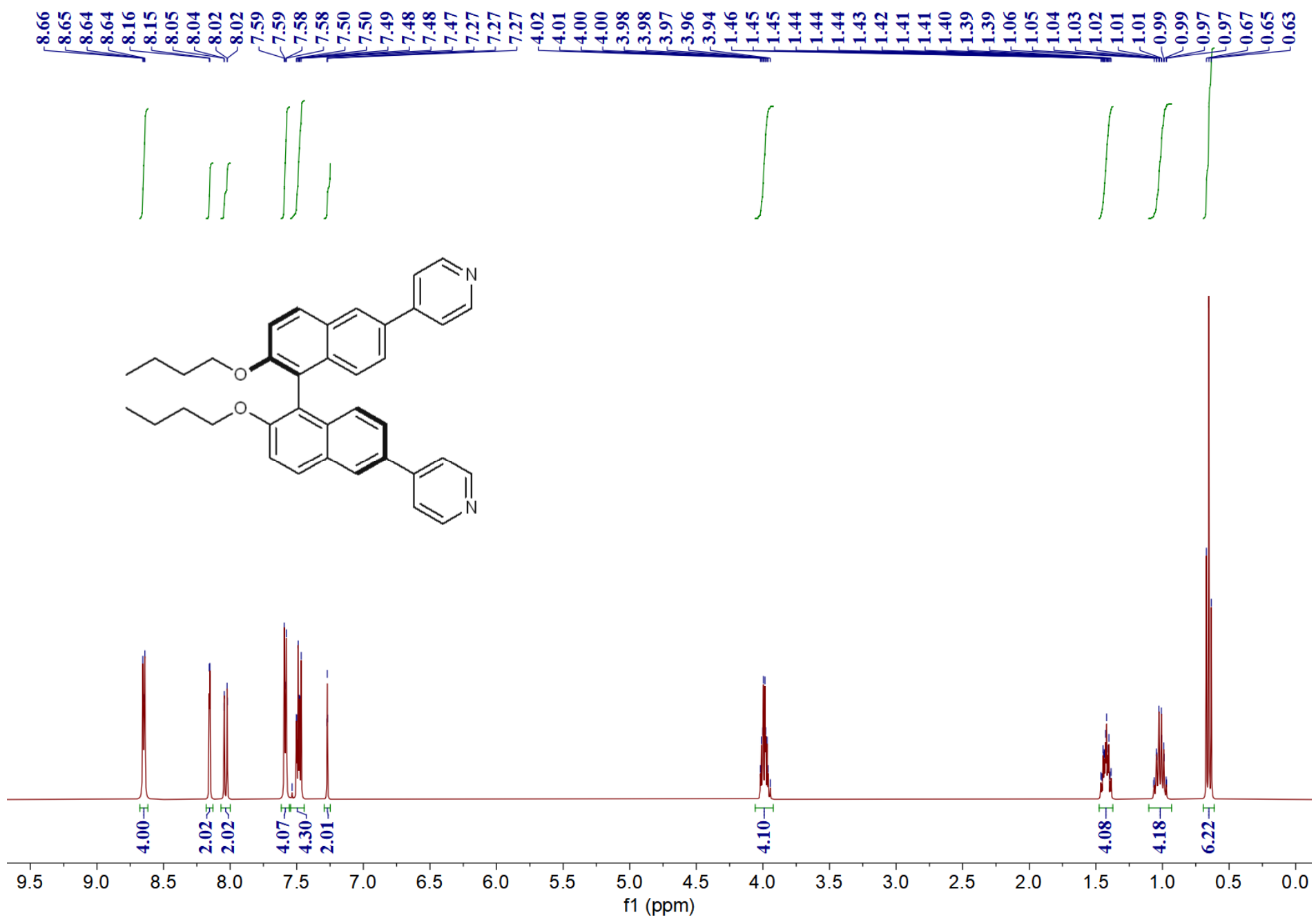

Supplementary Figure 41 | ^{1}H-NMR spectra of S-PyBN.

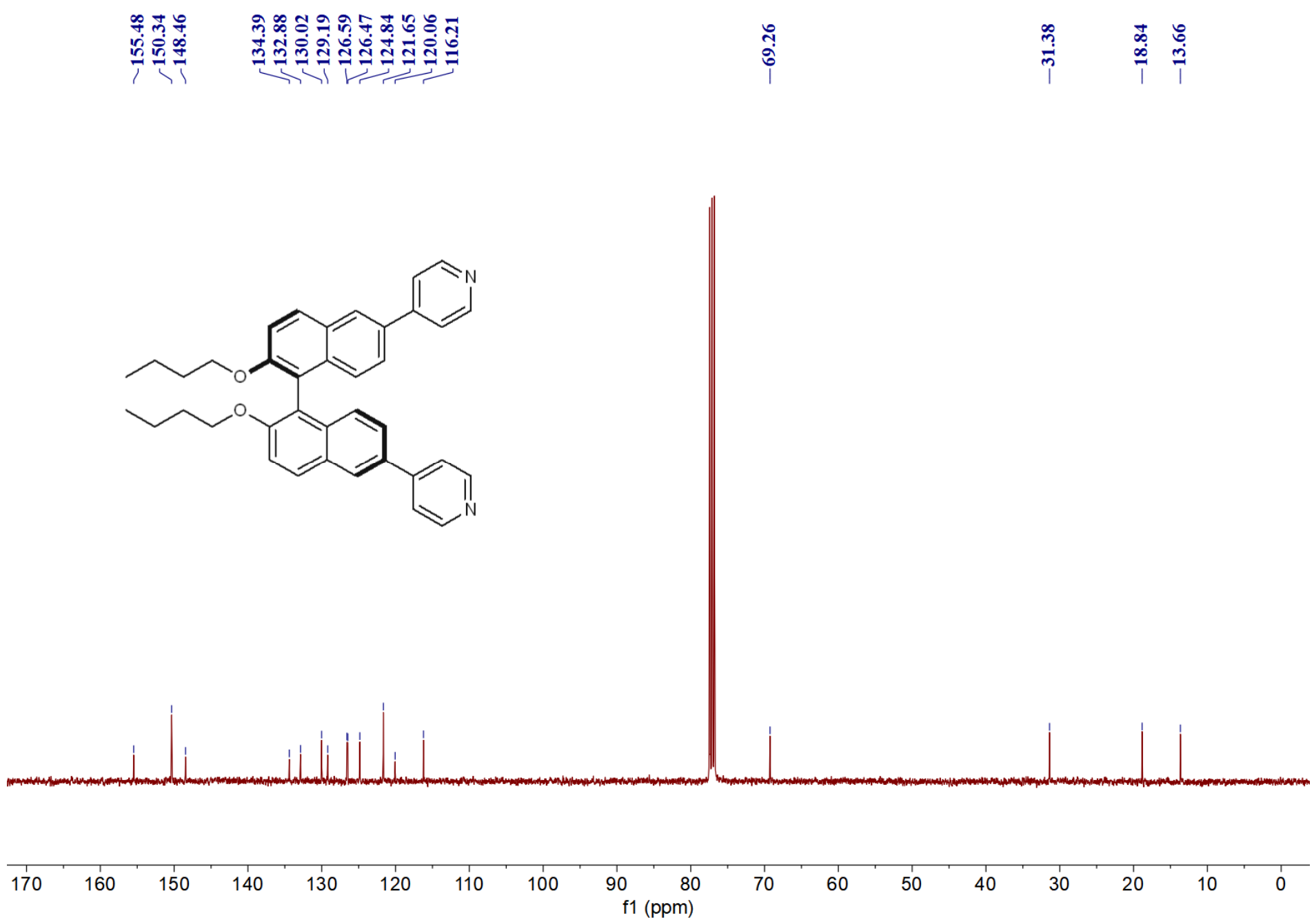

Supplementary Figure 42 | ^{13}C-NMR spectra of S-PyBN.

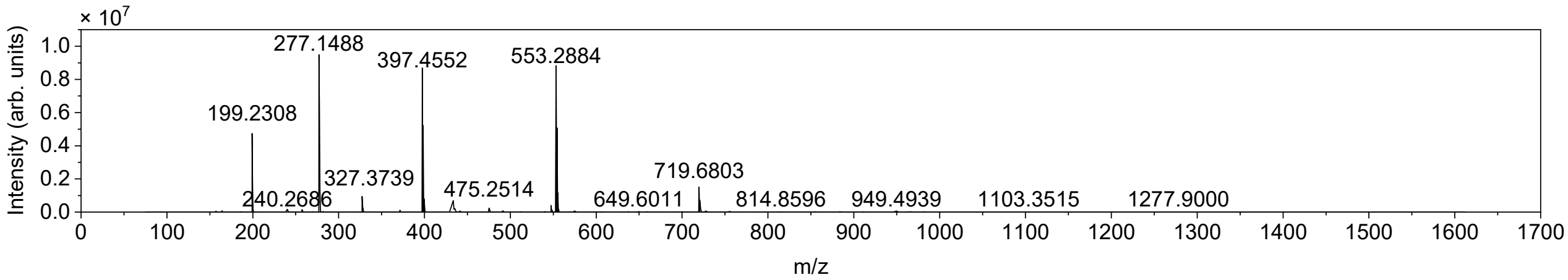

Supplementary Figure 43 | HRMS spectra of S-PyBN.

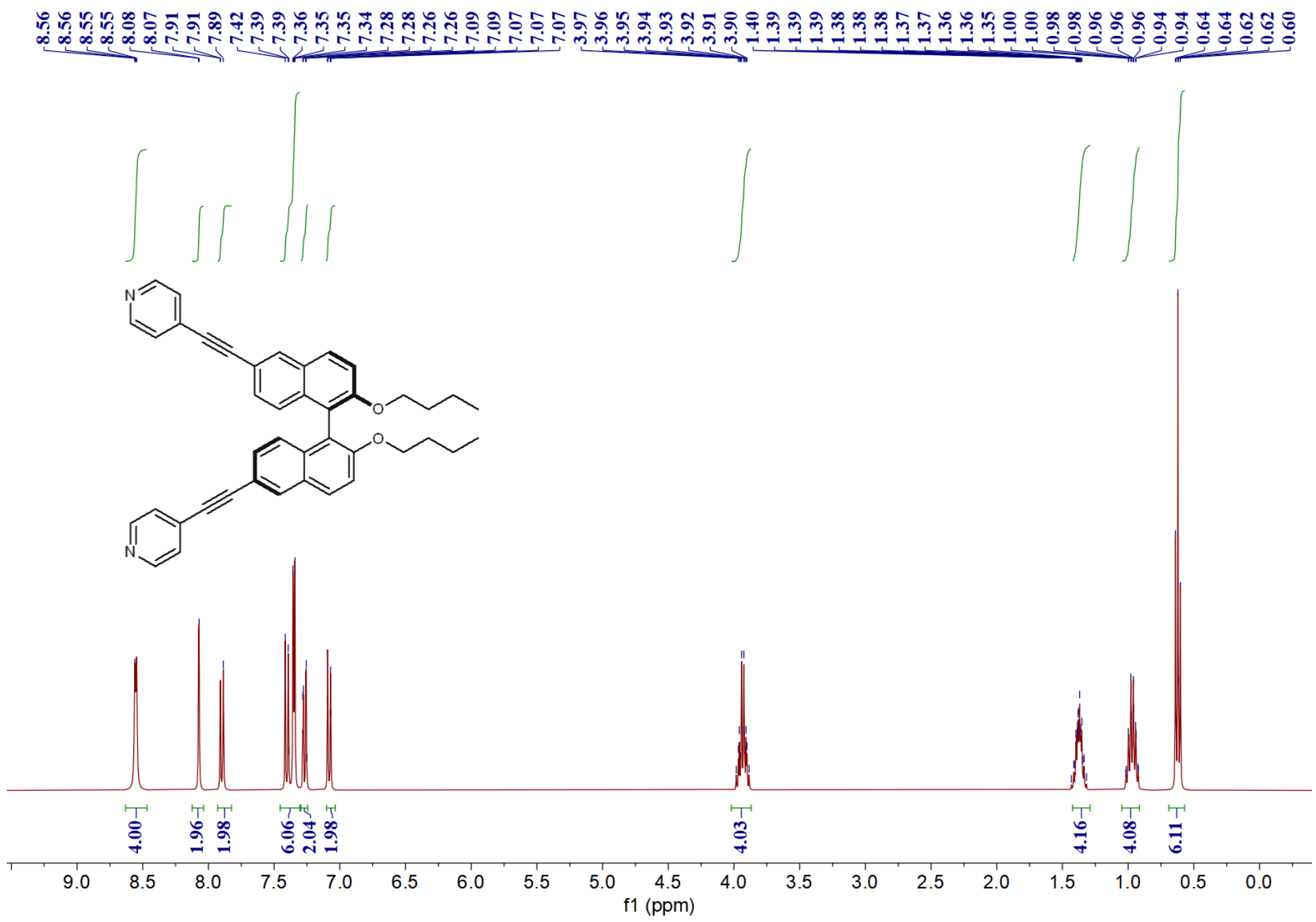

Supplementary Figure 44 | ^{1}H-NMR spectra of R-APyBN.

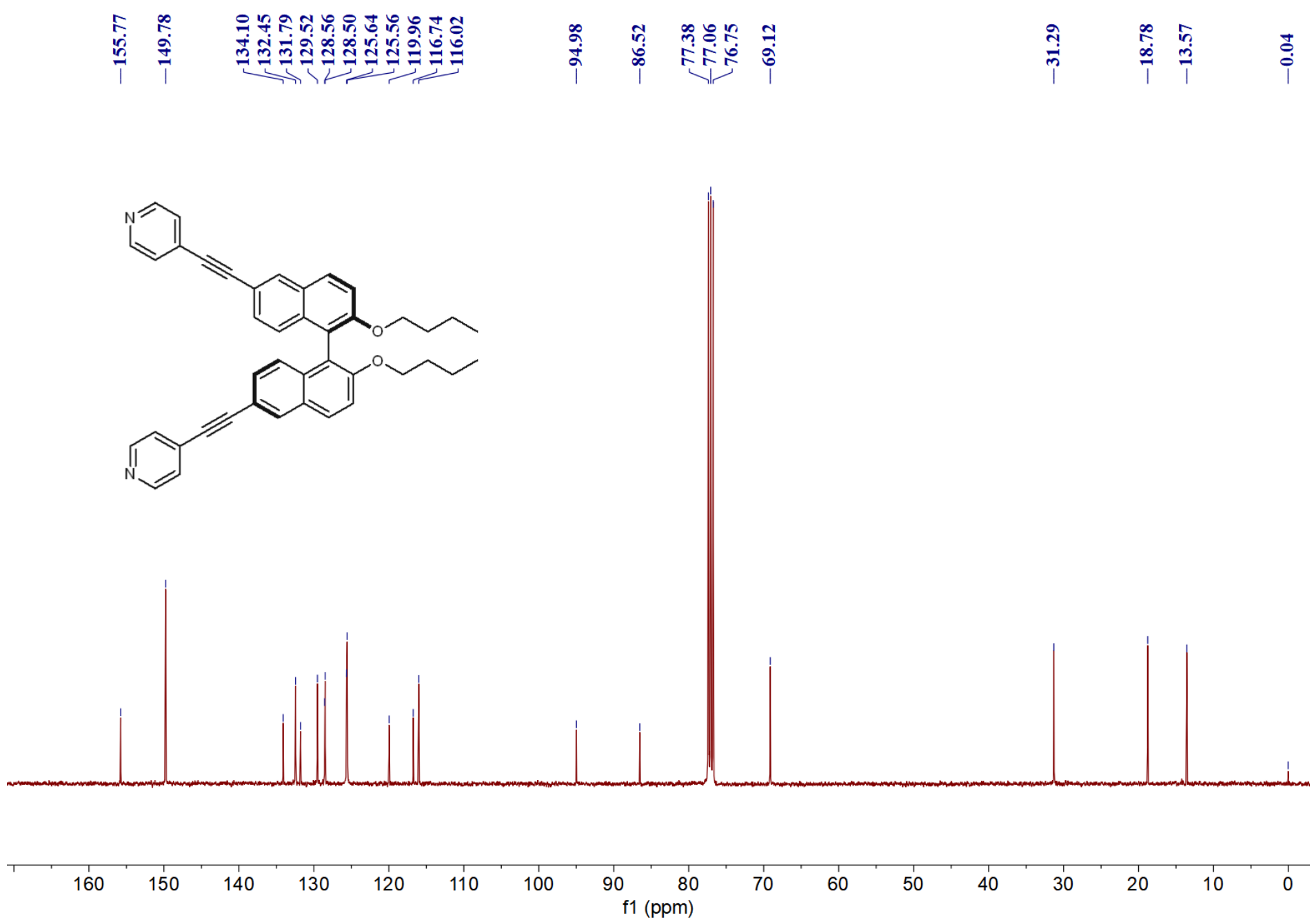

Supplementary Figure 45 | ^{13}C-NMR spectra of R-APyBN.

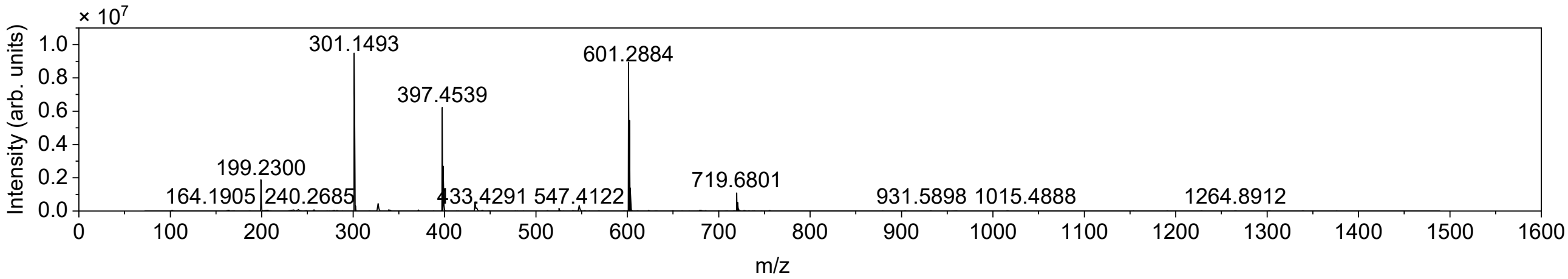

Supplementary Figure 46 | HRMS spectra of R-APyBN.

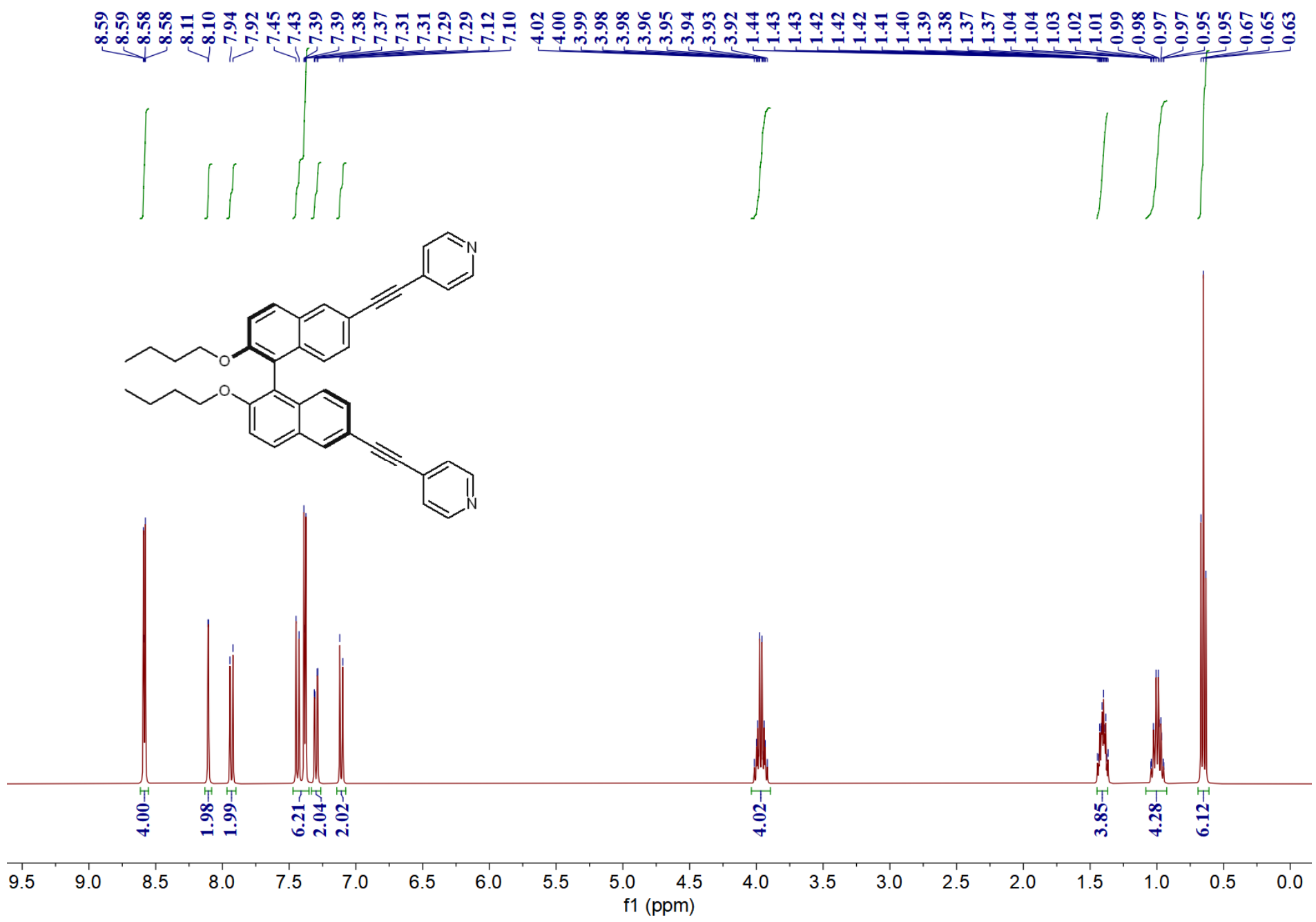

Supplementary Figure 47 | ¹H-NMR spectra of S-APyBN.

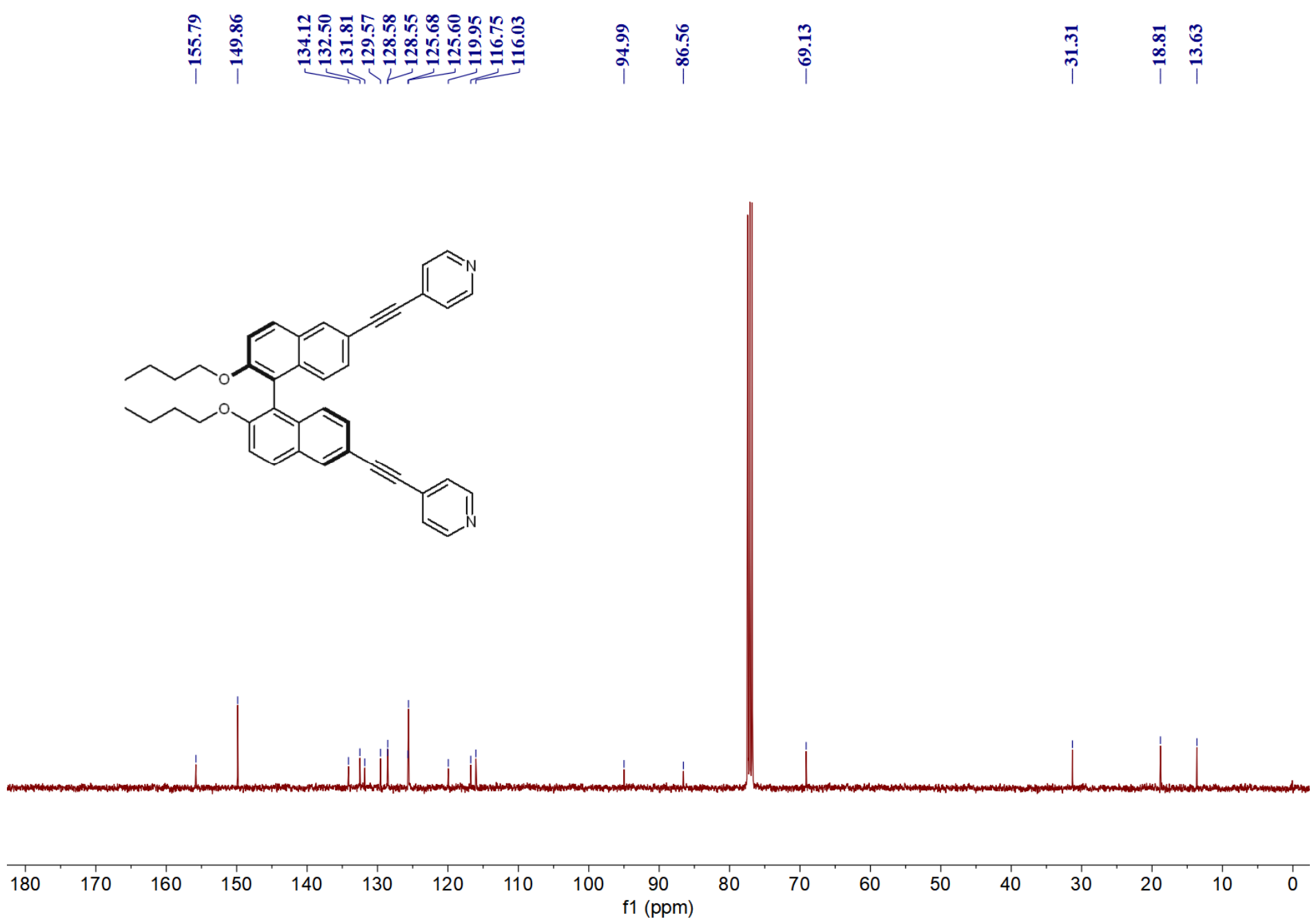

Supplementary Figure 48 | ^{13}C-NMR spectra of S-APyBN.

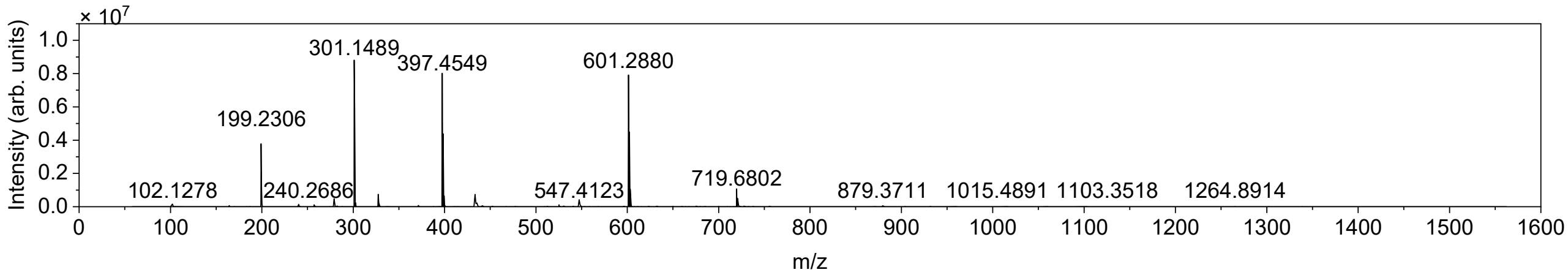

Supplementary Figure 49 | HRMS spectra of S-APyBN.

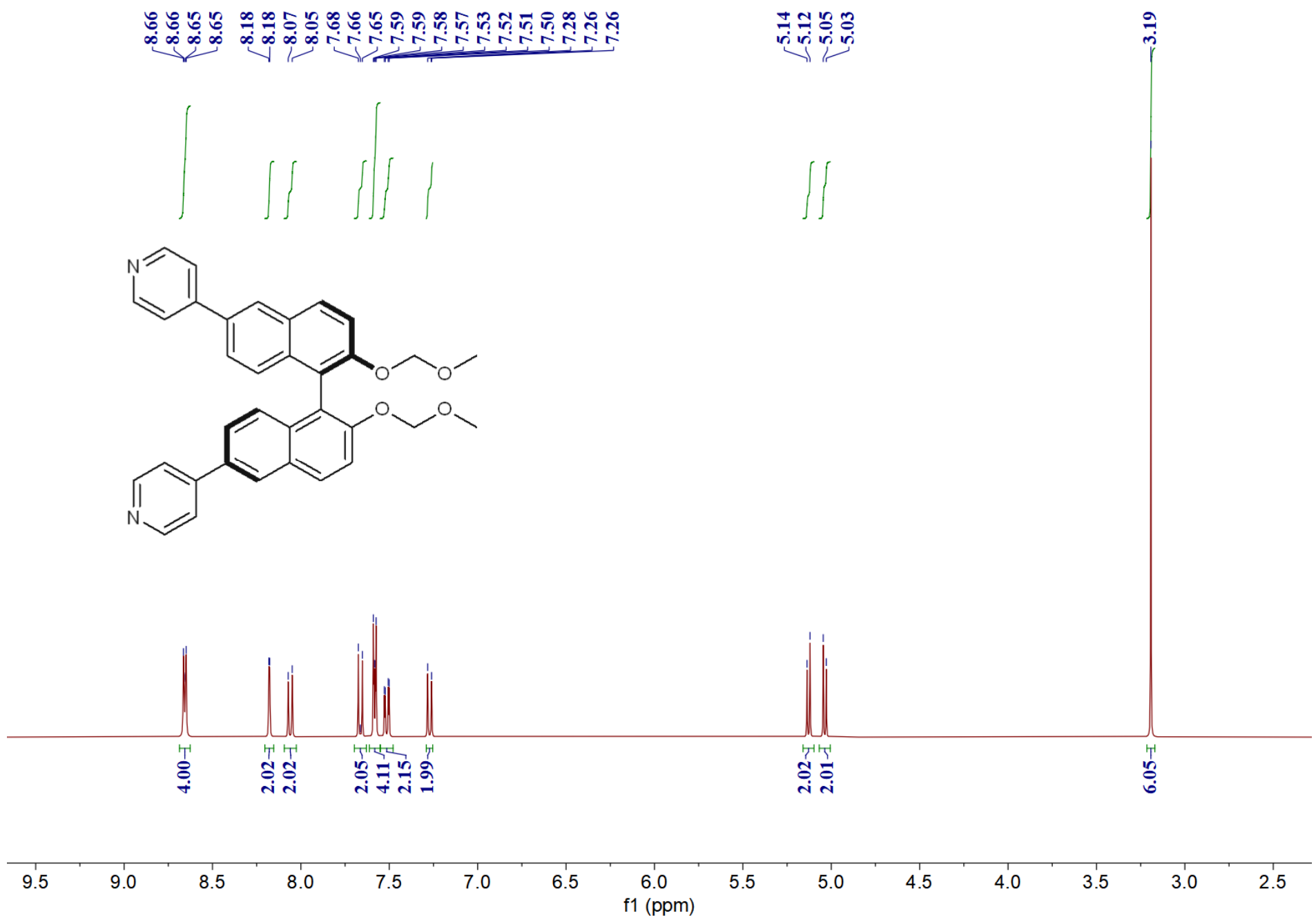

Supplementary Figure 50 | ^{1}H-NMR spectra of R-PyBNMO.

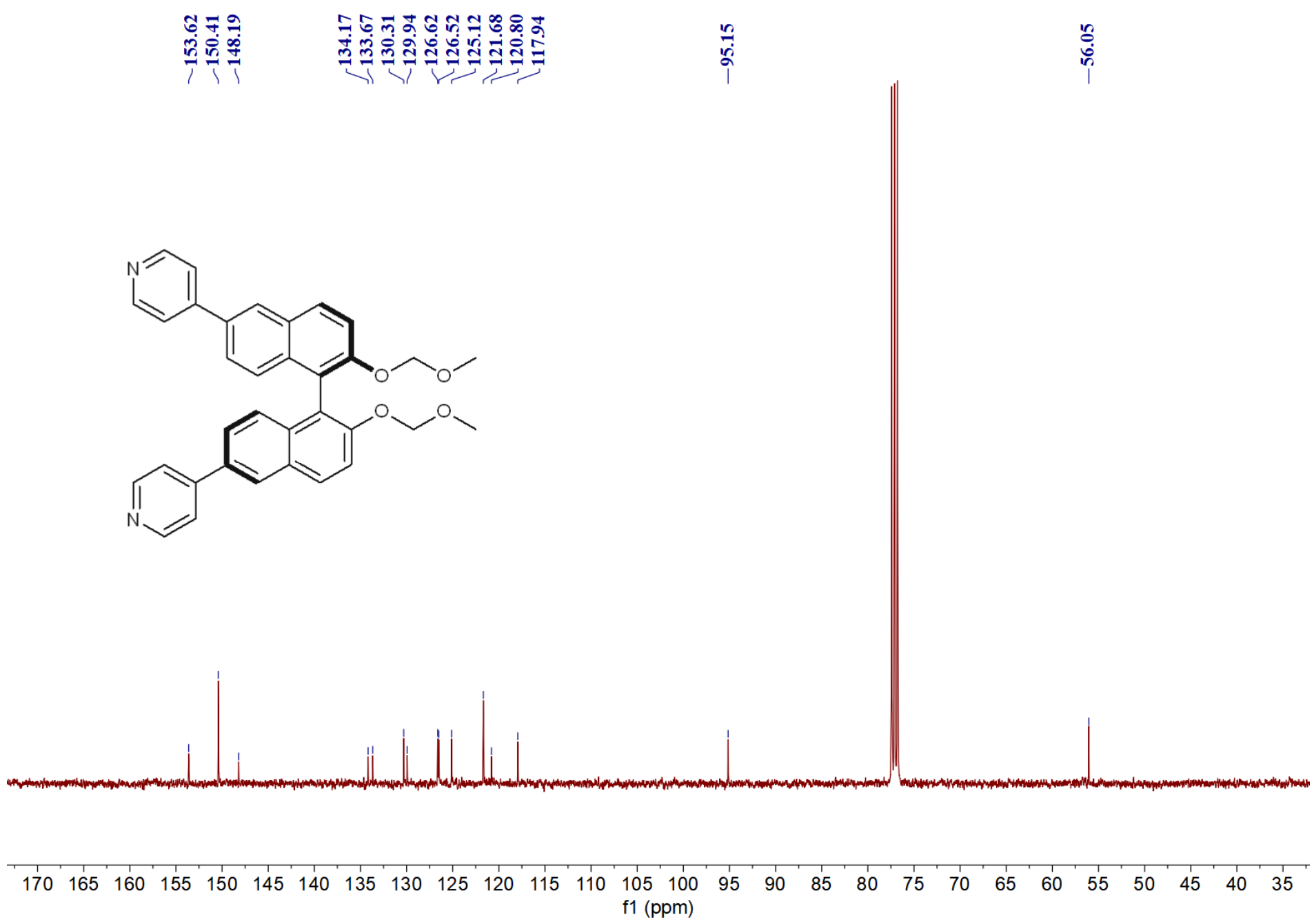

Supplementary Figure 51 | ^{13}C-NMR spectra of R-PyBNMO.

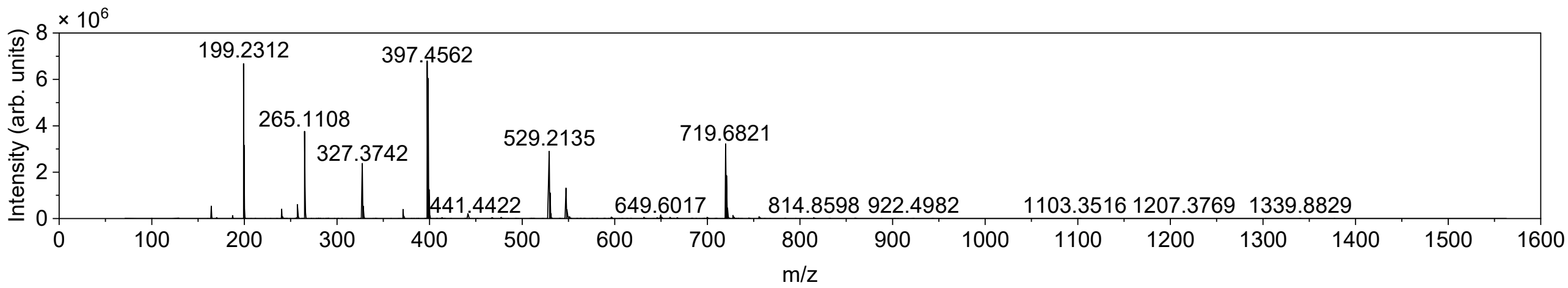

Supplementary Figure 52 | HRMS spectra of R-PyBNMO.

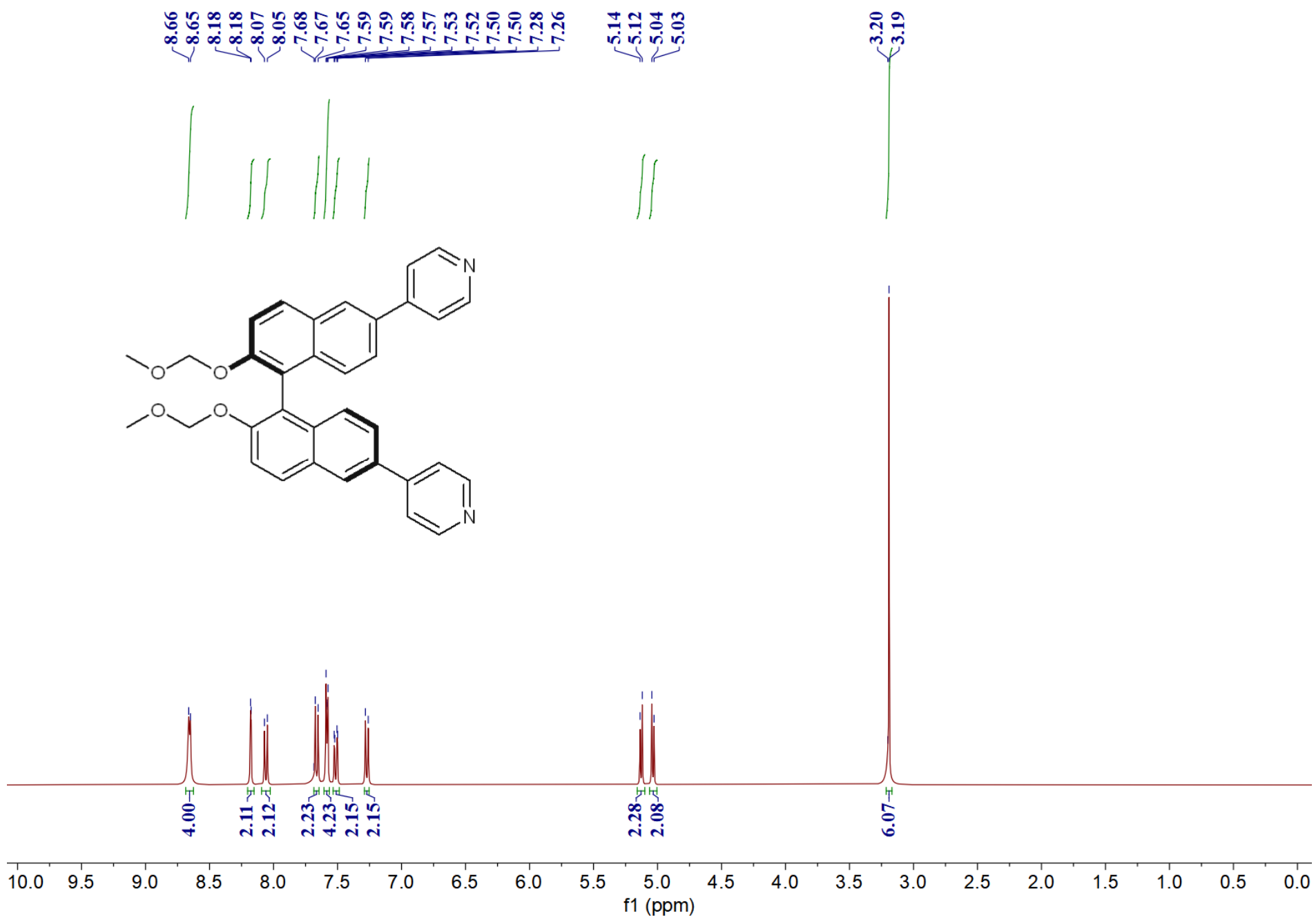

Supplementary Figure 53 | ^{1}H-NMR spectra of S-PyBNMO.

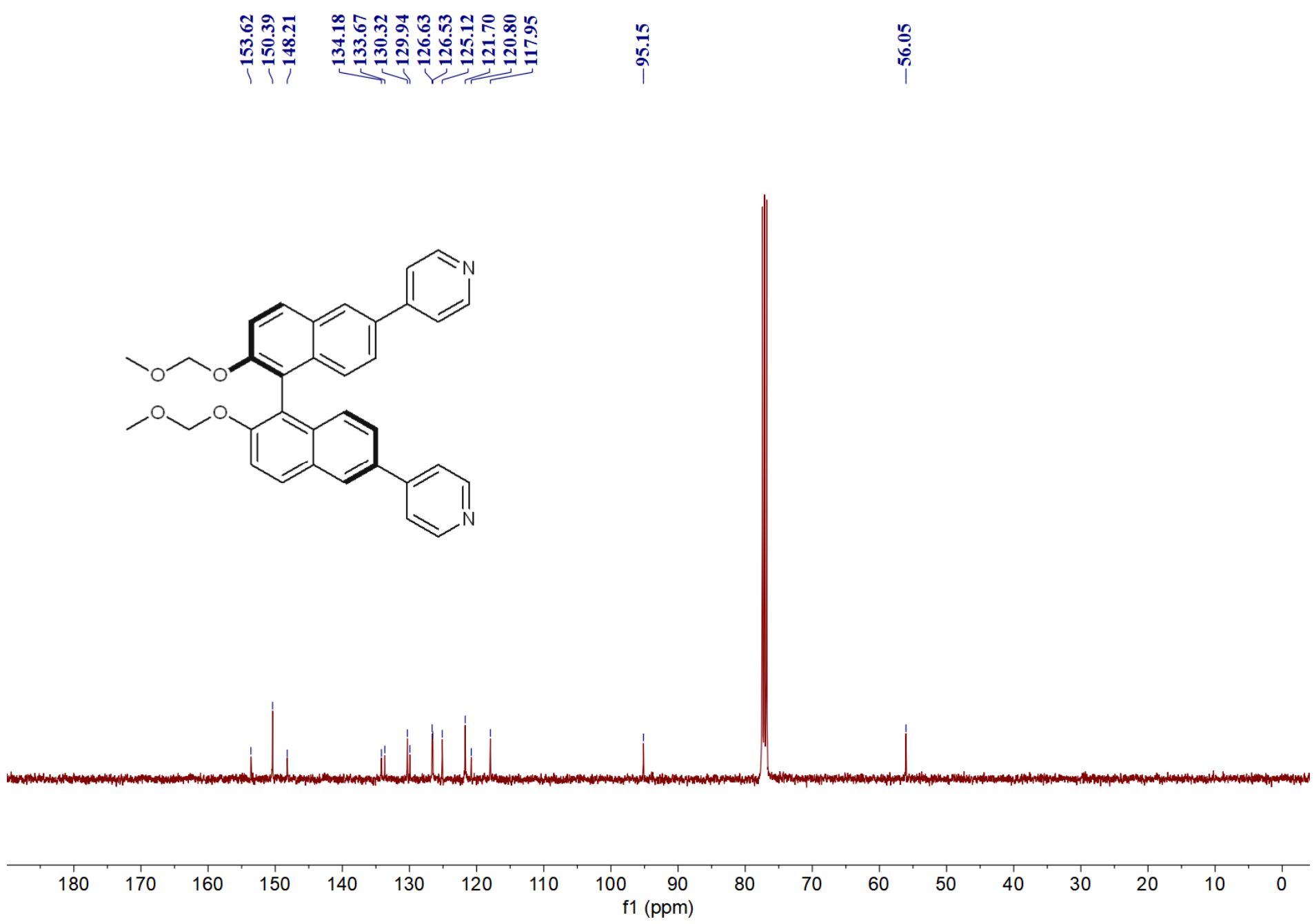

Supplementary Figure 54 | ^{13}C-NMR spectra of S-PyBNMO.

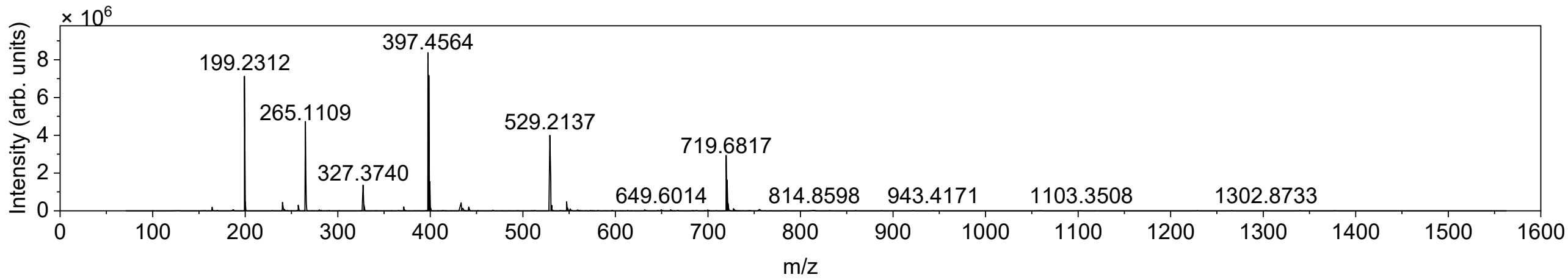

Supplementary Figure 55 | HRMS spectra of S-PyBNMO.

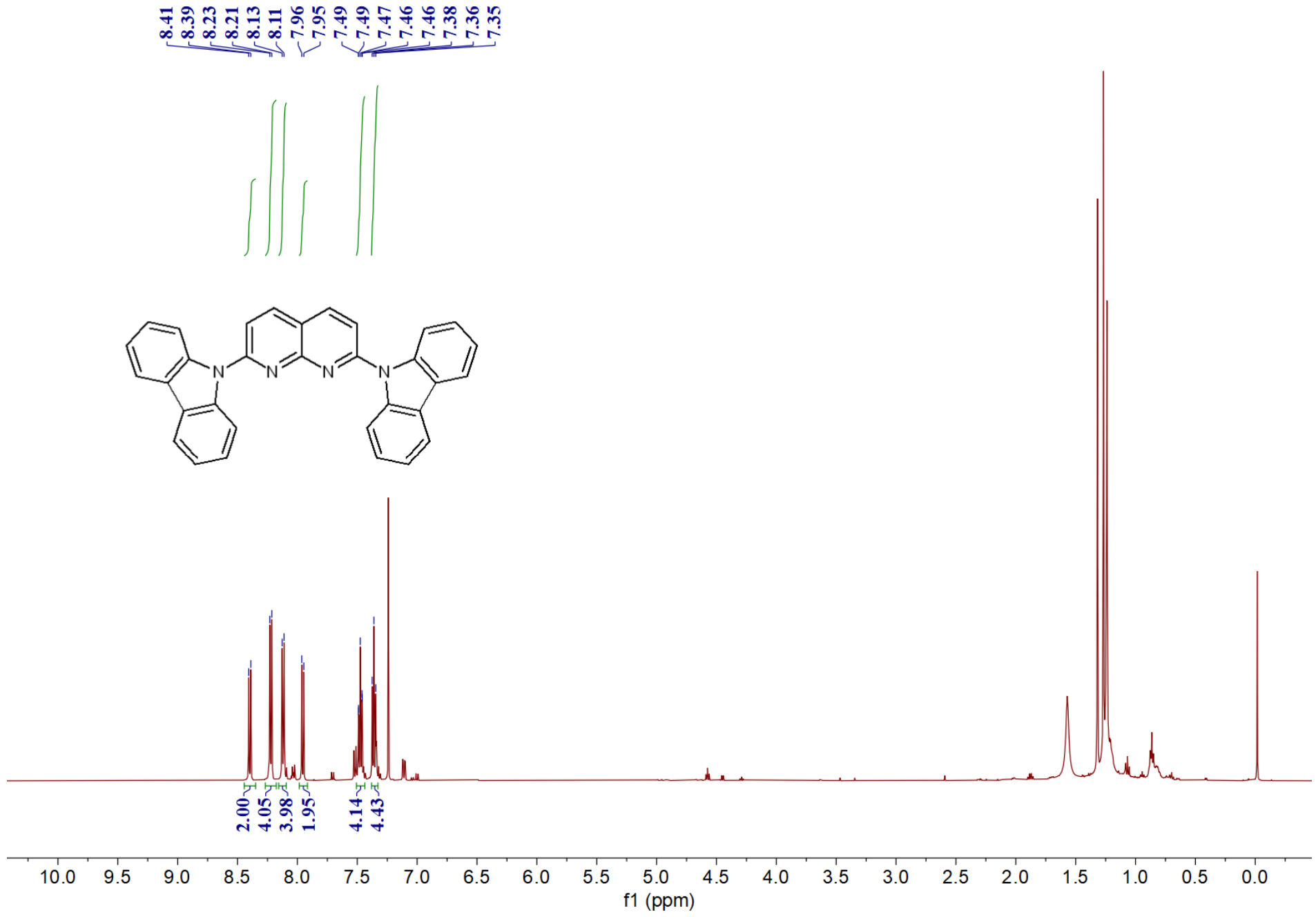

Supplementary Figure 56 | ^{1}H-NMR spectra of DCz-ND.

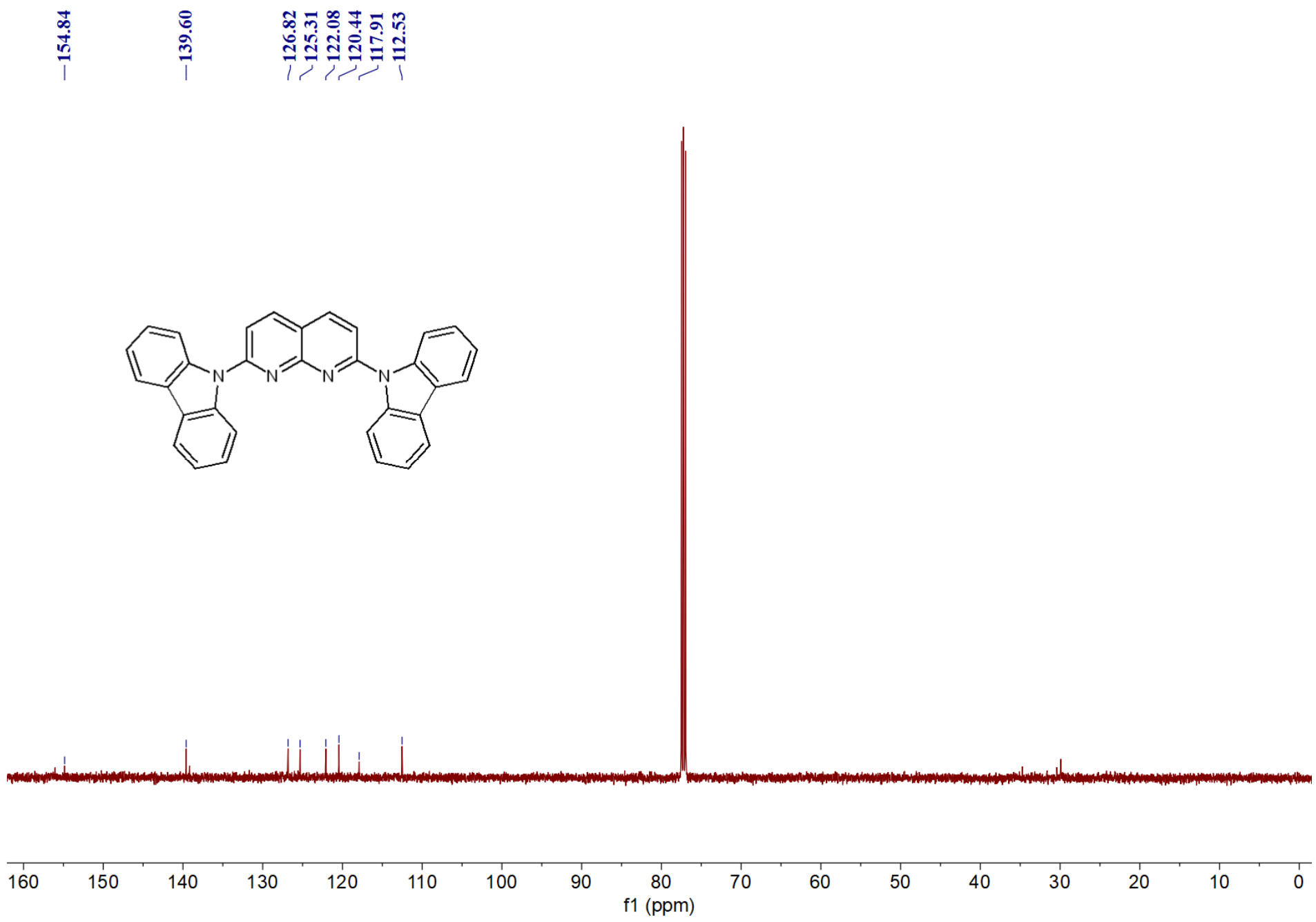

Supplementary Figure 57 | ^{13}C-NMR spectra of DCz-ND.

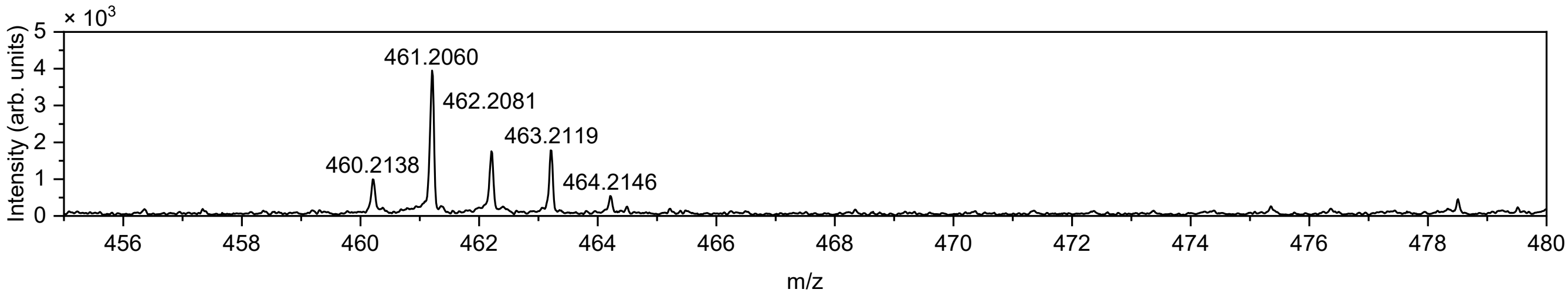

Supplementary Figure 58 | HRMS spectra of DCz-ND.

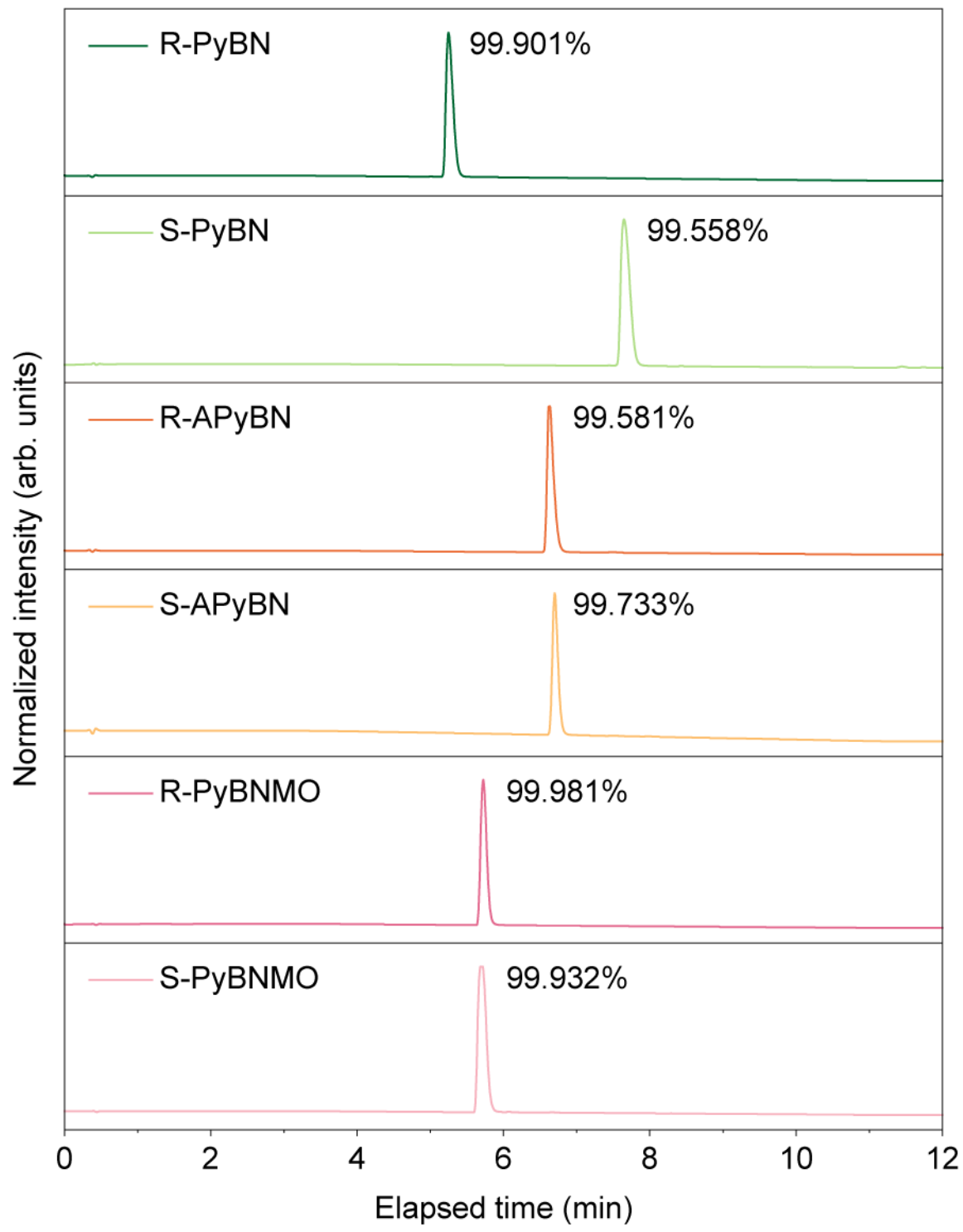

Supplementary Figure 59 | HPLC profiles of R/S-PyBN, R/S-APyBN, and R/S-PyBNMO monitored at 220 nm. Temperature, 40°C; mobile phase, H_2O/MeCN/TFA; flow rate = 2.5 mL min^{-1}; column, ultimate XB-C18 (50 × 4.6 mm, 5 μm).

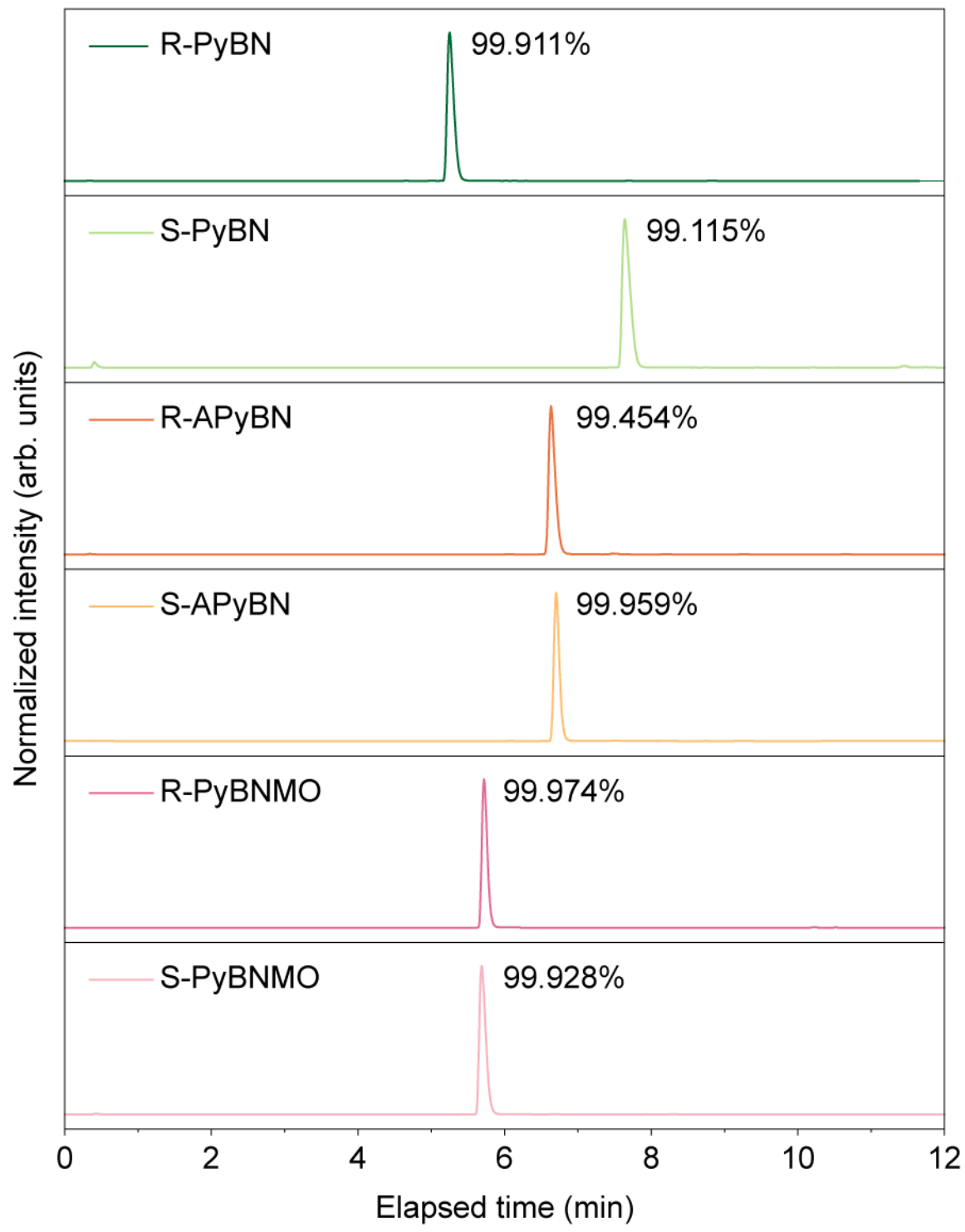

Supplementary Figure 60 | HPLC profiles of R/S-PyBN, R/S-APyBN, and R/S-PyBNMO monitored at 254 nm. Temperature, 40°C; mobile phase, H_2O/MeCN/TFA; flow rate = 2.5 mL min^{-1}; column, ultimate XB-C18 (50 × 4.6 mm, 5 µm).

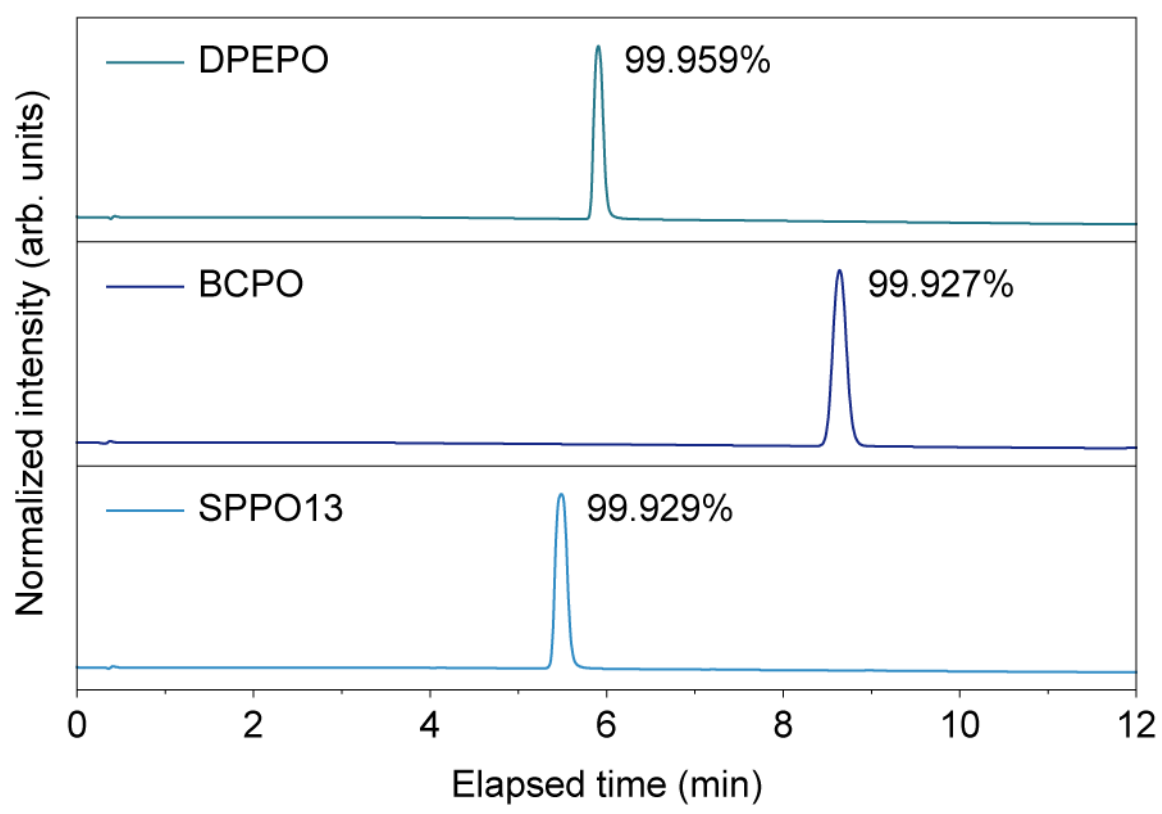

Supplementary Figure 61 | HPLC profiles of DPEPO, BCPO, and SPPO13 monitored at 220 nm. Temperature, 40°C; mobile phase, H_2O/MeCN/TFA; flow rate = 2.5 mL min^{-1}; column, ultimate XB-C18 (50 × 4.6 mm, 5 µm).

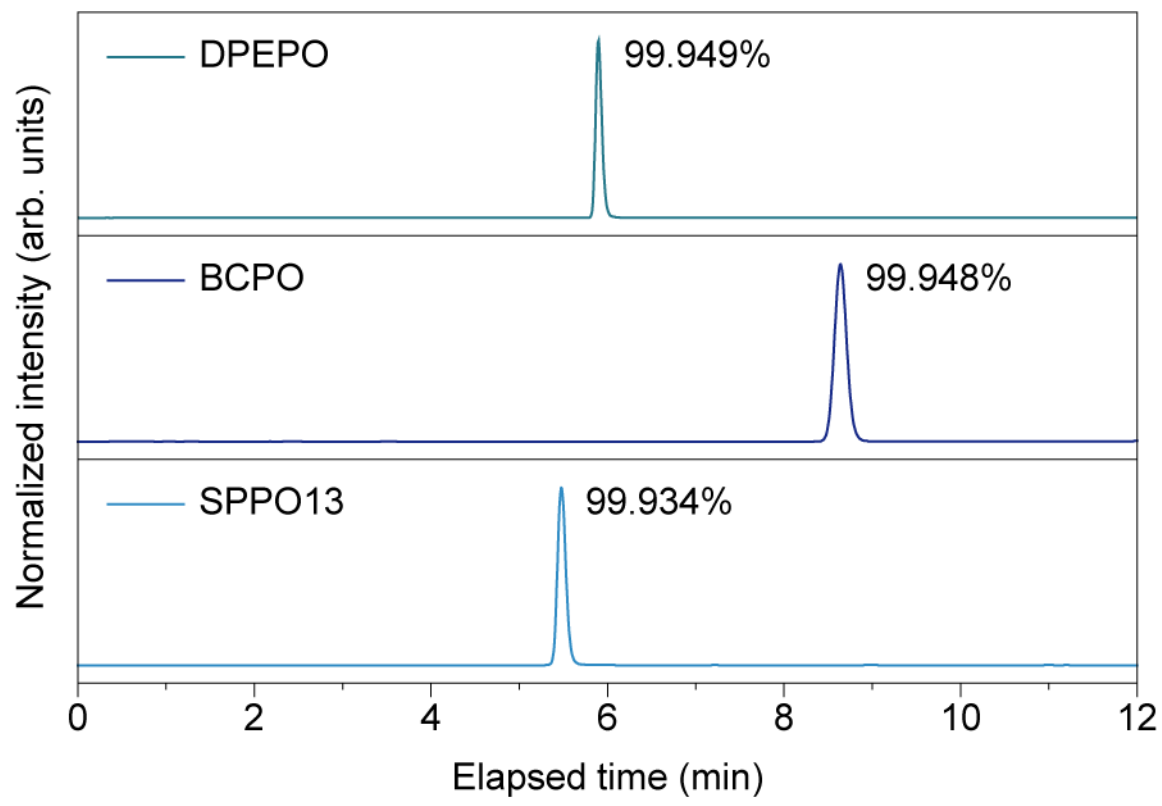

Supplementary Figure 62 | HPLC profiles of DPEPO, BCPO, and SPPO13 monitored at 254 nm. Temperature, 40°C; mobile phase, H_2O/MeCN/TFA; flow rate = 2.5 mL min^{-1}; column, ultimate XB-C18 (50 × 4.6 mm, 5 µm).

X. Supplementary References

1. Deng, W.-T. *et al.* Facile synthesis of homochiral compounds integrating circularly polarized luminescence and two-photon excited fluorescence. *Chem. Commun.* **55**, 2210–2213 (2019).
2. Shen, Y.-F. *et al.* Naphthyridine-based thermally activated delayed fluorescence emitters for highly efficient blue OLEDs. *Dyes and Pigments* **178**, 108324 (2020).
3. Lin, C. J., Zhuang, Y. X., Li, W. H., Zhou, T.-L. & Xie, R.-J. Blue, green, and red full-color ultralong afterglow in nitrogen-doped carbon dots. *Nanoscale* **11**, 6584–6590 (2019).
4. Wu, T. Y. *et al.* Stable white afterglow along the blackbody radiation line enabled by assisted partial phosphorescence resonance energy transfer. *Adv. Funct. Mater.* **35**, 2415525 (2025).
5. Jin, J. B. *et al.* Modulating tri-mode emission for single-component white organic afterglow. *Angew. Chem. Int. Ed.* **60**, 24984–24990 (2021).
6. Zhang, X. H., Chong, K. C., Xie, Z. L. & Liu, B. Color-tunable dual-mode organic afterglow for white-light emission and information encryption based on carbazole doping. *Angew. Chem. Int. Ed.* **62**, e202310335 (2023).
7. Kuila, S. M., Garain, S., Bandi, S. & George, S. J. All-organic, temporally pure white afterglow in amorphous films using complementary blue and greenish-yellow ultralong room temperature phosphors. *Adv. Funct. Mater.* **30**, 2003693 (2020).
8. Wang, Y. G. *et al.* Persistent organic white-emitting afterglow from ultralong thermally activated delayed fluorescence and room-temperature phosphorescence. *Adv. Opt. Mater.* **9**, 2101075 (2021).
9. Liang, Y. H. *et al.* Enabling highly robust full-color ultralong room-temperature phosphorescence and stable white organic afterglow from polycyclic aromatic hydrocarbons. *Angew. Chem. Int. Ed.* **63**, e202318516 (2024).
10. Jinnai, K. Z. Y., Kabe, R. & Adachi, C. H. Y. Wide-range tuning and enhancement of organic long-persistent luminescence using emitter dopants. *Adv. Mater.* **30**, 1800365 (2018).
11. Liang, X., Zheng, Y. & Zuo, J. Two-photon ionization induced stable white organic long persistent luminescence. *Angew. Chem. Int. Ed.* **60**, 16984–16988 (2021).
12. Zeng, M. J. *et al.* Enabling robust blue circularly polarized organic afterglow through self-confining isolated chiral chromophore. *Nat. Commun.* **15**, 3053 (2024).
13. Ning, W. M., Wang, H., Gong, S. L., Zhong, C. & Yang, C. L. Simple sulfone-bridged heterohelicene structure realizes ultraviolet narrowband thermally activated delayed fluorescence, circularly polarized luminescence, and room temperature phosphorescence. *Sci. China: Chem.* **65**, 1715–1719 (2022).
14. Garain, S., Sarkar, S., Chandra Garain, B., Pati, S. K. & George, S. J. Chiral arylene diimide phosphors: circularly polarized ambient phosphorescence from bischromophoric pyromellitic diimides. *Angew. Chem. Int. Ed.* **61**, e202115773 (2022).
15. Gu, L. *et al.* Circularly polarized organic room temperature phosphorescence from amorphous copolymers. *J. Am. Chem. Soc.* **143**, 18527–18535 (2021).
16. Yu, F. *et al.* Long-persistent circularly polarized luminescence from a host-guest system regulated by the multiple roles of a gold(I)-carbene motif. *Angew. Chem. Int. Ed.* **62**, e202312927 (2023).
17. Liang, X. *et al.* Organic room-temperature phosphorescence with strong circularly polarized luminescence based on paracyclophanes. *Angew. Chem. Int. Ed.* **58**, 17220–17225 (2019).
18. Li, H. *et al.* Single-component color-tunable circularly polarized organic afterglow through chiral clusterization. *Nat. Commun.* **13**, 429 (2022).
19. Li, H. *et al.* Stimuli-responsive circularly polarized organic ultralong room temperature phosphorescence. *Angew. Chem. Int. Ed.* **59**, 4756–4762 (2020).
20. Zhang, J. Y. *et al.* Highly bright pure room temperature phosphorescence for circularly polarized organic hyperafterglow. *Adv. Mater.* **37**, 2500953 (2025).
21. Wang, T. T. *et al.* Long-lived charge separation induced organic long-persistent luminescence with circularly

polarized characteristic. *Adv. Opt. Mater.* **11**, 2202613 (2023).
22. Yu, L. *et al.* Time-dependent colorful circularly polarized organic ultralong room temperature phosphorescence from a single-component chiral molecule. *Small* **19**, 2303579 (2023).
23. Jiang, Y. T. *et al.* Chiral-guest induced multicolor-tunable circularly polarized room temperature phosphorescence. *Adv. Opt. Mater.* **12**, 2302482 (2024).
24. Wei, L. Z. *et al.* Tuning circularly polarized afterglow color via modulation of energy and chirality transfer in Co-doped films. *Adv. Funct. Mater.* **34**, 2409681 (2024).
25. Zhang, C. Y. *et al.* Self-confining isolated chiral chromophore for circularly polarized organic afterglow from amorphous polymer systems. *Chem. Eng. J.* **485**, 149886 (2024).
26. Qin, T. *et al.* Circularly polarized organic long-persistent luminescence from chiral binaphthyl guests. *J. Phys. Chem. Lett.* **16**, 10712–10719 (2025).
27. Guo, G. Y. *et al.* A dynamic H-bonding network enables stimuli-responsive color-tunable chiral afterglow polymer for 4D encryption. *Adv. Mater.* **36**, 2412100 (2024).
28. Zhang, D., Li, M. & Chen, C. Linear axially chiral conjugated polymers exhibiting ultralong low-temperature phosphorescence and intense circularly polarized luminescence. *Angew. Chem. Int. Ed.* **61**, e202213130 (2022).
29. Chen, T. H. & Yan, D. P. Radical-induced hour-level afterglow and efficient circularly polarized luminescence from metal halide hybrid glasses. *Angew. Chem. Int. Ed.* **65**, e8889513 (2026).
30. Li, H. *et al.* Enabling multicolor circularly polarized organic long persistent luminescence through chiral exciplex. *Adv. Mater.* **37**, 2500841 (2025).
31. Malatong, R. *et al.* Circularly polarized long-persistent and photostimulated luminescence enabled through förster resonance energy transfer and upconversion strategies. *Adv. Sci.* **13**, e23415 (2026).
