# Supplementary material for: Trap-induced white circularly polarized persistent luminescence from a host-guest molecular system
Source: Nat Commun. 2026 Aug 7;17:9517. doi: 10.1038/s41467-026-76289-7 (PMC13547245; doi:10.1038/s41467-026-76289-7)
Supplement: Supplementary file 2 [file 41467_2026_76289_MOESM2_ESM.pdf]

Description of Additional Supplementary Information

Supplementary Data 1: The atomic coordinates of the optimized models calculated by the density functional theory (DFT). These files provide the atomic coordinates of the optimized computational models for the corresponding molecules and that of radical cations and anions.
