# Supplementary material for: Trap-induced white circularly polarized persistent luminescence from a host-guest molecular system
Source: Nat Commun. 2026 Aug 7;17:9517. doi: 10.1038/s41467-026-76289-7 (PMC13547245; doi:10.1038/s41467-026-76289-7)
Supplement: Supplementary file 3 [file 41467_2026_76289_MOESM4_ESM.pdf]

nature portfolio

Peer Review File

Trap-Induced White Circularly Polarized Persistent Luminescence from A Host-Guest Molecular System

Corresponding Author: Professor Rong-Jun Xie

This file contains all reviewer reports in order by version, followed by all author rebuttals in order by version.

Version 0:

Reviewer comments:

Reviewer #1

(Remarks to the Author)
The manuscript reports white circularly polarized persistent luminescence (CP-PersL) in organic host–guest systems, achieved via trap-mediated charge separation and recombination, enabling hour-long emission and moderate CPL activity. The authors propose that detrapped electrons redistribute between singlet and triplet excited states resulting in temporally stable white emission combined with chirality. The work showed a general strategy and demonstrated across several host–guest combinations with applications in information encryption. The topic is relevant, and the manuscript is experimentally rich and well illustrated. However, while the results are interesting, several conceptual, mechanistic, and methodological issues must be addressed before the work can meet the standards of Nature Communications. In particular, the novelty relative to existing organic PersL literature, the rigor of the trap analysis, and the mechanistic justification of singlet–triplet redistribution require clarification.
Recommendation: Major Revision

Here are some comments the authors are invited to address:
1)The authors present the work as a "new way" to realize white CP-PersL via trap states. However: trap-mediated organic persistent luminescence is already well established, including by the authors themselves. Also, charge separation → trapping → thermal detrapping → recombination seems very close to earlier exciplex-based and trap-based PersL systems. The authors should explicitly and quantitatively compare their system with existing white PersL systems (organic and inorganic) and
prior CP-PersL or long-lived CPL materials (especially those beyond the ms–s regime). Maybe a table can help.
The main claimed novelty appears to be: white emission from single guest species via combined S_1 and T_1 emission and retention of CPL during hour-long PersL.
Furthemore, the authors refer to the emission as white; however, the spectra are dominated by two discrete blue and yellow bands. Clarification on whether this should be considered warm-white rather than true broadband white would improve precision.
2) The persistent luminescence decay curves are presented without any fitting (e.g., graphs do not show fit curves) or extraction of kinetic parameters. Given that the proposed mechanism relies on trap-controlled recombination and multiple emissive channels, quantitative decay analysis would be necessary to support the interpretation.
3) The manuscript claims that detrapped electrons redistribute between singlet and triplet excited states, leading to white emission.
What direct experimental evidence supports this redistribution? In particular, can the authors provide wavelength-resolved decay curves or time-resolved spectra distinguishing singlet and triplet contributions during PersL?
4) The agreement between DFT-derived LUMO offsets and experimental trap depths is presented as strong validation of the model. However, the averaging of α and β spin orbital energies is not rigorously justified. Typically, DFT (with Gaussian) results provide qualitative support for open-shell species and solid-state environments. Quantitative support in these cases is questionable, although the provided numbers match well with the experiments. Explicit discussion of limitations is necessary.
5) The melt-casting method at 320 °C raises concerns about thermal degradation, which are not discussed.
6) Lifetime decay profiles of Figure S10 are characterized by low signal-to-noise ratios after a few ms, with data points scattered within an order of magnitude interval (in intensity). These should be improved if the authors have considered these points in the fitting procedure.
7) It is not clear if the structured phosphorescent component in R/S-PyBN is only observable at 77 K. If so, how did the

authors measure the decay at RT? Is the structured phosphorescent component present at RT only in the film?
8) Encryption demonstration is visually appealing but conceptually incremental, as similar demonstrations exist; this should be framed as a proof-of-concept rather than a major advance.
9) The authors said "the electron is mobile within host molecules, and then captured by another guest to generate (R/S-PyBN)•−*". Considering a 1% "doping" in the film, is it reasonable to also consider an intramolecular process?
10) The abstract claims "multi-dimensional properties". Does the paper deliver an actual case of multi-dimensional functionality with independent and orthogonal different properties? Please, discuss or rephrase.

Reviewer #2

(Remarks to the Author)
This paper (NCOMMS-26-007435) reported a general approach to achieve hour-level white persistent luminescence to CPL materials through the introduction of trap states into an organic host-guest molecular system. The R/S-PyBN@DPEPO can produce white circularly polarized persistent luminescence for 4 h at room temperature. This study demonstrates that two different chiral configurations leave the trap depth basically unaltered, while conferring pronounced CPL activity on these composites with a maximum luminescence dissymmetry factor glum of 1.6 × 10-3/-2.3 × 10-3. White circularly polarized persistent luminescence materials can be applied for Morse-code encryption and multidimensional information storage. In general, the manuscript presents an effective method to develop a kind of application field of CPL materials. I think this paper can be recommended for publication on Nature Communications after the following revision:

1. R/S-PyBN@SPPO13 should exhibit mirror-imaged CD bands. However, from the Supplementary Figure 15, the mirroring cannot be discerned.
2. Compared with R/S-PyBN in toluene, the fluorescence emission of R/S-PyBN film showed a red shift, the explanation should be provided in the maintext.
3. Lifetime decay profiles of DPEPO in a neat film at 77 K should be measured.
4. The phosphorescence spectra of R/S-PyBN@DPEPO showed a blue shift compared to that of pure R/S-PyBN in neat films at 77 K. Please explain it.
5. The author mentioned "different chiral configurations leave the trap depth basically unaltered", but the log-log plot of PersL decay curves of 1 wt% R/S-PyBN@SPPO13 showed significantly different luminescence times.
6. The CD spectra of R/S-APyBN@DPEPO (Ri/S-i), R/S-PyBNMO@DPEPO (R-ii/S-ii), R/S-PyBN@BCPO (R-iii/S-iii), and R/S-PyBN@SPPO13 (R-iv/S-iv) should be measured.
7. There are some textual errors in the text, such as "R-PyBN@SPPO13" should be "S-PyBN@SPPO13"in Supplementary Figure 25 d. And the format of reference 51, 55 is different from the others. Please correct it.

Reviewer #3

(Remarks to the Author)
This manuscript reports a host-guest molecular system (R/S-PyBN@DPEPO, etc.) that achieves white circularly polarized persistent luminescence (CP-PersL) through the introduction of trap states. The authors claim that the material exhibits persistent luminescence for over 4 hours at room temperature, with a maximum luminescence dissymmetry factor |glum| on the order of 10-3. Applications in Morse code encryption and multidimensional information storage are also demonstrated. After careful review, I find that this manuscript has the following fundamental issues and does not meet the publication standards of Nature Communications:

1.Trap-induced organic persistent luminescence was not first proposed in this study. Furthermore, the strategy of achieving white persistent luminescence through a combination of singlet-singlet and singlet-triplet transitions has been extensively reported in organic afterglow materials. Examples include Angew. Chem. Int. Ed. 2021, 60, 24984; Angew. Chem. Int. Ed. 2023, 62, e202310335, among others. This work merely introduces chiral molecules into an existing trap-induced luminescence system. Such "chiralization" represents incremental work and lacks a substantial breakthrough.
2.The claim of achieving white light due to comparable singlet and triplet emission intensities is not well-supported by the data. Figure 2d shows that in the persistent luminescence spectrum, the yellow/orange emission component (~550-600 nm) is significantly stronger than the blue component (~400 nm). The CIE coordinates (0.36, 0.42) are actually closer to yellow light than to the ideal white point (0.33, 0.33).
3.The performance metrics lack competitiveness, and the dissymmetry factor is relatively low. The reported |glum| values are only on the order of 10^{-4}-10^{-3}. This is not suitable for the claimed encryption applications, as chiral luminescent materials with |glum| < 10^{-2} are highly susceptible to producing false positive/negative signals due to sample preparation variations. Moreover, the two chiral enantiomers show no significant difference in their afterglow luminescence, rendering them not directly meaningful for chirality-based encryption.
4.The luminescence quantum yield is not explicitly reported. This is crucial for evaluating the practical application value of luminescent materials.
5.The purity of the host and guest materials used in the study needs to be verified by high-performance liquid chromatography (HPLC) to rule out room-temperature phosphorescence originating from impurities.
6.The investigation into the properties of the host and guest materials is insufficient. The authors need to demonstrate whether the host and guest materials, when existing independently in powder or thin-film form, exhibit room-temperature phosphorescence.
7.The authors claim that the material exhibits persistent luminescence for over 4 hours at room temperature. However, in the text, there is only " The phosphorescence spectra of the R/S-PyBN@DPEPO showed two emission peaks at 531/531 and

572/572 nm), with lifetime of 2.72/5.34 and 3.87/3.73 ms at RT (Fig. 2a and Supplementary Fig. 14)." The decay lifetime should be scientifically measured by instrument but not by sense.
8.White emission has a wide application in light. White emission has no advantage for the application in optical information encryption.
9.White emission is usually composed of blue+yellow emission or blue+green+red emission. The efficiency of white emission form ultraviolet+yellow emission is often low with poor CIE and CRI (color rendering index). What are the advantages of this system?
10.Fig. 3d is ESR spectra recorded after UV irradiation at 110 K (green curves) and 300 K (orange curves). The authors contribute it to the formation of radicals with unpaired electrons. Since the persistent luminescence comes form triplet, which also has unpaired electrons, the authors need to conduct more experiments to rule out this possibility.

Version 1:

Reviewer comments:

Reviewer #1

(Remarks to the Author)
The authors have responded to all the requests and critical issues raised by this reviewer. Following the proposed revision, the manuscript has reached the standards required for publication and deserves to be published in its present form.

Reviewer #2

(Remarks to the Author)
Authors have carefully revised this manuscript according to reviewers' comments. The revised manuscript can be accepted for publication in Nature Communication.

Reviewer #3

(Remarks to the Author)
The phosphorescence spectra of the R/S-PyBN@DPEPO showed two emission peaks at531/531 and 572/572 nm, with lifetime of 2.72/5.34 and 3.87/3.73 ms at RT (Fig. 2a and Supplementary Fig. 14). If the phosphorescence lifetime is not 4 h but much lower tban 1 s, I think the innovation of this manuscript is low and does not meet the publication standards of Nature Communications.

Open Access This Peer Review File is licensed under a Creative Commons Attribution 4.0 International License, which permits use, sharing, adaptation, distribution and reproduction in any medium or format, as long as you give appropriate credit to the original author(s) and the source, provide a link to the Creative Commons license, and indicate if changes were made.
In cases where reviewers are anonymous, credit should be given to 'Anonymous Referee' and the source.
The images or other third party material in this Peer Review File are included in the article's Creative Commons license, unless indicated otherwise in a credit line to the material. If material is not included in the article's Creative Commons

license and your intended use is not permitted by statutory regulation or exceeds the permitted use, you will need to obtain permission directly from the copyright holder.
To view a copy of this license, visit https://creativecommons.org/licenses/by/4.0/

Dear reviewers:

We sincerely appreciate all the three reviewers for taking time to review our manuscript and providing constructive suggestions, which we believe are highly important to improve the quality of the manuscript. We have made necessary revisions to address all the concerns. The modifications are marked in red color in the revised manuscript and Supplementary Information file. The followings are the **point-by-point response** to *the reviewers' comments* and the **actions** we taken in this round of revision.

Reviewer #1:

The manuscript reports white circularly polarized persistent luminescence (CP-PersL) in organic host–guest systems, achieved via trap-mediated charge separation and recombination, enabling hour-long emission and moderate CPL activity. The authors propose that detrapped electrons redistribute between singlet and triplet excited states resulting in temporally stable white emission combined with chirality. The work showed a general strategy and demonstrated across several host–guest combinations with applications in information encryption. The topic is relevant, and the manuscript is experimentally rich and well illustrated. However, while the results are interesting, several conceptual, mechanistic, and methodological issues must be addressed before the work can meet the standards of Nature Communications. In particular, the novelty relative to existing organic PersL literature, the rigor of the trap analysis, and the mechanistic justification of singlet–triplet redistribution require clarification.

Recommendation: Major Revision

Here are some comments the authors are invited to address:

Response: We sincerely appreciate the review for the constructive comments and suggestions on the manuscript, which are very valuable to improve the quality and potential impact of this work. We have carefully addressed your concerns especially those regarding the novelty relative to existing organic persistent luminescence (PersL) literatures, the rigor of the trap analysis, and the mechanistic justification of singlet-triplet redistribution. We hope that these revisions are satisfactory from your perspective as well.

Comment #1: *The authors present the work as a "new way" to realize white CP-PersL via trap states. However: trap-mediated organic persistent luminescence is already well established, including by the authors themselves. Also, charge separation → trapping → thermal detrapping → recombination seems very close to earlier exciplex-based and trap-based PersL systems. The authors should explicitly and quantitatively compare their system with existing white PersL systems (organic and inorganic) and prior CP-PersL or long-lived CPL materials (especially those beyond the ms–s regime). Maybe a table can help. The main claimed novelty appears to be: white emission from single guest species via combined S_1 and T_1 emission and retention of CPL during hour-long PersL. Furthermore, the authors refer to the emission as white; however, the spectra are dominated by two discrete blue and yellow bands. Clarification on whether this should be considered warm-white rather than true broadband white would improve precision.*

Response: We thank the reviewer for the professional suggestions regarding the differences between this work and previous literatures, as well as the accuracy of the terminology. Indeed, the trap-induced organic PersL model has been established in recent publications. Nevertheless, the trap-induced model does not address complex systems involving interactions among different excited states, different chiral molecules, and trap states. This work, building on the previous model, further **advances the mechanistic understanding of organic PersL by elucidating the redistribution of detrapped charge carriers into singlet and triplet states**, thereby enables hour-long white PersL and CPL. Following your suggestion, we have added a detailed performance comparison of white PersL systems and long-lived CPL materials in **Supplementary Table 6**. The emission wavelengths, $|g_{lum}|$ values, and lifetime/decay time of reported organic white PersL and circularly

polarized persistent luminescence (CP-PersL) materials are listed in Columns 4–6. Considering that the lifetime and decay time may be defined differently in the literature, both parameters are listed in column 6 and described at the end of the table. This table clearly shows that **the materials studied in this work exhibit remarkable hour-long white PersL compared to existing materials, while maintaining moderate CPL**. It demonstrates that **introducing trap states into organic systems to populate both singlet and triplet states of luminescent molecules (a strategy first reported in this work) represents a promising design strategy for achieving white PersL**.

Regarding the description of emission color, we acknowledge that the original manuscript lacked sufficient accuracy. Accordingly, for specific materials such as R/S-PyBN@DPEPO, we have revised the description to "warm white" as their CIE chromaticity coordinates deviate from ideal white point. Since white light comprising two emission bands with different CIE coordinates can be reasonably anticipated by applying the same design strategy with different guest or host molecules, we consider the use of "white emission" in general discussions not involving specific materials. We hope to get your kind understanding.

Actions: We have added **Supplementary Table 6** into the Supplementary Information to show the different performances of reported white PersL and CP-PersL materials and included corresponding descriptions in the manuscript. Also, we have revised the description of PersL color of R/S-PyBN@DPEPO as "warm white" to improve precision.

*"Dual-band white PersL is achieved by releasing trapped electrons that populate both the singlet (S_1) and triplet (T_1) states of guest molecules characterized by a large singlet–triplet energy gap (ΔE_{ST}) (**Fig. 1b**)."* (Page 2 of the revised manuscript, left column, lines 3-6)

Supplementary Table 6 | Representative white PersL and CP-PersL materials.[a]

Molecular formula		Excitation source (nm)	Emission (nm)	$\|g_{lum}\|$ ($\times 10^{-3}$)	Lifetime (ms-h)[b] or decay time (s-h)[c]	Reference
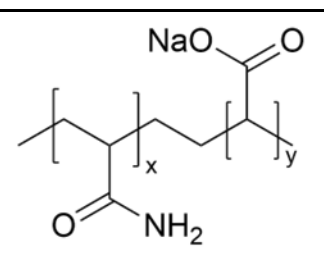 Host-1: APAM	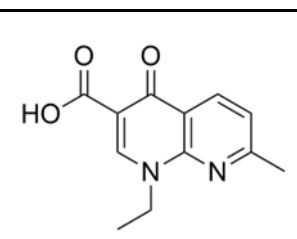 Guest-1: NDA	365	457, 567, 675	-	98.36 ms[a]	4
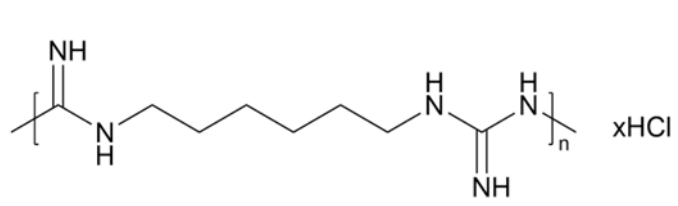 Host-2: PHMB	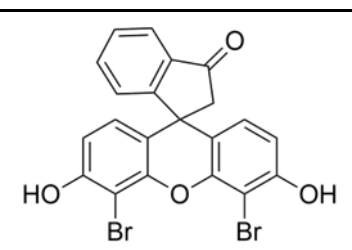 Guest-2: DBF					
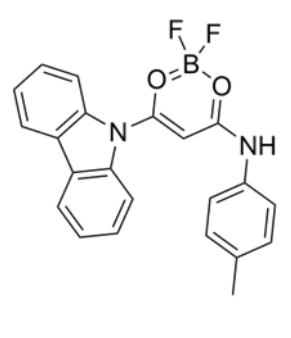 CzBMe		365	430, 460, 535	-	0.61 s[a]	5
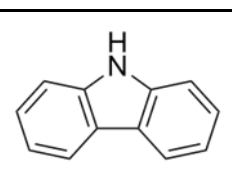 Host: Cz	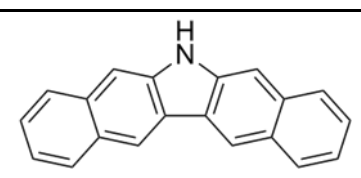 Guest: DBCz	365	458, 550 (40/60°C)	-	632 ms[a]	6
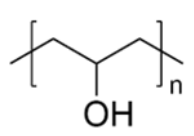 Host: PVA	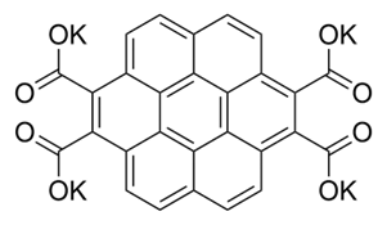 Guest-1: CS	346	400-700	-	~2.6 s[a]	7

	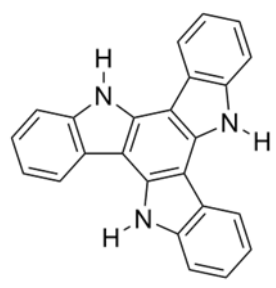 Guest-2: TAT					
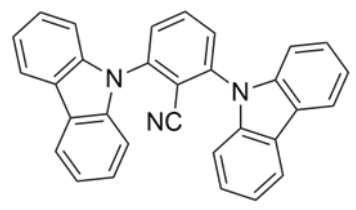 DCBN		365	460, 544	-	964.3 ms[a]	8
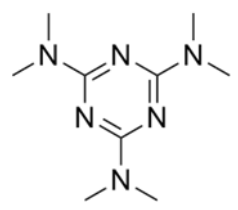 Host: MF	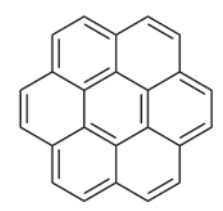 Guest: COR	365	448, 568 (370 K)	-	4.83 s[a]	9
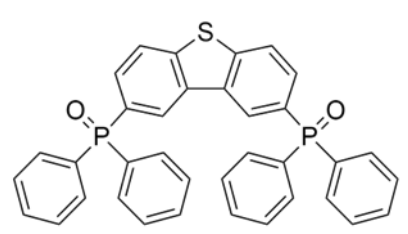 Host: PPT	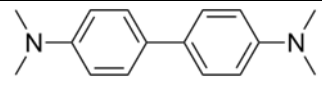 Guest-1: TMB	355	522, 497, 665	-	92 s[b]	10
	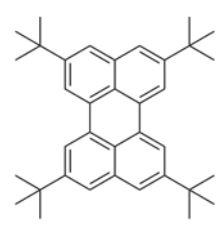 Guest-2: TBPe					
	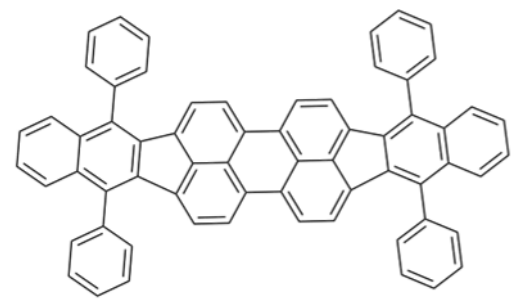 Guest-3: DBP					
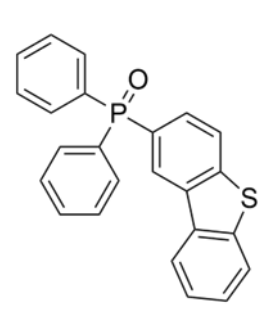 Host: DBTSPO	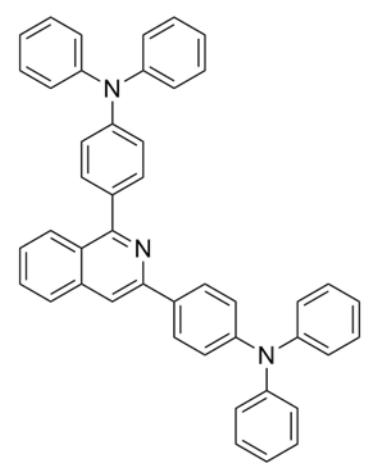 Guest: KZLDTPA	410	465, 577	-	112.3 ms[a] / 40 min[b]	11
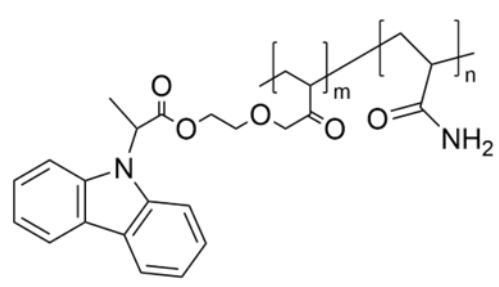 Host: R/S-PAMCOOCz$_x$	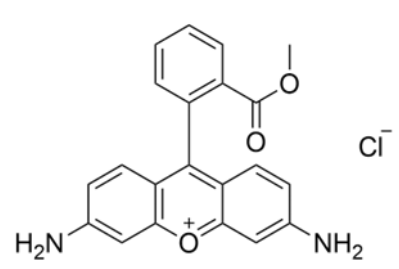 Guest: Rh123	299	414, 442, 470, 574	5.5	1.9 s[a]	12
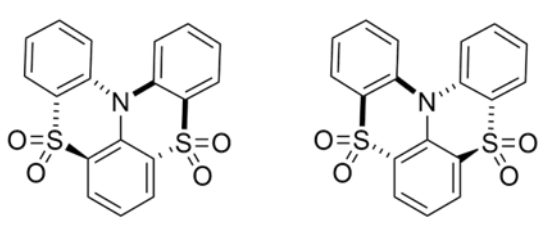 (+)-(P)/(-)-(M)-BTPT		-	458	7.8	13.7 ms[a]	13

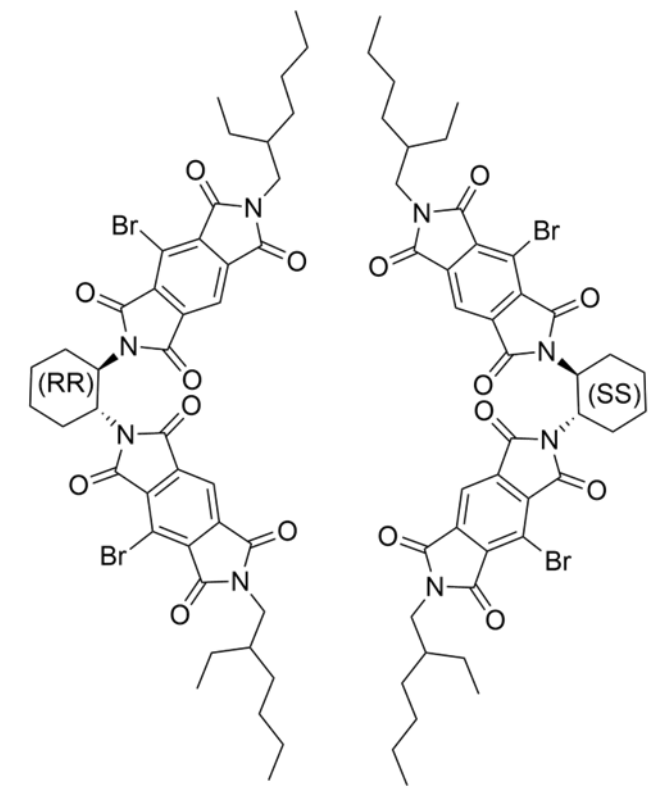<br>(RR)/(SS)-BrPmDI	310	500	1.3	31.5 ms[a]	14
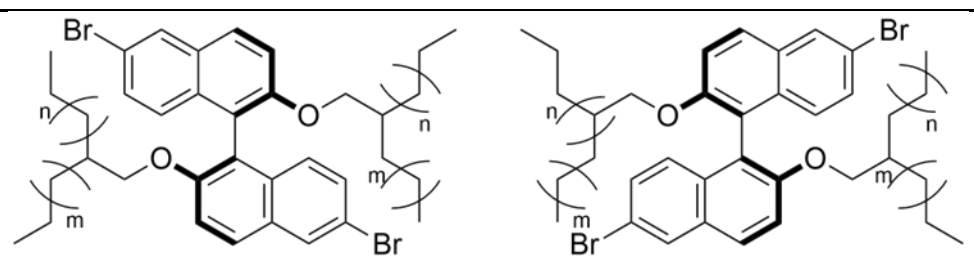<br>(R/S)-PBNA	290	551, 520	9.4	34 ms[a]	15
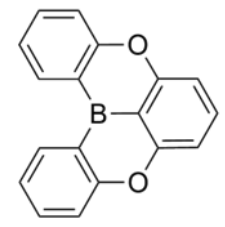<br>Host: DOBA<br>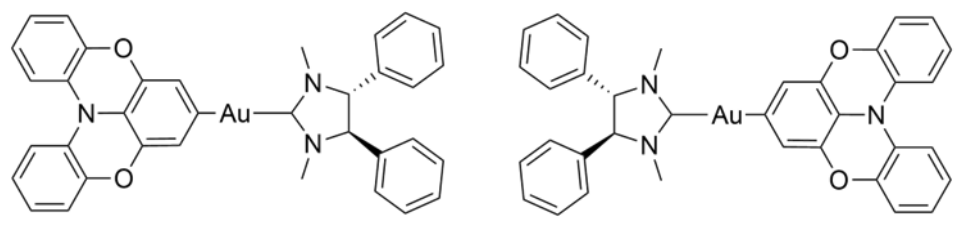<br>Guest: R/S-DPXZAu	340	492	1.4	42 ms[a]	16
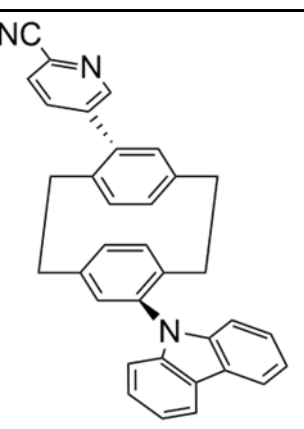<br>(+)-RP/(-)-SP-PCP-PyCNCz	-	550	7.2	528 ms[a]	17
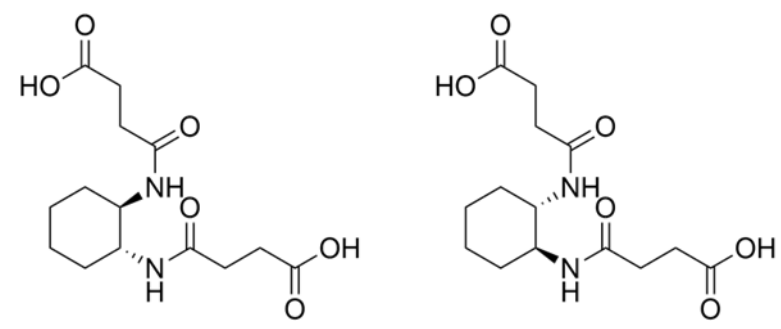<br>(R, R)/(S, S)-DAACH	240-360	470-530	2.3	587 ms[a]	18
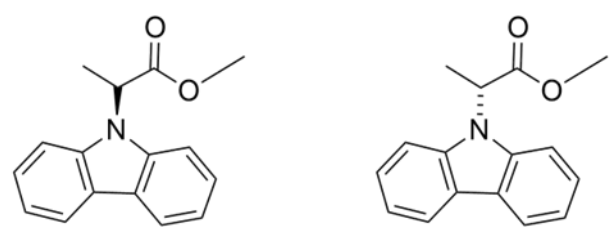<br>R/S-COOCz	365	550, 596	2.3	600 ms[a]	19
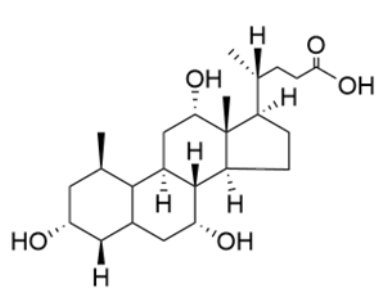<br>Host: CLA<br>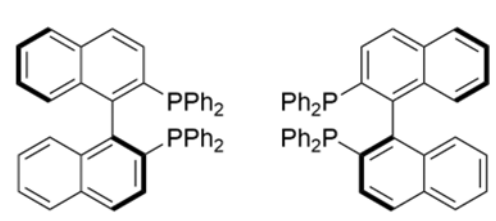<br>Guest-1: R/S-BINAP	300	502	3.5	720 ms[a]	20

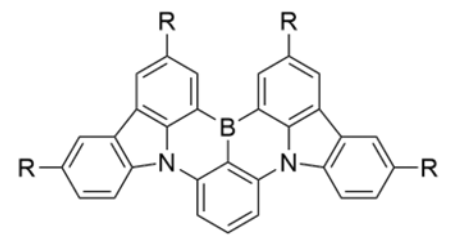<br>Guest-2: BCzBN, R=$C(CH)_3$						
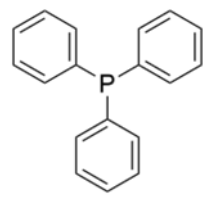<br>Host-1: TPP<br>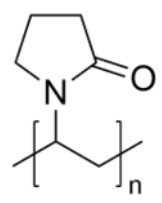<br>Host-2: PVP	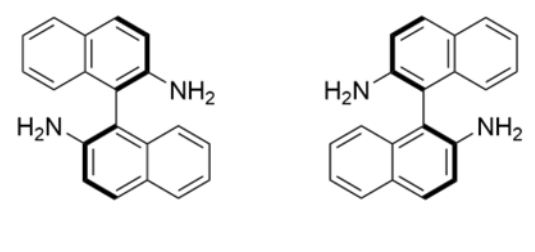<br>Guest: R/S-NA	365	535	0.9	780.5 ms[a]	21
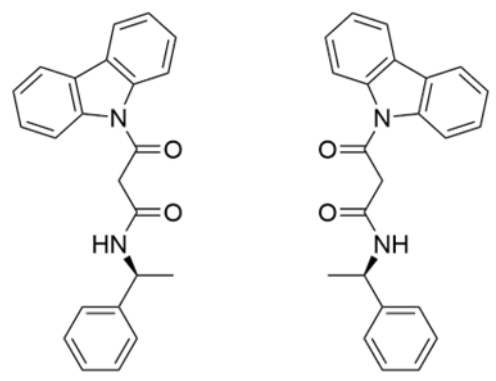<br>R/S-CzCOPEA		300	365, 385, 408, 528, 574, 624	5.6	946.44 ms[a]	22
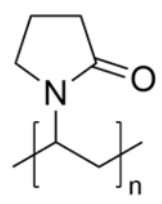<br>Host: PVP	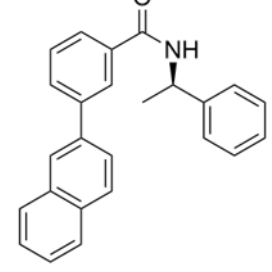<br>Guest-1: Na-R/S	300	514	2.1	601 ms[a]	23
	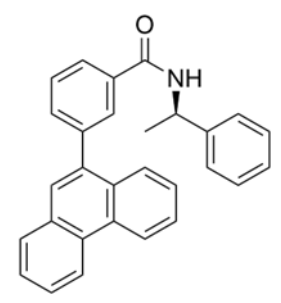<br>Guest-2: Pa-R/S	360	533	2.2	1017 ms[a]	
	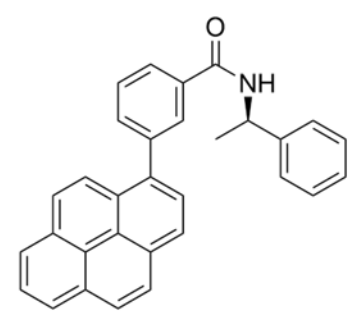<br>Guest-3: Py-R/S	380	612	2.5	341 ms[a]	
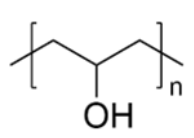<br>Host: PVA	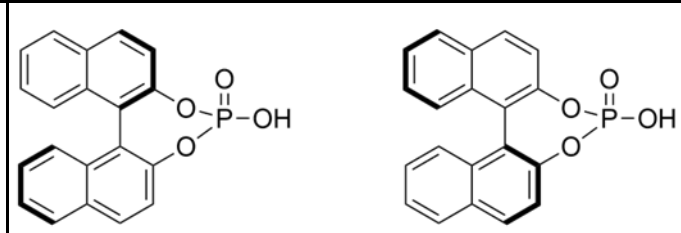<br>Guest: R/S-BNDHP	312	501, 534	6.89	1025.5 ms[a]	24
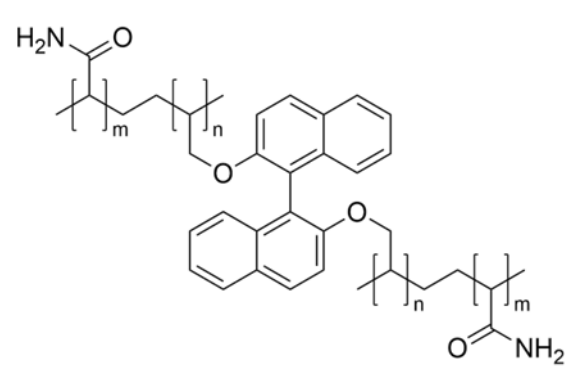<br>R/S-PAMBNAX		254	545	10.6	1078.44 ms[a]	25

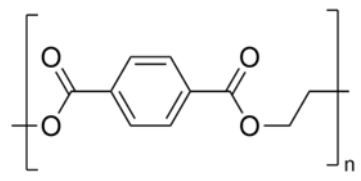<br>Host: PET<br>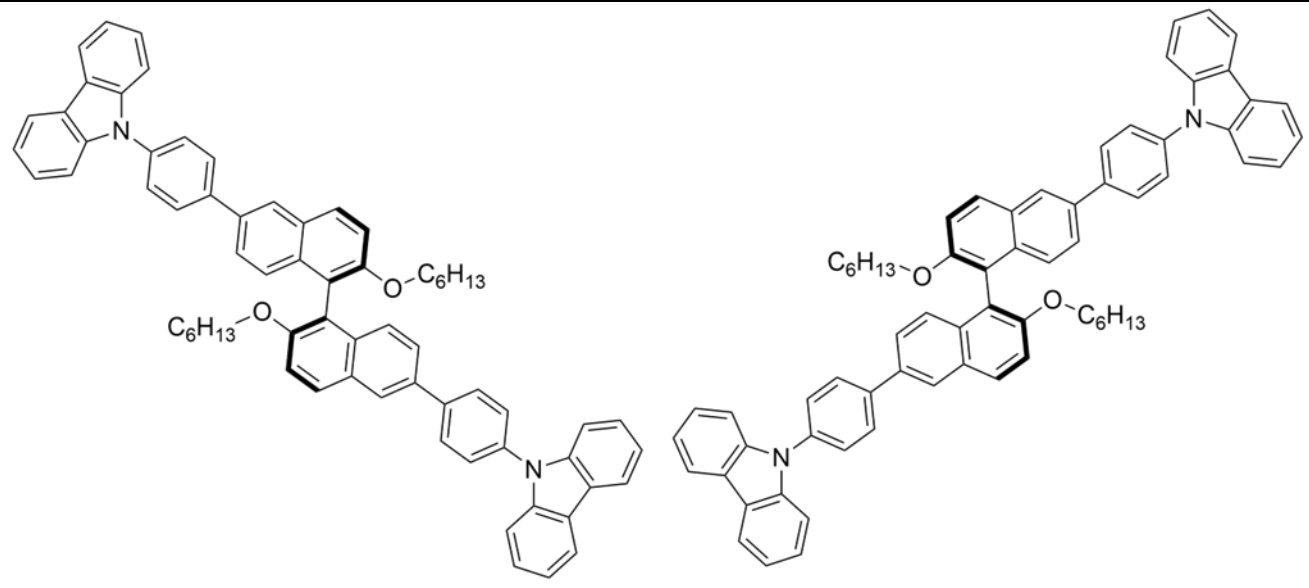<br>Guest: R/S-BINAC	365	580	0.61	2960 ms[a] / > 1 h[b]	26
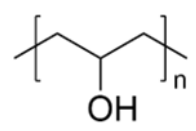<br>Host: PVA<br>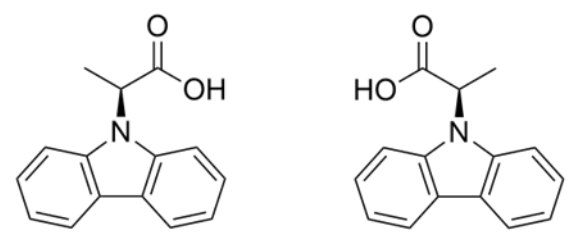<br>Guest: R/S-COOHCz	300	416, 444	50	3.3 s[a]	27
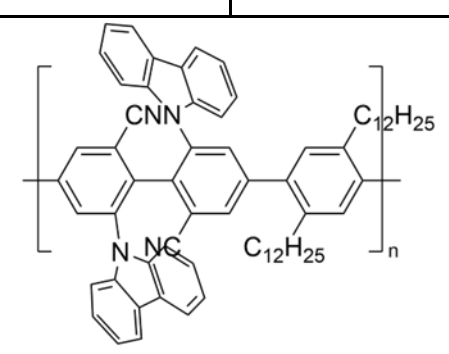<br>R/S-P12-BCzBCN	365	489	5.2	33 s[a] (at 77 K)	28
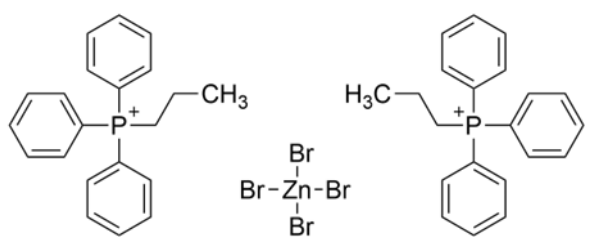<br>Host: $(PTP)_2ZnBr_4$<br>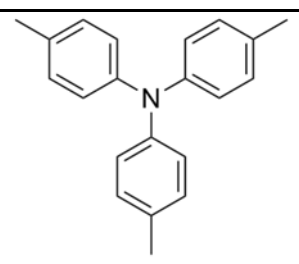<br>Guest-1: R1<br>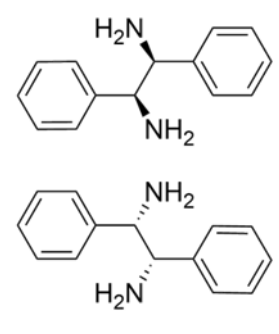<br>Guest-2: (R,R)/(S,S)	395	510	11	> 1 h[b]	29
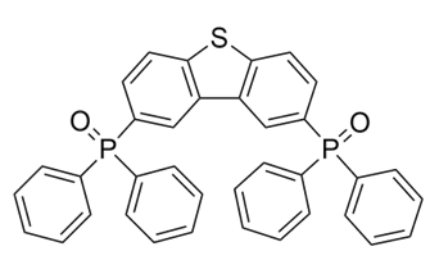<br>Host: PPT<br>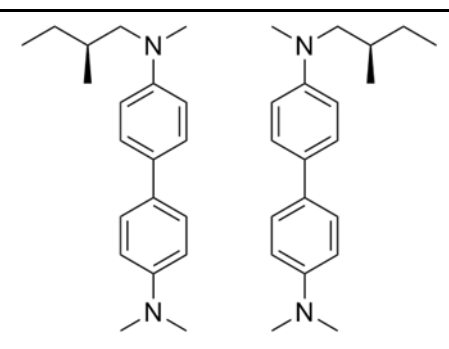<br>Guest-1: R/S-TBTMB	365	525	4.5	> 1.5 h[b]	30
Host: PPT<br>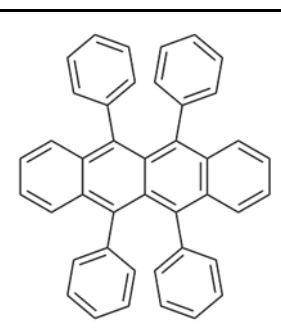<br>Guest-1+Guest-2: Rubrene	365	600	2.3	> 1 h[b]	30

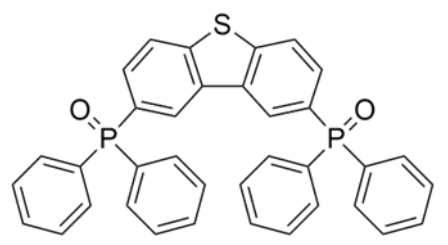<br>Host-1: PPT	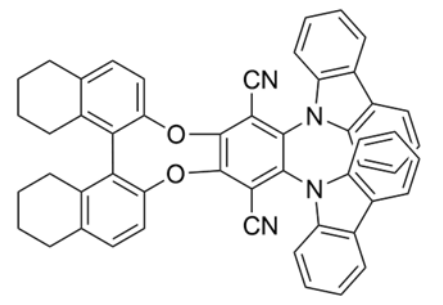<br>Guest-1: R/S-OBN-Cz<br>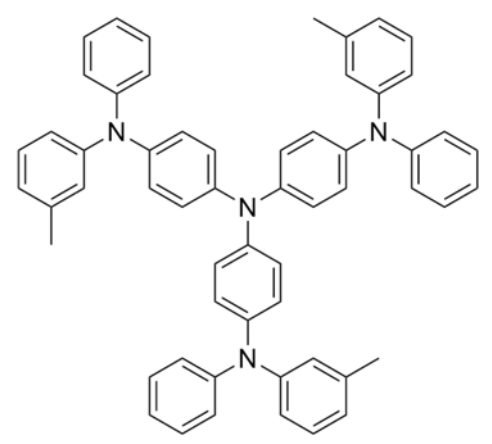<br>Guest-2: m-MTDATA	365	520	0.28	> 2 h[b]	31
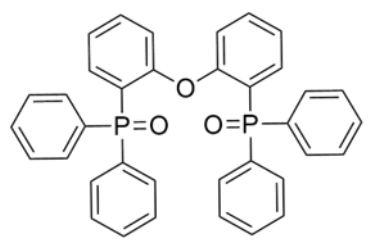<br>Host-1: DPEPO<br>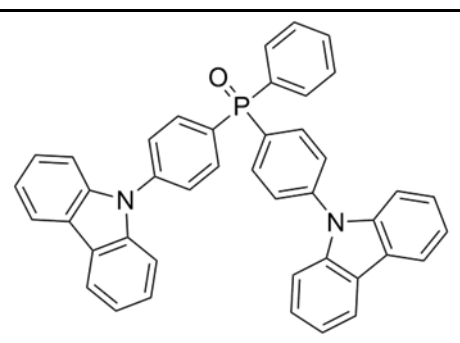<br>Host-2: BCPO<br>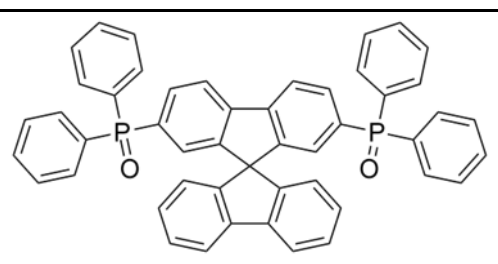<br>Host-3: SPPO13	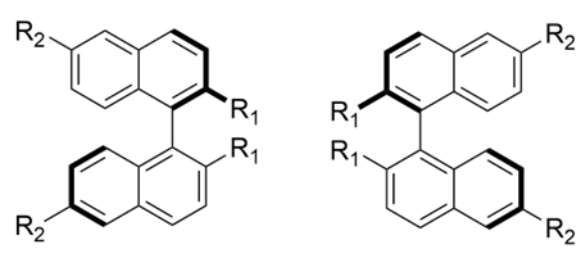<br>Guest:<br>R/S-PyBN (R_1 = C_4H_9O, R_2 = C_5H_5N)<br>R/S-APyBN (R_1 = C_4H_9O, R_2 = C_7H_5N)<br>R/S-PyBNMO (R_1 = $C_2H_5O_2$, R_2 = C_5H_5N)	365	390-700	0.82-2.3	0.75-4.78 h[b] (R/S-PyBN: 762.13 ms[a])	This work

[a]The selected parameters are the best values reported in the references.
[b]Measured by using a time-correlated single photon counting method (lifetime).
[c]Measured by using a photomultiplier tube (decay time).

Comment #2: *The persistent luminescence decay curves are presented without any fitting (e.g., graphs do not show fit curves) or extraction of kinetic parameters. Given that the proposed mechanism relies on trap-controlled recombination and multiple emissive channels, quantitative decay analysis would be necessary to support the interpretation.*

Response: We appreciate the reviewer's insightful comment regarding the PersL decay curves. We fully agree that decay kinetics analysis is essential for validating the trap-induced PersL model. Following your suggestion, we have now fitted the PersL decay curves of all composite films shown in **Fig. 2c** and **Supplementary Fig. 24**.

As shown in the log-log plots, the decay curves exhibit two distinct kinetic regimes. During the initial stage (0 to several seconds), the intensity undergoes rapid decay governed by an exponential decay function. Subsequently, the decay transforms into **an inverse power-law dependence on time ($I \sim t^{-m}$)**. However, a single power-law function does not provide a satisfactory fit across the entire time range. Therefore, we have adopted a piecewise power-law fitting approach to analyze the decay curves.

According to the Debye–Edwards law, power-law decay behavior with *m* in the range from 0.1-1.5 is characteristic of long-range charge separation and recombination following excitation, thereby **supporting the trap-controlled PersL model** (*Nature* **2017**, *550*, 384; *Nat. Commun.* **2020**, *11*, 191; *J. Chem. Phys.* **1952**, *20*, 236). As trap depths could be distributed over a certain range, the detrapping and recombination kinetics vary across different time windows, yielding

distinct *m* values (*Phys. Rev. B* **2013**, *87*, 045126; *Adv. Mater.* **2024**, *36*, 2314083).

Actions: We have added the fitted curves and the fitting values of *m* into **Fig. 2c** and **Supplementary Fig. 24**. Also, we have incorporated the relevant discussion in the revised manuscript as follows.

"*The emission intensity followed an exponential decay in the first several seconds and then obeyed an inverse power-law dependence on decay time ($I = A \cdot t^{-m}$ or $lgI = lgA - m \cdot lgt$). Such power-law decay ($m = 0.1$-2) is explained as the long-range charge separation and recombination process according to the Debye-Edwards law*[36,39,64]." (Page 4 of the revised manuscript, right column, lines 1-6)

"*The PersL decay curves all followed the Debye-Edwards power-law relationship.*" (Page 6 of the revised manuscript, right column, lines 7-8)

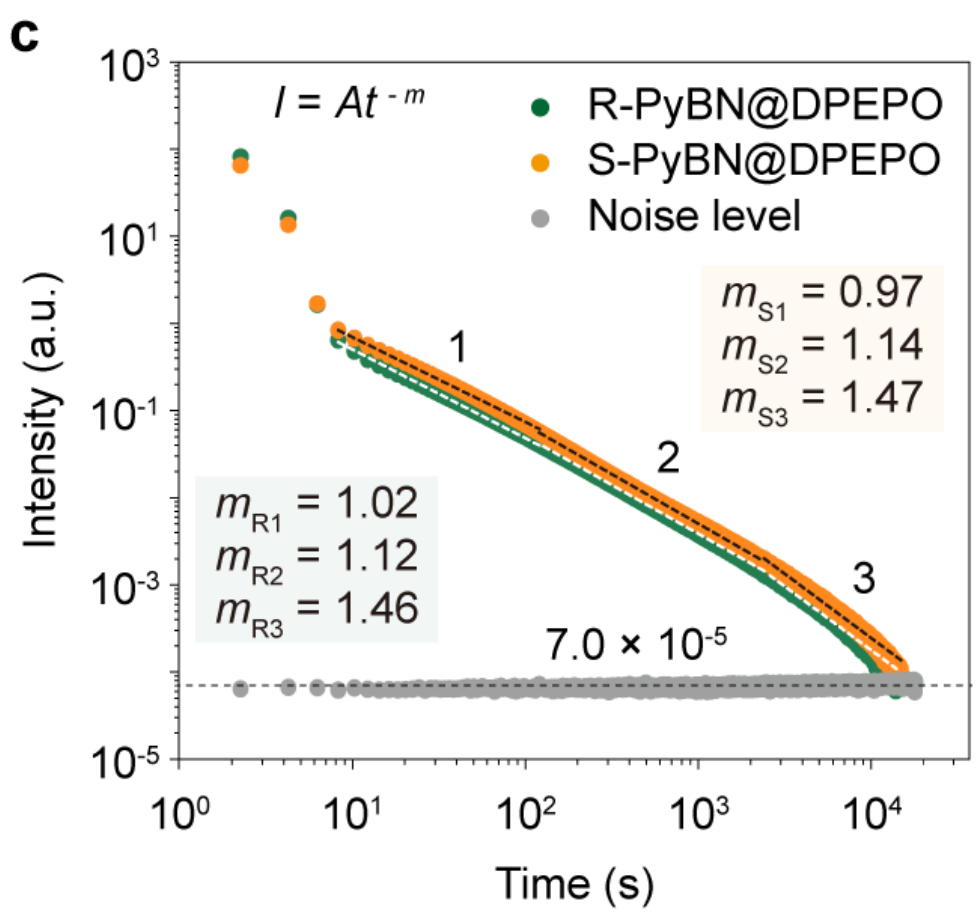

Figure 2c | PersL decay curves at RT after UV irradiation for 60 s. The noise level of the detector is also given in grey dots.

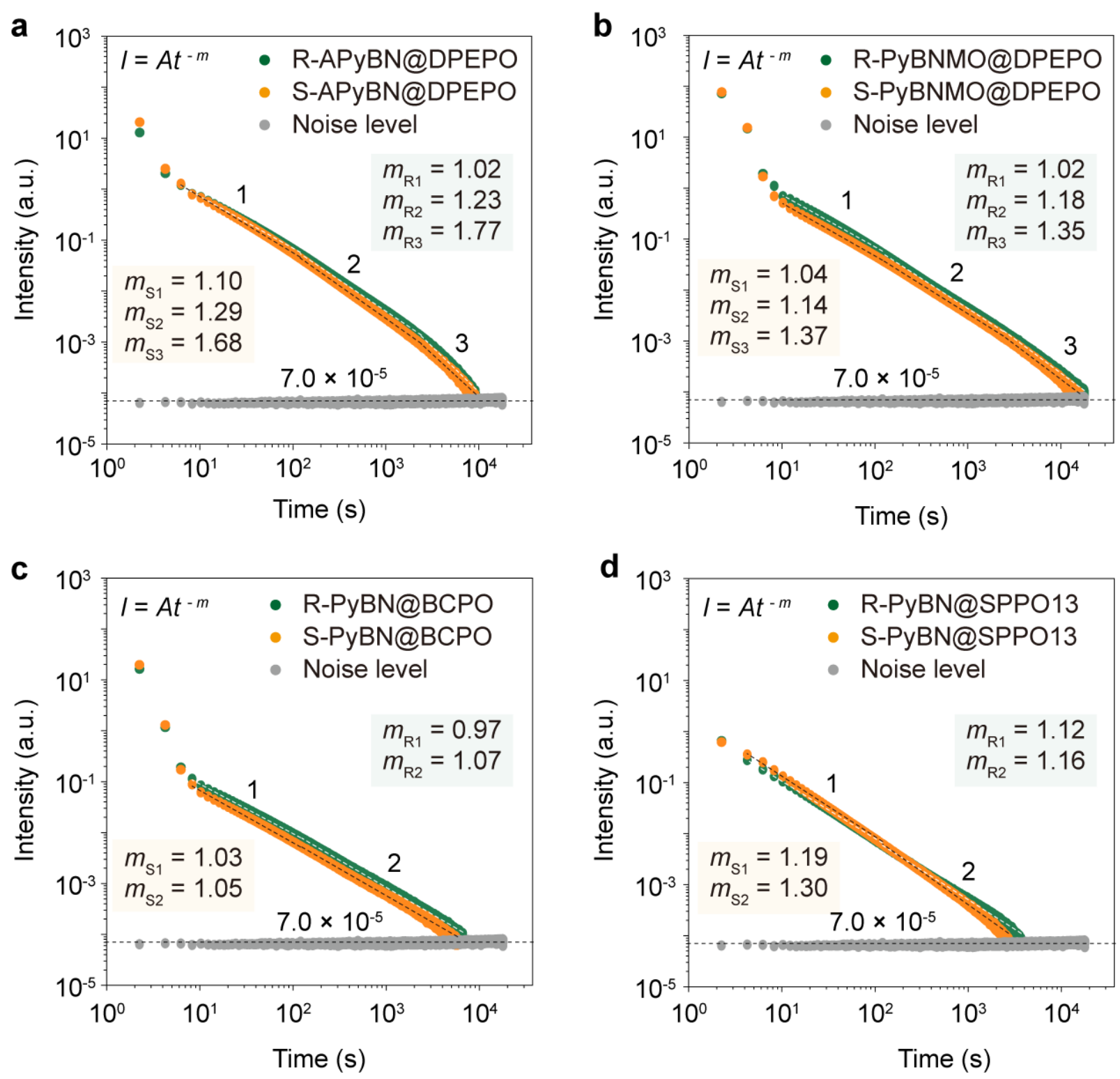

Supplementary Figure 24 | Log-log plot of PersL decay curves of R/S-APyBN@DPEPO (a), R/S-PyBNMO@DPEPO (b),

R/S-PyBN@BCPO (c) and R/S-PyBN@SPPO13 (d) under 365 nm excitation. The excitation temperature and recoding temperature were RT. The excitation time was 60 s.

Comment #3: *The manuscript claims that detrapped electrons redistribute between singlet and triplet excited states, leading to white emission. What direct experimental evidence supports this redistribution? In particular, can the authors provide wavelength-resolved decay curves or time-resolved spectra distinguishing singlet and triplet contributions during PersL?*

Response: We are grateful to the reviewer for the constructive comments on detrapped electrons distribution. Following this suggestion, we have provided the time-resolved spectra of R/S-PyBN@DPEPO in **Supplementary Fig. 14** to exhibit the evolution of singlet and triplet contribution to emission.

As shown in **Supplementary Fig. 14**, the spectra are dominated by triplet emissions both from 1 to 15 ms (after cessation of short-pulse excitation) and from 1 to 5 s (after continuous excitation). **Only after 5 s (from 5 s to 60 min) does the relative contribution of singlet states increases substantially, reaching an intensity comparable to that of triplet emissions.** The time-resolved spectra clearly reveal that the afterglow emissions include phosphorescence and PersL components. **The phosphorescence component lasts for several seconds and arises exclusively from the triplet emission**, whereas **the PersL with weaker intensity and much longer durations encompasses contributions from both triplet and singlet states**. The time scales are consistent with the two-stage decay behavior shown in **Fig. 2c**. Therefore, unlike phosphorescence, which originates from singlet-to-triplet intersystem crossing (ISC), PersL arises from the redistribution of detrapped electrons into singlet and triplet states. We believe this may provide an alternative viewpoint for investigating the charge carrier migration in organic luminescent materials.

Actions: We have provided time-resolved spectra in **Supplementary Fig. 14** and added clarification regarding the detrapped electrons redistribution in the revised manuscript as follows.

*"Notably, the time-resolved spectra revealed the evolution of singlet and triplet contribution in the entire emission processes (**Fig. 2d** and **Supplementary Fig. 14**). The emission spectra recorded in the range of 1 ms to 5 s were predominantly governed by the triplet states. In contrast, the relative contribution of singlet states increased substantially after 5 s (from 5 s to 60 min), reaching an intensity comparable to that of triplet emissions. These results indicate that, in the PersL process electrons are populated into the S_1 and T_1 states, causing both singlet-singlet and triplet–singlet transitions to generate long-lasting PersL."* (Page 4 of the revised manuscript, right column, lines 8-15)

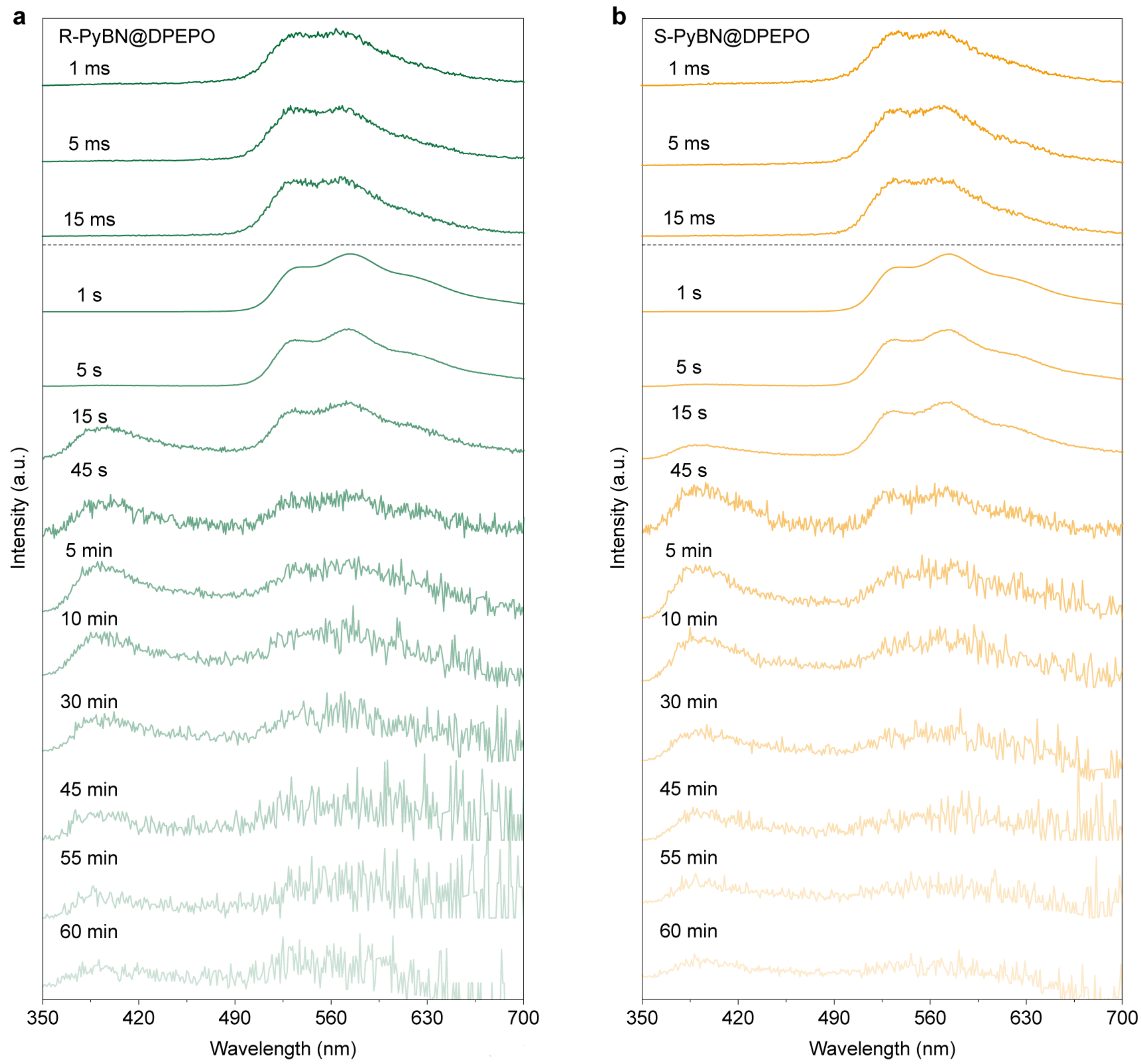

Supplementary Figure 14 | Time-resolved emission spectra of R-PyBN@DPEPO (a) and S-PyBN@DPEPO (b) films. The spectra recorded at 1, 5, and 15 ms were obtained after cessation of 355 nm short-pulse excitation, whereas the spectra from 1 s to 60 min were collected after 1 min of continuous 365 nm excitation.

Comment #4: *The agreement between DFT-derived LUMO offsets and experimental trap depths is presented as strong validation of the model. However, the averaging of α and β spin orbital energies is not rigorously justified. Typically, DFT (with Gaussian) results provide qualitative support for open-shell species and solid-state environments. Quantitative support in these cases is questionable, although the provided numbers match well with the experiments. Explicit discussion of limitations is necessary.*

Response: We sincerely thank the reviewer for raising this important point. Indeed, DFT (with Gaussian) calculations provide molecular orbital energies for open-shell molecules in discrete α and β spin states. Consequently, comparing the LUMO energy levels between the two molecular systems yields **four different values** for the energy gap. By contrast, thermoluminescence (TL) estimation based on the Randall–Wilkins model (peak method) yield **a single value**, which is statistically the most probable energy gap for the macroscopic sample (comprising a large number of molecules). This discrepancy necessitates a mathematical treatment of the DFT results. Under thermal equilibrium, the two spin orientations are equally populated. Therefore, we employed a simplified arithmetic averaging approach to obtain a single energy level difference. Admittedly, as the reviewer mentioned, DFT calculations are inherently qualitative, and the

simplified treatment inevitably introduces limitations when correlating these computational results with TL data. We have therefore discussed in the revised manuscript the rationale for this simplification and the limitation of the model.

Actions: In accordance with your constructive suggestion, we have revised the statement on the rationale for the simplification and the limitation of the model in the manuscript as follows.

"The DFT calculations were performed on simplified models of isolated molecules in vacuum." (Page 9 of the revised manuscript, left column, lines 21-22)

"For the open-shell radical anions, the discrete α and β spin molecular orbitals resulted in four different LUMO energy differences between the two molecular systems. To compare with the single trap-depth value estimated from TL measurements for the macroscopic sample (composed of a large number of molecules), we adopted a simplified arithmetic averaging approach to obtain a single energy-level difference. However, it should be noted that such simplification represents the overall trend and cannot fully represent the actual solid-state host-guest environment. Furthermore, the DFT-derived energy levels values could be also limited by the choice of functionals and basis sets. The DFT calculations should be regarded as a qualitative evaluation method of the trap depth rather than the quantitative support." (Page 9 of the revised manuscript, left column, lines 25-38)

Comment #5: *The melt-casting method at 320 °C raises concerns about thermal degradation, which are not discussed.*

Response #5: We thank the reviewer for the concern on thermal degradation during sample preparation. According to your comment, we have performed thermogravimetric analysis (TGA) on R/S-PyBN, DPEPO, and R/S-PyBN@DPEPO. As shown in **Supplementary Fig. 9**, the decomposition temperatures (T_d, 10% weight loss) for R-PyBN, S-PyBN and DPEPO are 337°C, 329°C, and 380°C, respectively. After blending R/S-PyBN with DPEPO at a mass ratio of 1:99 under a nitrogen atmosphere, the decomposition temperatures of R-PyBN@DPEPO and S-PyBN@DPEPO composite films were 370°C and 378°C, respectively (exceeding 320°C). The preparation temperature would not cause significant thermal degradation on the samples. In addition, the preparation temperatures for the pure host (DPEPO) and guest (R/S-PyBN) films were 300 °C and 230°C respectively, and both were within the safe temperature range without degradation risks.

Actions: We have added the following text and **Supplementary Fig. 9** into the manuscript and Supplementary Information to discuss the potential risks associated with the preparation temperatures.

*"Thermogravimetric analysis (TGA) demonstrated that the R/S-PyBN@DPEPO films (1 wt%, unless otherwise specified) did not undergo obvious thermal decomposition under a nitrogen atmosphere, with their decomposition temperatures (T_d, 10% weight loss) of 370/378°C, exceeding the preparation temperature of 320°C (**Supplementary Fig. 9**)."* (Page 3 of the revised manuscript, left column, lines 8-9, and right column, lines 1-4)

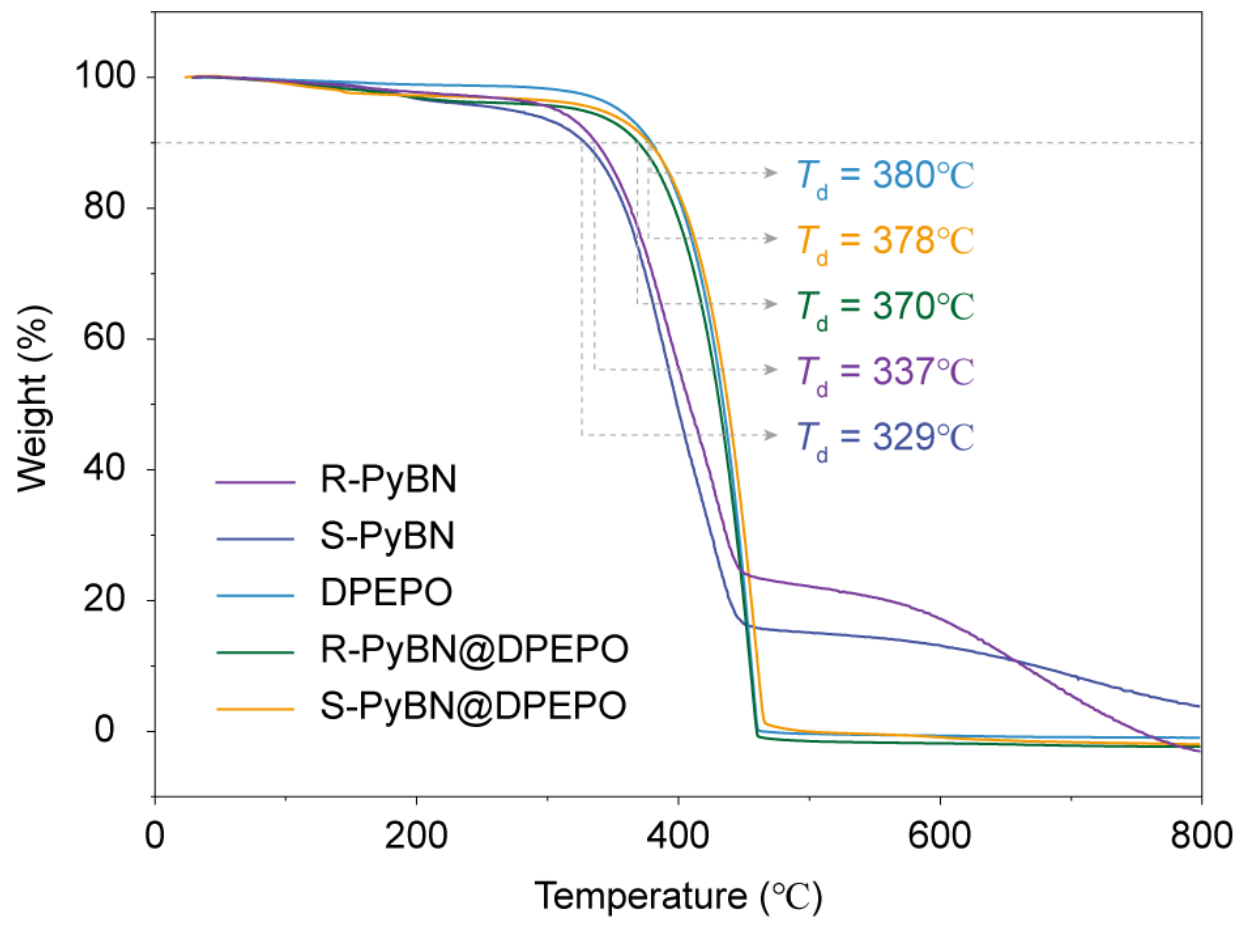

Supplementary Figure 9 | Thermogravimetric analysis (TGA) curves of R/S-PyBN, DPEPO, and R/S-PyBN@DPEPO under a nitrogen atmosphere.

Comment #6: *Lifetime decay profiles of Figure S10 are characterized by low signal-to-noise ratios after a few ms, with data points scattered within an order of magnitude interval (in intensity). These should be improved if the authors have considered these points in the fitting procedure.*

Response: We thank the reviewer for the suggestion on lifetime decay profiles. We apologize for the issues with data quality and fitting mistake in the previous submission. Following your suggestion, we have remeasured the prompt lifetime decay curves using the same samples and conditions and refitted the data. The corresponding decay profiles have now been provided in **Supplementary Fig. 4**. The fitting parameters are summarized in **Supplementary Table 1**. We hope that these revisions may improve the accuracy and reliability of the data analysis.

Actions: We have updated the transient lifetime decay curves of the R/S-PyBN films in the revised Supplementary Information.

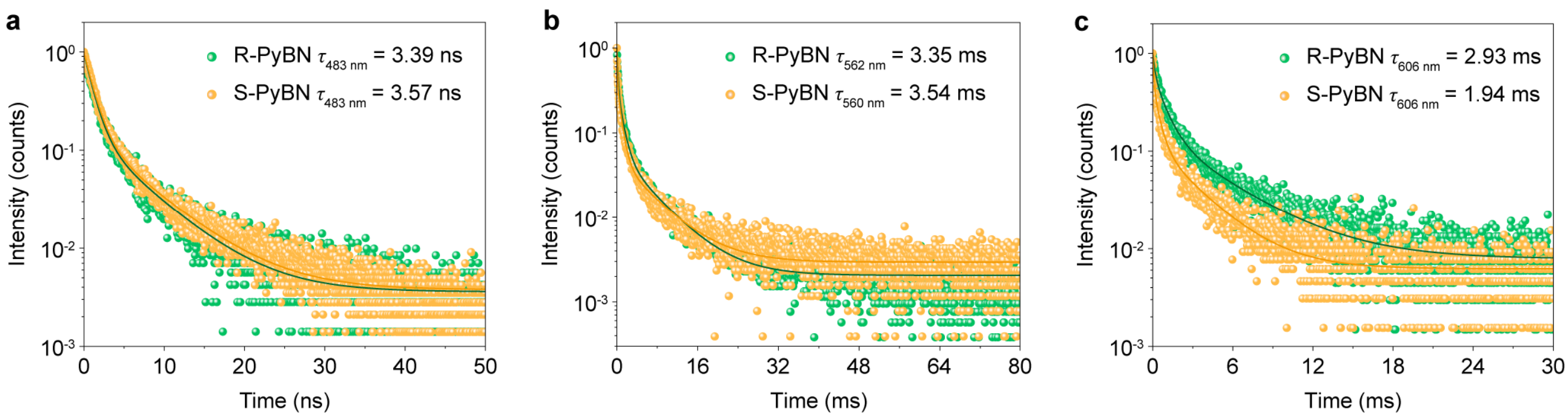

Supplementary Figure 4 | Normalized fluorescence (prompt) lifetime decay curves of the emission bands at 483/483 nm (a), 562/560 nm (b) and 606/606 nm (c) for R/S-PyBN films at RT.

Supplementary Table 1 | Fitted fluorescence and phosphorescence lifetimes of R/S-PyBN in neat films.

Film	Wavelength (nm)	Condition	Fluorescence					Phosphorescence						
			τ_1 (ns)	A_1 (%)	τ_2 (ns)	A_2 (%)	τ^a (ns)	τ_1 (ms)	A_1 (%)	τ_2 (ms)	A_2 (%)	τ_3 (ms)	A_3 (%)	τ^a (ms)
**R-PyBN**	483	RT	1.09	50.63	5.74	49.37	3.39	-	-	-	-	-	-	-
	562	RT	-	-	-	-	-	0.19	15.13	0.99	37.54	6.24	47.33	3.35
	606	RT	-	-	-	-	-	0.17	5.55	0.86	35.67	4.44	58.78	2.93
**S-PyBN**	483	RT	1.16	51.49	6.12	48.51	3.57	-	-	-	-	-	-	-
	560	RT	-	-	-	-	-	0.82	40.12	0.11	10.43	6.47	49.46	3.54
	606	RT	-	-	-	-	-	0.08	7.91	0.47	31.56	2.95	60.53	1.94

[a]Determined from the fitting function of $I = \sum A_i e^{-t/\tau_i}$ according to lifetime decay profiles, where A_i is the exponential factor for lifetime τ_i. The average lifetimes of fluorescence/phosphorescence are calculated by $\tau = \sum A_i \tau_i$.

Comment #7: *It is not clear if the structured phosphorescent component in R/S-PyBN is only observable at 77 K. If so, how did the authors measure the decay at RT? Is the structured phosphorescent component present at RT only in the film?*

Response: We thank the reviewer once again for the constructive comment on the photophysical properties of R/S-PyBN.

To address this point, we have measured the fluorescence (Flu.) and phosphorescence (Phos.) spectra of R/S-PyBN in three distinct forms (melt-casting films, powders, and R/S-PyBN@PMMA) at RT and 77 K (**Supplementary Figs. 2, 3, 12**). The results reveal that the structured phosphorescence of R/S-PyBN in the film form was indeed observable only at low temperatures (77 K). At RT, these triplet emissions became broadened and yielded a broad emission band. Meanwhile, the intensity of this broad band was relatively weak owing to the enhanced non-radiative relaxation. Therefore, although **measuring the decays at 560 or 606 nm at RT was feasible, the signal-to-noise ratio was quite low** (see the response to **Comment #6** and **Supplementary Fig. 4**).

For the powder form, the structured phosphorescence could be detected at 77 K but became too weak to be resolved at RT (**Supplementary Fig. 2**). This is likely due to the looser packing in the powder state, which facilitates more pronounced molecular motions and more serious quenching of the triplet emissions.

On the other hand, similar phosphorescence as that in melt-casting film was observed in 1wt% R/S-PyBN@PMMA at both 77 K and RT (**Supplementary Fig. 12**). This not only confirms that the triplet emissions in the composite system originates from the guest molecules, but also demonstrates that an appropriate host matrix can effectively suppress non-radiative pathways of triplet emissions. We hope that the above data, incorporated in response to the reviewer's suggestions, make the discussion of the photophysical properties of R/S-PyBN more complete.

Actions: We have added **Supplementary Figs. 2, 3, 12** in the Supplementary Information and revised the following texts in the manuscript.

*"For R/S-PyBN, blue fluorescence emission was observed in both the toluene solution (5×10^{-5} mol/L), powder and neat film (**Supplementary Figs. 1-3**). Compared with the well-resolved structured emission of isolated molecules in toluene solution, the fluorescence of R/S-PyBN in powders and films featured a broad emission band with a pronounced red shift, which could be attributed to the complex aggregation forms with strong intermolecular charge transfer[56,57]. Meanwhile, the phosphorescence spectra of R/S-PyBN in both the powder and film forms displayed clear vibronic fine structures at 77 K, which are characteristics of radiative transitions from the local excited state (LE) excitons[58,59]."* (Page 2 of the revised manuscript, left column, line 23, and right column, lines 1-10)

*"Similar phosphorescence features were also observed in 1 wt% R/S-PyBN@PMMA, further supporting that the emission remained dominated by the guest molecules (**Supplementary Fig. 12**)."* (Page 4 of the revised manuscript, left column, lines 11-14)

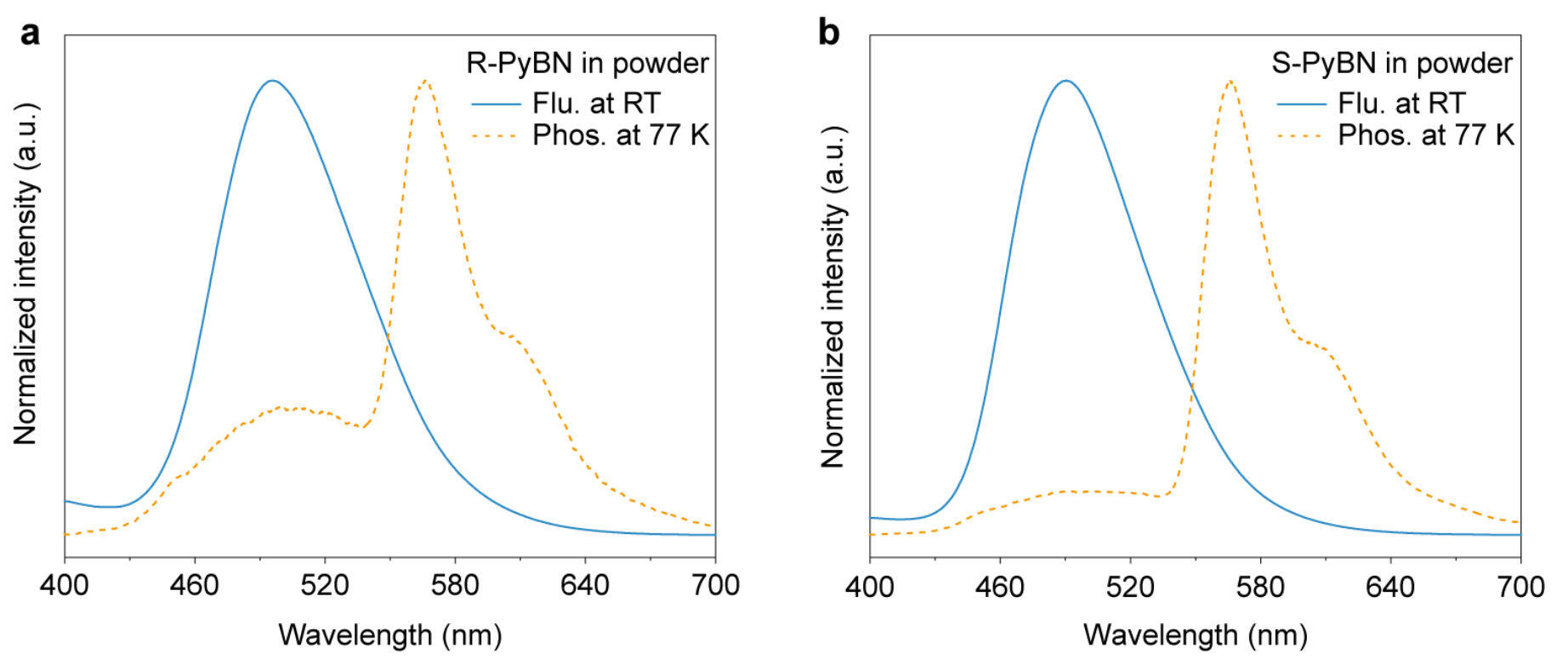

Supplementary Figure 2 | Fluorescence (Flu., at RT) and phosphorescence (Phos., delayed time 8 ms, at 77 K) spectra of R/S-PyBN in powder form. a, S-PyBN. b, S-PyBN.

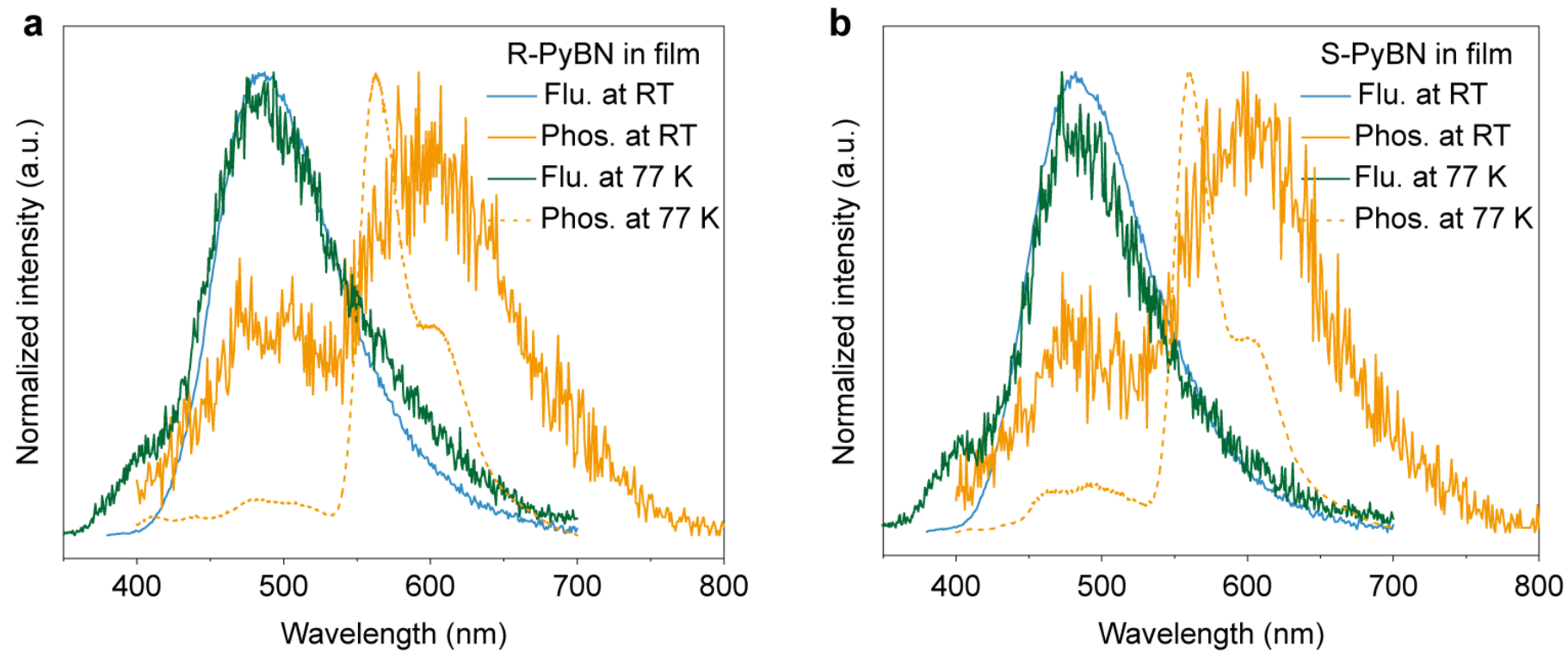

Supplementary Figure 3 | Fluorescence (Flu.) and phosphorescence (Phos.) spectra of R-PyBN (a) and S-PyBN (b) in neat film. The delayed time of phosphorescence was 100 μs at RT and 8 ms at 77 K.

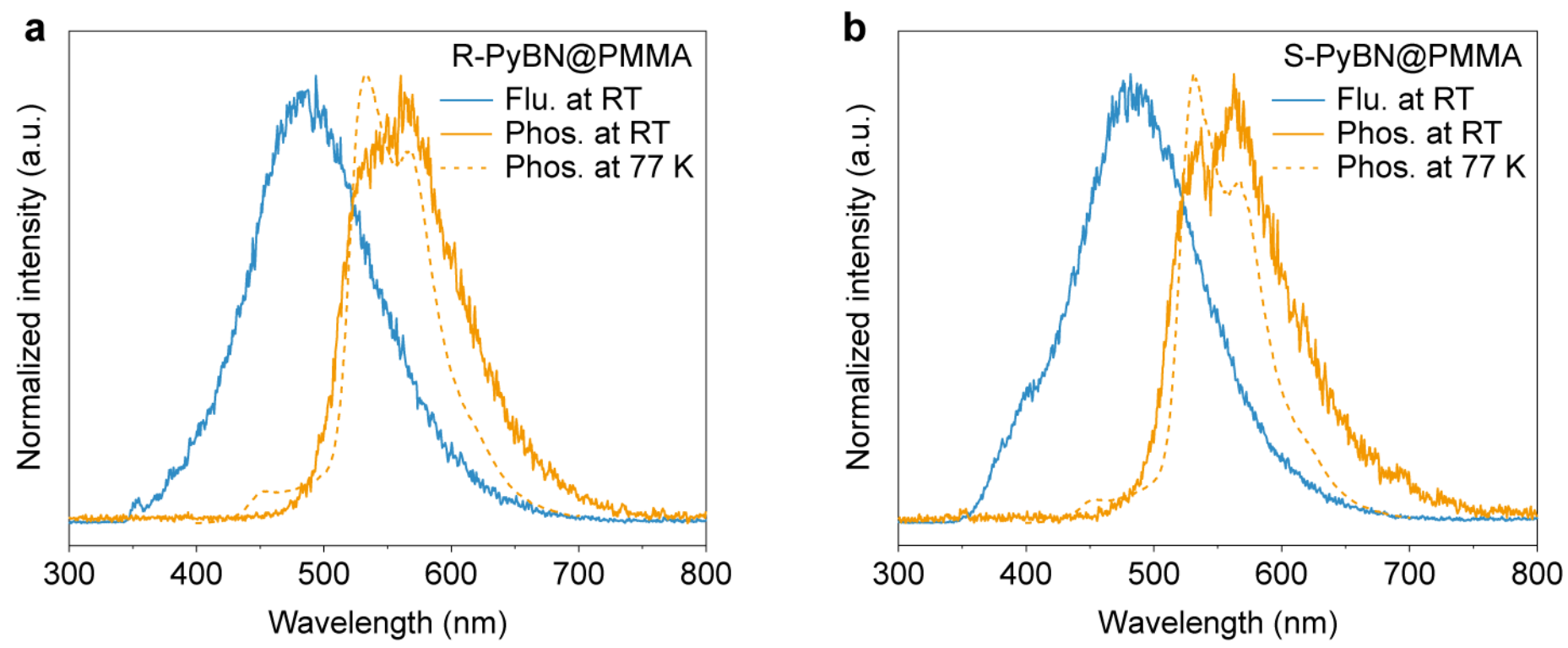

Supplementary Figure 12 | Fluorescence (Flu.) and phosphorescence (Phos.) spectra of 1 wt% R-PyBN@PMMA (a) and S-PyBN@PMMA (b) films. The delayed time of phosphorescence was 5 ms at RT and 8 ms at 77 K.

Comment #8: *Encryption demonstration is visually appealing but conceptually incremental, as similar demonstrations exist; this should be framed as a proof-of-concept rather than a major advance.*

Response and Actions: We are grateful to the reviewer for this constructive suggestion. We fully agree that translating the CP-PersL phenomenon from a concept to applications remains challenging at current stage, and the encryption demonstration presented in the manuscript should be regarded as a proof of concept. We have revised the relevant descriptions throughout the manuscript accordingly. We believe this revision improves the accuracy of the statements.

"Leveraging these characteristics, we present a proof-of-concept demonstration of warm-white circularly polarized persistent luminescence materials for Morse-code encryption and multimodal information storage, indicating the potential of organic luminescence materials in the next-generation optical information technologies." (Page 1 of the revised manuscript, left column, lines 16-21)

*"Considering the unique properties of warm-white PersL combined with CPL in the synthesized materials, we further conducted a proof-of-concept experiment on optical information encryption (**Fig. 5**)."* (Page 8 of the revised manuscript, left column, lines 2-4)

Comment #9: *The authors said "the electron is mobile within host molecules, and then captured by another guest to generate (R/S-PyBN)•−*". Considering a 1% "doping" in the film, is it reasonable to also consider an intramolecular process?*

Response: We appreciate the reviewer's valuable suggestion. First, we agree that intramolecular processes in the host–guest system are entirely reasonable and indeed unavoidable. For instance, the blue fluorescence under UV irradiation and the yellow phosphorescence observed within seconds after ceasing the excitation are attributed to the intramolecular S_1-S_0 and T_1-S_0 transitions of the guests, respectively. Nevertheless, we believe that the PersL in this host–guest system **primarily** arises from intermolecular charge transfer and trap-mediated processes between the host and guest molecules. As shown in **Supplementary Fig. 17**, almost no thermoluminescence (TL) signal was detected in pure R/S-PyBN. Although pure DPEPO exhibited a relatively weak TL signal, its peak temperature (~180 K) differed substantially from the T_m values of R/S-PyBN@DPEPO (~234/224 K). This result confirms that traps contributing to the PersL recorded at RT **require the participation of both the hosts and guests**. Furthermore, the DFT calculations (albeit qualitative) also support the conclusion of intermolecular electron transfer.

Actions: We have revised the relevant discussion in the manuscript to improve the accuracy of statements as follows.

*"It was noteworthy that R/S-PyBN@DPEPO showed a T_m value of ~234/224 K under a heating rate of 50 K/min. In contrast, the pure R/S-PyBN exhibited almost no TL peak and DPEPO displayed a peak at ~180 K, indicating that the deep traps were not attributed exclusively to either the host or guest molecule. In R/S-PyBN@DPEPO films, those traps were generated due to the intermolecular interactions between the guest and host molecules (**Supplementary Fig. 17**)."* (Page 5 of the revised manuscript, left column, lines 22-29)

"The mechanism suggests that under UV irradiation, electrons in the chiral guest R/S-PyBN are excited. Some excited electrons rapidly return to the guest molecule to undergo radiative emission. Others are transferred to the DPEPO, producing two metastable radical species (R/S-PyBN)$^{\bullet+}$ and DPEPO$^{\bullet-*}$." (Page 5 of the revised manuscript, right column, lines 39-44)

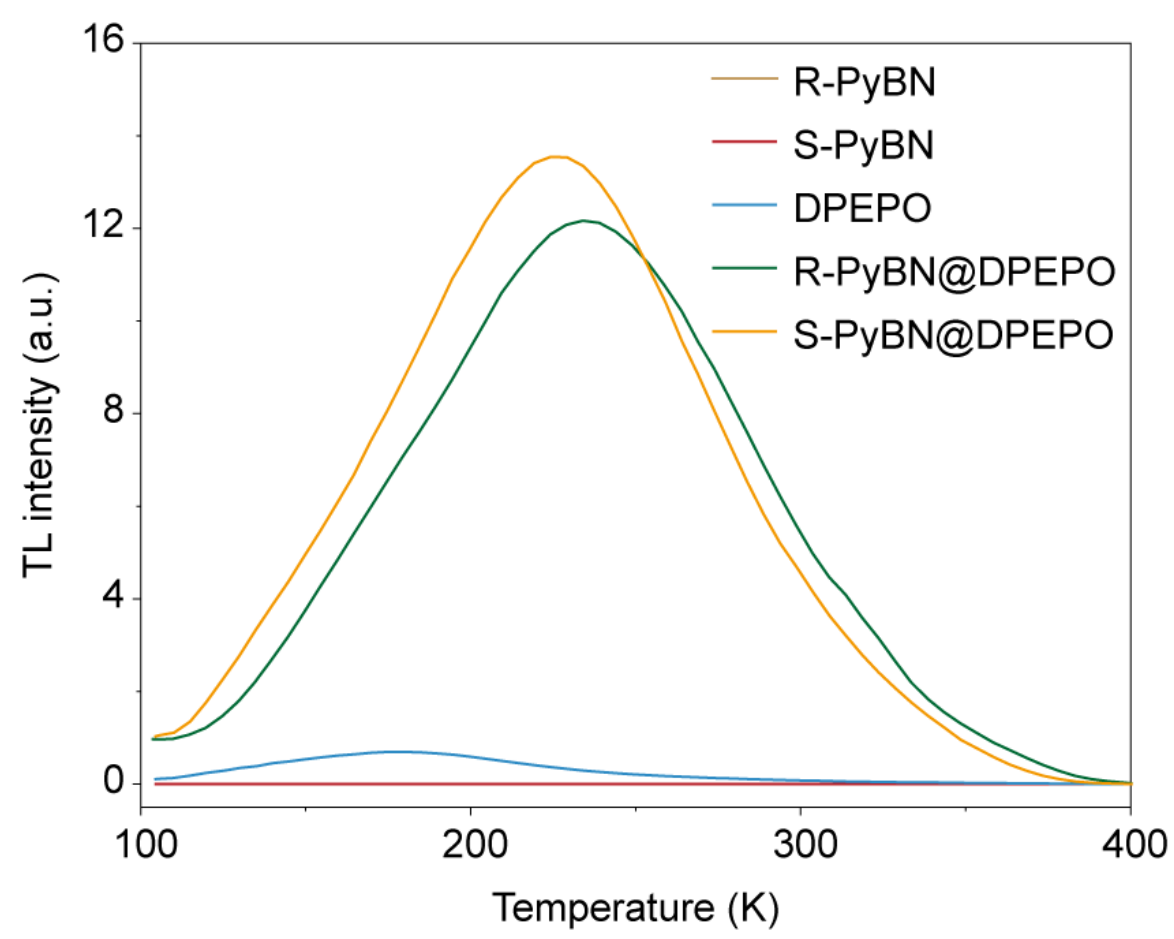

Supplementary Figure 17 | TL glow curves of R/S-PyBN, DPEPO, and R/S-PyBN@DPEPO with a heating rate of 50 K/min. The samples were irradiated by UV light for 300 s before the TL measurements.

Comment #10: *The abstract claims "multi-dimensional properties". Does the paper deliver an actual case of multi-dimensional functionality with independent and orthogonal different properties? Please, discuss or rephrase.*

Response: We sincerely thank the reviewer for the professional feedback regarding the terminology. As the manuscript does not present multi-dimensional functionality with independent and orthogonal different properties, the use of "multidimensional properties" is indeed inappropriate. Alternatively, we believe that "multimodal properties" can be used to describe the distinct luminescent behaviors of the materials under different conditions (*e.g.,* under photoexcitation *versus* after excitation cessation, and with or without polarization). We thank the reviewer again for all

the above enlightening comments and suggestions.

Actions: We have accordingly revised the relevant descriptions throughout the manuscript.

"Leveraging these characteristics, we present a proof-of-concept demonstration of warm-white circularly polarized persistent luminescence materials for Morse-code encryption and multimodal information storage, indicating the potential of organic luminescence materials in the next-generation optical information technologies." (Page 1 of the revised manuscript, left column, lines 16-21)

Referee #2:

This paper (NCOMMS-26-007435) reported a general approach to achieve hour-level white persistent luminescence to CPL materials through the introduction of trap states into an organic host-guest molecular system. The R/S-PyBN@DPEPO can produce white circularly polarized persistent luminescence for 4 h at room temperature. This study demonstrates that two different chiral configurations leave the trap depth basically unaltered, while conferring pronounced CPL activity on these composites with a maximum luminescence dissymmetry factor glum of 1.6 × 10^{-3}/-2.3 × 10^{-3}. White circularly polarized persistent luminescence materials can be applied for Morse-code encryption and multidimensional information storage. In general, the manuscript presents an effective method to develop a kind of application field of CPL materials. I think this paper can be recommended for publication on Nature Communications after the following revision:

Response: We sincerely thank the reviewer for the positive comments on our manuscript. We have carefully revised the manuscript in response to your detailed feedback. Our point-by-point responses are provided below. We hope that these revisions, guided by your valuable suggestions, will further improve the completeness and precision of this work.

Comment #1: *R/S-PyBN@DPEPO should exhibit mirror-imaged CD bands. However, from the Supplementary Figure 15, the mirroring cannot be discerned.*

Response: We would like to express our sincere gratitude to the reviewer for the professional comments regarding the circular dichroism (CD) measurements. Theoretically, the CD bands of samples containing R and S chiral molecules should, as the reviewer mentioned, exhibit mirror-image symmetry. To address this concern, we remeasured the CD spectra of R/S-PyBN@DPEPO and compared them with those of R/S-PyBN dispersed in toluene (**Supplementary Fig. 13**). The results indicate that, although the CD signals of R/S-PyBN@DPEPO are predominantly positive and negative, respectively, perfect mirror-image symmetry was not achieved. In contrast, R/S-PyBN in toluene displayed well-defined mirror-image symmetric signals.

The deviation from ideal mirror-image symmetry in R/S-PyBN@DPEPO could be attributed to two factors. First, the relatively low concentration (1 wt%) of guest chiral molecules in the host–guest system resulted in weak signals (poor signal to noise ratio). Furthermore, significant light-scattering in the melt-casting samples substantially attenuated the CD signals. We note that the CD measurements in solid materials have long been challenging owing to scattering artifacts. Analogous results have been reported in the literature (*e.g.*, *Nat. Commun.* **2023**, *14*, 1654; *Angew. Chem. Int. Ed.* **2021**, *60*, 3745; *Angew. Chem. Int. Ed.* **2023**, *62*, e202302751; *Angew. Chem.* **2024**, *136*, e202407596).

We have made considerable efforts during the revision (repeated the measurements for several times), but we were unfortunately unable to achieve perfect mirror-image CD bands for R/S-PyBN@DPEPO. We appreciate your understanding of the inherent experimental limitations.

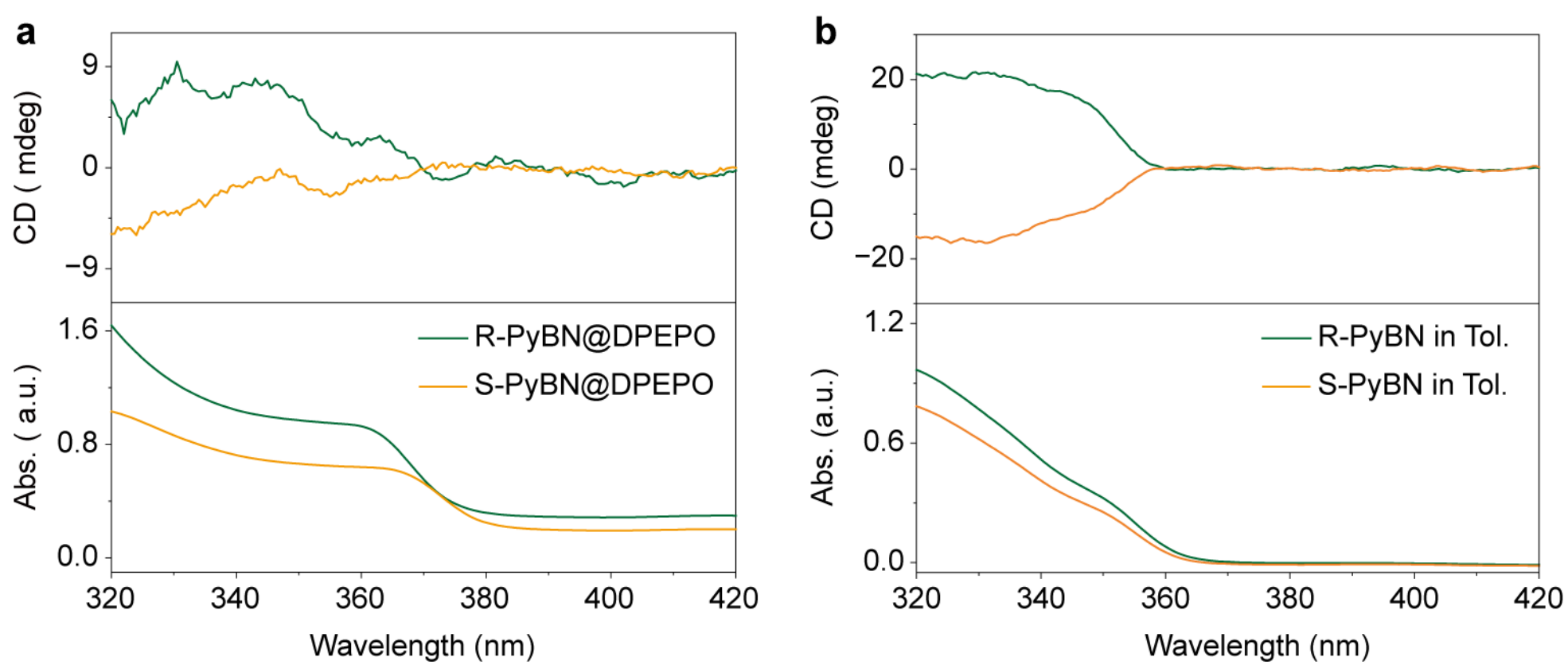

Supplementary Figure 13 | Circular dichroism (CD, top) and ultraviolet-visible absorption (Abs., bottom) spectra of R/S-PyBN@DPEPO in melt-casting films (a) and R/S-PyBN in toluene (b) at RT.

Comment #2: *Compared with R/S-PyBN in toluene, the fluorescence emission of R/S-PyBN film showed a red shift, the explanation should be provided in the main text.*

Response: We are grateful to the reviewer for the constructive comment. Compared with R/S-PyBN dispersed in toluene, the red shift of fluorescence emission in R/S-PyBN films and powders are primarily attributed to molecule aggregation. In aggregated states, reduced intermolecular distances enhance intermolecular interactions and promote orbital overlap between adjacent molecules. This aggregation induces Davydov splitting, yielding lower excited-state energy and thereby causing red shift in the emission. Additionally, intermolecular polarization effects in the film may also lower the excited-state energy, also contributing to the observed red shift. Similar aggregation-induced red shifts have been reported in the literature (*e.g., Adv. Mater.* **2014**, *26*, 5429–5479; *Adv. Mater.* **2019**, *31*, 1808242; *Nat. Commun.* **2019**, *10*, 2952).

Actions: We have included the corresponding explanations into the main text according to your suggestion.

"Compared with the well-resolved structured emission of isolated molecules in toluene solution, the fluorescence of R/S-PyBN in powders and films featured a broad emission band with a pronounced red shift, which could be attributed to aggregation forms with stronger intermolecular interactions[56,57]." (Page 2 of the revised manuscript, right column, lines 2-6)

Comment #3: *Lifetime decay profiles of DPEPO in a neat film at 77 K should be measured.*

Response: We appreciate the reviewer's comments on the photophysical properties of DPEPO. Indeed, the characterization on DPEPO in the original manuscript was insufficient. In this revision, we have accordingly provided the fluorescence and delayed fluorescence spectra of DPEPO in both powder and neat film forms (**Supplementary Fig. 5**). Meanwhile, we have measured the lifetime decay curve of the DPEPO neat film at 77 K (**Supplementary Fig. 6**). The results show that DPEPO, irrespective of in powder or neat film form, exhibits nearly identical fluorescence and delayed fluorescence peaks at both 77 K and RT. Consequently, the decay curve monitored at 465 nm comprises a short-lived component (τ_1 5.52 ms) and long-lived components (τ_2 72.89 ms, τ_3 277.88 ms,), which together yield an average lifetime of 215.31 ms (**Supplementary Table 1**).

These results confirm that the fluorescence (also delayed fluorescence) of DPEPO at approximately 490 nm is not directly associated with the fluorescence (also phosphorescence and PersL) in R/S-PyBN@DPEPO. This further supports the assignment of the luminescence in the host–guest system to the guest molecules R/S-PyBN (**Fig. 2a**).

Actions: We have added the following text in the manuscript and figures/table in the Supplementary Information.

*"In contrast, DPEPO, irrespective of in powder or neat film form, exhibited nearly identical fluorescence and delayed fluorescence bands either at 77 K or at RT (**Supplementary Fig. 5**). At 77 K, the emission band of DPEPO film is peaked at approximately at 465 nm with a fluorescence lifetime of 215.31 ms (**Supplementary Fig. 6** and **Supplementary Table 1**)." (Page 2 of the revised manuscript, right column, lines 16-21)*

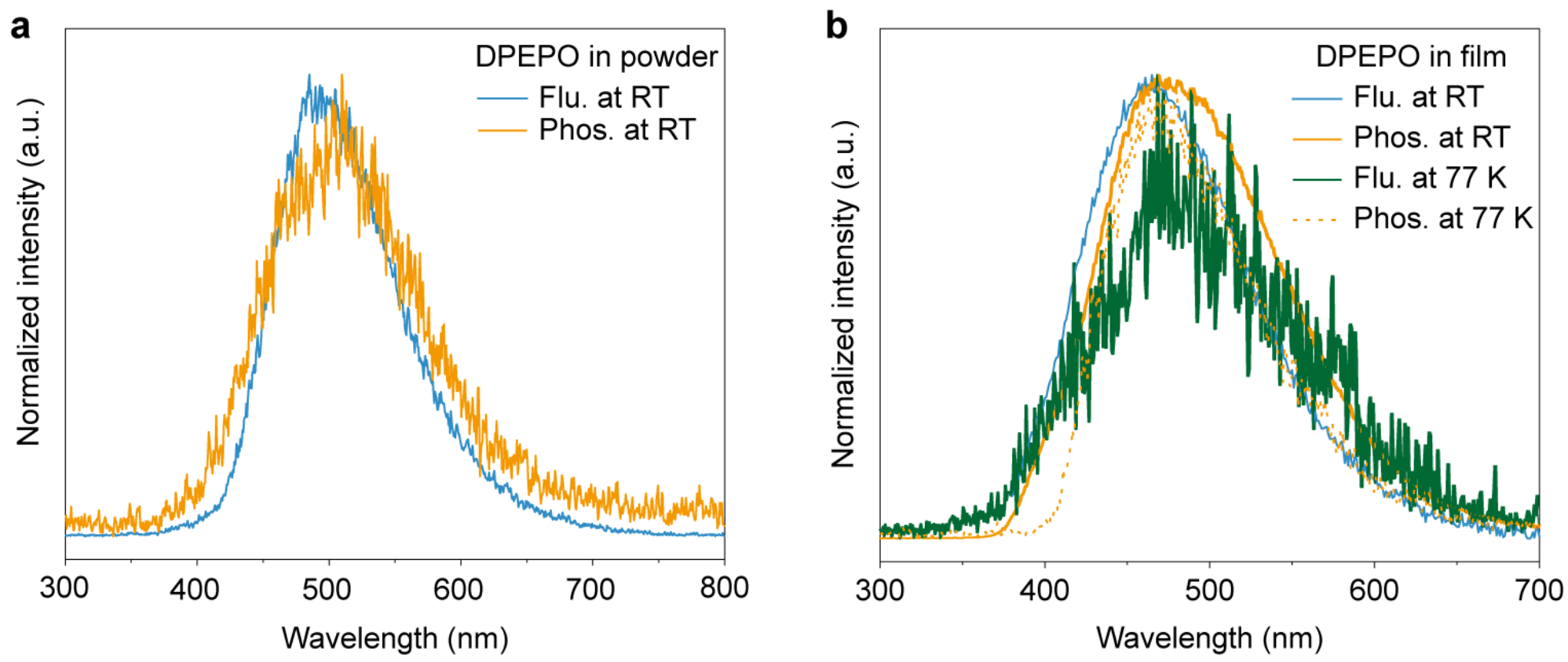

Supplementary Figure 5 | Fluorescence (Flu.) and phosphorescence (Phos.) spectra of DPEPO in powder (a) and in neat film (b). The delayed time of phosphorescence was 5 ms at RT and 8 ms at 77 K.

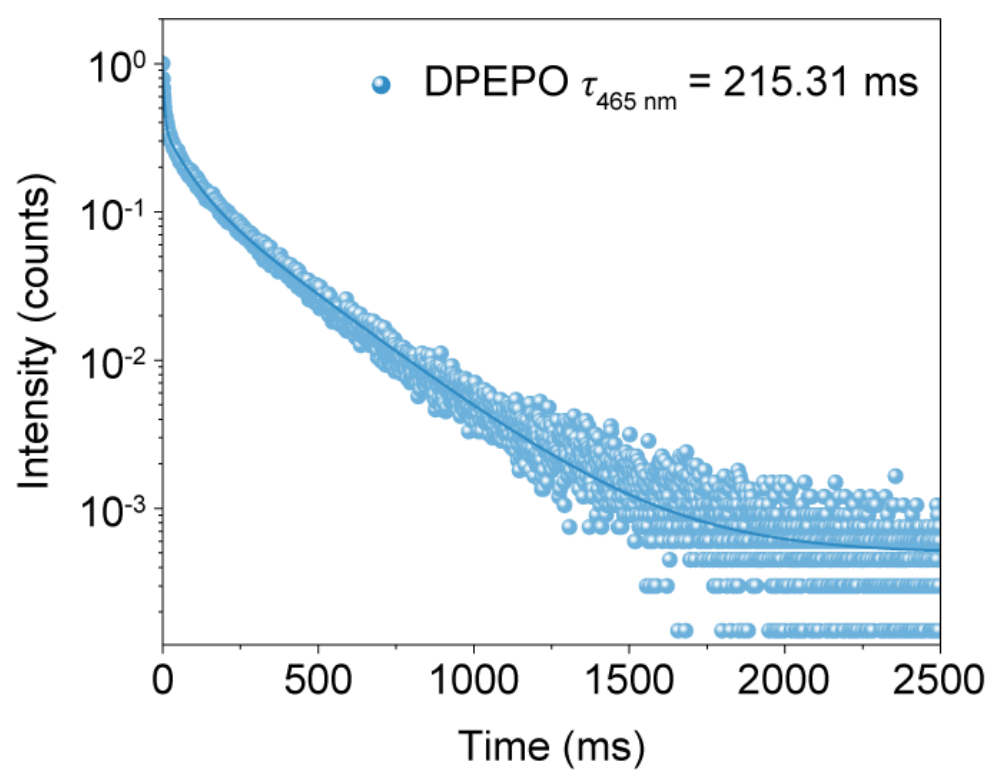

Supplementary Figure 6 | Normalized fluorescence (prompt) lifetime decay curves of the emissions at 465 nm for DPEPO films at 77 K.

Comment #4: *The phosphorescence spectra of R/S-PyBN@DPEPO showed a blue shift compared to that of pure R/S-PyBN in neat films at 77 K. Please explain it.*

Response: We thank the reviewer once again for the valuable suggestion regarding the spectral property of R/S-PyBN. As shown in **Supplementary Fig. 10**, the phosphorescence bands of R/S-PyBN@DPEPO at 77 K (peaked at ~530 nm) exhibited an obvious blue shift relative to those of R/S-PyBN film (peaked at ~570 nm). This is primarily attributed to the dispersion of the guest molecules within the host–guest system, wherein the increased distance between guest molecules suppresses intermolecular interactions, thereby giving rise to the observed blue-shifted emission.

Actions: We have added the following text into the manuscript according to your suggestion.

"We note that, owing to the weakened aggregation effect caused by the dispersion of guest molecules in the host-guest system, both the fluorescence and phosphorescence emissions of the R/S-PyBN@DPEPO films showed a blue shift compared to those of pure R/S-PyBN film[62,63]*."* (Page 4 of the revised manuscript, left column, lines 13-18)

Comment #5: *The author mentioned "different chiral configurations leave the trap depth basically unaltered", but the log-log plot of PersL decay curves of 1 wt% R/S-PyBN@SPPO13 showed significantly different luminescence times.*

Response: We sincerely thank the reviewer for pointing out the drawback of this work. Significant discrepancies were indeed presented in the previous PersL decay curves of R/S-PyBN@SPPO13. The discrepancies were attributed to

experimental errors such as sample dimension, positioning, excitation power, and other measurement parameters. Accordingly, we remeasured the two samples under identical experimental conditions but with stricter control over the uncertainties**.** The updated results are provided in **Supplementary Fig. 24d**. The results indicate that, **while minor deviations in the decay curves between two chiral configurations remain unavoidable, the differences have been substantially reduced relative to the previous results**. We believe these updated experimental data may support the conclusion that the trap depth is basically unaltered across different chiral configurations.

Actions: We have updated the following figure in the Supplementary Information.

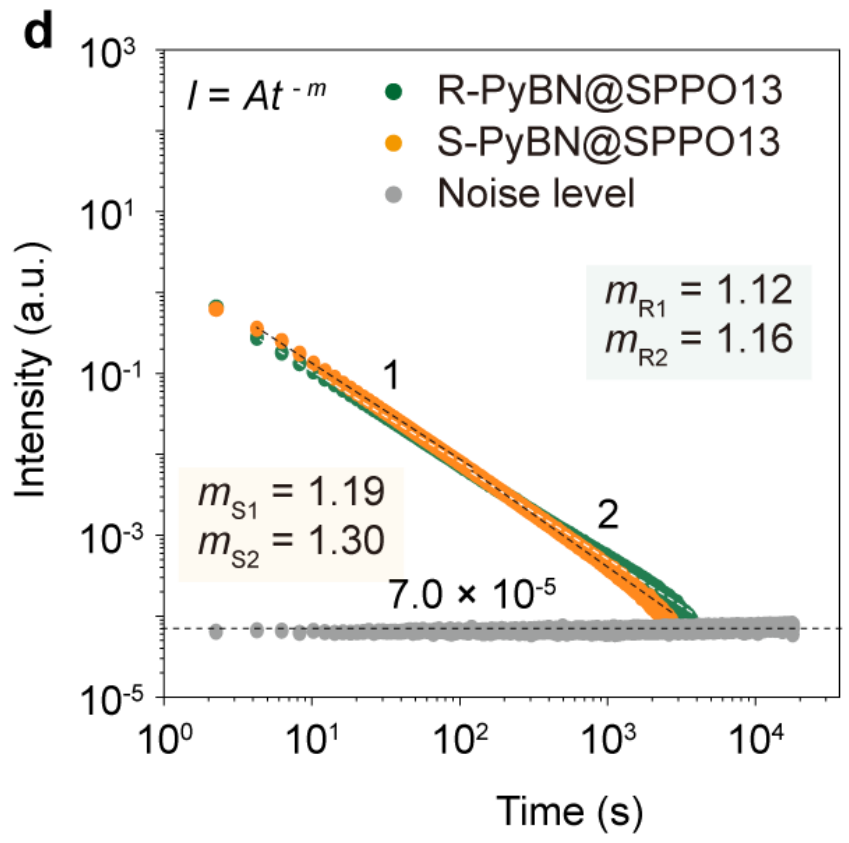

Supplementary Figure 24d | Log-log plot of PersL decay curves of R/S-PyBN@SPPO13 under 365 nm excitation. The excitation temperature and recoding temperature were RT. The excitation time was 60 s.

Comment #6: *The CD spectra of R/S-APyBN@DPEPO (Ri/S-i), R/S-PyBNMO@DPEPO (R-ii/S-ii), R/S-PyBN@BCPO (R-iii/S-iii), and R/S-PyBN@SPPO13 (R-iv/S-iv) should be measured.*

Response: We are grateful for the reviewer's constructive suggestion to provide the CD spectra of other composite films, which we believe would significantly enrich the discussion of this work. Accordingly, we have performed CD measurements on other four samples (**Supplementary Figure 31**). The results confirmed that the samples containing R- and S-type guest molecules generally exhibited positive and negative Cotton effects, respectively. However, similar to R/S-PyBN@DPEPO in **Supplementary Fig. 13a**, the resulting CD spectra displayed a relatively poor signal-to-noise ratio owing to the low guest concentrations and light scattering, and thus did not exhibit ideal mirror-image symmetry.

Actions: We have included these data in the Supplementary Information and added a short description on the date in the manuscript as follows.

*"Furthermore, we confirmed that all these host-guest systems maintained the chiral characteristics of the ground state and the excited state (**Fig. 4f** and **Supplementary Fig. 31**)."* (Page 7 of the revised manuscript, right column, lines 3-4)

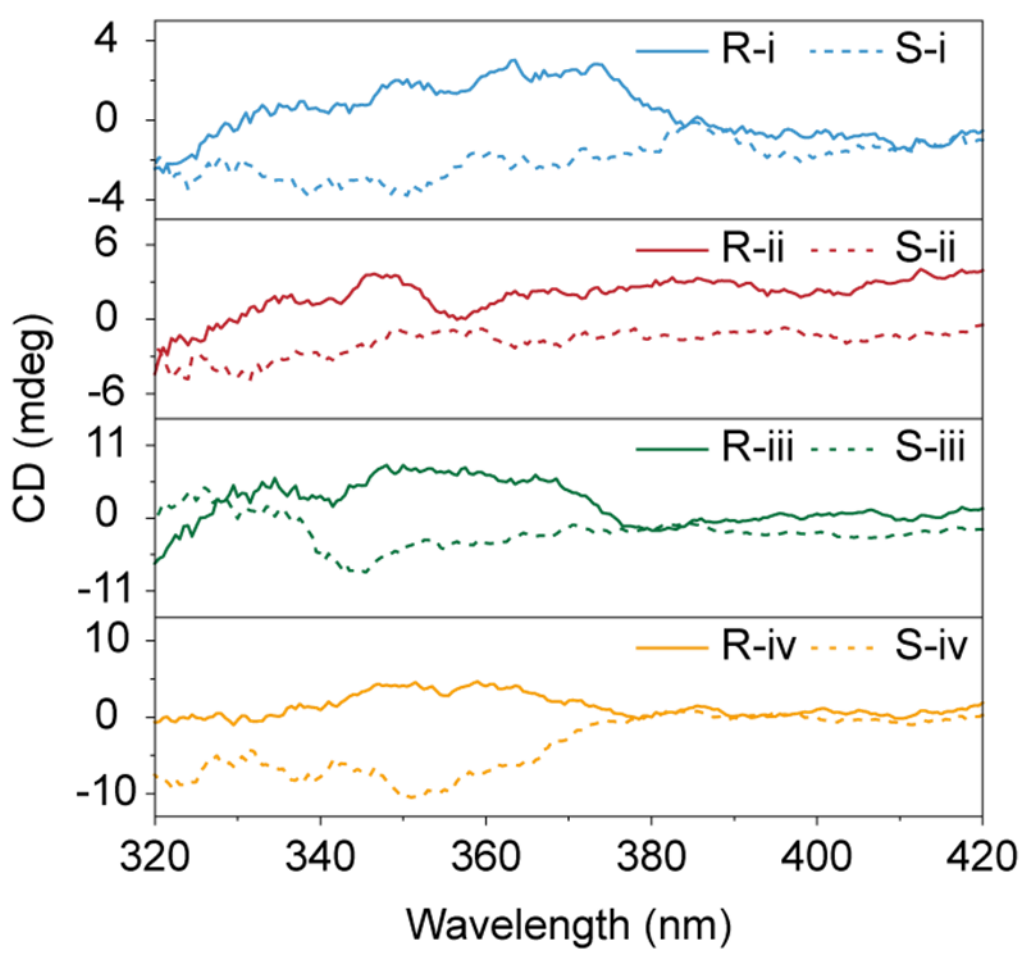

Supplementary Figure 31 | Circular dichroism (CD) spectra of R/S-APyBN@DPEPO (R-i/S-i), R/S-PyBNMO@DPEPO (R-ii/S-ii), R/S-PyBN@BCPO (R-iii/S-iii), and R/S-PyBN@SPPO13 (R-iv/S-iv) melt-casting films at RT.

Comment #7: *There are some textual errors in the text, such as "R-PyBN@SPPO13" should be "S-PyBN@SPPO13"in Supplementary Figure 25 d. And the format of reference 51, 55 is different from the others. Please correct it.*

Response and Actions: We appreciate the reviewer's meticulous reviews. We are very sorry for our carelessness. We have relabeled the tags in **Supplementary Fig. 24d** and modified the format of **References 51, 55**, as shown below. Moreover, we also carefully checked the references to avoid similar mistakes.

Updated References 51, 55 in the manuscript:

51. Huo, S. W., Duan, P. F., Jiao, T. F., Peng, Q. M. & Liu, M. H. Self-assembled luminescent quantum dots to generate full-color and white circularly polarized light. Angew. Chem. Int. Ed. ***56****, 12174–12178 (2017).*

55. Li, J.-A. et al. Colour-tunable dual-mode afterglows and helical-array-induced mechanoluminescence from AIE enantiomers: effects of molecular arrangement on formation and decay of excited states. Chem. Eng. J. ***418****, 129167 (2021).*

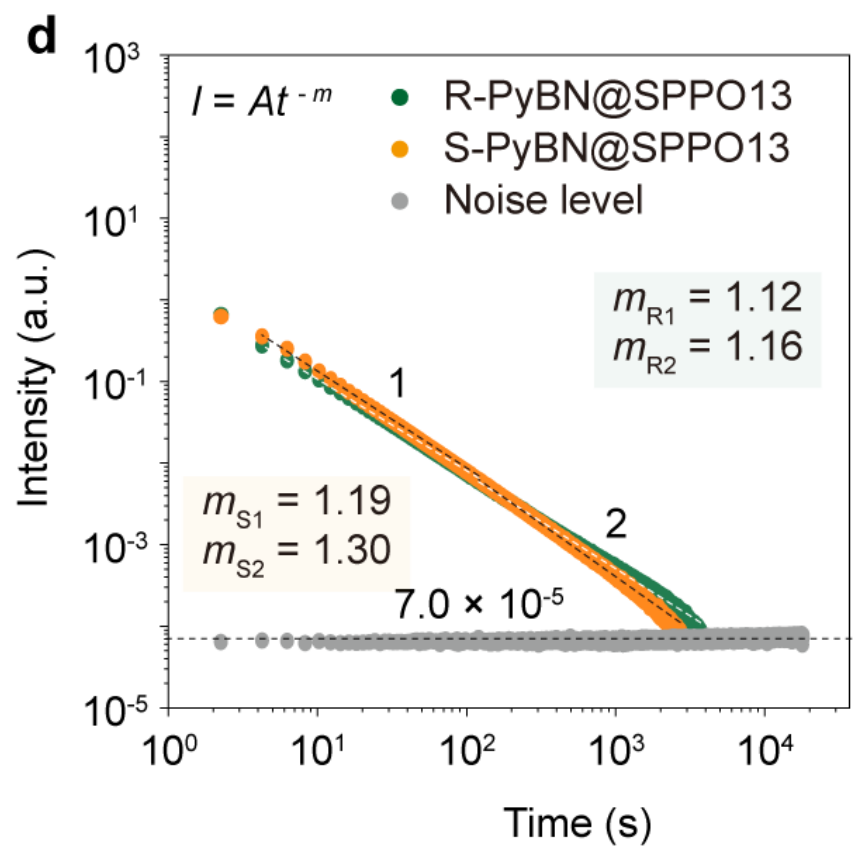

Supplementary Figure 24d | Log-log plot of PersL decay curves of R/S-PyBN@SPPO13 under 365 nm excitation. The excitation temperature and recoding temperature were RT. The excitation time was 60 s.

Referee #3:

This manuscript reports a host-guest molecular system (R/S-PyBN@DPEPO, etc.) that achieves white circularly polarized persistent luminescence (CP-PersL) through the introduction of trap states. The authors claim that the material exhibits persistent luminescence for over 4 hours at room temperature, with a maximum luminescence dissymmetry factor $|g_{lum}|$ *on the order of* 10^{-3}*. Applications in Morse code encryption and multidimensional information storage are also demonstrated. After careful review, I find that this manuscript has the following fundamental issues and does not meet the publication standards of Nature Communications:*

Response: We sincerely thank the reviewer for carefully reviewing our manuscript and providing constructive comments. We believe all your comments are of great value to this work. We have carefully considered your comments and have accordingly made comprehensive revisions to the manuscript and Supplementary Information. As shown in details in the following point-by-point responses, we have reorganized the presentation of this work's innovation, supplemented necessary photophysical and purity data, and refined the terminology and discussion of application prospects. We sincerely hope that, under the guidance of your constructive comments and suggestions, these revisions have significantly improved the quality of the manuscript.

Comment #1: *Trap-induced organic persistent luminescence was not first proposed in this study. Furthermore, the strategy of achieving white persistent luminescence through a combination of singlet-singlet and singlet-triplet transitions has been extensively reported in organic afterglow materials. Examples include Angew. Chem. Int. Ed. 2021, 60, 24984; Angew. Chem. Int. Ed. 2023, 62, e202310335, among others. This work merely introduces chiral molecules into an existing trap-induced luminescence system. Such "chiralization" represents incremental work and lacks a substantial breakthrough.*

Response: We sincerely thank the reviewer for the insightful comments regarding the differences between this work and previous reports, which have made us understand that the novelty of our work was not expressed with sufficient clarity in the first submission. We apologize for this shortcoming and have revised the manuscript accordingly. We hope that the following two points will help present the innovation of this work more clearly.

(1) We have carefully examined the references provided by the reviewer. These reports achieve white afterglow through a strategy that combines delayed fluorescence (short-wavelength band) and phosphorescence (long-wavelength band) using materials with a **small ΔE_{ST}**, thereby enabling simultaneous acquisition of both types of long-lived emissions at RT. In contrast, our work utilizes the concept of trap-induced organic PersL, which **differs substantially from the previous reports in terms of luminescence mechanism and material design strategy**. Employing trap depth as a tuning parameter **allows precise modulation of the emission duration and working temperature**, which we believe offers distinct advantages.

Indeed, trap-induced organic PersL was not first demonstrated by this work. Nevertheless, **the key advance of this study lies in revealing an unreported electron repopulation mechanism in which detrapped electrons repopulate both triplet and singlet states**. As shown in **Supplementary Fig. 14**, within several seconds after ceasing UV excitation (1 ms-5 s), R/S-PyBN@DPEPO exhibits almost exclusive yellow phosphorescence (triplet emission); unexpectedly, thereafter, warm-white PersL emerges with contributions from both triplet and singlet emissions. This arises because the guest molecules possess a **large ΔE_{ST}**, such that direct excitation to the S_1 state at RT produces only phosphorescence without generating conventional delayed fluorescence (which relies on RISC). Detrapped electrons from higher-lying energy levels repopulate both the triplet and singlet states of the guest molecules, thereby achieving dual-peak emission lasting for hours. This not only provides an important complement to the trap-induced PersL mechanism but also offers an effective tool to probe charge-carrier migration processes in organic luminescent materials.

(2) Another notable aspect of this work is the introduction of chirality into the trap-induced organic PersL system. Prior to this work, the effect of chiral configurations on trap-induced organic PersL had remained unexplored. In this study, **we**

demonstrate for the first time that chirality exerts negligible influence on both trap depth and emission wavelength. We believe this finding provides valuable guidance for fine-tuning the performance parameters of organic luminescent materials toward applications such as multimodal information storage.

Actions: We have revised the manuscript to more clearly highlight the novelty and key advances of this study relative to previous reports.

"The electrons released from the trap states populate both the singlet and triplet states of the chiral guest molecules, producing dual-band white circularly polarized persistent luminescence for 4 h at room temperature. Systematic characterization reveals that two different chiral configurations leave the trap depth basically unaltered, while conferring pronounced CPL activity on these composites with a maximum luminescence dissymmetry factor g_{lum} of 1.6×10^{-3}/-2.3×10^{-3}." (Page 1 of the revised manuscript, left column, lines 9-16)

"Dual-band white PersL is achieved by releasing trapped electrons that populate both the singlet (S_1) and triplet (T_1) states of guest molecules characterized by a large singlet–triplet energy gap (ΔE_{ST}) (Fig. 1b)." (Page 2 of the revised manuscript, left column, lines 3-6)

*"Notably, the time-resolved spectra revealed the evolution of singlet and triplet contribution in the entire emission processes (**Fig. 2d** and **Supplementary Fig. 14**). The emission spectra recorded in the range of 1 ms to 5 s were predominantly governed by the triplet states. In contrast, the relative contribution of singlet states increased substantially after 5 s (from 5 s to 60 min), reaching an intensity comparable to that of triplet emissions. These results indicate that, in the PersL process electrons are populated into the S_1 and T_1 states, causing both singlet-singlet and triplet–singlet transitions to generate long-lasting PersL."* (Page 4 of the revised manuscript, right column, lines 6-15)

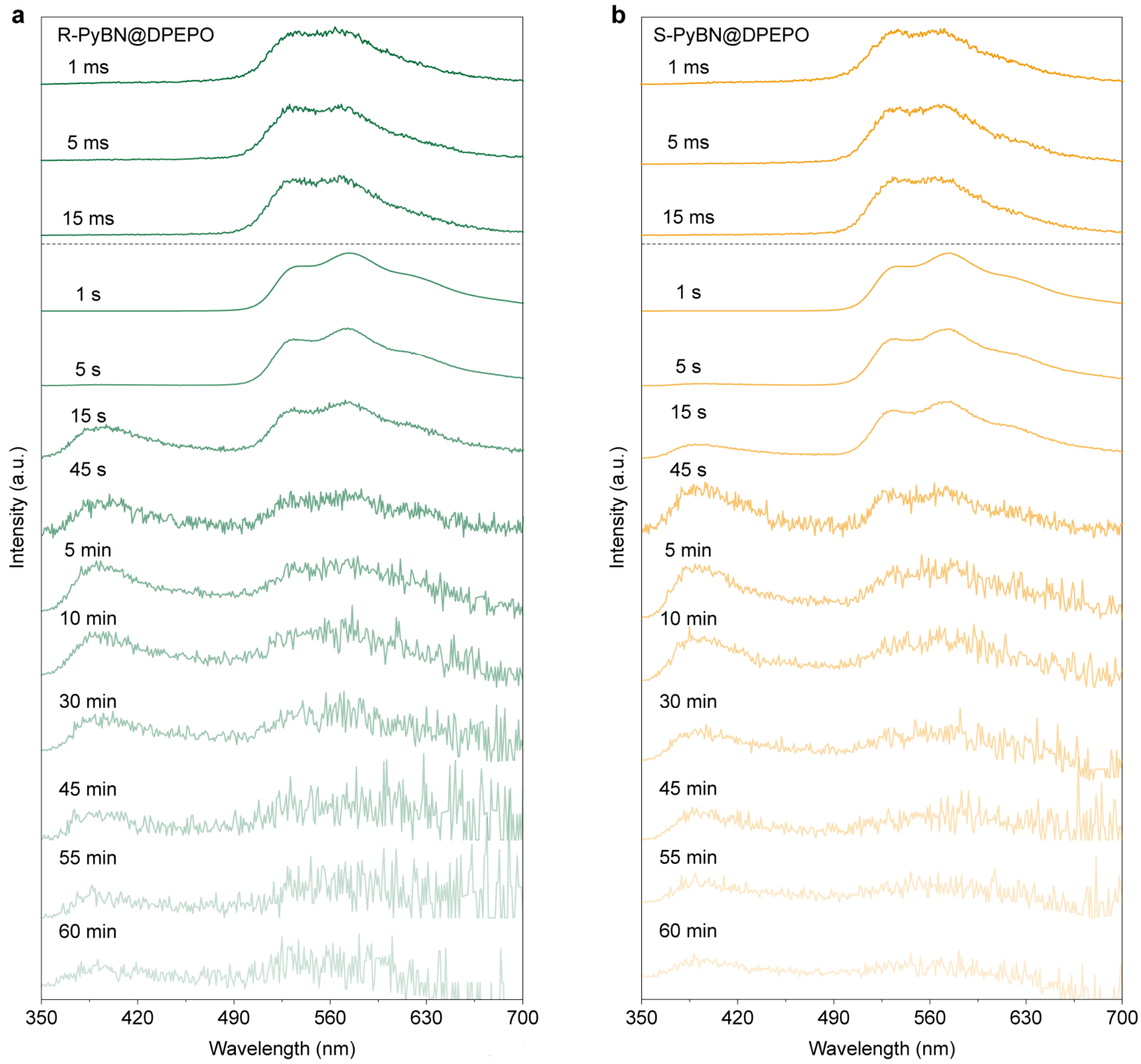

Supplementary Figure 14 | Time-resolved emission spectra of R-PyBN@DPEPO (a) and S-PyBN@DPEPO (b) films. The spectra recorded at 1, 5, and 15 ms were obtained after cessation of 355 nm short-pulse excitation, whereas the spectra from 1 s to 60 min were collected after 1 min of continuous 365 nm excitation.

Comment #2: *The claim of achieving white light due to comparable singlet and triplet emission intensities is not well-supported by the data. Figure 2d shows that in the persistent luminescence spectrum, the yellow/orange emission component (~550-600 nm) is significantly stronger than the blue component (~400 nm). The CIE coordinates (0.36, 0.42) are actually closer to yellow light than to the ideal white point (0.33, 0.33).*

Response: We apologize for the imprecise description in the previous manuscript. As the reviewer pointed out, the PersL of R/S-PyBN@DPEPO exhibited a higher yellow component, with its CIE coordinates deviating from the ideal white point toward the yellow region. Accordingly, we have revised the description of the emission color for this sample to "warm-white" throughout the manuscript to improve accuracy.

We note that the CIE coordinates of white light generated by two complementary emissions are, in principle, readily tunable. Using the strategy proposed in this work, the deviation of the R/S-PyBN@DPEPO emission from the ideal white point can be addressed by adjusting the wavelengths and relative intensities of the two PersL bands. We sincerely thank the reviewer for the constructive comments.

Actions: We have revised the description of the emission color of R/S-PyBN@DPEPO throughout the manuscript to improve accuracy. One of the revisions is cited below.

*"The resultant dual-band PersL spectra covered complementary blue and yellow regions, yielding warm-white PersL with Commission International de l'Eclairage (CIE) coordinates of (0.36, 0.40)/(0.37, 0.40) (**Fig. 2e**)" (Page 4 of the revised manuscript, right column, lines 15-18)*

Comment #3: *The performance metrics lack competitiveness, and the dissymmetry factor is relatively low. The reported |glum| values are only on the order of 10^{-4}-10^{-3}. This is not suitable for the claimed encryption applications, as chiral luminescent materials with $|g_{lum}| < 10^{-2}$ are highly susceptible to producing false positive/negative signals due to sample preparation variations. Moreover, the two chiral enantiomers show no significant difference in their afterglow luminescence, rendering them not directly meaningful for chirality-based encryption.*

Response: We thank the reviewer for the insightful comments regarding the dissymmetry factor. We acknowledge that the $|g_{lum}|$ values of our samples are relatively small, which constitutes a limitation of this work. We consider this is primarily attributed to the intrinsic properties of the selected chiral molecules, specifically their inherently weak magnetic transition dipole moments. As is well documented, many reported organic CPL materials exhibit small dissymmetry factors, typically on the order of 10^{-5}~10^{-3} (*Angew. Chem.* **2025**, *137*, e202417906.; *Coord. Chem. Rev.* **2022**, *453*, 214329). Such small $|g_{lum}|$ values may indeed introduce sign ambiguity, thereby limiting practical applications. Accordingly, the applications of information encryption and multimodal information storage utilizing PersL and CPL features as presented in the work **should be regarded as proof-of-concept demonstrations**.

Furthermore, we believe that the general design strategy proposed in this work to extend the emission lifetime of CPL-active materials, if implemented using chiral guest molecules with superior CPL performance, is expected to afford larger $|g_{lum}|$ values while preserving hour-level PersL. This represents an important direction for our future efforts. We thank the reviewer for this valuable comment.

Actions: We have revised the descriptions of the application demonstrations in the manuscript.

"Leveraging these characteristics, we present a proof-of-concept demonstration of warm-white circularly polarized

persistent luminescence materials for Morse-code encryption and multimodal information storage, indicating the potential of organic luminescence materials in the next-generation optical information technologies." (Page 1 of the revised manuscript, left column, lines 16-21)

"Considering the unique properties of warm-white PersL combined with CPL in the synthesized materials, we further conducted a proof-of-concept experiment on optical information encryption" (Page 8 of the revised manuscript, left column, lines 2-5)

Comment #4: *The luminescence quantum yield is not explicitly reported. This is crucial for evaluating the practical application value of luminescent materials.*

Response and Actions: We are grateful to the reviewer for this valuable suggestion about quantum yield measurements. Accordingly, we have measured the luminescence quantum yields of all host-guest systems and incorporated the corresponding data into the revised manuscript and Supplementary Information (**Supplementary Table 4**). The descriptions in the main text are also cited below.

"The luminescence quantum yields (QY) of R/S-PyBN@DPEPO films were 38.4/39.8% under ambient condition, indicating the existence of a rigid environment provided by the host matrix." (Page 4 of the revised manuscript, left column, lines 3-6)

*"Under UV irradiation, all of these composite films exhibited blue emission, with corresponding QY values of 19.0/20.3% for R/S-APyBN@DPEPO, 33.8/42.6% for R/S-PyBNMO@DPEPO, 31.9/35.2% for R/S-PyBN@BCPO, and 21.1/20.1% for R/S-PyBN@SPPO13 (**Supplementary Table 4**)." (Page 6 of the revised manuscript, right column, lines 1-5)*

Comment #5: *The purity of the host and guest materials used in the study needs to be verified by high-performance liquid chromatography (HPLC) to rule out room-temperature phosphorescence originating from impurities.*

Response: We appreciate the reviewer's insightful suggestion regarding purity verification. Following your suggestion, we have assessed the purity of the host and guest materials by high-performance liquid chromatography (HPLC) with detection at both 220 and 254 nm. The corresponding chromatograms are presented in **Supplementary Figs. 48-51**. The results show that all materials display a single dominant peak, confirming high chemical purity (>99%) and suitability for this study.

Actions: We have included the HPLC results in the Supplementary Information and incorporated the relevant discussion into the manuscript.

*"The purity of the used guests and hosts were verified by high-performance liquid chromatography (HPLC, LCMS2020, Shimadzu). The NMR and HPLC spectra are provided in **Supplementary Figs. 34-51**. The results confirmed the high chemical purity of the samples and suitability for this study" (Page 8 of the revised manuscript, left column, line 53-56 and right column, lines 1-2)*

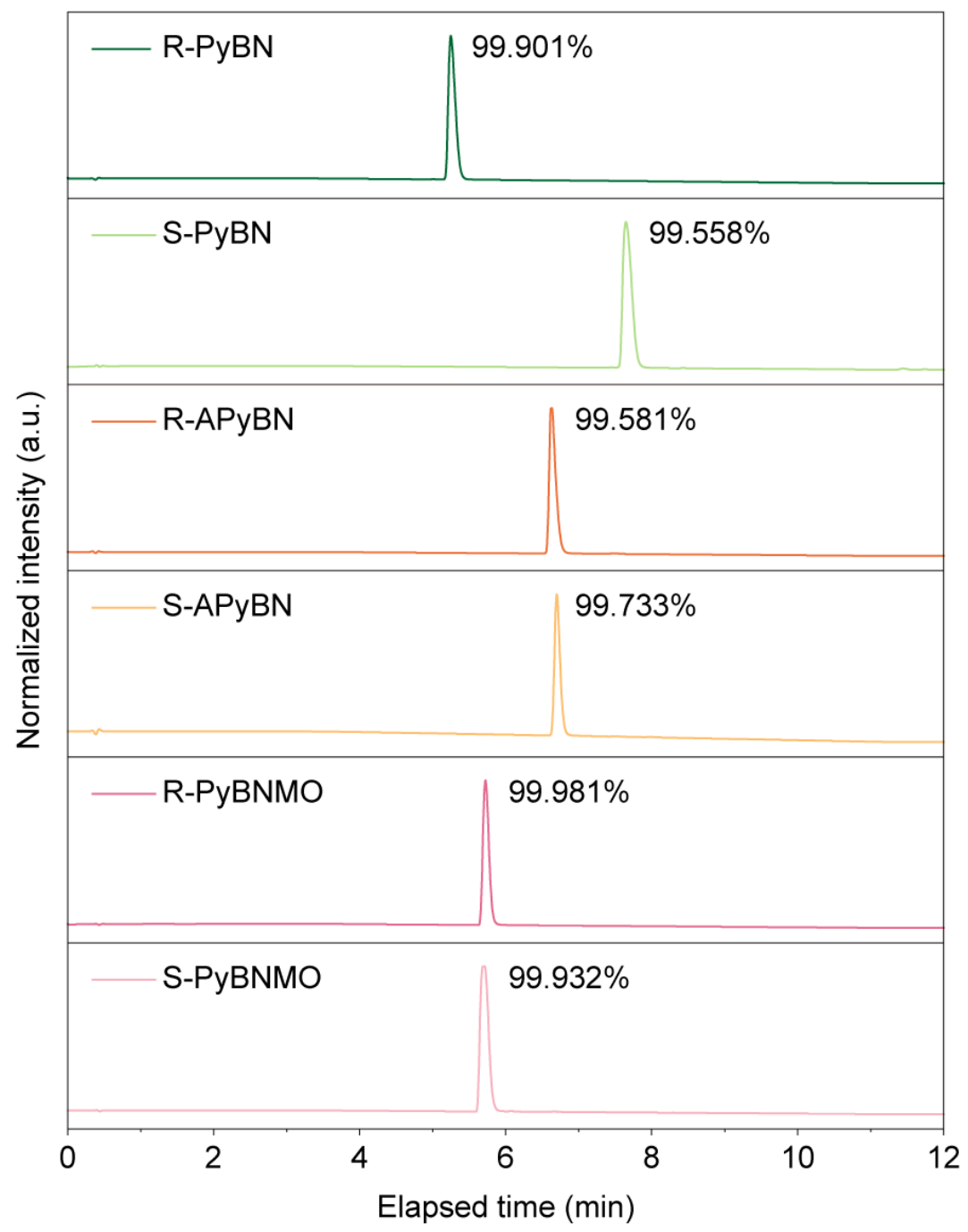

Supplementary Figure 48 | HPLC profiles of R/S-PyBN, R/S-APyBN, and R/S-PyBNMO monitored at 220 nm. Temperature, 40°C; mobile phase, H_2O/MeCN/TFA; flow rate = 2.5 mL/min; column, ultimate XB-C18 (50 × 4.6 mm, 5 μm).

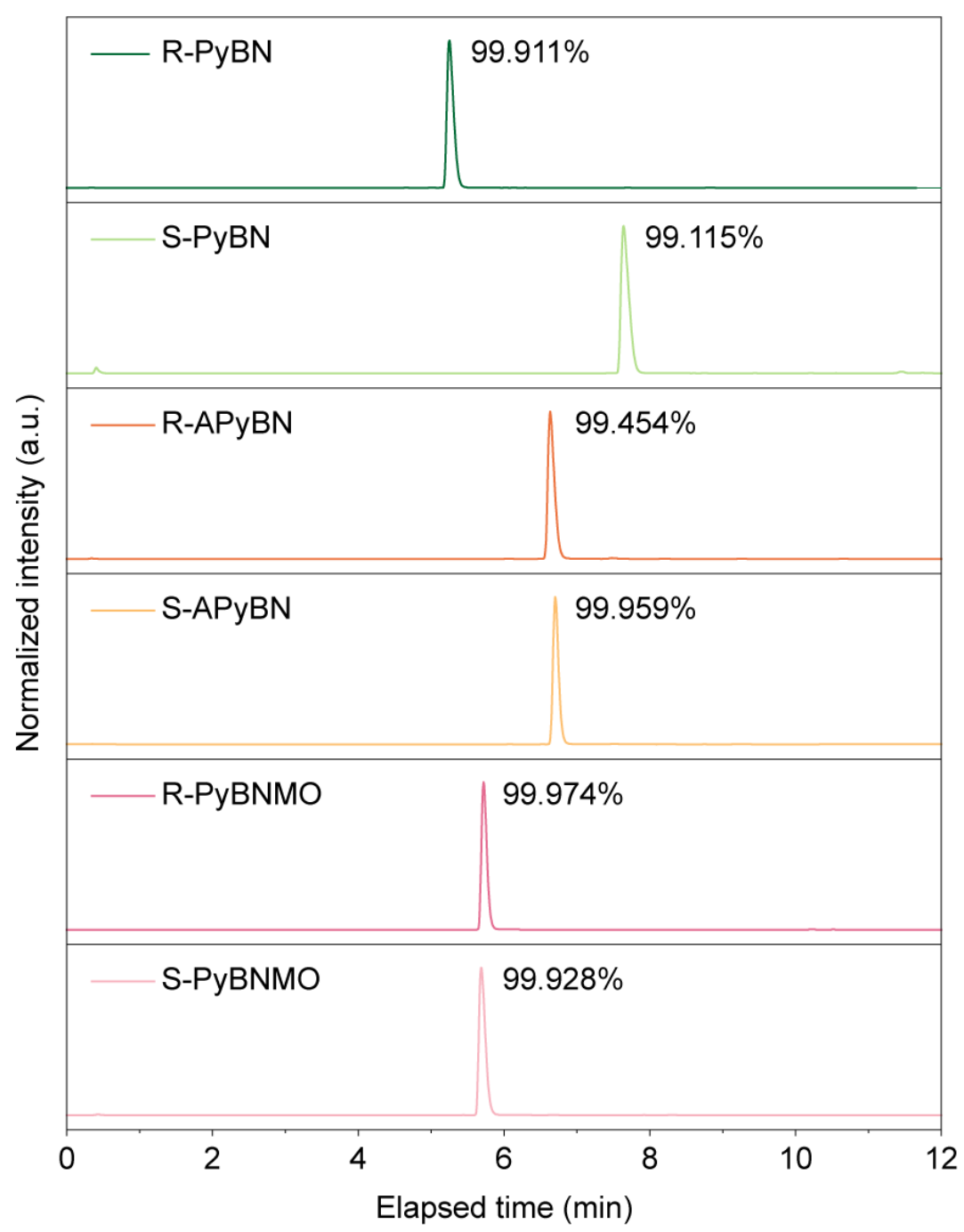

Supplementary Figure 49 | HPLC profiles of R/S-PyBN, R/S-APyBN, and R/S-PyBNMO monitored at 254 nm. Temperature, 40°C; mobile phase, H_2O/MeCN/TFA; flow rate = 2.5 mL/min; column, ultimate XB-C18 (50 × 4.6 mm, 5 μm).

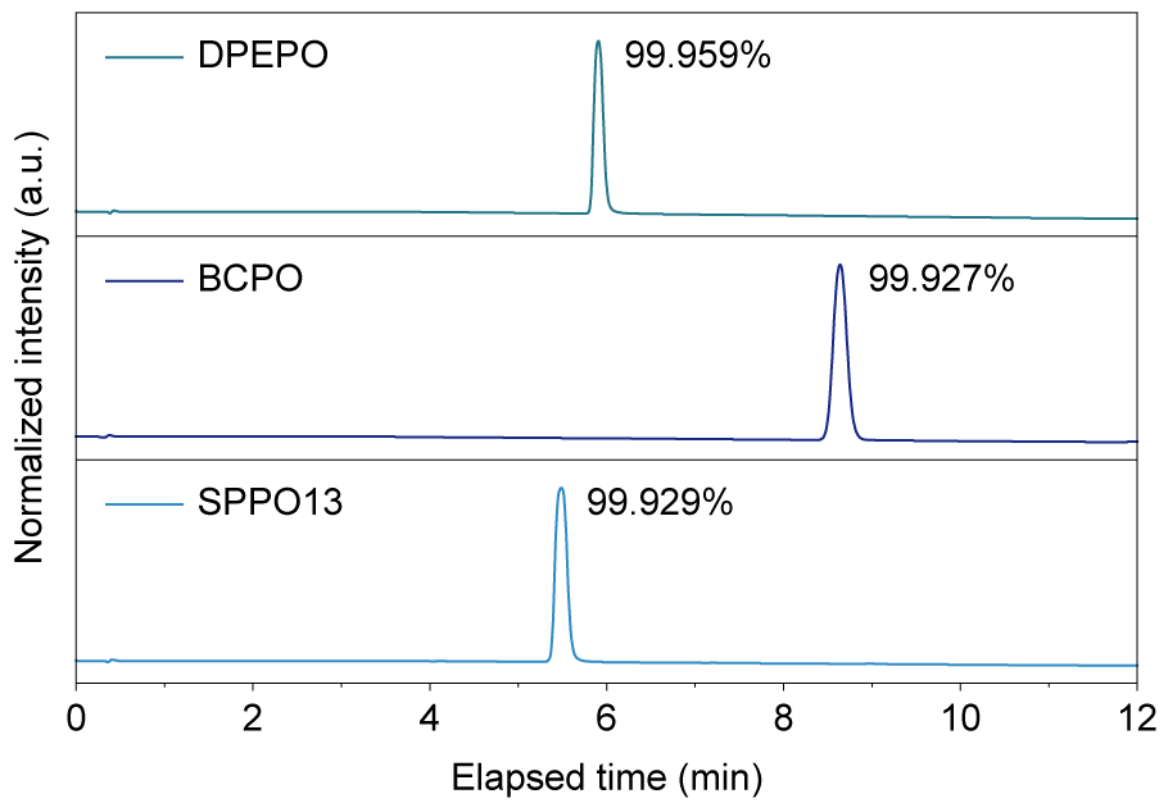

Supplementary Figure 50 | HPLC profiles of DPEPO, BCPO, and SPPO13 monitored at 220 nm. Temperature, 40°C; mobile phase, H_2O/MeCN/TFA; flow rate = 2.5 mL/min; column, ultimate XB-C18 (50 × 4.6 mm, 5 µm).

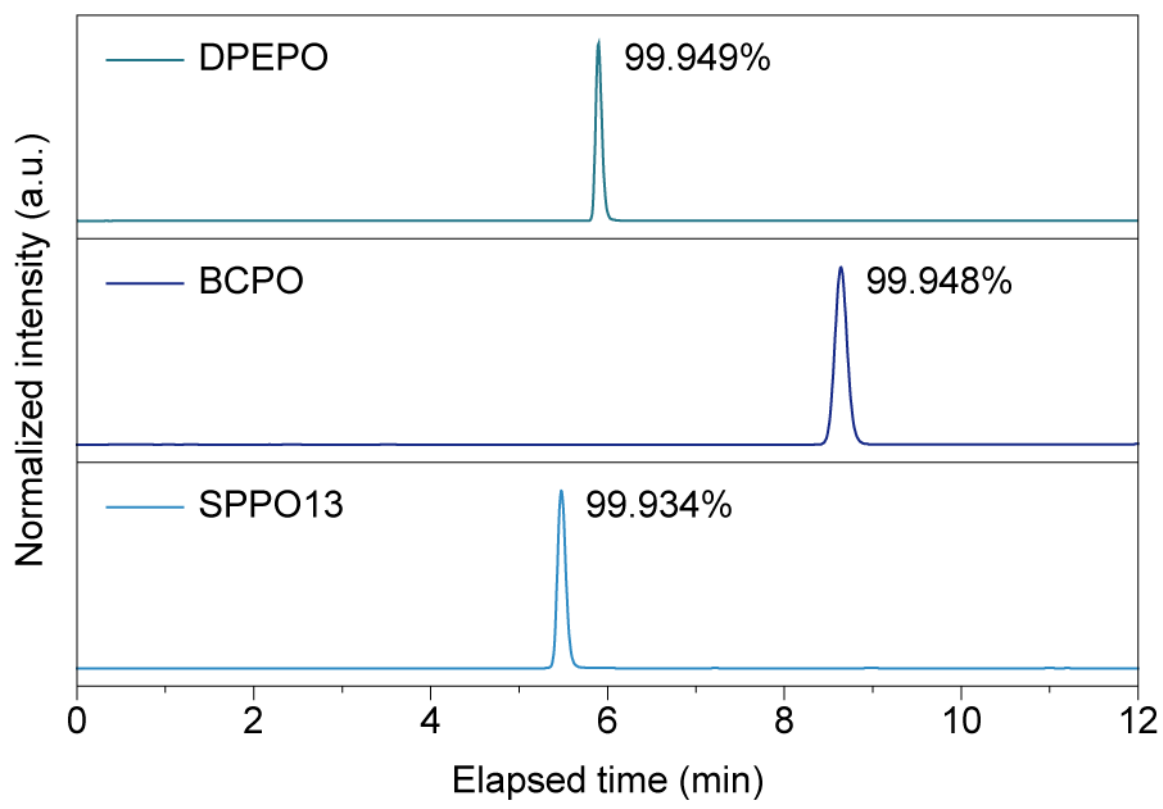

Supplementary Figure 51 | HPLC profiles of DPEPO, BCPO, and SPPO13 monitored at 254 nm. Temperature, 40°C; mobile phase, H_2O/MeCN/TFA; flow rate = 2.5 mL/min; column, ultimate XB-C18 (50 × 4.6 mm, 5 µm).

Comment #6: *The investigation into the properties of the host and guest materials is insufficient. The authors need to demonstrate whether the host and guest materials, when existing independently in powder or thin-film form, exhibit room-temperature phosphorescence.*

Response: We appreciate the reviewer's suggestion on the photophysical characterizations of the host and guest materials. Following your suggestion, we have measured the fluorescence (Flu.) and phosphorescence (Phos.) spectra of the guest R/S-PyBN in both powder and thin-film forms (**Supplementary Fig. 2, 3**). At 77 K, both the powder and neat films exhibit structured phosphorescence peaking at approximately 565 nm. At RT, the phosphorescence intensity decreases drastically owing to enhanced non-radiative relaxation, becoming weak in the film and undetectable in the powder. A comparison of the fluorescence and phosphorescence spectra of R/S-PyBN with those of R/S-PyBN@DPEPO (**Figs. 2a, 2d**) reveals that, although a blue shift arising from suppressed aggregation in the host–guest system is observed, the luminescence features of R/S-PyBN@DPEPO should be attributed to the guest molecules.

In addition, we have examined the fluorescence and phosphorescence spectra of the host DPEPO in powder and thin-film forms (**Supplementary Fig. 5**). In both morphologies, DPEPO shows no discernible phosphorescence; instead, it displays a delayed fluorescence peak that closely resembles its fluorescence spectra. This spectral feature is distinct from the phosphorescence observed in R/S-PyBN@DPEPO.

We sincerely thank the reviewer for this valuable suggestion. These additional photophysical data are essential for correctly establishing the origin of the luminescence in R/S-PyBN@DPEPO.

Actions: We have included these figures in the Supplementary Information and incorporated the necessary discussion into the revised manuscript.

*"For R/S-PyBN, blue fluorescence emission was observed in the toluene solution (5×10^{-5} mol/L), powder and neat film (**Supplementary Figs. 1-3**). Compared with the well-resolved structured emission of isolated molecules in toluene solution, the fluorescence of R/S-PyBN in powders and films featured a broad emission band with a pronounced red shift, which could be attributed to aggregation forms with stronger intermolecular interactions[56,57]. Meanwhile, the phosphorescence spectra of R/S-PyBN in both the powder and film forms displayed clear vibronic fine structures at 77 K, which are characteristics of radiative transitions from the local excited state (LE) excitons[58,59]."* (Page 2 of the revised manuscript, right column, lines 1-9)

*"In contrast, DPEPO, irrespective of in powder or neat film form, exhibited nearly identical fluorescence and delayed fluorescence bands either at 77 K or at RT (**Supplementary Fig. 5**)." (Page 2 of the revised manuscript, right column, lines 16-18)*

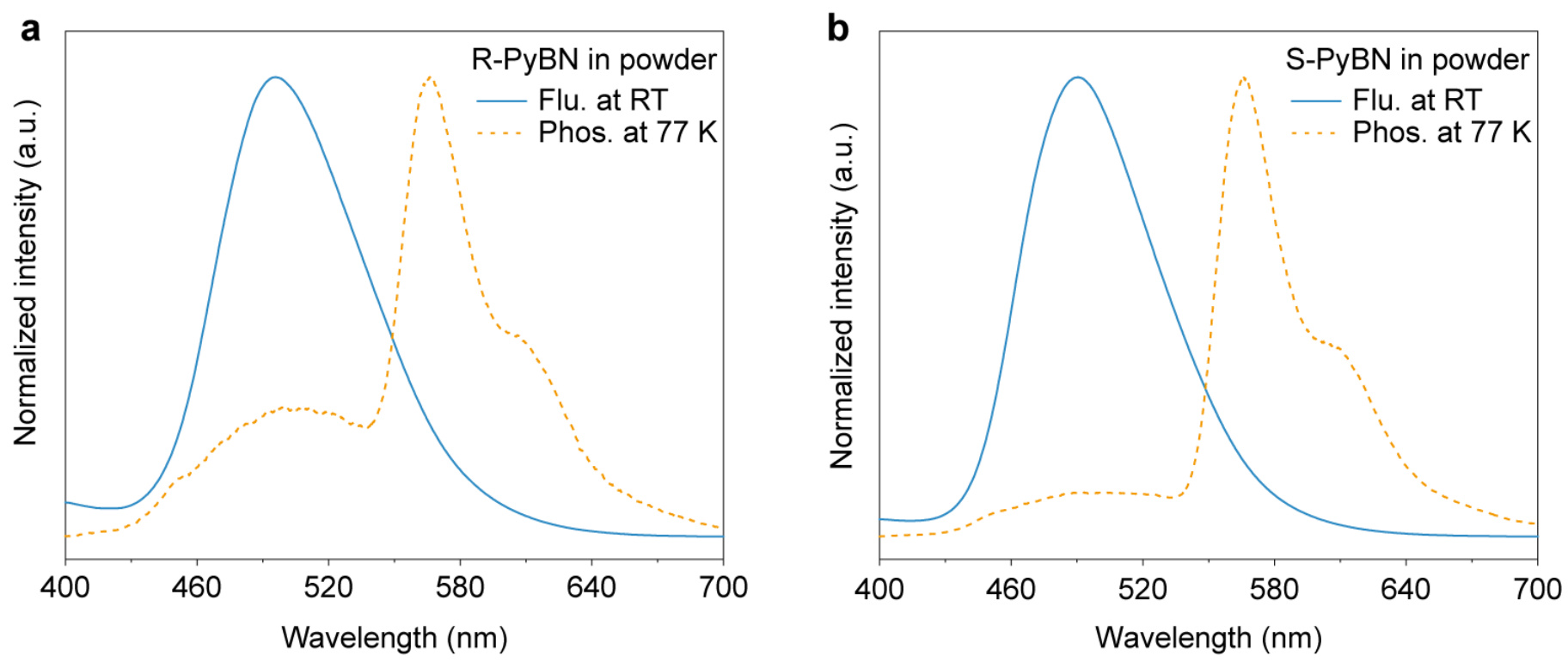

Supplementary Figure 2 | Fluorescence (Flu., at RT) and phosphorescence (Phos., delayed time 8 ms, at 77 K) spectra of R/S-PyBN in powder form. a, S-PyBN. b, S-PyBN.

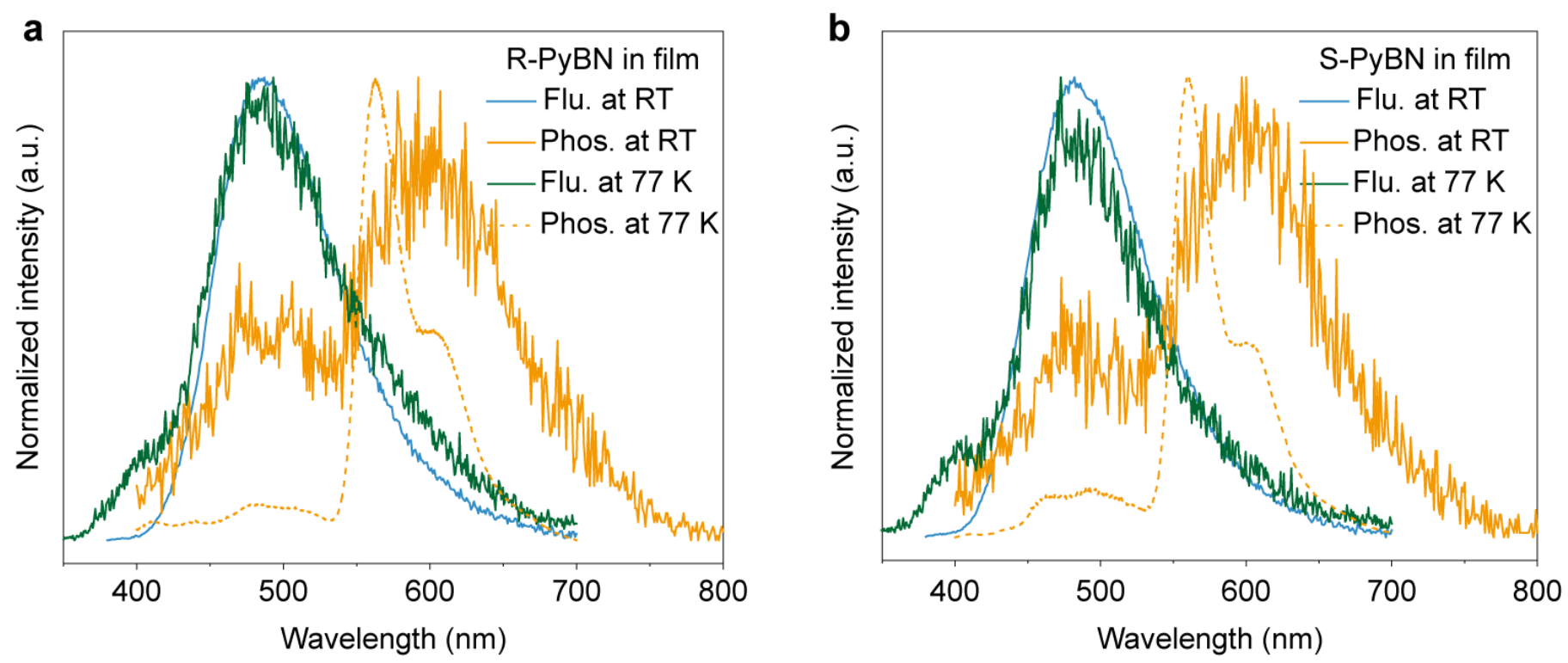

Supplementary Figure 3 | Fluorescence (Flu.) and phosphorescence (Phos.) spectra of R-PyBN (a) and S-PyBN (b) in film. The delayed time of phosphorescence was 100 μs at RT and 8 ms at 77 K.

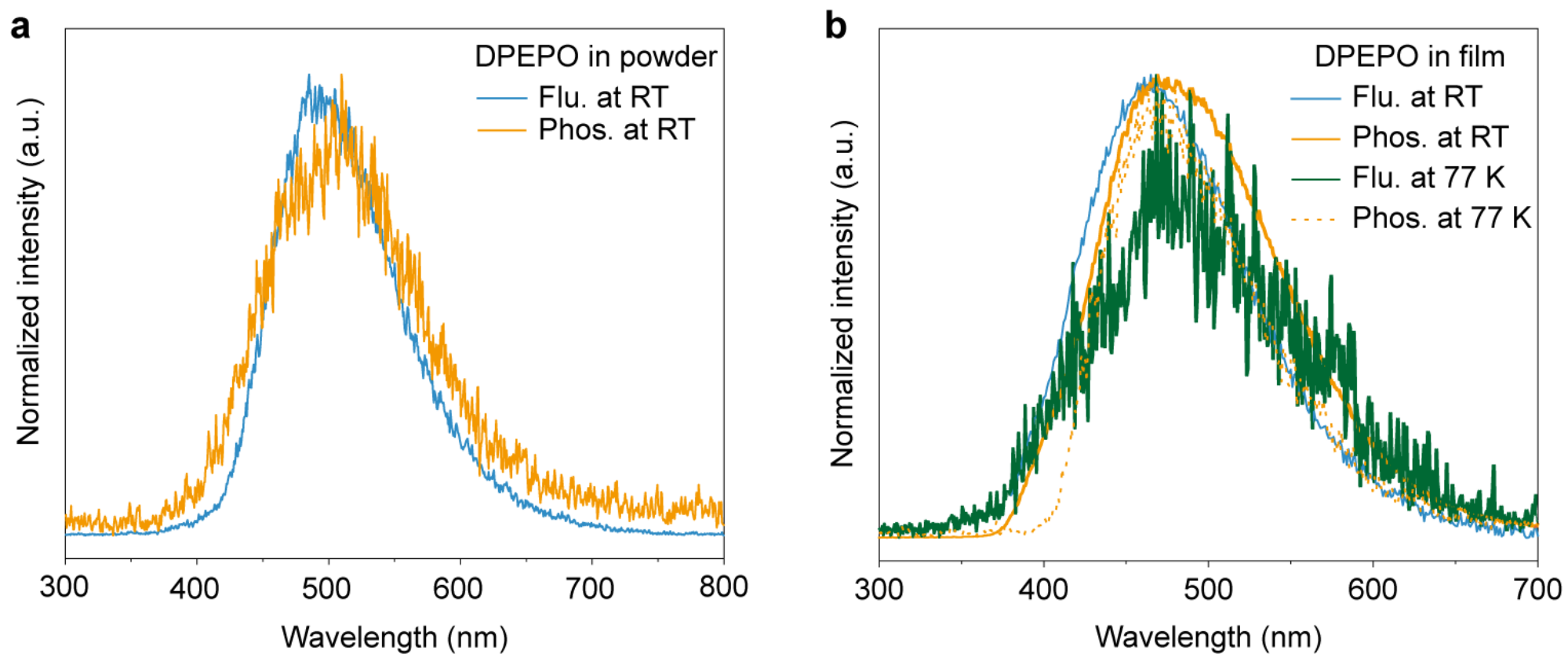

Supplementary Figure 5 | Fluorescence (Flu.) and phosphorescence (Phos.) spectra of DPEPO in powder (a) and in neat film (b). The delayed time of phosphorescence was 5 ms at RT and 8 ms at 77 K.

Comment #7: *The authors claim that the material exhibits persistent luminescence for over 4 hours at room temperature. However, in the text, there is only "The phosphorescence spectra of the R/S-PyBN@DPEPO showed two emission peaks at 531/531 and 572/572 nm, with lifetime of 2.72/5.34 and 3.87/3.73 ms at RT (Fig. 2a and Supplementary Fig. 14)." The decay lifetime should be scientifically measured by instrument but not by sense.*

Response: We thank the reviewer for this valuable comment regarding the PersL decay time. We sincerely apologize for the confusion that we may have caused. We wish to clarify that **the PersL decay time of R/S-PyBN@DPEPO (~4 h) after cessation of UV irradiation was measured by using a photomultiplier tube (PMT) and determined under a specific definition**. The PersL decay time is fundamentally different from the phosphorescence lifetime (typically in microsecond-to-second range) in terms of experimental methodology, lifetime definition, and absolute magnitude. To avoid further confusion, we have now provided the definitions of phosphorescence lifetime and PersL decay time in **Supplementary Figs. 1a and 1b** to clarify their differences.

(1) Phosphorescence lifetime is generally defined as the time when the emission intensity drops to 1/e of its initial intensity in the case of single-exponential fitting (*Chem. Soc. Rev.*, **2013**,*42*, 7834).

(2) In contrast, PersL decay does not follow exponential kinetics and **it has been defined in three different ways in the literature**. As shown in **Supplementary Fig. 1b**, $\boldsymbol{\tau_1}$ is defined as the time at which the PersL intensity decays to the absolute threshold of 0.32 mcd/m^2. This definition is widely used in the field of night emergency illumination of inorganic PersL phosphors (*J. Lumin.*, **2019**, *205*, 581). $\boldsymbol{\tau_2}$ is defined as the time at which intensity decays to 0.0032 mcd/m^2, corresponding to the visual perception threshold of dark-adapted human vision (*J. Electrochem. Soc.*, **1996**,*143*, 2670). $\boldsymbol{\tau_3}$ is defined as the time at which the PersL signal decays to the photodetector noise level (detection limit). Unlike $\boldsymbol{\tau_1}$ and $\boldsymbol{\tau_2}$, the definition of $\boldsymbol{\tau_3}$ does not rely on absolute luminance, rendering it susceptible to detector sensitivity and ambient noise. It represents a non-quantitative metric. Nevertheless, because precise determination of $\boldsymbol{\tau_1}$ and $\boldsymbol{\tau_2}$ requires strict standardization of sample dimensions and excitation intensity, $\boldsymbol{\tau_3}$ **is currently the widely adopted method in organic PersL research when such stringent conditions cannot be met** (*Nature*, **2017**, *550,* 384; *Adv. Mater.*, **2020**, *32*, 2001026). We hope that the above explanation adequately clarifies the definition of PersL decay time.

Actions: We have incorporated the definitions of phosphorescence lifetime and PersL decay into the Supplementary Information and added corresponding clarifications to the manuscript.

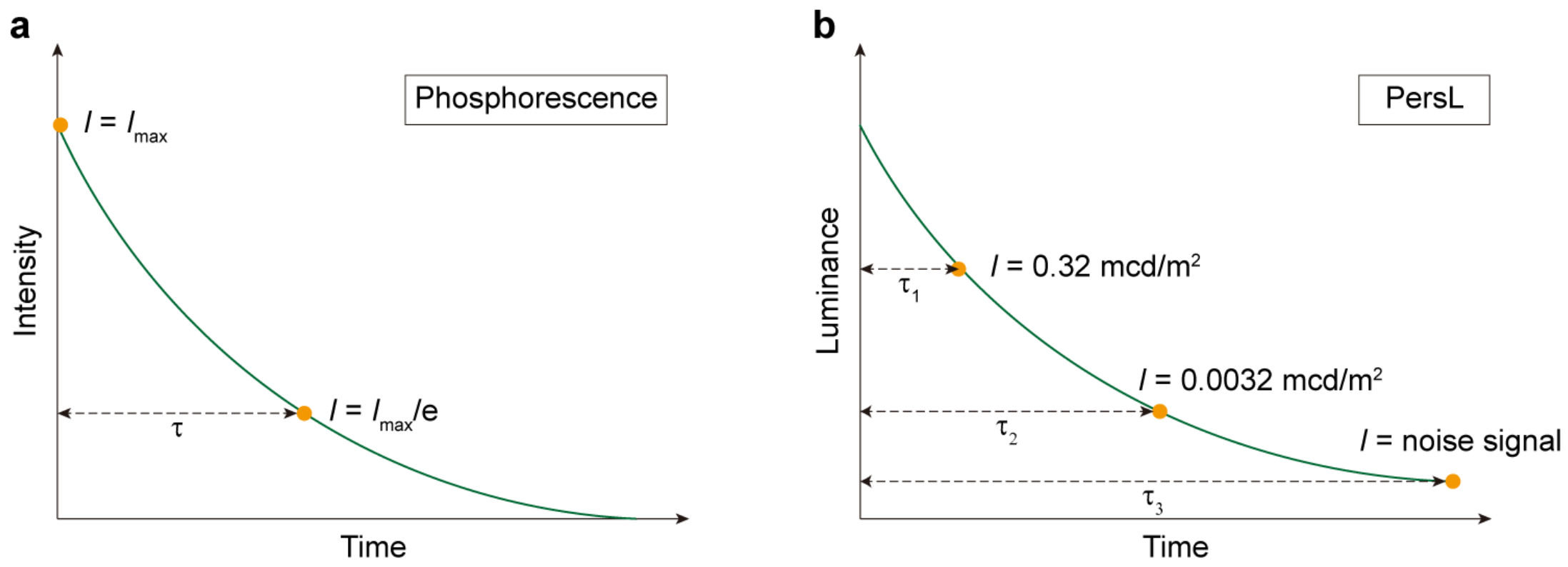

Response Figure 1 | Different definitions of decay time between phosphorescence and PersL. a, Schematic illustration of the definition of phosphorescence lifetime. The phosphorescence lifetime is generally defined as the time when the emission intensity drops to 1/e of the initial intensity in the case of single-exponential fitting. **b**, Schematic illustration of the definition of PersL lifetimes. The PersL lifetimes can be defined as the time when the emission intensity drops to 0.32 mcd/m^2 (τ_1), 0.0032 mcd/m^2 (τ_2) and noise level of photodetector (τ_3).

Comment #8: *White emission has a wide application in light. White emission has no advantage for the application in optical information encryption.*

Response and Actions: We thank the reviewer for this constructive comment. As the reviewer noted, from a purely spectroscopic perspective, white light indeed offers no inherent advantage over monochromatic light for optical information encryption. In this study, we demonstrated an encryption strategy based on multimodal luminescence properties, including fluorescence, phosphorescence, PersL, and CPL. Among these modalities, **significant spectral changes (*e.g.,* from blue fluorescence to yellow phosphorescence and subsequently to white PersL) provide more intricate and distinctive optical signatures, which increase information capacity and encryption complexity.** However, we fully acknowledge that the multimodal luminescence properties exhibited in this work remain far from practical applications. Accordingly, we have revised the relevant descriptions as **proof-of-concept demonstrations** in the revised manuscript to reflect this limitation.

Comment #9: *White emission is usually composed of blue+yellow emission or blue+green+red emission. The efficiency of white emission from ultraviolet+yellow emission is often low with poor CIE and CRI (color rendering index). What are the advantages of this system?*

Response: We sincerely thank the reviewer for this valuable comment. We fully agree that a high CRI and luminous efficacy are essential for solid-state lighting. **Although the dual-band white PersL presented in this work deviates from the ideal white point, its substantial spectral change from single-band phosphorescence is sufficient for the proof-of-concept optical information encryption application**.

Motivated by this comment, we also recognize that enhancing the CRI of white organic PersL through spectral modulation would significantly broaden its practical applicability. By incorporating guest molecules with a lower singlet (S_1) energy level, the dual-band white PersL strategy proposed here is expected to yield improved blue + yellow emission, thereby achieving more ideal white light. We hope that the clarification addresses the reviewer's concern.

Comment #10: *Fig. 3d is ESR spectra recorded after UV irradiation at 110 K (green curves) and 300 K (orange curves). The authors contribute it to the formation of radicals with unpaired electrons. Since the persistent luminescence comes from triplet, which also has unpaired electrons, the authors need to conduct more experiments to rule out this possibility.*

Response: We sincerely thank the reviewer for the insightful suggestion. We agree that unpaired electrons associated with the triplet state can also give rise to ESR signals. To exclude this possibility, we exploited the difference in timescales to distinguish between transient triplet signals and long-lived radical signals.

Theoretically, triplet-state electrons in pure organic systems decay on timescales from microseconds to seconds, whereas trapped radicals are expected to remain stable for hours. Accordingly, we monitored the ESR spectra of R/S-PyBN@DPEPO after 365-nm excitation over storage periods from 10 minutes to 28 days (**Supplementary Fig. 21**). Although the signal intensity gradually decreased over time, the ESR signals remained detectable after 28 days at RT. These results indicate that the signals should originate predominantly from long-lived stable radicals rather than from short-lived triplet-state electrons.

Actions: We apologize for the ambiguity in the previous manuscript regarding the origin of the ESR signals. In response to the reviewer's suggestion, we have now included comprehensive ESR measurements in the Supplementary Information and added the corresponding clarification to the revised manuscript.

*"To exclude potential interference from short-lived triplet electrons, the ESR signals of R/S-PyBN@DPEPO were recorded at various storage time (from 10 min to 28 days) at RT. The ESR signals decayed slowly over time, yet remained detectable after 28 days at RT (**Supplementary Fig. 21**). The above results confirm the formation of radicals with unpaired electrons upon UV irradiation and their long-term persistence at RT."* (Page 5 of the revised manuscript, left column, lines 51-55, and right column, lines 1-2)

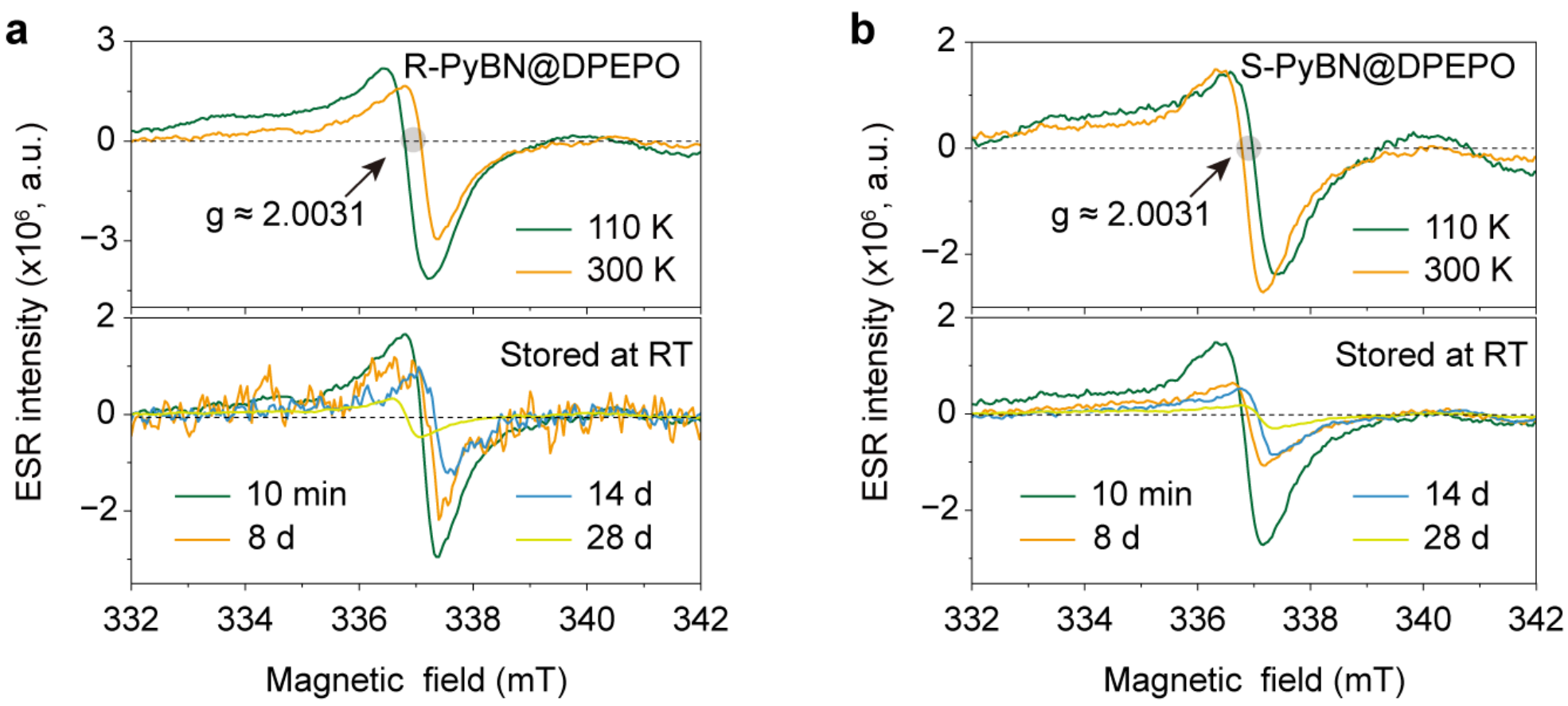

Supplementary Figure 21 | ESR spectra of 1 wt% R-PyBN@DPEPO (a) and S-PyBN@DPEPO (b) films recorded after UV irradiation at 110 and 300 K (top) and after 365 nm excitation at different storage time (bottom, from 10 minutes to 28 days).

Point-by-Point Response to Referees

Editorial Note:

Reviewer 3 still has a couple of ongoing concerns but we feel that they could be addressed by incorporating some additional discussion into the manuscript, potentially regarding the further studies that need to be undertaken in the future. Please clarify the relative emission peaks and lifetimes.

Response: We are grateful to the editor for the positive assessment of our work. Regarding the final comment from Reviewer 3, we fully appreciate their concert. Nevertheless, we wish to emphasize that the central advance of this work lies in achieving dual-band PersL lasting for hours through the redistribution of released electrons, rather than via phosphorescence (which indeed exhibits a decay time of less than 1s). In the revised manuscript, we have incorporated clarifications that rigorously distinguish the definitions and experimental methodologies for phosphorescence lifetime and PersL decay duration in the relevant sections, and have added a brief discussion of future research directions in the Conclusions.

Actions: We have incorporated additional discussion on phosphorescence lifetime and PersL decay, as well as further studies that need to be undertaken in the future.

*"Notably, the phosphorescence spectra of the R/S-PyBN@DPEPO films retained vibronic fine structures consistent with those of the neat guest films, showing two emission peaks at 531/531 and 572/572 nm. The corresponding lifetimes measured at RT under microsecond pulsed-lamp excitation were 701.33/677.07 ms and 762.13/716.39 ms, respectively, as determined from exponential fits by the time required for the emission intensity to decrease to 1/e of its initial value (**Supplementary Fig. 11** and **Supplementary Table 2**)."* (Page 4 of the revised manuscript, left column, lines 10-17)

*"The R/S-PyBN@DPEPO exhibited long-lasting emissions after continuous excitation for 1 min, which could be detected with a photomultiplier tube (PMT) for over 4 h before the signal attenuated to the noise level of detector (**Fig. 2c**)."* (Page 4 of the revised manuscript, right column, lines 6-9)

"The dual emission of PersL from triplet–singlet and singlet–singlet transitions in this work expands the material family of organic CP-PersL and provides a theoretical framework for developing organic emitters with lifetimes including millisecond/second-level phosphorescence and hour-level PersL. The differentiation and spectral tuning of two distinct long-lived luminescence types coexisting within a material system hold considerable promise for organic luminescent materials, although the performance demonstrated herein still warrants improvement. Future molecular engineering of chiral guest molecules with superior CPL performance and lower singlet energy levels may further enhance the g_{lum} values and achieve a more ideal white-light emission, potentially opening new avenues for optical information storage and anti-counterfeiting." (Page 8 of the revised manuscript, right column, lines 1-13)

Referee #1:

The authors have responded to all the requests and critical issues raised by this reviewer. Following the proposed revision, the manuscript has reached the standards required for publication and deserves to be published in its present form.

Response: Thank you very much for your recommendation. We appreciate your suggestion for us in the previous review to improve the quality of this manuscript.

Referee #2:

Authors have carefully revised this manuscript according to reviewers' comments. The revised manuscript can be accepted for publication in Nature Communication.

Response: We appreciate the reviewer's suggestion and recognition of our work. All your suggestions help us a lot to

improve the manuscript.

Referee #3:

The phosphorescence spectra of the R/S-PyBN@DPEPO showed two emission peaks at531/531 and 572/572 nm, with lifetime of 2.72/5.34 and 3.87/3.73 ms at RT (Fig. 2a and Supplementary Fig. 14). If the phosphorescence lifetime is not 4 h but much lower than 1 s, I think the innovation of this manuscript is low and does not meet the publication standards of Nature Communications.

Response: We thank the reviewer for this valuable comment regarding the PersL decay time. We sincerely apologize that our revisions have not fully met your expectations and for any confusion this may have caused. Indeed, as we clarified in the previous Response Letter, the PersL decay time is fundamentally different from the phosphorescence lifetime (typically in microsecond-to-second range) in terms of experimental methodology, lifetime definition, and absolute magnitude. Phosphorescence lifetime is generally defined as the time when the emission intensity drops to 1/e of its initial intensity in the case of single-exponential fitting. The phosphorescence spectra of R/S-PyBN@DPEPO show two emission peaks at 531/531 and 572/572 nm, with corresponding lifetimes measured at RT **under microsecond pulsed-lamp excitation** of **701.33/677.07 ms** and **762.13/716.39 ms**, respectively. By contrast, after **continuous excitation for 1 min**, the PersL emission lasted for over 4 h before it decayed to the noise level of PMT. **We believe that the central advance of this work lies in achieving dual-band PersL lasting for hours through the repopulation of released electrons, rather than via phosphorescence (which indeed exhibits a decay time of less than 1s).** To avoid misunderstanding to readers, we have added the definitions of phosphorescence lifetime and PersL decay duration to the revised manuscript to clarify their differences.

Actions: We have incorporated the definitions of phosphorescence lifetime and PersL decay into the manuscript.

*"Notably, the phosphorescence spectra of the R/S-PyBN@DPEPO films retained vibronic fine structures consistent with those of the neat guest films, showing two emission peaks at 531/531 and 572/572 nm. The corresponding lifetimes measured at RT under microsecond pulsed-lamp excitation were 701.33/677.07 ms and 762.13/716.39 ms, respectively, as determined from exponential fits by the time required for the emission intensity to decrease to 1/e of its initial value (**Supplementary Fig. 11** and **Supplementary Table 2**)."* (Page 4 of the revised manuscript, left column, lines 10-17)

*"The R/S-PyBN@DPEPO exhibited long-lasting emissions after continuous excitation for 1 min, which could be detected with a photomultiplier tube (PMT) for over 4 h before the signal attenuated to the noise level of detector (**Fig. 2c**)."* (Page 4 of the revised manuscript, right column, lines 6-9)
